# Design, synthesis and application of a new type of bifunctional **Le-Phos** in highly enantioselective γ-addition reactions of N-centered nucleophiles to allenoates†
†Electronic supplementary information (ESI) available. CCDC 1819863, 1819864, 1819865 and 1860469. For ESI and crystallographic data in CIF or other electronic format see DOI: 10.1039/c9sc04073k


**DOI:** 10.1039/c9sc04073k

**Published:** 2019-09-03

**Authors:** Haile Qiu, Xiaofeng Chen, Junliang Zhang

**Affiliations:** a Shanghai Key Laboratory of Green Chemistry and Chemical Processes , School of Chemistry and Molecular Engineering , East China Normal University , 3663 N. Zhongshan Road , Shanghai , P. R. China (200062) . Email: jlzhang@chem.ecnu.edu.cn; b Department of Chemistry , Fudan University , 2005 Songhu Road , Shanghai , P. R. China (200438) . Email: junliangzhang@fudan.edu.cn

## Abstract

A novel class of cyclic phosphine derived bifunctional catalysts (**Le-Phos**) is reported, which can be readily prepared from inexpensive and commercially available starting materials and exhibit good performances in enantioselective γ-addition reactions of N-centered nucleophiles and allenoates under mild conditions. The salient features of this reaction include high product yields, good enantioselectivity, mild reaction conditions, and broad substrate scope and gram-scale scalability.

## Abstract 2

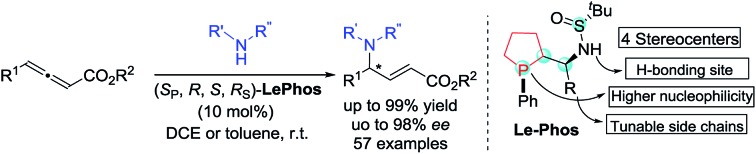
A novel class of bifunctional cyclic phosphine catalysts (**Le-Phos**) is reported, which showed good performances in enantioselective γ-addition reactions of N-centered nucleophiles and allenoates under mild conditions.

## Introduction

**Fig. 1:**
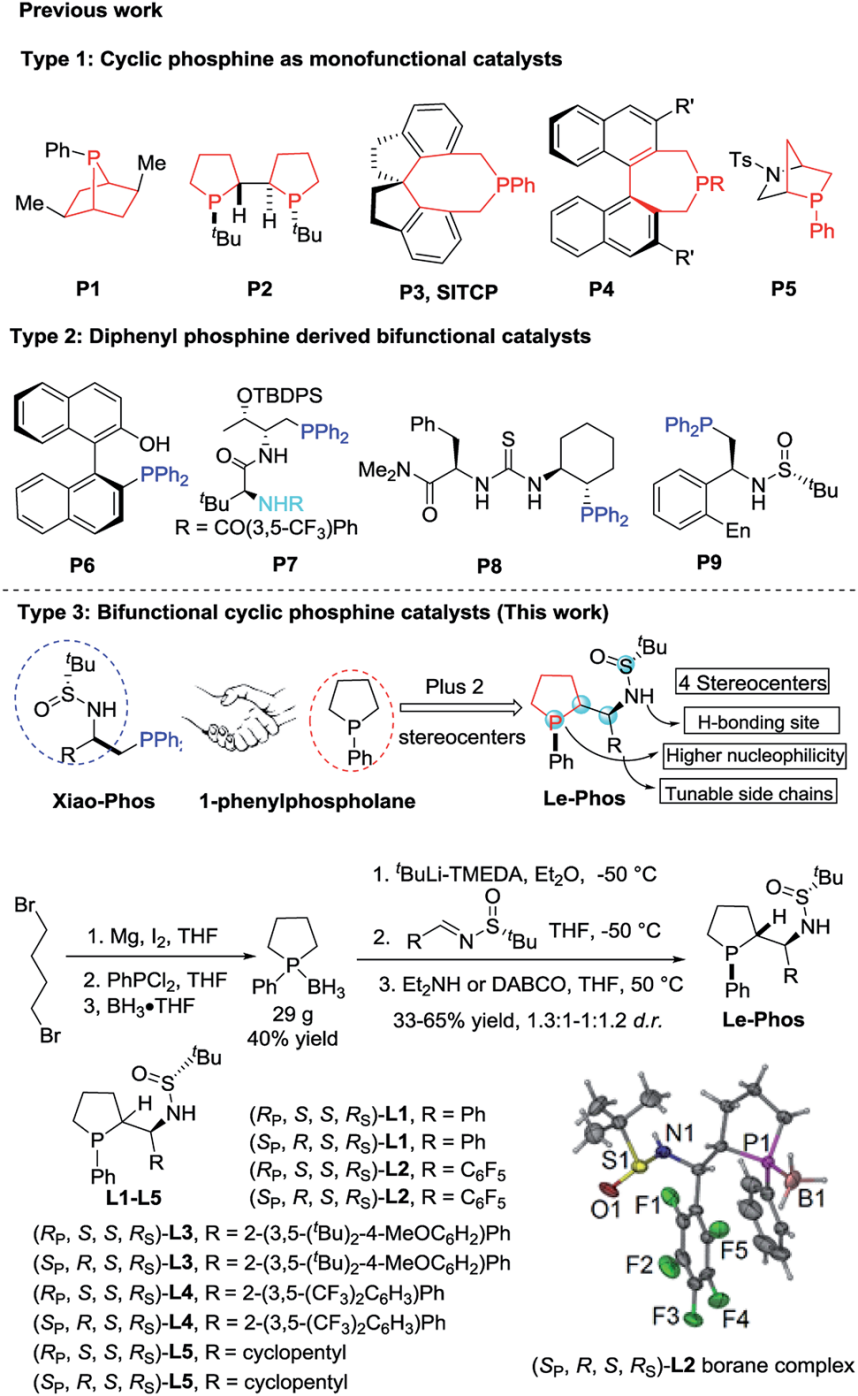
Different types of chiral phosphine catalysts.

Over the past few years, asymmetric phosphine-catalyzed reactions have emerged as powerful and versatile tools for the construction of C–C and C–X bonds,^1^ which relies very much on the evolution of various new chiral phosphine catalysts.^2^ There are mainly two types of chiral phosphine catalysts developed: highly nucleophilic monofunctional phosphine catalysts such as cyclic phosphines **P1–P5** (Fig. 1, Type 1) and diphenylphosphine-derived bifunctional catalysts bearing a hydrogen donor such as **P6–P9** (Fig. 1, Type 2). Both displayed good catalytic activities and were effective in enantiomeric control in asymmetric phosphine catalysis.1a,g,^3^ Recently, we developed several novel diphenylphosphine-derived bifunctional phosphines from commercially available chiral sulfinamide.^4^ To further advance a new catalyst design, we aimed to combine the advantages of the aforementioned two types of phosphine catalysts, thus developing a novel bifunctional cyclic phosphine catalyst. We report herein the design and synthesis of **Le-Phos**, and its application in highly enantioselective phosphine catalyzed γ-addition of N-centered nucleophiles to allenoates.

## Results and discussion

Fortunately, we found that **Le-Phos** could be easily prepared from commercially available inexpensive *tert*-butylsulfinamide, aldehyde and 1-phenylphospholane borane complexes in simple steps. Treatment of 1-phenylphospholane borane complexes^5^ with ^*t*^BuLi in the presence of TMEDA at –50 °C for 4 h gave the lithium intermediate, which added to chiral (*R*_S_)-sulfinimines, furnishing a pair of major diastereomers of **Le-PhosL1–L5** in 33–65% total yields after removal of borane.^6^ To our delight, these two major diastereoisomers could be separated by flash column chromatography on silica gel. The absolute configurations of (*R*_P_,*S*,*S*,*R*_S_)-**L2** and (*S*_P_,*R*,*S*,*R*_S_)-**L2** were established by single crystal X-ray diffraction analysis.^7^

Asymmetric phosphine-catalyzed γ-addition reactions of various nucleophiles to allenoates have attracted much attention in the past few years.^8^–^10^ In 1998, Zhang and co-workers reported the catalyzed asymmetric γ-addition of 1,3-dicarbonyl compounds to terminal allenoates using bicyclic phosphine **P2** for the first time.^9^ Furthermore, Fu, Jacobsen, Lu and our groups have successfully expanded the scope of nucleophiles such as alcohols, thiols, carbon, amides and ketimines by the employment of different types of phosphine catalysts.^10^ The asymmetric γ-addition^8^–^11^ of N-centered nucleophiles with p*K*_a_ values between 8 and 10 (in H_2_O) to γ-substituted allenoates has been only partially realized by the group of Jacobsen, in which **P8** was used as the catalyst.10*n* Very recently, Guo and coworkers successfully extended N-centered nucleophiles to pyrazoles and imidazoles with the use of (*S*)-SITCP and (*S*)-BINOL as cocatalysts.^13^ However, there still lacks a robust catalyst system for the asymmetric γ-addition of various N-centered nucleophiles to allenoates. For example, (*S*)-SITCP, **P8** and our developed Xiao-Phos **P9** could not yield satisfactory results for the asymmetric γ-addition of 2-oxazolidone **1a** to allenoate **2a** (Table 1, entries 1–3). Interestingly, (*S*_P_,*R*,*S*,*R*_S_)-**L1–L4** showed much higher catalytic activity and much better enantioselectivity than their diastereoisomers (*R*_P_,*S*,*S*,*R*_S_)-**L1–L4** (Table 1, entries 4–11). To our delight, 54% yield of **3aa** with 97% ee and *E*/*Z* > 20 : 1 could be achieved with the use of (*S*_P_,*R*,*S*,*R*_S_)-**L4** (Table 1, entry 11). Due to the competitive isomerization and partial kinetic resolution,10*l* increasing allenoate **2a** to two equivalents could improve the 68% yield (Table 1, entry 13). Changing the solvent from toluene to PhCF_3_, DCM and DCE led to around 90% yield with 96–97% ees (Table 1, entries 14–17).

**Table 1:** Screening reaction conditions^*a*^

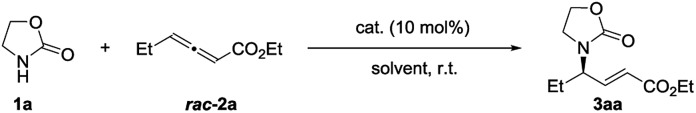					
Entry	Catalyst	Solvent	*E*/*Z*^*b*^	Yield^*b*^ (%)	ee^*c*^ (%)
1	(*S*)-SITCP	Toluene	5 : 1	39	87
2	**P8**	Toluene	4 : 1	11	72
3	**P9**	Toluene	2 : 1	21	57
4	(*R*_P_,*S*,*S*,*R*_S_)-**L1**	Toluene	3 : 1	7	19
5	(*R*_P_,*S*,*S*,*R*_S_)-**L2**	Toluene	—	NR	—
6	(*R*_P_,*S*,*S*,*R*_S_)-**L3**	Toluene	2 : 1	5	46
7	(*R*_P_,*S*,*S*,*R*_S_)-**L4**	Toluene	2 : 1	9	11
8	(*S*_P_,*R*,*S*,*R*_S_)-**L1**	Toluene	>20 : 1	40	86
9	(*S*_P_,*R*,*S*,*R*_S_)-**L2**	Toluene	>20 : 1	10	69
10	(*S*_P_,*R*,*S*,*R*_S_)-**L3**	Toluene	>20 : 1	46	97
11	(*S*_P_,*R*,*S*,*R*_S_)-**L4**	Toluene	>20 : 1	54	97
12^*d*^	(*S*_P_,*R*,*S*,*R*_S_)-**L4**	Toluene	>20 : 1	60	97
13^*e*^	(*S*_P_,*R*,*S*,*R*_S_)-**L4**	Toluene	>20 : 1	68	97
14^*e*^	(*S*_P_,*R*,*S*,*R*_S_)-**L4**	Et_2_O	>20 : 1	60	97
15^*e*^	(*S*_P_,*R*,*S*,*R*_S_)-**L4**	PhCF_3_	>20 : 1	90	97
16^*e*^	(*S*_P_,*R*,*S*,*R*_S_)-**L4**	DCM	>20 : 1	89	96
17^*e*^	(*S*_P_,*R*,*S*,*R*_S_)-**L4**	DCE	>20 : 1	90	97

^*a*^Reaction conditions: **1a** (0.10 mmol), **2a** (0.12 mmol), and the catalyst (0.01 mmol) in toluene (1.5 mL) at room temperature.

^*b*^NMR yield with the use of CH_2_Br_2_ as the internal standard.

^*c*^Determined by HPLC analysis on a chiral stationary phase.

^*d*^Performed with **2a** (0.15 mmol).

^*e*^Performed with **2a** (0.20 mmol). DCM = dichloromethane, DCE = 1,2-dichloroethane.

**Scheme 1:**
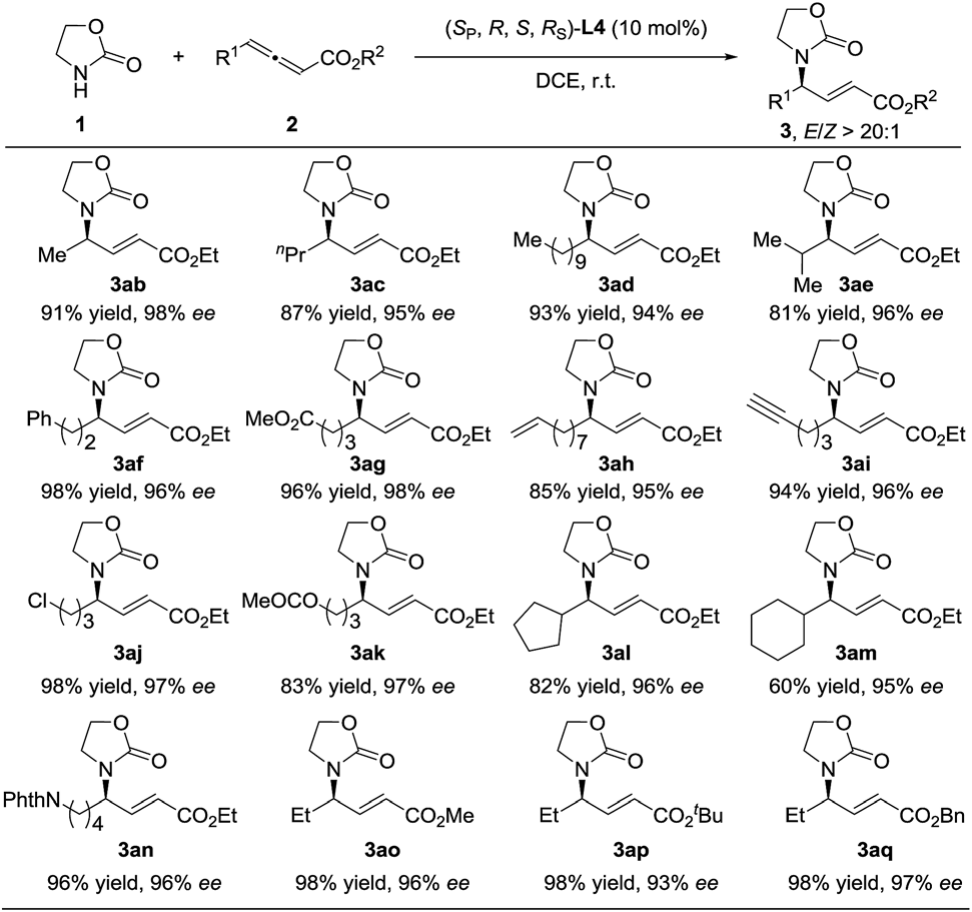
Investigation of the scope by variation of the allenoate component.

Having identified the optimal reaction conditions, the substrate scope was then examined and it proved to be quite general (Scheme 1). Linear alkyl (**3ab–3ad**), branched alkyl (**3ae**), and various alkyl groups bearing functional groups such as phenyl (**3af**), esters (**3ag** and **3ak**), terminal alkenes and alkynyl (**3ah–3ai**), and halogen (**3aj**) were well tolerated and provided high levels of yields and enantioselectivities (94–98% ees). Cyclic alkyl groups such as cyclopentyl (**3al**), cyclohexyl (**3am**), and NPhth groups (**3an**) could also be well compatible, delivering the corresponding adducts in high yields with 95–96% ees. It seems that the ester moiety did not affect the reaction much, furnishing **3ao–3aq** in high yields with 93–97% ees and *E*/*Z* > 20 : 1.

**Scheme 2:**
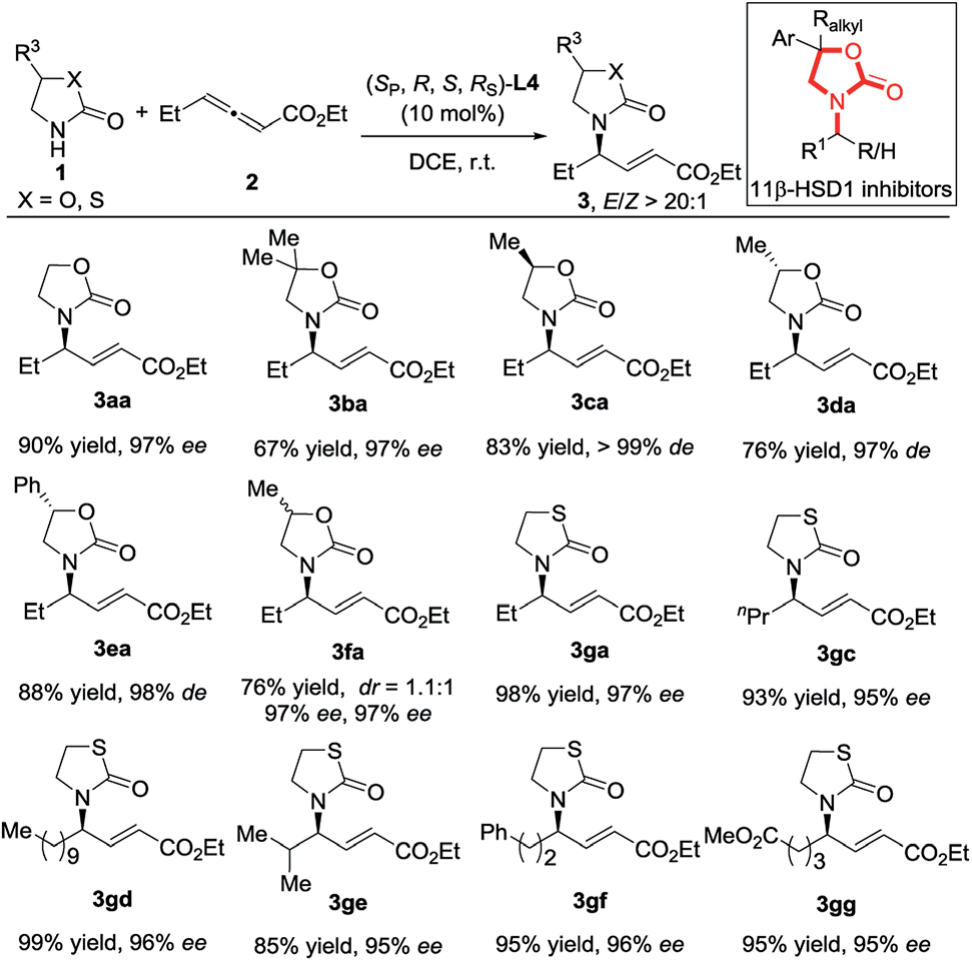
Investigation of the scope by variation of 2-oxazolidone.

The reactions of chiral 2-oxazolidones also proceeded well, delivering **3ca–3ea** in satisfactory yields with high *des* and *E*/*Z* > 20 : 1 (Scheme 2). The addition of racemic 2-oxazolidone **1f** did not show good diastereoselectivity but still delivered high enantioselectivity. Then, the reactions of thiazolidin-2-one (p*K*_a_ ∼ 12.8) with various allenoates also proceeded smoothly, furnishing products **3ga** and **3gc–3gg** in 85–99% yields with 95–96% ees. It should be pointed out that these products share the same skeleton with patented 11β-HSD1 inhibitors (11β-hydroxysteroid dehydrogenase type 1 inhibitors).^12^

**Scheme 3:**
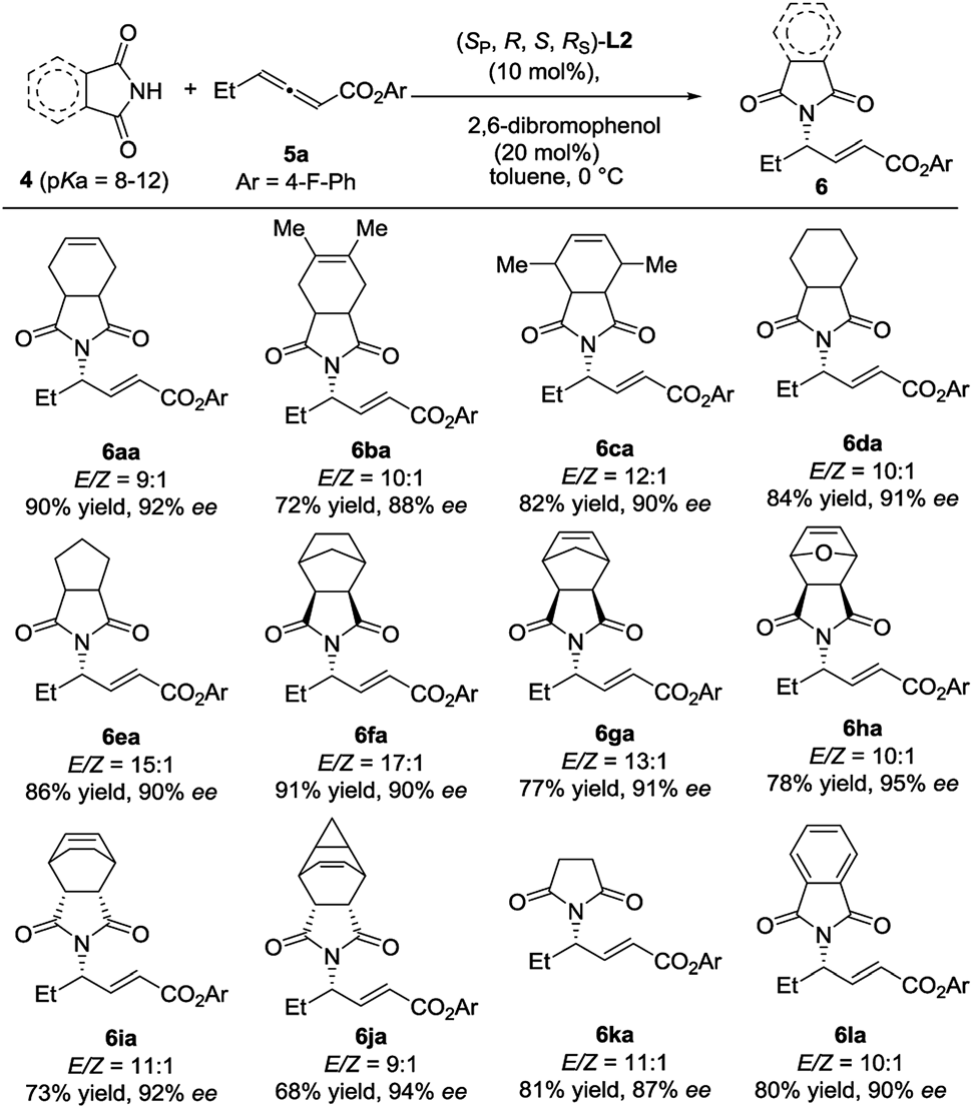
Investigation of the scope of pyrrolidine-2,5-diones.

The scope of N-centered nucleophiles was then extended to much weak nucleophilic pyrrolidine-2,5-diones (Scheme 3). In this case, (*S*_P_,*R*,*S*,*R*_S_)-**L2** was found to be the most efficient catalyst, indicating that the reaction is quite sensitive to the structure of N-centered nucleophiles, which further supports that the development of new catalysts with structural diversity is quite important. The reactions of various substituted pyrrolidine-2,5-diones with **5a** delivered the desired γ-addition adducts in 68–91% yields with 87–94% ees. The absolute configuration of **6ba** was established by single crystal X-ray diffraction analysis.^7^ It is interesting to find that the absolute configuration of **6ba** is different from that of compound **3**; despite this, the catalysts have the same absolute configuration.

**Scheme 4:**
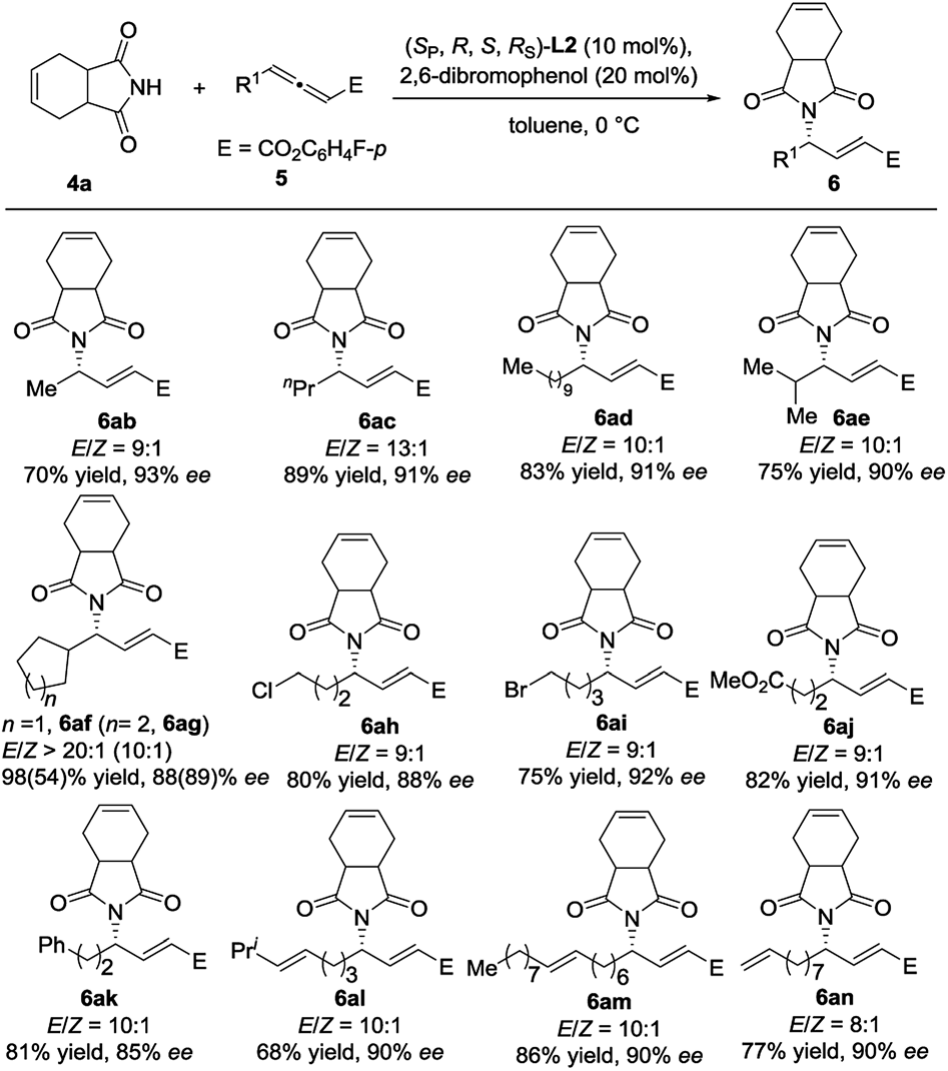
Investigation of the scope by variation of the allenoate component.

We next examined the reaction scope with respect to the allenoate component (Scheme 4). A variety of γ-substituted allenoates (R^1^) were applicable to this asymmetric γ-addition. In general, both linear and branched cycloalkyl groups at the γ-position were well tolerated. For example, allenoates **5b–5g** with various acyclic and cyclic alkyl groups at the γ-position could be well compatible, and the desired adducts were obtained in high yields with up to 93% ee. Satisfactorily, various functional groups such as halogens (**5h** and **5i**), ester (**5j**), phenyl (**5k**), and terminal and internal alkenes (**5l–5n**) were well tolerated and the desired adducts were obtained in moderate to good yields with up to 92% ee and >20 : 1 *E*/*Z* selectivity.

Additionally, the additions of TsNH_2_ (p*K*_a_ ∼ 10.2), PhSO_2_NH_2_ (p*K*_a_ ∼ 10.1), (BocNH)_2_ (p*K*_a_ ∼ 8.7) and pyrazole (p*K*_a_ ∼ 2.5)^13^ also proceeded smoothly under the catalysis of **Le-Phos** with different R groups (eqn (1)–(3)).
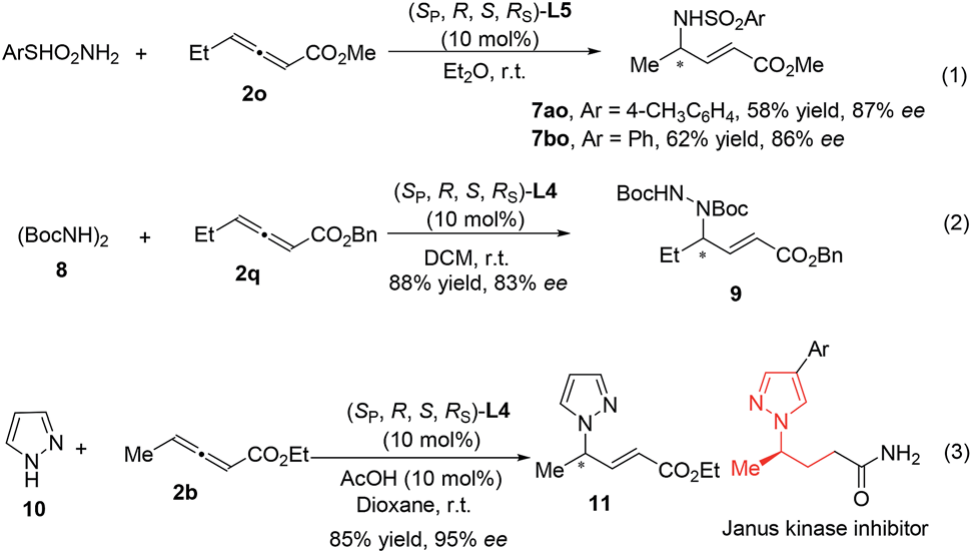


**Scheme 5:**
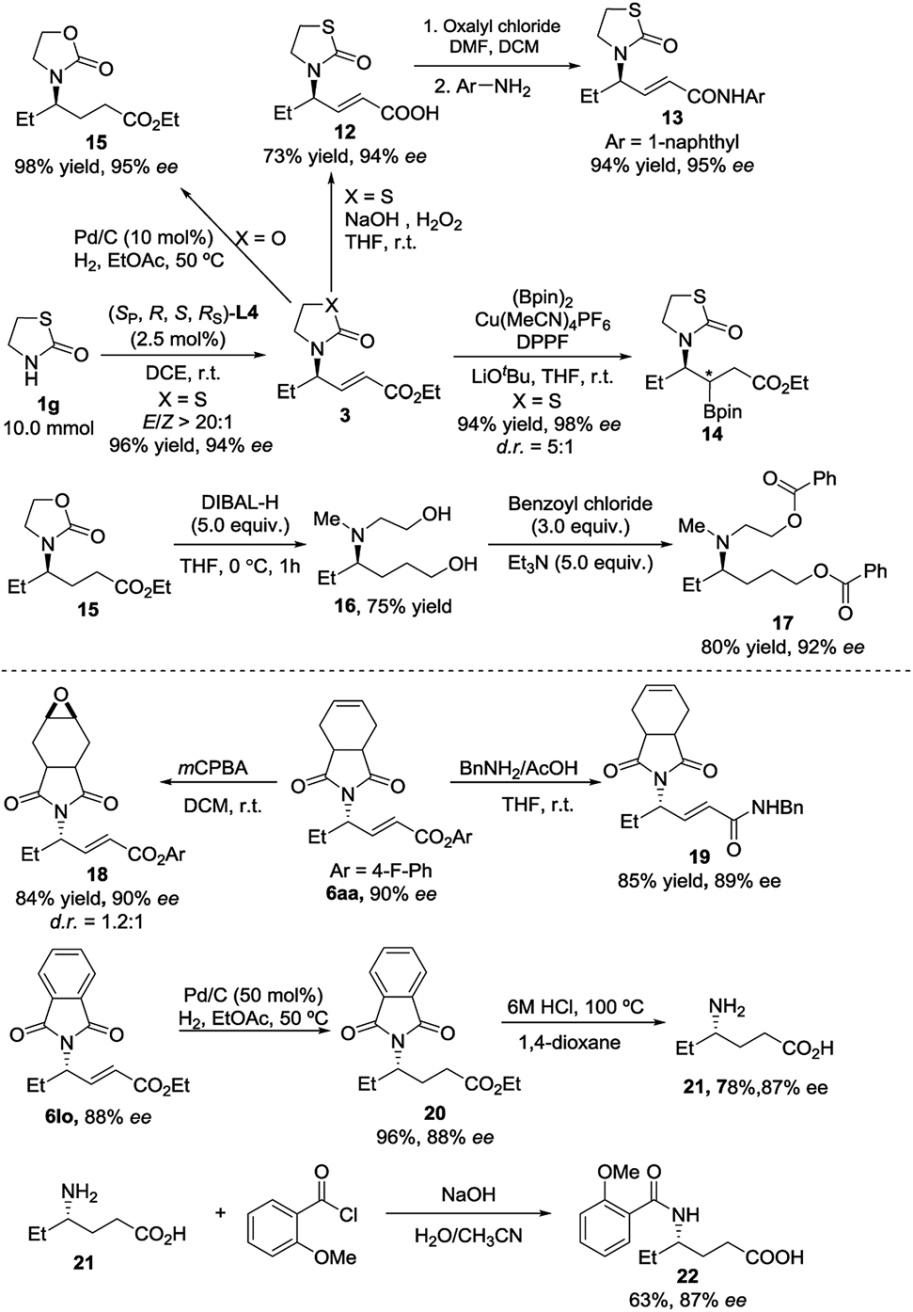
Elaboration of γ-addition adducts.

We were pleased to find that the desired product **3ga** could be obtained in 96% yield, 94% ee and *E*/*Z* > 20 : 1 with only 2.5 mol% catalyst loading on a 10 mmol scale (Scheme 5). The synthetic utilities of the representative product **3ga** were then showcased. The hydrolysis of the ester moiety was realized with NaOH/H_2_O_2_ 14 to give acid **12** in 73% yield without loss of enantioselectivity. The corresponding amide **13**^7^ could be further delivered in 94% yield with 95% ee. The copper-catalyzed conjugate borylation of **3ga** proceeded smoothly at room temperature, furnishing the desired product **14** in 94% yield with 98% ee and 5 : 1 d.r.^15^ Reduction of the double bond furnished the product **15** in 98% yield with 95% ee. Moreover, we could obtain an amino alcohol derivative **16** through reductive ring-opening of **15**, which afforded the diester **17** after further esterification. Furthermore, with the use of *m*CPBA,^16^ the C–C double bond of **6aa** would undergo epoxidation to deliver the corresponding product **18** in good yield without loss of the enantioselectivity. The amidation reaction of **6aa** with BnNH_2_/AcOH^17^ proceeded smoothly at room temperature, delivering the corresponding amide **19** in 85% yield with 89% ee. The reduction of the double bond of **6lo** was achieved *via* the Pd/C-catalyzed hydrogenation, furnishing product **20** in 96% yield without loss of the ee. The corresponding γ-aminoacid **21** was obtained in 78% yield by acidic deprotection.^18^ Then, **21** was reacted with benzoyl chloride to deliver an amino acid derivative **22** in 63% yield with 87% ee.^19^

**Scheme 6:**
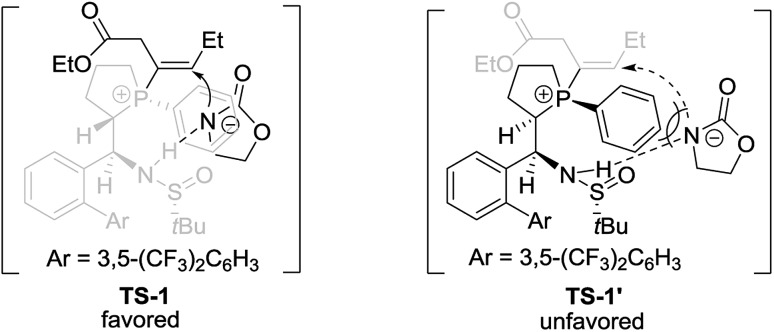
Comparison of two transition states.

Based on the above experimental results and previous relevant studies, a possible transition state (**TS-1**) for (*S*_P_,*R*,*S*,*R*_S_)-**L4** and possible transition state (**TS-1′**) for (*R*_P_,*S*,*S*,*R*_S_)-**L4** to control stereoselectivity are proposed in Scheme 6. For the reaction using (*S*_P_,*R*,*S*,*R*_S_)-**L4** as the catalyst, the nucleophile and the double bond are located on the same side (transition state **TS-1**) *via* the hydrogen-bonding between nucleophiles and the NH moiety, which favors the formation of the *R*-enantiomer of **3**. In contrast, when (*R*_P_,*S*,*S*,*R*_S_)-**L4** was used as the catalyst, another transition state **TS-1′** was proposed, in which there may exist a steric repulsion between the phenyl linked to P and the nucleophile. Additionally, the nucleophile is located on different sides of the double bond and thus hindered the addition reaction to give the product in low yield and ee.

## Conclusions

In summary, we have developed a novel type of bifunctional chiral sulfinamide cyclic phosphine catalyst **Le-Phos**, which can be easily prepared on a gram scale from inexpensive commercially available starting materials in short steps. (*S*_P_,*R*,*S*,*R*_S_)-**Le-Phos** has shown excellent performance in the enantioselective γ-addition reactions of various N-centered nucleophiles to γ-substituted allenoates, acquiring a series of γ-addition adducts in high yields with up to 98% ees and excellent regioselectivity and diastereoselectivity under mild conditions. Its prominent characteristics are general substrate scope, mild reaction conditions, good yields, high enantioselectivities, ease of scale-up to gram scale, and further synthetic transformations of products. Further explorations of **Le-Phos** as the organocatalyst and chiral ligand of transition metals in asymmetric catalysis are currently underway in our group and will be reported in due course.

## Conflicts of interest

There are no conflicts to declare.

## Supplementary Material

Supplementary information

Crystal structure data
