# Supplementary material for: Design, synthesis and application of a new type of bifunctional Le-Phos in highly enantioselective γ-addition reactions of N-centered nucleophiles to allenoates
Source: Chem Sci. 2019 Sep 3;10(45):10510–5. doi: 10.1039/c9sc04073k (PMC6988743; doi:10.1039/c9sc04073k)

Electronic Supplementary Material (ESI) for Chemical Science.
This journal is © The Royal Society of Chemistry 2019

Supporting Information

D Design, Synthesis and Application of a New Type of Bifunctional Le-Phos in Highly Enantioselective *γ*-Addition Reactions of *N*-centered Nucleophiles to Allenoates

Haile Qiu+, Xiaofeng Chen+ and Junliang Zhang*

Shanghai Key Laboratory of Green Chemistry and Chemical Processes, School of Chemistry and Molecular Engineering, East China Normal University, 3663 N. Zhongshan Road, Shanghai 200062, P. R. China;

Department of Chemistry, Fudan University, 2005 Songhu Road, Shanghai 200438, P.R. China

Email: jlzhang@chem.ecnu.edu.cn; junliangzhang@fudan.edu.cn

1. General Information……………………………………………………………………..S2

2. General procedure for the synthesis of catalysts………………………………………………S3

3. Optimization of reaction conditions………………………………………………………. S13

4. General procedure for the cascade reaction of 2-oxazolidones……………………………….S17

5. General procedure for cascade reaction of other *N*-centered nucleophiles……………………S40

6. Experimental procedure, HPLC spectra for the transformations of **3ga**, **6aa**, **6lo**…………….S70

7. The X-ray structure of (R_P, *S*, *S*, R_S)-**L2** borane complex, (S_P, *R*, *S*, R_S)-**L2** borane complex, **6ba** and **13**…………………………………………………………………………… S81

8. References…………………………………………………………………………………..S85

9. NMR spectra………………………………………………………………………………..S86

1. General Information:

Unless otherwise noted, all reactions were carried out under a argon atmosphere; materials obtained from commercial suppliers were used directly without further purification. The [±]D was recorded using PolAAr 3005 High Accuracy Polarimeter. ^{1}H NMR spectra, ^{13}C NMR spectra, and ^{31}P NMR spectra were recorded on a Bruker 400 MHz spectrometer in $CDCl_3$. NMR experiments are reported in δ units, parts per million (ppm), and were referenced to $CDCl_3$ (δ 7.26 or 77.0 ppm) as the internal standard. The data is being reported as (s = singlet, d = doublet, dd = doublet of doublet, t = triplet, m = multiplet or unresolved, br = broad signal, coupling constant(s) in Hz, integration). Trichloromethane ($CHCl_3$), carbon tetrachlorid， dichloromethane, dichloroethane and acetonitrile were freshly distilled from CaH_2; tetrahydrofuran (THF), toluene and ether were dried with sodium benzophenone and distilled before use; Reactions were monitored by thin layer chromatography (TLC) using silicycle pre-coated silica gel plates. Flash column chromatography was performed on silica gel 60 (particle size 300-400 mesh ASTM, purchased from Yantai, China) and eluted with petroleum ether/ethyl acetate.

2. General procedure for the synthesis of catalysts:

2.0 Synthesis of 1-phenylphospholane borane complex:[1]

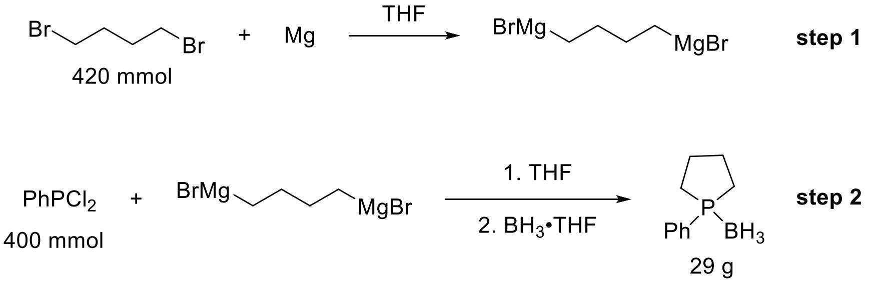

Step 1: Three-mouth flask of 3 liters was used for the reaction. 1,4-Dibromobutane (48 mL, 0.40 mol) diluted with 400 mL THF was slowly added to a stirred suspension of Mg (24 g, 0.99 mol) in THF at 0 °C under an argon atmosphere over a period of 30 min. Then the mixture was allowed to warm to room temperature.

Step 2: After the mixture was stirred for 2 h, the three-mouth flasks was placed at -20 °C and dichlorophenylphosphine (0.40 mol in THF (400 mL)) was slowly added over 60 min. The mixture was allowed to warm to room temperature and stir for 12 h. Then the flask was placed at -20 °C. After BH_3•THF complex (0.44 mol, 1.0 M THF solution,) was added over 1 h, the mixture was stirred for 8 h at room temperature. Then the flask was placed at -20 °C, followed by hydrolysis with 100 mL of saturated NH_4Cl . The organic layer was separated, the aqueous phase was extracted three times with EtOAc (4×100 mL). The combined organic phases were washed with brine, dried over anhydrous Na_2SO_4 and the solvents were removed in vacuo. The residue was purified by silica gel chromatography (PE/EtOAc = 20:1–10:1). Distillation under reduced pressure (b.p. 190 °C/20 mm Hg) gave 1-phenylphospholane borane complex (29 g, 40%).

2.1 Synthesis of catalysts:

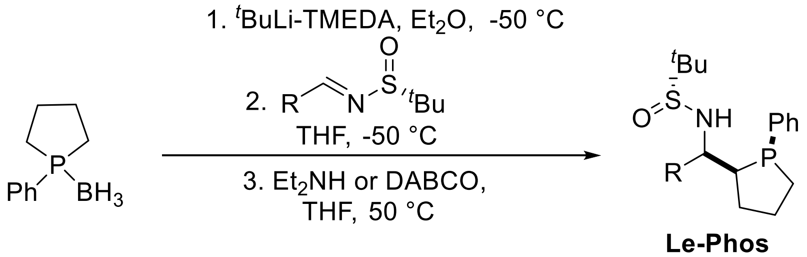

Le-Phos

6.0 mmol ^{t}BuLi (1.3 M in hexane) was slowly added to a Schlenk tube flask that containing anhydrous TMEDA (6.0 mmol) at -50 °C in Et_2O (4.0 mL), the mixture was stirred for 1 hour to a bright yellow precipitate. Then 1-phenylphospholane borane complex[1] (3.0 mmol) dissolved by ether (4.0 mL), was added slowly to the solution of ^{t}BuLi and TMEDA. The mixture was stirred for 4 hours, a wine precipitate was generated.

The solution of corresponding chiral sulfinyl imines (4.50 mmol chiral sulfinyl imines in 5.0 mL anhydrous THF) was added to the prepared mixture at -50 °C. The mixture was stirred until the disappearance of 1-phenylphospholane borane complex as indicated by TLC, lasting approximately 6 hours, followed by hydrolysis with 10 mL of water . The organic layer was separated, the aqueous phase was extracted three times with EtOAc (3×10 mL). The combined organic phases were dried over anhydrous Na_2SO_4 and the solvents were removed in vacuo. The residue was purified by silica gel chromatography to afford the desired (R_P, *S*, *S*, R_S)-Le-Phos borane complex and (S_P, *R*, *S*, R_S)-Le-Phos borane complex, using petroleum ether/EtOAc as the eluent.

The Le-Phos borane complex was added to a Schlenk tube. Then Et_2NH (2.0 mL) was added to the Schlenk tube. The solution was stirred for 4 h at 50 °C, and concentrated.[2] Then the residue was purified by column chromatography (SiO_2, petroleum ether/EtOAc as the eluent) to afford the desired Le-Phos. Another way we added DABCO (4.0 mmol in 2.0 mL THF) to the Schlenk tube.[3] The solution was stirred for 4 h at 50 °C. 1N aq. HCl (5.0 mL) was added to the solution, The organic layer was separated, the aqueous phase was extracted three times with EtOAc (3×10 mL). The combined organic phases were dried over anhydrous Na_2SO_4 and the solvent were removed in vacuo. Then the residue was purified by silica gel chromatography using petroleum ether/EtOAc as the eluent to afford the desired Le-Phos.

2.2 Synthesis and general data of catalysts L1:

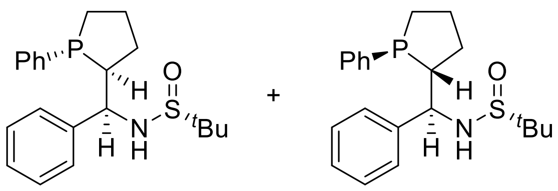

(R_P, S, S, R_S)-**L1** (S_P, R, S, R_S)-**L1**

The general procedure was followed by using 1-phenylphospholane borane complex (534 mg, 3.0 mmol) dissolved by ether (4.0 mL) and the corresponding chiral sulfinyl imines (942 mg, 4.50 mmol chiral sulfinyl imines in 5.0 mL anhydrous THF). After purification by column chromatography (PE/EtOAc = 2:1), (R_P, S, S, R_S)-**L1** borane complex (523 mg, 45%) and (S_P, R, S, R_S)-**L1** borane complex (407 mg, 35%) were obtained as white solids (*d.r.* = 1.29 : 1).

2.2.1 Synthesis and general data of catalysts (R_P, S, S, R_S)-**L1**

The general procedure was followed by using (R_P, S, S, R_S)-**L1** borane complex (523 mg, 1.35 mmol) and Et_2NH (2.0 mL). The resulting solution was stirred at 50 ℃ for 5 hours and then concentrated under vacuum. The residue was purified by silica gel flash chromatography (20% EtOAc in PE) to afford (R_P, S, S, R_S)-**L1** as a white solid (454 mg, 90%). $[\alpha]^{22}_D$ = -51.2 (*c* 0.25, acetone); ^{1}H NMR (400 MHz, $CDCl_3$) δ 7.44 – 7.38 (m, 2H), 7.38 – 7.31 (m, 2H), 7.30 – 7.26 (m, 1H), 7.25 – 7.16 (m, 5H), 4.48 (dt, *J* = 10.4, 7.6 Hz, 1H), 4.07 (dd, *J* = 8.0, 3.2 Hz, 1H), 2.89 – 2.74 (m, 1H), 2.11 – 2.03 (m, 1H), 2.03 – 1.88 (m, 2H), 1.88 – 1.75 (m, 1H), 1.69 – 1.58 (m, 1H), 1.55 – 1.41 (m, 1H), 1.23 (s, 9H); ^{31}P NMR (162 MHz, $CDCl_3$) δ -13.83; ^{13}C NMR (100 MHz, $CDCl_3$) δ 142.75 (d, *J* = 5.4 Hz), 140.93, 140.72, 130.70 (d, *J* = 15.9 Hz), 128.60, 128.31 (d, *J* = 5.5 Hz), 127.62 (d, *J* = 4.6 Hz), 127.15 (d, *J* = 1.9 Hz), 63.86, 63.65, 56.59, 54.01 (d, *J* = 14.1 Hz), 33.39, 28.20 (d, *J* = 3.6 Hz), 26.89 (d, *J* = 10.5 Hz), 22.72; HRMS (ESI) calcd. For $C_{21}H_{28}NNaOPS$ $[M+Na]^+$: 396.1521, found: 396.1524.

2.2.2 Synthesis and general data of catalysts (S_P, R, S, R_S)-**L1**

The general procedure was followed by using (S_P, R, S, R_S)-**L1** borane complex (407 mg, 1.05 mmol) and Et_2NH (2.0 mL). The resulting solution was stirred at 50 ℃ for 5 hours and then concentrated under vacuum. The residue was purified by silica gel

flash chromatography (25% EtOAc in PE) to afford (S_P, *R*, *S*, R_S)-**L1** as a white solid (357 mg, 91%). $[\alpha]^{22}_D$ = 82.4 (*c* 0.25, acetone); ^{1}H NMR (400 MHz, $CDCl_3$) δ 7.39 – 7.36 (m, 2H), 7.33 – 7.25 (m, 3H), 7.25 – 7.13 (m, 5H), 4.55 (td, *J* = 8.8, 6.4 Hz, 1H), 3.73 (d, *J* = 6.4 Hz, 1H), 2.96 – 2.83 (m, 1H), 2.30 – 2.13 (m, 1H), 2.02 – 1.88 (m, 2H), 1.78 – 1.66 (m, 2H), 1.55 – 1.41 (m, 1H), 1.18 (s, 9H); ^{31}P NMR (162 MHz, $CDCl_3$) δ -12.31; ^{13}C NMR (100 MHz, $CDCl_3$) δ 142.11 (d, *J* = 2.6 Hz), 140.85, 140.63, 131.21 (d, *J* = 17.2 Hz), 128.43, 128.16 (d, *J* = 5.8 Hz), 127.91 (d, *J* = 4.0 Hz), 127.83, 127.74, 63.70, 63.41, 56.34, 52.52 (d, *J* = 14.2 Hz), 32.87, 28.37 (d, *J* = 3.6 Hz), 26.63 (d, *J* = 10.6 Hz), 22.64; HRMS (ESI) calcd. For $C_{21}H_{29}NOPS$ $[M+H]^+$: 374.1702, found: 374.1700.

2.3 Synthesis and general data of catalysts L2:

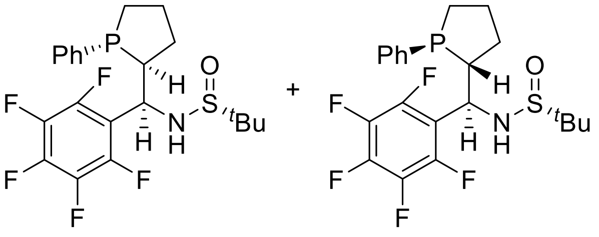

(R_P, *S*, *S*, R_S)-**L2** (S_P, *R*, *S*, R_S)-**L2**

The general procedure was followed by using 1-phenylphospholane borane complex (534 mg, 3.0 mmol) dissolved by ether (4.0 mL) and the corresponding chiral sulfinyl imines (1.35 g, 4.50 mmol chiral sulfinyl imines in 5.0 mL anhydrous THF). After purification by column chromatography (PE/EtOAc = 2:1), (R_P, *S*, *S*, R_S)-**L2** borane complex (415 mg, 29%) and (S_P, *R*, *S*, R_S)-**L2** borane complex (530 mg, 37%) were obtained as white solids (*d.r.* = 1 : 1.28).

2.3.1 Synthesis and general data of catalysts (R_P, *S*, *S*, R_S)-**L2**

The general procedure was followed by using (R_P, *S*, *S*, R_S)-**L2** borane complex (415 mg, 0.87 mmol) and DABCO (195 mg, 1.74 mmol). The resulting solution was stirred at 50 ℃ for 5 hours and then concentrated under vacuum. The residue was purified by silica gel flash chromatography (20% EtOAc in PE) to afford (R_P, *S*, *S*, R_S)-**L2** as colorless oily matter (254 mg, 63%). $[\alpha]^{22}_D$ = -19.2 (*c* 0.25, acetone); ^{1}H NMR (400

MHz, $CDCl_3$) δ 7.52 – 7.44 (m, 2H), 7.40 – 7.28 (m, 3H), 4.70 (td, *J* = 11.2, 7.2 Hz, 1H), 3.88 (d, *J* = 11.2 Hz, 1H), 2.90 – 2.78 (m, 1H), 2.10 – 1.98 (m, 3H), 1.83 – 1.72 (m, 2H), 1.35 – 1.28 (m, 1H), 1.19 (s, 9H); ^{31}P NMR (162 MHz, $CDCl_3$) δ -4.58 (t, *J* = 4.9 Hz); ^{13}C NMR (100 MHz, $CDCl_3$) δ 140.75, 140.52, 131.28 (d, *J* = 17.2 Hz), 128.61 (d, *J* = 6.0 Hz), 128.35, 56.78, 56.50, 51.45 (d, *J* = 13.9 Hz), 32.94 (d, *J* = 1.7 Hz), 29.66, 28.24 (d, *J* = 3.3 Hz), 27.27 (d, *J* = 10.7 Hz), 22.35; HRMS (ESI) calcd. For $C_{21}H_{24}F_5NOPS$ $[M+H]^+$: 464.1231, found: 464.1228.

2.3.2 Synthesis and general data of catalysts (S_P, *R*, *S*, R_S)-**L2**

The general procedure was followed by using (S_P, *R*, *S*, R_S)-**L2** borane complex (530 mg, 1.11 mmol) and DABCO (249 mg, 2.22 mmol). The resulting solution was stirred at 50 ℃ for 5 hours and then concentrated under vacuum. The residue was purified by silica gel flash chromatography (25% EtOAc in PE) to afford (S_P, *R*, *S*, R_S)-**L2** as a white solid (334 mg, 65%). $[\alpha]^{22}_D$ = 67.6 (*c* 0.25, acetone); ^{1}H NMR (400 MHz, $CDCl_3$) δ 7.26 – 7.21 (m, 3H), 7.15 – 7.10 (m, 2H), 4.61 (td, *J* = 11.2, 4.0 Hz, 1H), 3.79 (d, *J* = 11.2 Hz, 1H), 2.86 (tt, *J* = 11.6, 6.8 Hz, 1H), 2.38 – 2.25 (m, 1H), 2.21 – 2.10 (m, 1H), 2.09 – 2.01 (m, 2H), 1.98 – 1.89 (m, 1H), 1.70 – 1.60 (m, 1H), 1.21 (s, 9H); ^{31}P NMR (162 MHz, $CDCl_3$) δ -7.74 (t, *J* = 16.8 Hz); ^{31}P NMR (162 MHz, $CDCl_3$) δ -7.88 (t, *J* = 16.8 Hz); ^{13}C NMR (100 MHz, $CDCl_3$) δ 139.52, 139.31, 131.09 (d, *J* = 18.0 Hz), 128.55, 128.46 (d, *J* = 6.3 Hz), 56.79, 56.66, 56.37, 51.36 (d, *J* = 11.9 Hz), 33.63 (d, *J* = 2.3 Hz), 28.29 (d, *J* = 3.2 Hz), 26.34 (d, *J* = 10.5 Hz), 22.43; HRMS (ESI) calcd. For $C_{21}H_{24}F_5NOPS$ $[M+H]^+$: 464.1231, found: 464.1237.

2.4 Synthesis and general data of catalysts L3:

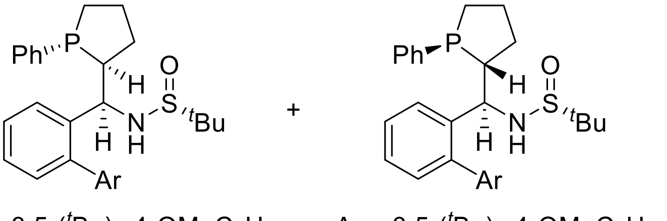

Ar = 3,5-(^{t}Bu)$_2$-4-OMeC_6H_2 Ar = 3,5-(^{t}Bu)$_2$-4-OMeC_6H_2

(R_P, *S*, *S*, R_S)-**L3** (S_P, *R*, *S*, R_S)-**L3**

The general procedure was followed by using 1-phenylphospholane borane complex (534 mg, 3.0 mmol) dissolved by ether (4.0 mL) and the corresponding chiral sulfinyl imines (1.92 g, 4.50 mmol chiral sulfinyl imines in 5.0 mL anhydrous THF). After purification by column chromatography (PE/EtOAc = 2:1), (R_P, *S*, *S*, R_S)-**L3** borane complex (690 mg, 38%) and (S_P, *R*, *S*, R_S)-**L3** borane complex (545 mg, 30%) were obtained as white solids (*d.r.* = 1.27 : 1).

2.4.1 Synthesis and general data of catalysts (R_P, *S*, *S*, R_S)-**L3**

The general procedure was followed by using (R_P, *S*, *S*, R_S)-**L3** borane complex (690 mg, 1.14 mmol) and Et_2NH (2.0 mL). The resulting solution was stirred at 50 ℃ for 5 hours and then concentrated under vacuum. The residue was purified by silica gel flash chromatography (20% EtOAc in PE) to afford (R_P, *S*, *S*, R_S)-**L3** as a white solid (540 mg, 80%). $[\alpha]^{22}_D$ = -20.0 (*c* 0.25, acetone); ^{1}H NMR (500 MHz, $CDCl_3$) δ 7.66 (d, *J* = 7.0 Hz, 1H), 7.45 – 7.40 (m, 1H), 7.34 – 7.30 (m, 1H), 7.26 – 7.10 (m, 7H), 7.07 – 7.02 (m, 2H), 4.66 (s, 1H), 4.20 (s, 1H), 3.74 (s, 3H), 2.54 (dd, *J* = 9.0, 6.0 Hz, 1H), 2.08 – 2.02 (m, 1H), 1.89 – 1.84 (m, 1H), 1.77 – 1.66 (m, 1H), 1.58 – 1.51 (m, 2H), 1.43 (s, 18H), 1.23 (s, 9H); ^{31}P NMR (202 MHz, $CDCl_3$) δ -17.50; ^{13}C NMR (125 MHz, $CDCl_3$) δ 158.43, 143.24, 141.88, 140.90, 140.74, 135.28, 130.55, 130.43, 128.30 (d, *J* = 5.4 Hz), 127.91, 127.68, 127.54, 127.02, 64.28, 56.70, 35.84, 33.55, 32.18, 28.01 (d, *J* = 3.4 Hz), 26.52 (d, *J* = 10.1 Hz), 22.75; HRMS (ESI) calcd. For $C_{36}H_{51}NO_2PS$ $[M+H]^+$: 592.3373, found: 592.3375.

2.4.2 Synthesis and general data of catalysts (S_P, *R*, *S*, R_S)-**L3**

The general procedure was followed by using (S_P, *R*, *S*, R_S)-**L3** borane complex (545 mg, 0.90 mmol) and Et_2NH (2.0 mL). The resulting solution was stirred at 50 ℃ for 5 hours and then concentrated under vacuum. The residue was purified by silica gel flash chromatography (25% EtOAc in PE) to afford (S_P, *R*, *S*, R_S)-**L3** as a white solid (426 mg, 80%). $[\alpha]^{22}_D$ = 23.6 (*c* 0.25, acetone); ^{1}H NMR (500 MHz, $CDCl_3$) δ 7.53 – 7.49 (m, 1H), 7.36 (s, 2H), 7.35 – 7.16 (m, 7H), 7.16 – 7.11 (m, 2H), 4.81 – 4.72 (m,

1H), 3.77 (s, 3H), 3.73 (d, J = 8.0 Hz, 1H), 2.83 – 2.75 (m, 1H), 2.03 – 1.94 (m, 2H), 1.84 (s, 1H), 1.76 – 1.61 (m, 2H), 1.49 (s, 18H), 1.17 (s, 9H); ^{31}P NMR (202 MHz, $CDCl_3$) δ -11.71; ^{13}C NMR (125 MHz, $CDCl_3$) δ 158.39, 142.99, 142.30, 141.15, 140.96, 140.15 (d, J = 2.6 Hz), 135.44, 130.94, 130.80, 130.38, 128.63, 128.22 (d, J = 5.5 Hz), 127.55, 127.39, 127.11, 64.22, 59.76, 59.53, 56.38, 53.24, 53.12, 35.94, 32.54, 32.30, 29.73, 28.16 (d, J = 3.6 Hz), 26.15, 26.06, 22.57; HRMS (ESI) calcd. For $C_{36}H_{51}NO_2PS$ $[M+H]^+$: 592.3373, found: 592.3373.

2.5 Synthesis and general data of catalysts L4:

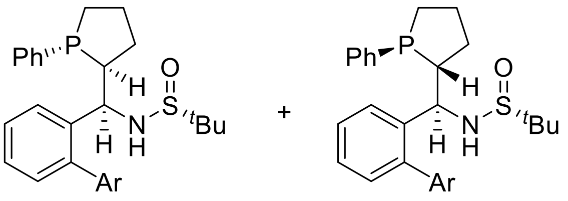

Ar = 3,5-$(CF_3)_2C_6H_3$ (*R*P, *S*, *S*, *R*S)-**L4**

Ar = 3,5-$(CF_3)_2C_6H_3$ (*S*P, *R*, *S*, *R*S)-**L4**

The general procedure was followed by using 1-phenylphospholane borane complex (534 mg, 3.0 mmol) dissolved by ether (4.0 mL) and the corresponding chiral sulfinyl imines (1.90 g, 4.50 mmol chiral sulfinyl imines in 5.0 mL anhydrous THF). After purification by column chromatography (PE/EtOAc = 2:1), (R_P, *S*, *S*, R_S)-**L4** borane complex (629 mg, 35%) and (S_P, *R*, *S*, R_S)-**L4** borane complex (539 mg, 30%) were obtained as white solids (*d.r.* = 1.17 : 1).

To prove the practical usefulness of the catalysts, the reaction was carried out on a gram scale. The general procedure was followed by using 1-phenylphospholane borane complex (1.78 g, 10.0 mmol) dissolved by ether (20.0 mL) and the corresponding chiral sulfinyl imines (6.33 g, 4.50 mmol chiral sulfinyl imines in 10.0 mL anhydrous THF). After purification by column chromatography (PE/EtOAc = 2:1), (R_P, *S*, *S*, R_S)-**L4** borane complex (2.15 g, 36%) and (S_P, *R*, *S*, R_S)-**L4** borane complex (1.79 g, 30%) were obtained as white solids (*d.r.* = 1.2 : 1).

2.5.1 Synthesis and general data of catalysts (R_P, *S*, *S*, R_S)-**L4**

The general procedure was followed by using (R_P, S, S, R_S)-**L4** borane complex (629 mg, 1.05 mmol) and Et_2NH (2.0 mL). The resulting solution was stirred at 50 ℃ for 5 hours and then concentrated under vacuum. The residue was purified by silica gel flash chromatography (20% EtOAc in PE) to afford (R_P, S, S, R_S)-**L4** as a white solid (492 mg, 80%). $[\alpha]^{22}_D$ = -45.6 (*c* 0.25, acetone); ^{1}H NMR (400 MHz, $CDCl_3$) δ 7.82 (s, 1H), 7.76 (s, 2H), 7.67 (d, J = 7.6 Hz, 1H), 7.41 (t, J = 7.2 Hz, 1H), 7.27 (t, J = 7.2 Hz, 1H), 7.21 – 7.13 (m, 6H), 7.08 (d, J = 7.6 Hz, 1H), 4.22 – 4.03 (m, 2H), 2.57 (s, 1H), 1.97 – 1.86 (m, 1H), 1.79 – 1.66 (m, 2H), 1.63 – 1.46 (m, 2H), 1.10 (s, 9H); ^{31}P NMR (202 MHz, $CDCl_3$) δ -13.78; ^{19}F NMR (282 MHz, $CDCl_3$) δ -62.71; ^{13}C NMR (100 MHz, $CDCl_3$) δ 143.32, 140.50, 140.30, 140.15, 140.09, 138.29, 131.73, 131.39, 131.06, 130.90, 129.93 (d, J = 8.7 Hz), 129.41, 128.49 (d, J = 5.8 Hz), 128.02, 127.72, 124.65, 121.94, 121.36 – 120.97 (m), 56.70, 33.35, 28.01 (d, J = 3.4 Hz), 26.88, 26.77, 22.56; HRMS (ESI) calcd. For $C_{29}H_{31}F_6NOPS$ $[M+H]^+$: 586.1763, found: 586.1771.

2.5.2 Synthesis and general data of catalysts (S_P, R, S, R_S)-**L4**

The general procedure was followed by using (S_P, R, S, R_S)-**L4** borane complex (539 mg, 0.90 mmol) and Et_2NH (2.0 mL). The resulting solution was stirred at 50 ℃ for 5 hours and then concentrated under vacuum. The residue was purified by silica gel flash chromatography (25% EtOAc in PE) to afford (S_P, R, S, R_S)-**L4** as a white solid (411 mg, 78%). $[\alpha]^{22}_D$ = 5.1 (*c* 0.25, acetone); ^{1}H NMR (400 MHz, $CDCl_3$) δ 8.00 (s, 2H), 7.81 (s, 1H), 7.35 – 7.29 (m, 1H), 7.29 – 7.20 (m, 2H), 7.13 – 7.05 (m, 4H), 6.97 – 6.91 (m, 2H), 4.22 – 4.13 (m, 1H), 3.53 (d, J = 8.4 Hz, 1H), 2.68 – 2.57 (m, 1H), 2.08 – 1.98 (m, 2H), 1.93 – 1.81 (m, 2H), 1.74 – 1.52 (m, 1H), 1.06 (s, 9H); ^{31}P NMR (202 MHz, $CDCl_3$) δ -11.08; ^{19}F NMR (282 MHz, $CDCl_3$) δ -62.62; ^{13}C NMR (100 MHz, $CDCl_3$) δ 143.42, 140.63 (d, J = 2.1 Hz), 140.29, 140.07, 138.46, 131.70, 131.37, 131.20, 131.02, 130.84, 130.70, 129.98, 129.17, 128.26 (d, J = 6.0 Hz), 127.97, 127.66, 127.53, 127.35, 124.82, 122.11, 121.10 – 120.73 (m), 119.40, 61.17, 60.80, 56.48, 54.36, 54.23, 33.32, 28.50 (d, J = 3.4 Hz), 25.82, 25.71, 22.44; HRMS (ESI) calcd. For $C_{29}H_{31}F_6NOPS$ $[M+H]^+$: 586.1763, found: 586.1767.

2.6 Synthesis and general data of catalysts L5:

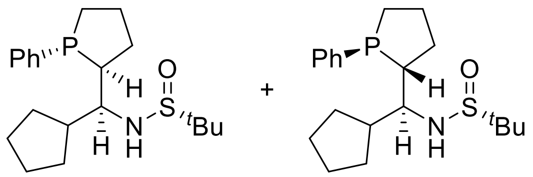

(R_P, S, S, R_S)-**L5** (S_P, R, S, R_S)-**L5**

The general procedure was followed by using 1-phenylphospholane borane complex (1.78 g, 10.0 mmol) dissolved by ether (20.0 mL) and the corresponding chiral sulfinyl imines (3.00 g, 15.0 mmol chiral sulfinyl imines in 5.0 mL anhydrous THF). After purification by column chromatography (PE/EtOAc = 2:1), (R_P, S, S, R_S)-**L5** borane complex (1.15 g, 30%) and (S_P, R, S, R_S)-**L5** borane complex (1.30 g, 34%) were obtained as white solids (*d.r.* = 1 : 1.13).

2.6.1 Synthesis and general data of catalysts (R_P, S, S, R_S)-L5

The general procedure was followed by using (R_P, S, S, R_S)-**L5** borane complex (380 mg, 1.0 mmol) and Et_2NH (2.0 mL). The resulting solution was stirred at 50 ℃ for 5 hours and then concentrated under vacuum. The residue was purified by silica gel flash chromatography (25% EtOAc in PE) to afford (R_P, S, S, R_S)-**L5** as a white solid (307 mg, 84%). $[\alpha]^{22}_D$ = -44.2 (*c* 1.0, acetone); ^{1}H NMR (400 MHz, $CDCl_3$) δ 7.48 – 7.41 (m, 2H), 7.35 – 7.24 (m, 3H), 3.67 (dd, *J* = 8.0, 2.0 Hz, 1H), 3.28 – 3.17 (m, 1H), 2.65 (ddd, *J* = 13.0, 8.4, 2.1 Hz, 1H), 2.19 – 2.04 (m, 3H), 2.04 – 1.99 (m, 1H), 1.97 – 1.86 (m, 1H), 1.77 – 1.33 (m, 10H), 1.26 (s, 9H); ^{31}P NMR (162 MHz, $CDCl_3$) δ -20.05 (s); ^{13}C NMR (100 MHz, $CDCl_3$) δ 141.28, 141.07, 131.55, 131.38, 128.43 (d, *J* = 5.9 Hz), 127.89, 64.24, 64.09, 56.64, 51.00 (d, *J* = 12.2 Hz), 47.98 (d, *J* = 7.9 Hz), 33.61, 31.05, 30.66, 28.26 (d, *J* = 4.0 Hz), 27.62, 27.52, 25.38, 25.26, 23.24. HRMS (ESI) calcd. For $C_{20}H_{33}NOPS$ $[M+H]^+$: 366.2010, found: 366.2002.

2.6.2 Synthesis and general data of catalysts (S_P, R, S, R_S)-L5

The general procedure was followed by using (S_P, R, S, R_S)-**L5** borane complex (1.30 g, 3.40 mmol) and Et_2NH (4.0 mL). The resulting solution was stirred at 50 ℃ for 5 hours and then concentrated under vacuum. The residue was purified by silica gel

flash chromatography (25% EtOAc in PE) to afford (S_P, *R*, *S*, R_S)-**L5** as a white solid (1.10 g, 88%). $[\alpha]^{22}_D$ = -2.9 (*c* 1.0, acetone); ^{1}H NMR (500 MHz, $CDCl_3$) δ 7.41 (dd, *J* = 10.8, 4.0 Hz, 2H), 7.34 – 7.29 (m, 2H), 7.26 – 7.21 (m, 1H), 3.59 – 3.50 (m, 1H), 3.27 (d, *J* = 6.1 Hz, 1H), 2.54 (td, *J* = 12.7, 6.3 Hz, 1H), 2.25 – 2.15 (m, 1H), 2.13 – 2.00 (m, 3H), 1.84 – 1.75 (m, 2H), 1.74 – 1.66 (m, 3H), 1.63 – 1.57 (m, 1H), 1.57 – 1.46 (m, 3H), 1.46 – 1.38 (m, 1H), 1.34 (ddd, *J* = 17.1, 8.9, 3.7 Hz, 1H), 1.25 (s, 9H); ^{31}P NMR (202 MHz, $CDCl_3$) δ -9.95; ^{13}C NMR (126 MHz, $CDCl_3$) δ 141.92 (d, *J* = 22.8 Hz), 130.76 (d, *J* = 16.5 Hz), 128.37 (d, *J* = 5.4 Hz), 127.59, 63.62, 63.42, 56.53, 51.49 (d, *J* = 13.3 Hz), 45.06 (d, *J* = 10.0 Hz), 30.53 (d, *J* = 10.5 Hz), 29.35, 28.44 (d, *J* = 3.8 Hz), 26.48 (d, *J* = 11.4 Hz), 25.43 (s), 25.29, 23.06 (d, *J* = 0.9 Hz). HRMS (ESI) calcd. For $C_{20}H_{33}NOPS$ $[M+H]^+$: 366.2015, found: 366.2006.

Optimization of reaction conditions

3.1 Table S-1: Reaction conditions screening of pyrrolidine-2,5-diones with Allenyl esters.[a]

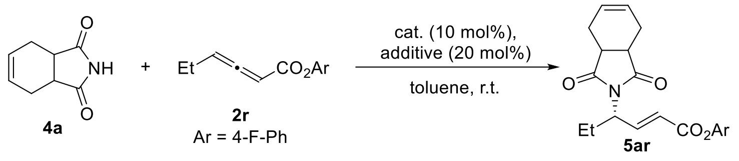

Entry	Catalyst	Additive	T (h)	*E/Z*[b]	*ee* (%)[c]
1	**P9**	-	8	4:1	60
2	(R_P, *S*, *S*, R_S)-**L1**	-	4	2:1	25
3	(R_P, *S*, *S*, R_S)-**L2**	-	4	2:1	14
4	(S_P, *R*, *S*, R_S)-**L1**	-	4	6:1	48
5	(S_P, *R*, *S*, R_S)-**L2**	-	4	6:1	88
6	(S_P, *R*, *S*, R_S)-**L2**	PhOH	6	8:1	88
7	(S_P, *R*, *S*, R_S)-**L2**	AcOH	12	4:1	88
8	(S_P, *R*, *S*, R_S)-**L2**	2,6-dibromophenol	6	9:1	89
9[d]	(S_P, *R*, *S*, R_S)-**L2**	2,6-dibromophenol	12	10:1	84
10[e]	(S_P, *R*, *S*, R_S)-**L2**	2,6-dibromophenol	12	10:1	89
11[f]	(S_P, *R*, *S*, R_S)-**L2**	2,6-dibromophenol	12	-	-
12[g, j]	(S_P, *R*, *S*, R_S)-**L2**	2,6-dibromophenol	12	10:1	90
13[h]	(S_P, *R*, *S*, R_S)-**L2**	2,6-dibromophenol	12	9:1	92
14[i]	(S_P, *R*, *S*, R_S)-**L2**	2,6-dibromophenol	12	6:1	92
15[h]	(*S*, *S*)-DIOP	2,6-dibromophenol	18	4:1	11
16	(*R*, *R*)-Et-BPE	2,6-dibromophenol	18	3:1	31
17[h]	(*S*)-SITCP	2,6-dibromophenol	12	3:5	22

[a]Reaction conditions: **4a** (0.10 mmol), **2r** (0.20 mmol), and the catalyst (0.01 mmol) in toluene (1.5 mL) at room temperature. [b]Determined by ^{1}H NMR analysis of the crude mixture. [c]Determined by HPLC analysis on a chiral stationary phase. [d]In dichloromethane. [e]In Et_2O. [f]In 1,4-dioxane. [g]In xylene. [h]At 0 °C. [i]At -10 °C. [j]60% conversion.

3.2 Table S-2: Allenyl esters optimization.[4]

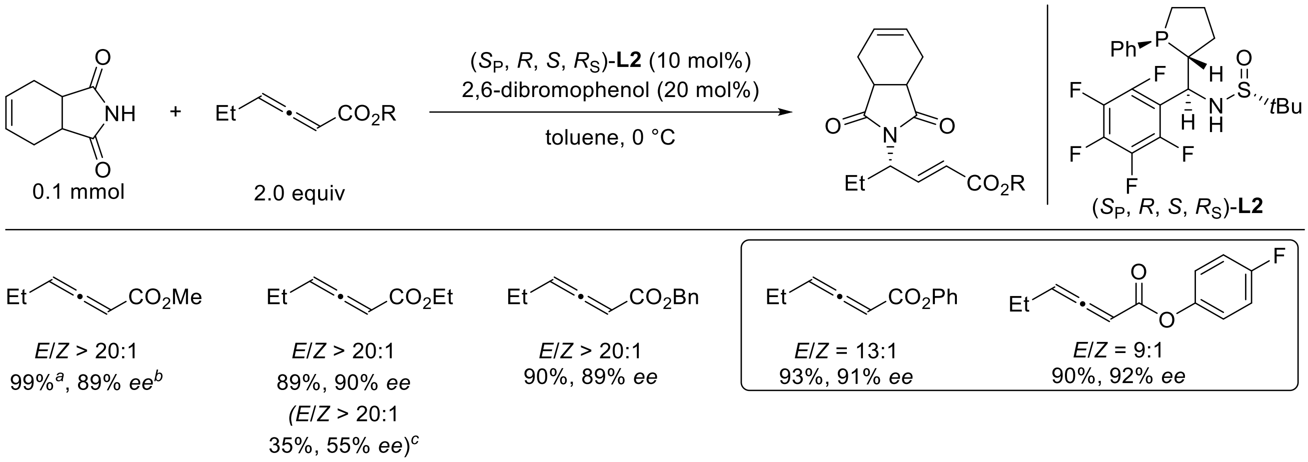

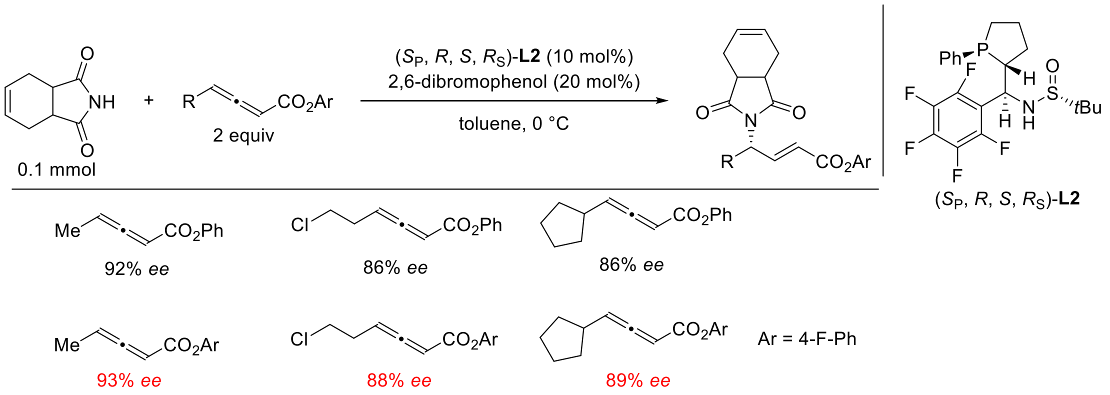

[a]isolated yield. [b]Determined by HPLC analysis on a chiral stationary phase. [c]Performed with catalyst **P8** (0.01 mmol) in toluene (1.5 mL) at room temperature.

3.3 Table S-3: Reaction conditions screening of $TsNH_2$ with Allenyl esters. [a]

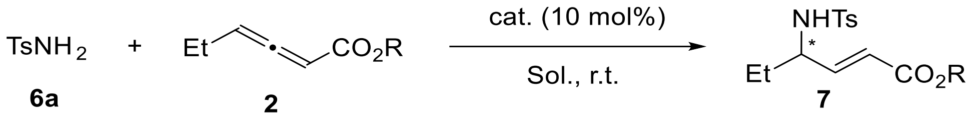

Entry	Catalyst	Solvent	R	*E/Z*[b]	Yield (%)[b]	*ee* (%)[c]
1	(S_P, *R*, *S*, R_S)-**L1**	DCE	Et	> 20:1	41	0
2	(S_P, *R*, *S*, R_S)-**L3**	DCE	Et	> 20:1	22	8
3	(S_P, *R*, *S*, R_S)-**L4**	DCE	Et	> 20:1	28	50
4	(S_P, *R*, *S*, R_S)-**L5**	DCE	Et	> 20:1	59	60
5	(S_P, *R*, *S*, R_S)-**L5**	Toluene	Et	> 20:1	36	65
6	(S_P, *R*, *S*, R_S)-**L5**	Et_2O	Et	> 20:1	34	70

7	(S_P, *R*, *S*, R_S)-**L5**	$PhCF_3$	Et	> 20:1	40	65
8	(S_P, *R*, *S*, R_S)-**L5**	DCM	Et	> 20:1	55	54
9	(S_P, *R*, *S*, R_S)-**L5**	MeCN	Et	> 20:1	13	66
10	(S_P, *R*, *S*, R_S)-**L5**	Et_2O	Bn	> 20:1	28	83
11	(S_P, *R*, *S*, R_S)-**L5**	Et_2O	Me	> 20:1	34	87
12	(S_P, *R*, *S*, R_S)-**L5**	Et_2O	^{t}Bu	> 20:1	28	78
13[d]	(S_P, *R*, *S*, R_S)-**L5**	Et_2O	Me	> 20:1	45	87
14[e]	(S_P, *R*, *S*, R_S)-**L5**	Et_2O	Me	> 20:1	58	87
15	**P8**	Et_2O	Me	> 20:1	30	86

[a]Reaction conditions: **6a** (0.10 mmol), **2** (0.20 mmol), and the catalyst (0.01 mmol) in solvent(1.5 mL) at room temperature. [b]NMR yield with the use of CH_2Br_2 as internal standard. [c]Determined by HPLC analysis on a chiral stationary phase. [d]Performed with **L5** (0.15 equiv.). [e]Performed with **L5** (0.20 equiv.). DCM = Dichloromethane, DCE = 1,2-Dichloroethane.

3.4 Table S-4: Reaction conditions screening of $(BocNH)_2$ with Allenyl esters. [a]

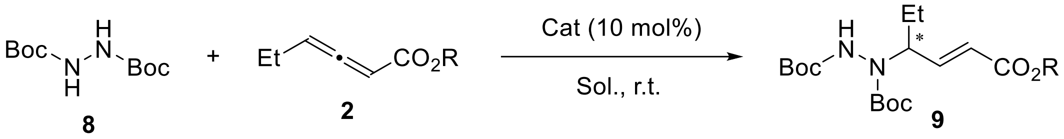

Entry	Catalyst	Solvent	R	*E*/*Z*[b]	Yield (%)[b]	*ee* (%)[c]
1	(S_P, *R*, *S*, R_S)-**L1**	DCE	Et	> 20:1	18	65
2	(S_P, *R*, *S*, R_S)-**L3**	DCE	Et	> 20:1	51	70
3	(S_P, *R*, *S*, R_S)-**L4**	DCE	Et	> 20:1	60	77
4	(S_P, *R*, *S*, R_S)-**L5**	DCE	Et	> 20:1	trace	-
5	(S_P, *R*, *S*, R_S)-**L4**	DCE	Bn	> 20:1	80	80
6	(S_P, *R*, *S*, R_S)-**L4**	DCE	Me	> 20:1	76	75
7	(S_P, *R*, *S*, R_S)-**L4**	DCE	Bu	> 20:1	62	63
8	(S_P, *R*, *S*, R_S)-**L4**	Toluene	Bn	> 20:1	36	80
9	(S_P, *R*, *S*, R_S)-**L4**	Et_2O	Bn	> 20:1	trace	-
10	(S_P, *R*, *S*, R_S)-**L4**	$CHCl_3$	Bn	> 20:1	58	82
11	(S_P, *R*, *S*, R_S)-**L4**	DCM	Bn	> 20:1	88	83

12	(S_P, *R*, *S*, R_S)-**L4**	MeCN	Bn	> 20:1	Trace	-
13	**P8**	DCM	Bn	> 20:1	38	21

[a]Reaction conditions: **8** (0.10 mmol), **2** (0.20 mmol), and the catalyst (0.01 mmol) in solvent(1.5 mL) at room temperature. [b]NMR yield with the use of CH_2Br_2 as internal standard. [c]Determined by HPLC analysis on a chiral stationary phase. DCM = Dichloromethane, DCE = 1,2-Dichloroethane.

3.5 Table S-5: Reaction conditions screening of pyrazole with Allenyl esters. [a]

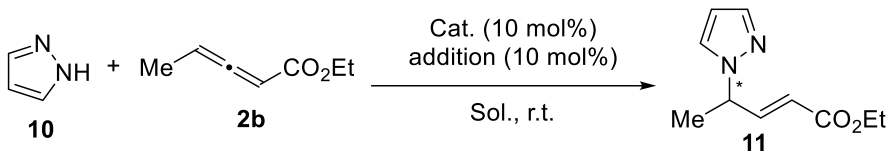

Entry	Catalyst	Solvent	Additive	*E/Z*[b]	Yield (%)[b]	*ee* (%)[c]
1	(S_P, *R*, *S*, R_S)-**L1**	Toluene	-	> 20:1	87	58
2	(S_P, *R*, *S*, R_S)-**L2**	Toluene	-	> 20:1	84	64
3	(R_P, *S*, *S*, R_S)-**L4**	Toluene	-	> 20:1	72	-22
4	(S_P, *R*, *S*, R_S)-**L4**	Toluene	-	> 20:1	79	84
5	(R_P, *S*, *S*, R_S)-**L5**	Toluene	-	> 20:1	79	-44
6	(S_P, *R*, *S*, R_S)-**L5**	Toluene	-	> 20:1	87	57
7	(S_P, *R*, *S*, R_S)-**L4**	Et_2O	-	> 20:1	69	85
8	(S_P, *R*, *S*, R_S)-**L4**	DCE	-	> 20:1	80	85
9	(S_P, *R*, *S*, R_S)-**L4**	DCM	-	> 20:1	82	83
10	(S_P, *R*, *S*, R_S)-**L4**	THF	-	> 20:1	70	88
11	(S_P, *R*, *S*, R_S)-**L4**	Dioxane	-	> 20:1	83	88
12	(S_P, *R*, *S*, R_S)-**L4**	Dioxane	PhOH	> 20:1	79	85
13	(S_P, *R*, *S*, R_S)-**L4**	Dioxane	(S)-BINOL	> 20:1	85	84
14	(S_P, *R*, *S*, R_S)-**L4**	Dioxane	(R)-BINOL	> 20:1	79	83
15	(S_P, *R*, *S*, R_S)-**L4**	Dioxane	KH_2PO_4	> 20:1	77	85
16	(S_P, *R*, *S*, R_S)-**L4**	Dioxane	CH_3COOH	> 20:1	65(66[d])	95
17[e]	(S_P, *R*, *S*, R_S)-**L4**	Dioxane	CH_3COOH	> 20:1	76[d]	95
18[f]	(S_P, *R*, *S*, R_S)-**L4**	Dioxane	CH_3COOH	> 20:1	85[d]	95

[a]Reaction conditions: **10** (0.10 mmol), **2b** (0.20 mmol), and the catalyst (0.01 mmol) in solvent(1.5 mL) at room temperature. [b]NMR yield with the use of CH_2Br_2 as internal standard. [c]Determined by HPLC analysis on a chiral stationary phase. [d]Yield of isolated product. [e]Performed with **2b** (0.25 mmol). [f]Performed with **2b** (0.30 mmol). DCM = Dichloromethane, DCE = 1,2-Dichloroethane.

3. General procedure for the cascade reaction of 2-oxazolidones:

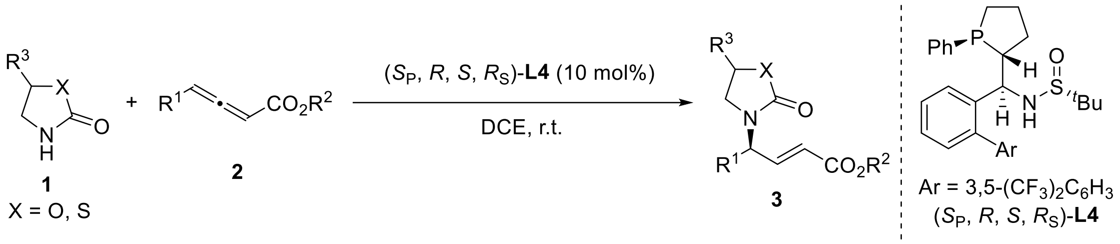

To a flame-dried glass tube with a magnetic stirring bar were added 2-oxazolidone **1a** (8.7 mg, 0.10 mmol) and (S_P, *R*, *S*, R_S)-**L4** (5.9 mg, 0.01 mmol), followed by the addition of dry 1,2-Dichloroethane (1.5 mL).[5] Then the allenoate **2a** (28.0 mg, 0.20 mmol) was slowly added via syringe at room temperature under inert atmosphere. The reaction mixture was stirred for 24 h, and TLC show that the rection was completed. Then 1,2-Dichloroethane was removed under reduced pressure. The residue was purified by column chromatography on silica gel (petroleum ether/ethyl acetate = 2:1) to afford **3aa** (20.4 mg, 90% yield).

4.1 Synthesis of ethyl (*R*, *E*) -4-(2-oxooxazolidin-3-yl)pent-2-enoate (3ab).

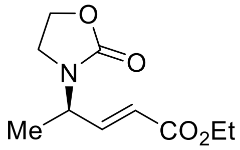

3ab

The general procedure was followed using **1a** (8.7 mg, 0.10 mmol) and **2b** (25.2 mg, 0.20 mmol). After purification by column chromatography (PE/EtOAc = 2:1), **3ab** (19.6 mg, 91%) was obtained as a colorless oil. $[\alpha]^{22}_D$ = -11.6 (*c* 0.10, acetone); ^1H NMR (400 MHz, $CDCl_3$) δ 6.83 (dd, *J* = 15.6, 4.8 Hz, 1H), 5.90 (dd, *J* = 16.0, 0.8 Hz, 1H), 4.71 – 4.65 (m, 1H), 4.34 (t, *J* = 8.4 Hz, 2H), 4.18 (q, *J* = 7.2 Hz, 2H), 3.58 – 3.35 (m, 2H), 1.34 (d, *J* = 7.2 Hz, 3H), 1.27 (t, *J* = 7.2 Hz, 3H); ^{13}C NMR (100 MHz,

CDCl3) δ 165.88, 157.72, 145.54, 122.55, 62.08, 60.71, 49.05, 40.28, 16.49, 14.20; Enantiomeric excess: 98%, determined by HPLC (Chiralpak IF hexane/*i*-PrOH = 70/30; flow rate 0.6 ml/min; 25 ℃; 254 nm), first peak: t_R = 19.9 min, second peak: t_R = 20.9 min. HRMS (ESI) calcd. for $C_{10}H_{15}NNaO_4$ $[M+Na]^+$: 236.0893, found: 236.0890.

<Chromatogram>

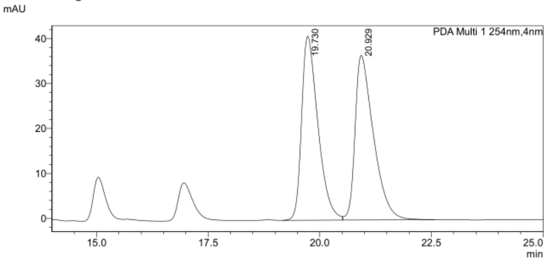

<Peak Table>

PDA Ch1 254nm

Peak#	Ret. Time	Area	Area%	Height	Height%
1	19.730	1040798	49.666	40923	52.775
2	20.929	1054782	50.334	36620	47.225
Total		2095580	100.000	77543	100.000

<Chromatogram>

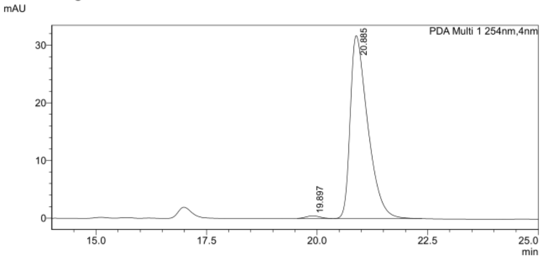

<Peak Table>

PDA Ch1 254nm

Peak#	Ret. Time	Area	Area%	Height	Height%
1	19.897	9986	1.123	458	1.422
2	20.885	879541	98.877	31744	98.578
Total		889527	100.000	32201	100.000

4.2 Synthesis of ethyl (*R, E*)-4-(2-oxooxazolidin-3-yl)hept-2-enoate (3ac).

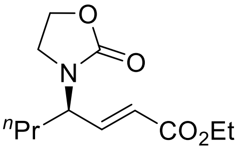

3ac

The general procedure was followed using **1a** (8.7 mg, 0.10 mmol) and **2c** (30.8 mg, 0.20 mmol). After purification by column chromatography (PE/EtOAc = 2:1), **3ac** (21.0 mg, 87%) was obtained as a colorless oil. $[\alpha]^{22}_D$ = -0.2 (*c* 0.25, acetone); ^{1}H NMR (400 MHz, CDCl3) δ 6.79 (dd, *J* = 16.0, 6.0 Hz, 1H), 5.91 (dd, *J* = 16.0, 1.6 Hz, 1H), 4.53 - 4.44 (m, *J* = 14.0, 1H), 4.34 (t, *J* = 8.0 Hz, 2H), 4.18 (q, *J* = 7.2 Hz, 2H), 3.52 – 3.41 (m, 2H), 1.65 – 1.58 (m, 2H), 1.38 – 1.31 (m, 2H), 1.27 (t, *J* = 7.2 Hz, 3H), 0.94 (t, *J* = 7.2 Hz, 3H); ^{13}C NMR (100 MHz, CDCl3) δ 165.92, 158.01, 144.30, 123.01, 62.08, 60.67, 53.39, 40.23, 33.06, 19.18, 14.19, 13.58; Enantiomeric excess: 95%, determined by HPLC (Chiralpak IF hexane/*i*-PrOH = 75/25; flow rate 0.5 ml/min; 25 ℃; 190 nm), first peak: t_R = 21.5 min, second peak: t_R = 23.7 min. HRMS (ESI) calcd. for $C_{12}H_{19}NNaO_4$ $[M+Na]^+$: 264.1206, found: 264.1201.

<Chromatogram>

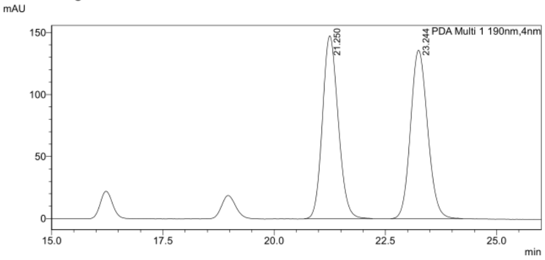

<Peak Table>

PDA Ch1 190nm

Peak#	Ret. Time	Area	Area%	Height	Height%
1	21.250	3632824	50.057	147412	52.057
2	23.244	3624580	49.943	135763	47.943
Total		7257404	100.000	283175	100.000

<Chromatogram>

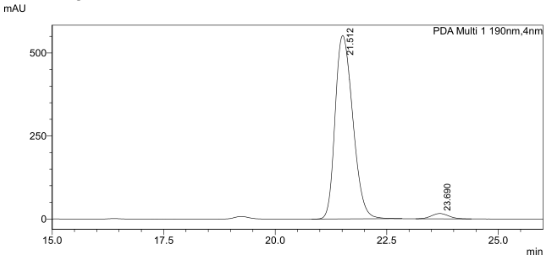

<Peak Table>

PDA Ch1 190nm

Peak#	Ret. Time	Area	Area%	Height	Height%
1	21.512	15217630	97.260	552416	97.183
2	23.690	428778	2.740	16013	2.817
Total		15646407	100.000	568429	100.000

4.3 Synthesis of ethyl (*R, E*)-4-(2-oxooxazolidin-3-yl)tetradec-2-enoate (3ad).

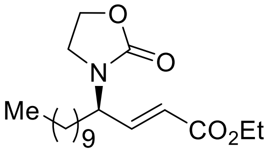

3ad

The general procedure was followed using **1a** (8.7 mg, 0.10 mmol) and **2d** (50.5 mg, 0.20 mmol). After purification by column chromatography (PE/EtOAc = 2:1), **3ad** (31.5 mg, 93%) was obtained as a colorless oil. $[\alpha]^{22}_{D}$ = -19.8 (*c* 0.10, acetone); ^{1}H NMR (400 MHz, $CDCl_3$) δ 6.80 (dd, *J* = 16.0, 6.0 Hz, 1H), 5.91 (d, *J* = 16.0 Hz, 1H), 4.46 (dd, *J* = 14.0, 6.8 Hz, 1H), 4.34 (t, *J* = 8.0 Hz, 2H), 4.18 (q, *J* = 7.2 Hz, 2H), 3.46 (t, *J* = 8.8 Hz, 2H), 1.67 – 1.58 (m, 2H), 1.32 – 1.21 (m, 19H), 0.85 (t, *J* = 6.0 Hz, 3H); ^{13}C NMR (100 MHz, $CDCl_3$) δ 165.95, 158.02, 144.36, 122.97, 62.08, 60.68, 53.69, 40.21, 31.87, 31.02, 29.53 (d, *J* = 3.2 Hz), 29.41, 29.28, 29.15, 25.94, 22.66, 14.15 (d, *J* = 10.1 Hz); Enantiomeric excess: 94%, determined by HPLC (Chiralpak IF hexane/*i*-PrOH = 70/30; flow rate 0.6 ml/min; 25 ℃; 190 nm), first peak: t_R = 13.7 min, second peak: t_R = 14.5 min. HRMS (ESI) calcd. for $C_{19}H_{33}NNaO_4$ $[M+Na]^+$: 362.2302, found: 362.2304.

<Chromatogram>

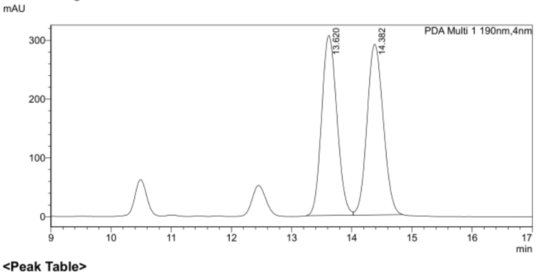

<Peak Table>

PDA Ch1 190nm

Peak#	Ret. Time	Area	Area%	Height	Height%
1	13.620	5418601	49.891	306174	51.339
2	14.382	5442265	50.109	290200	48.661
Total		10860865	100.000	596374	100.000

<Chromatogram>

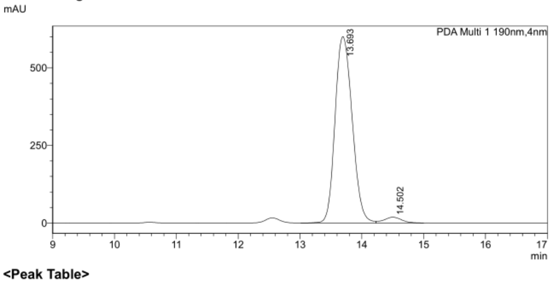

<Peak Table>

PDA Ch1 190nm

Peak#	Ret. Time	Area	Area%	Height	Height%
1	13.693	11574130	96.997	600739	97.014
2	14.502	358372	3.003	18490	2.986
Total		11932503	100.000	619229	100.000

4.4 Synthesis of ethyl (*R*, *E*)-5-methyl-4-(2-oxooxazolidin-3-yl)hex-2-enoate (3ae).

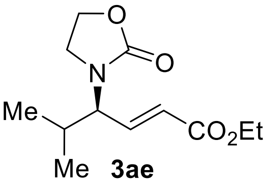

3ae

The general procedure was followed using **1a** (8.7 mg, 0.10 mmol) and **2e** (30.8 mg, 0.20 mmol). After purification by column chromatography (PE/EtOAc = 2:1), **3ae** (19.4 mg, 81%) was obtained as a colorless oil. $[\alpha]^{22}_{D}$ = -35.2 (*c* 0.10, acetone); ^{1}H NMR (400 MHz, $CDCl_3$) δ 6.83 (dd, *J* = 15.6, 8.0 Hz, 1H), 5.98 (d, *J* = 16.0 Hz, 1H), 4.34 (t, *J* = 8.0 Hz, 2H), 4.19 (q, *J* = 7.2 Hz, 2H), 4.03 (t, *J* = 9.2 Hz, 1H), 3.58 – 3.46 (m, 2H), 1.96 – 1.83 (m, 1H), 1.28 (t, *J* = 7.2 Hz, 3H), 0.96 (t, *J* = 7.6 Hz, 6H); ^{13}C NMR (100 MHz, $CDCl_3$) δ 165.91, 158.09, 142.49, 124.75, 62.06, 60.65 (d, *J* = 7.1 Hz), 40.79, 29.50, 19.67, 19.40, 14.19; Enantiomeric excess: 96%, determined by HPLC (Chiralpak IF hexane/*i*-PrOH = 70/30; flow rate 0.6 ml/min; 25 ℃; 190 nm), first peak: t_R = 13.6 min, second peak: t_R = 15.3 min. HRMS (ESI) calcd. for $C_{12}H_{19}NNaO_4$ $[M+Na]^+$: 264.1206, found: 264.1207.

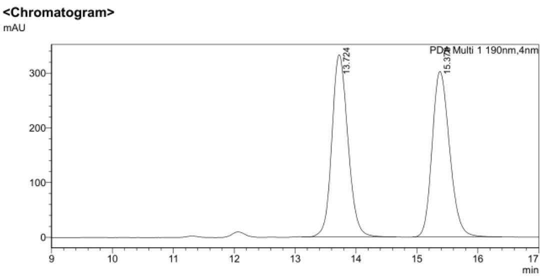

<Peak Table>
PDA Ch1 190nm

Peak#	Ret. Time	Area	Area%	Height	Height%
1	13.724	6100297	50.242	332692	52.362
2	15.378	6041524	49.758	302680	47.638
Total		12141821	100.000	635372	100.000

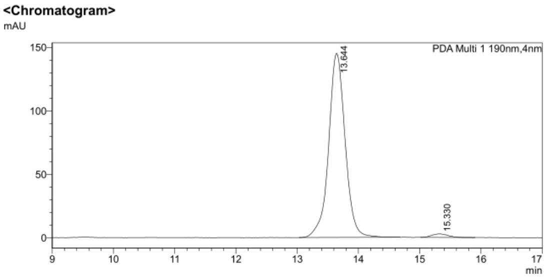

<Peak Table>
PDA Ch1 190nm

Peak#	Ret. Time	Area	Area%	Height	Height%
1	13.644	2713334	98.169	145023	98.117
2	15.330	50611	1.831	2783	1.883
Total		2763945	100.000	147806	100.000

4.5 Synthesis of ethyl (*R*, *E*)-4-(2-oxooxazolidin-3-yl)-6-phenylhex-2-enoate (3af).

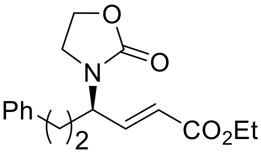

3af

The general procedure was followed using **1a** (8.7 mg, 0.10 mmol) and **2f** (43.3 mg, 0.20 mmol). After purification by column chromatography (PE/EtOAc = 2:1), **3af**

(29.7mg, 98%) was obtained as a colorless oil. [α]$^{22}_{D}$ = -9.3 (*c* 0.25, acetone); ^{1}H NMR (400 MHz, $CDCl_3$) δ 7.33 – 7.26 (m, 2H), 7.25 – 7.14 (m, 3H), 6.83 (dd, *J* = 16.0, 6.0 Hz, 1H), 5.94 (d, *J* = 15.6 Hz, 1H), 4.55 (q, *J* = 7.2 Hz, 1H), 4.37 – 4.28 (m, 1H), 4.27 – 4.16 (m, 3H), 3.51 – 3.34 (m, 2H), 2.76 – 2.59 (m, 2H), 2.00 (q, *J* = 7.6 Hz, 2H), 1.29 (t, *J* = 7.2 Hz, 3H); ^{13}C NMR (100 MHz, $CDCl_3$) δ 165.82, 157.93, 143.86, 140.50, 128.60, 128.32, 126.34, 123.40, 62.09, 60.74, 53.65, 40.38, 32.66, 32.41, 14.20; Enantiomeric excess: 96%, determined by HPLC (Chiralpak IF hexane/*i*-PrOH = 70/30; flow rate 0.6 ml/min; 25 ℃; 254 nm), first peak: t_R = 22.9 min, second peak: t_R = 25.9 min. HRMS (ESI) calcd. for $C_{17}H_{21}NNaO_4$ [M+Na]$^+$: 326.1363, found: 326.1359.

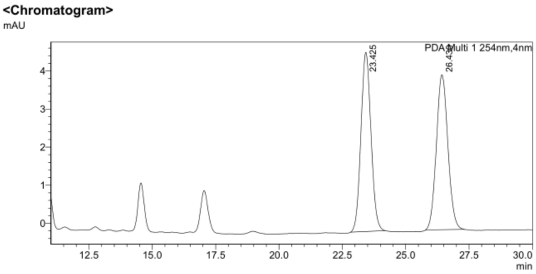

<Peak Table>
PDA Ch1 254nm

Peak#	Ret. Time	Area	Area%	Height	Height%
1	23.425	129998	50.176	4705	53.593
2	26.430	129083	49.824	4074	46.407
Total		259081	100.000	8779	100.000

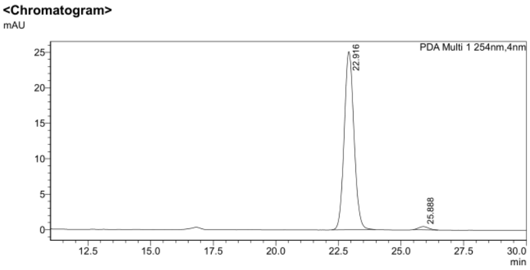

<Peak Table>
PDA Ch1 254nm

Peak#	Ret. Time	Area	Area%	Height	Height%
1	22.916	681327	98.215	25101	98.244
2	25.888	12381	1.785	449	1.756
Total		693708	100.000	25549	100.000

4.6 Synthesis of 1-ethyl 8-methyl (*R*, *E*)-4-(2-oxooxazolidin-3-yl)oct-2-enedioate (3ag).

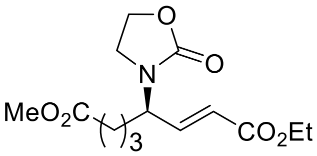

3ag

The general procedure was followed using **1a** (8.7 mg, 0.10 mmol) and **2g** (42.4 mg, 0.20 mmol). After purification by column chromatography (PE/EtOAc = 1:1), **3ag** (28.7 mg, 96%) was obtained as a colorless oil. [α]$^{22}_{D}$ = 10.4 (*c* 0.25, acetone); ^{1}H NMR (400 MHz, $CDCl_3$) δ 6.81 (dd, *J* = 15.6, 6.0 Hz, 1H), 5.95 (dd, *J* = 16.0, 1.6 Hz, 1H), 4.51 (dd, *J* = 12.8, 6.4 Hz, 1H), 4.39 (t, *J* = 8.0 Hz, 2H), 4.21 (q, *J* = 7.2 Hz, 2H), 3.68 (s, 3H), 3.61 – 3.45 (m, 2H), 2.51 – 2.30 (m, 2H), 1.78 – 1.60 (m, 4H), 1.30 (t, *J* = 7.2 Hz, 3H); ^{13}C NMR (100 MHz, $CDCl_3$) δ 173.47, 165.79, 158.08, 143.81, 123.32,

62.17, 60.74, 53.40, 51.64, 40.14, 32.99, 30.28, 21.15, 14.21; Enantiomeric excess: 98%, determined by HPLC (Chiralpak IE hexane/*i*-PrOH = 75/25; flow rate 0.5 ml/min; 25 ℃; 254 nm), first peak: t_R = 64.3 min, second peak: t_R = 66.6 min. HRMS (ESI) calcd. for $C_{14}H_{21}NNaO_4$ $[M+Na]^+$: 322.1263, found: 322.1261.

<Chromatogram>

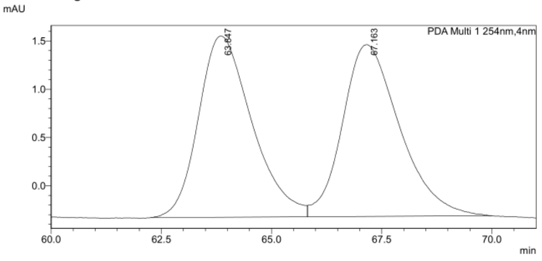

<Peak Table>

PDA Ch1 254nm

Peak#	Ret. Time	Area	Area%	Height	Height%
1	63.847	158580	49.699	1879	51.376
2	67.163	160501	50.301	1779	48.624
Total		319081	100.000	3658	100.000

<Chromatogram>

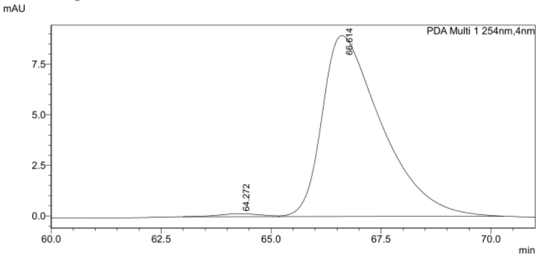

<Peak Table>

PDA Ch1 254nm

Peak#	Ret. Time	Area	Area%	Height	Height%
1	64.272	9820	1.155	146	1.607
2	66.614	840268	98.845	8944	98.393
Total		850088	100.000	9090	100.000

4.7 Synthesis of ethyl (*R, E*)-4-(2-oxooxazolidin-3-yl)trideca-2,12-dienoate (3ah).

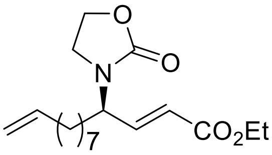

3ah

The general procedure was followed using **1a** (8.7 mg, 0.10 mmol) and **2h** (47.2 mg, 0.20 mmol). After purification by column chromatography (PE/EtOAc = 2:1), **3ah** (27.5 mg, 85%) was obtained as a colorless oil. $[\alpha]^{22}_D$ = -2.4 (*c* 0.25, acetone); ^1H NMR (400 MHz, $CDCl_3$) δ 6.80 (dd, *J* = 16.0, 6.0 Hz, 1H), 5.91 (d, *J* = 15.6 Hz, 1H), 5.84 – 5.72 (m, 1H), 5.03 – 4.86 (m, 2H), 4.51 – 4.43 (m, 1H), 4.34 (t, *J* = 8.0 Hz, 2H), 4.18 (q, *J* = 7.2 Hz, 2H), 3.50 – 3.42 (m, 2H), 2.05 – 1.98 (m , 2H), 1.67 – 1.58 (m, 2H), 1.36 – 1.25 (m, 13H); ^{13}C NMR (100 MHz, $CDCl_3$) δ 165.92, 158.00, 144.31, 139.05, 123.02, 114.21, 62.07, 60.68, 53.70, 40.24, 33.71, 31.02, 29.22, 29.07, 28.94, 28.82, 25.91, 14.20; Enantiomeric excess: 95%, determined by HPLC (Chiralpak IF hexane/*i*-PrOH = 75/25; flow rate 0.5 ml/min; 25 ℃; 190 nm), first peak: t_R = 19.4 min, second peak: t_R = 20.6 min. HRMS (ESI) calcd. for $C_{18}H_{29}NNaO_4$ $[M+Na]^+$: 346.1989, found: 346.1984.

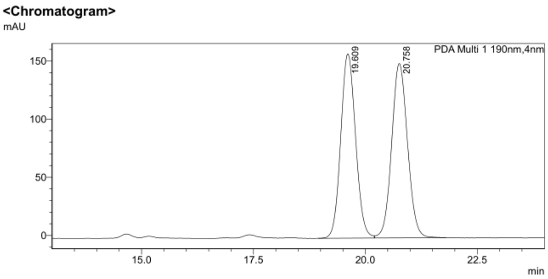

<Peak Table>

PDA Ch1 190nm

Peak#	Ret. Time	Area	Area%	Height	Height%
1	19.609	3652283	49.641	158444	51.358
2	20.758	3705053	50.359	150063	48.642
Total		7357336	100.000	308506	100.000

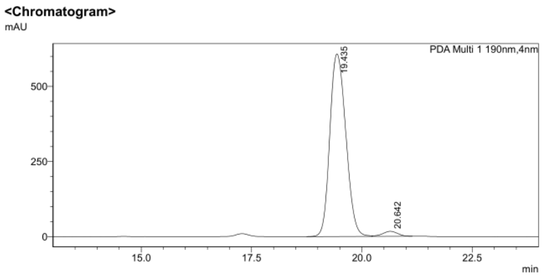

<Peak Table>

PDA Ch1 190nm

Peak#	Ret. Time	Area	Area%	Height	Height%
1	19.435	15333486	97.466	607287	97.327
2	20.642	398668	2.534	16680	2.673
Total		15732154	100.000	623967	100.000

4.8 Synthesis of ethyl (*R*, *E*)-4-(2-oxooxazolidin-3-yl)non-2-en-8-ynoate (3ai).

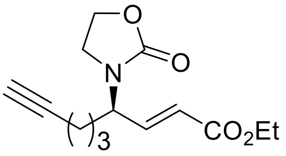

3ai

The general procedure was followed using **1a** (8.7 mg, 0.10 mmol) and **2i** (35.6 mg, 0.20 mmol). After purification by column chromatography (PE/EtOAc = 2:1), **3ai** (25.0 mg, 94%) was obtained as a colorless oil. $[\alpha]^{22}_{D}$ = -7.0 (*c* 0.25, acetone); ^{1}H NMR (400 MHz, $CDCl_3$) δ 6.80 (dd, *J* = 16.0, 6.0 Hz, 1H), 5.94 (dd, *J* = 16.0, 1.6 Hz, 1H), 4.53 – 4.46 (m, 1H), 4.38 – 4.32 (m, 2H), 4.18 (q, *J* = 7.2 Hz, 2H), 3.49 (dd, *J* = 8.8, 7.2 Hz, 2H), 2.31 – 2.17 (m, 2H), 1.96 (t, *J* = 2.86 Hz, 1H), 1.84 – 1.75 (m, 2H), 1.61 – 1.49 (m, 2H), 1.27 (t, *J* = 7.2 Hz, 3H); ^{13}C NMR (100 MHz, $CDCl_3$) δ 165.82, 158.03, 143.87, 123.36, 83.34, 69.27, 62.13, 60.75, 53.24, 40.18, 29.74, 24.66, 17.91, 14.20; Enantiomeric excess: 96%, determined by HPLC (Chiralpak ADH hexane/*i*-PrOH = 85/15; flow rate 0.8 ml/min; 25 ℃; 254 nm), first peak: t_R = 13.8 min, second peak: t_R = 14.6 min. HRMS (ESI) calcd. for $C_{14}H_{19}NNaO_4$ $[M+Na]^+$: 288.1206, found: 288.1201.

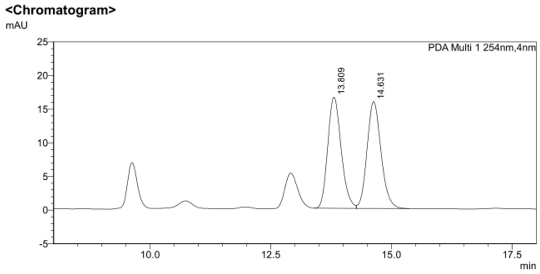

<Peak Table>

PDA Ch1 254nm

Peak#	Ret. Time	Area	Area%	Height	Height%
1	13.809	320483	49.959	16526	50.970
2	14.631	321011	50.041	15897	49.030
Total		641494	100.000	32423	100.000

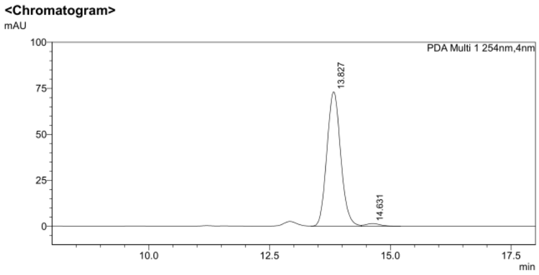

<Peak Table>

PDA Ch1 254nm

Peak#	Ret. Time	Area	Area%	Height	Height%
1	13.827	1455695	97.949	73037	98.019
2	14.631	30481	2.051	1476	1.981
Total		1486176	100.000	74513	100.000

4.9 Synthesis of ethyl (*R*, *E*)-7-chloro-4-(2-oxooxazolidin-3-yl)hept-2-enoate (3aj).

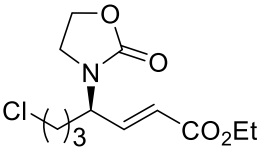

3aj

The general procedure was followed using **1a** (8.7 mg, 0.10 mmol) and **2j** (37.7 mg, 0.20 mmol). After purification by column chromatography (PE/EtOAc = 2:1), **3aj** (27.1 mg, 98%) was obtained as a colorless oil. $[\alpha]^{22}_D$ = -8.1 (*c* 0.25, acetone); ^{1}H NMR (400 MHz, $CDCl_3$) δ 6.83 (dd, *J* = 16.0, 6.0 Hz, 1H), 5.97 (d, *J* = 15.6 Hz, 1H), 4.53 (q, *J* = 6.4 Hz, 1H), 4.39 (t, *J* = 8.0 Hz, 2H), 4.21 (q, *J* = 7.2 Hz, 2H), 3.65 – 3.55 (m, 2H), 3.51 (t, *J* = 8.0 Hz, 2H), 1.90 – 1.76 (m, 4H), 1.30 (t, *J* = 7.2 Hz, 3H); ^{13}C NMR (100 MHz, $CDCl_3$) δ 165.72, 158.02, 143.54, 123.62, 62.16, 60.80, 53.01, 44.22, 40.18, 28.80, 28.11, 14.20; Enantiomeric excess: 97%, determined by HPLC (Chiralpak IF hexane/*i*-PrOH = 75/25; flow rate 0.5 ml/min; 25 ℃; 254 nm), first peak: t_R = 34.5 min, second peak: t_R = 44.6 min. HRMS (ESI) calcd. for $C_{12}H_{18}ClNNaO_4$ $[M+Na]^+$: 298.0817, found: 298.0809.

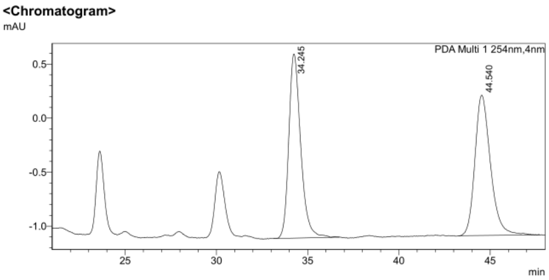

<Peak Table>

PDA Ch1 254nm

Peak#	Ret. Time	Area	Area%	Height	Height%
1	34.245	77153	49.999	1705	56.761
2	44.540	77154	50.001	1298	43.239
Total		154307	100.000	3003	100.000

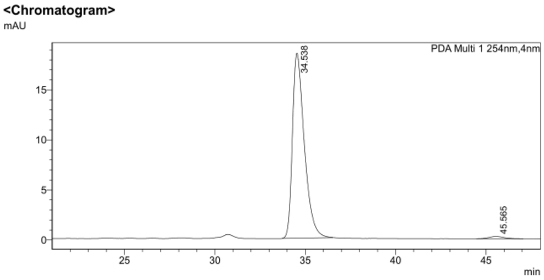

<Peak Table>

PDA Ch1 254nm

Peak#	Ret. Time	Area	Area%	Height	Height%
1	34.538	842878	98.431	18479	98.759
2	45.565	13434	1.569	232	1.241
Total		856312	100.000	18712	100.000

4.10 Synthesis of ethyl (*R*, *E*)-8-acetoxy-4-(2-oxooxazolidin-3-yl)oct-2-enoate (3ak).

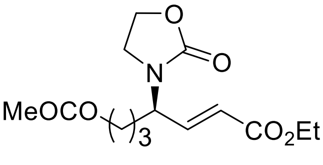

3ak

The general procedure was followed using **1a** (8.7 mg, 0.10 mmol) and **2k** (42.4 mg, 0.20 mmol). After purification by column chromatography (PE/EtOAc = 2:1), **3ak**

(24.7 mg, 96%) was obtained as a colorless oil. $[\alpha]^{22}_{D}$ = -1.4 (*c* 0.25, acetone); ^{1}H NMR (400 MHz, $CDCl_3$) δ 6.80 (dd, *J* = 16.0, 6.0 Hz, 1H), 5.94 (dd, *J* = 16.0, 1.6 Hz, 1H), 4.54 – 4.44 (m, 1H), 4.35 (t, *J* = 8.0 Hz, 2H), 4.19 (q, *J* = 7.2 Hz, 2H), 4.11 – 4.05 (m, 2H), 3.52 – 3.42 (m, 2H), 2.03 (s, 3H), 1.76 – 1.62 (m, 4H), 1.27 (t, *J* = 7.2 Hz, 3H); ^{13}C NMR (100 MHz, $CDCl_3$) δ 170.98, 165.74, 157.96, 143.53, 123.61, 63.51, 62.11, 60.77, 53.49, 40.28, 27.64, 25.25, 20.89, 14.18; Enantiomeric excess: 97%, determined by HPLC (Chiralpak IF hexane/*i*-PrOH = 70/30; flow rate 0.6 ml/min; 25 ℃; 210 nm), first peak: t_R = 34.8 min, second peak: t_R = 36.5 min. HRMS (ESI) calcd. for $C_{14}H_{21}NNaO_6$ $[M+Na]^+$: 322.1261, found: 322.1257.

<Chromatogram>

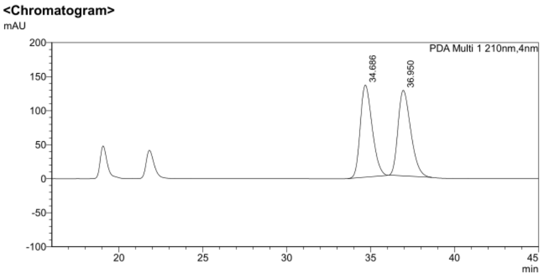

<Peak Table>

PDA Ch1 210nm

Peak#	Ret. Time	Area	Area%	Height	Height%
1	34.686	6676915	50.084	135446	51.843
2	36.950	6654528	49.916	125815	48.157
Total		13331443	100.000	261261	100.000

<Chromatogram>

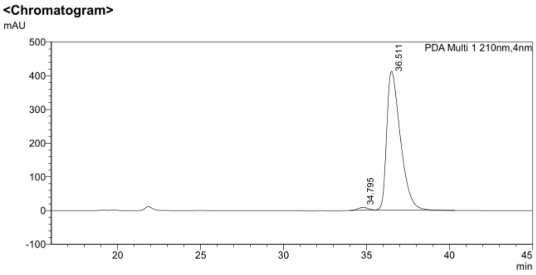

<Peak Table>

PDA Ch1 210nm

Peak#	Ret. Time	Area	Area%	Height	Height%
1	34.795	336591	1.405	7935	1.889
2	36.511	23625498	98.595	412067	98.111
Total		23962089	100.000	420002	100.000

4.11 Synthesis of ethyl (*R*, *E*)-4-cyclopentyl-4-(2-oxooxazolidin-3-yl)but-2-enoate (3al).

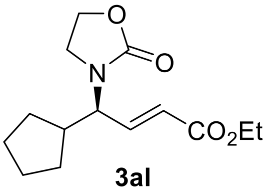

3al

The general procedure was followed using **1a** (8.7 mg, 0.10 mmol) and **2l** (36.1 mg, 0.20 mmol). After purification by column chromatography (PE/EtOAc = 2:1), **3al** (22.0 mg, 82%) was obtained as a colorless oil. $[\alpha]^{22}_{D}$ = -35.8 (*c* 0.10, acetone); ^{1}H NMR (400 MHz, $CDCl_3$) δ 6.84 (dd, *J* = 16.0, 6.8 Hz, 1H), 5.95 (d, *J* = 15.6 Hz, 1H), 4.36 (t, *J* = 8.0 Hz, 2H), 4.21 (dd, *J* = 13.6, 6.8 Hz, 3H), 3.57 – 3.50 (m, 2H), 2.18 – 2.06 (m, 1H), 1.83 – 1.54 (m, 7H), 1.33 – 1.27 (m, 4H); ^{13}C NMR (100 MHz, $CDCl_3$) δ 166.03, 158.05, 143.27, 123.58, 62.09, 60.67, 59.00, 40.89, 40.73, 30.30, 29.75, 25.55, 25.08, 14.20; Enantiomeric excess: 96%, determined by HPLC (Chiralpak IF

hexane/*i*-PrOH = 70/30; flow rate 0.6 ml/min; 25 ℃; 254 nm), first peak: t_R = 17.2 min, second peak: t_R = 19.1 min. HRMS (ESI) calcd. for $C_{14}H_{21}NNaO_4$ $[M+Na]^+$: 290.1363, found: 290.1363.

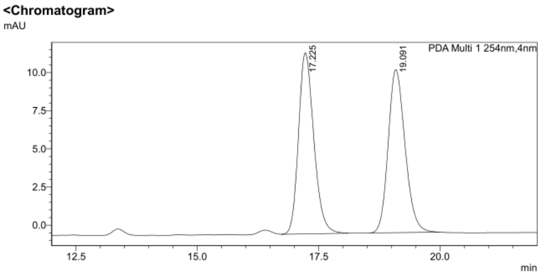

<Peak Table>
PDA Ch1 254nm

Peak#	Ret. Time	Area	Area%	Height	Height%
1	17.225	260612	50.158	11876	52.622
2	19.091	258973	49.842	10692	47.378
Total		519585	100.000	22568	100.000

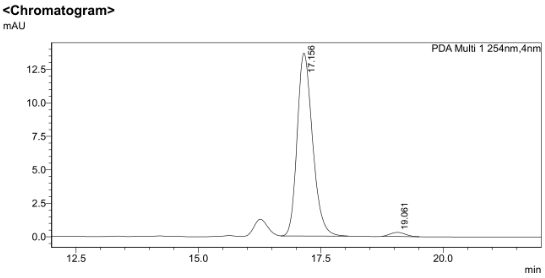

<Peak Table>
PDA Ch1 254nm

Peak#	Ret. Time	Area	Height	Area%	Conc.	Height%
1	17.156	298288	13660	97.872	0.000	97.815
2	19.061	6486	305	2.128	0.000	2.185
Total		304774	13965	100.000		100.000

4.12 Synthesis of ethyl (*R*, *E*)-4-cyclohexyl-4-(2-oxooxazolidin-3-yl)but-2-enoate (3am).

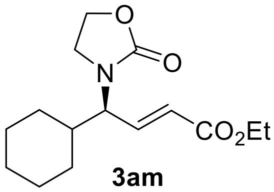

3am

The general procedure was followed using **1a** (8.7 mg, 0.10 mmol) and **2m** (38.9 mg, 0.20 mmol). After purification by column chromatography (PE/EtOAc = 2:1), **3am** (17.0 mg, 60%) was obtained as a colorless oil. $[\alpha]^{22}_D$ = -8.9 (*c* 0.25, acetone); ^{1}H NMR (400 MHz, $CDCl_3$) δ 6.82 (dd, *J* = 15.6, 8.4 Hz, 1H), 5.97 (d, *J* = 15.6 Hz, 1H), 4.32 (q, *J* = 8.0 Hz, 2H), 4.19 (q, *J* = 7.2 Hz, 2H), 4.11 (t, *J* = 9.6 Hz, 1H), 3.57 – 3.44 (m, 2H), 1.80 – 1.62 (m, 6H), 1.61 – 1.52 (m, 1H), 1.28 (t, *J* = 7.2 Hz, 3H), 1.25 – 1.09 (m, 2H), 1.01 (m, 2H); ^{13}C NMR (100 MHz, $CDCl_3$) δ 165.94, 158.10, 142.36, 124.82, 62.03, 60.68, 59.38, 40.82, 38.38, 30.06, 29.56, 26.06, 25.63, 14.20; Enantiomeric excess: 95%, determined by HPLC (Chiralpak IF hexane/*i*-PrOH = 80/20; flow rate 0.6 ml/min; 25 ℃; 254 nm), first peak: t_R = 21.5 min, second peak: t_R = 22.8 min. HRMS (ESI) calcd. for $C_{15}H_{23}NNaO_4$ $[M+Na]^+$: 304.1519, found: 304.1520.

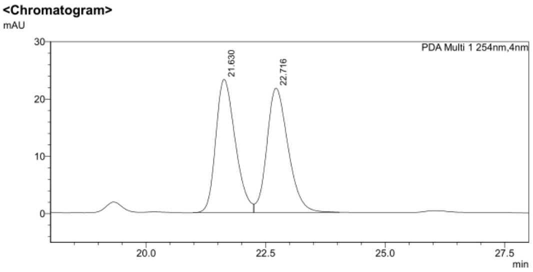

<Peak Table>
PDA Ch1 254nm

Peak#	Ret. Time	Area	Area%	Height	Height%
1	21.630	661444	49.669	23257	51.735
2	22.716	670251	50.331	21697	48.265
Total		1331695	100.000	44954	100.000

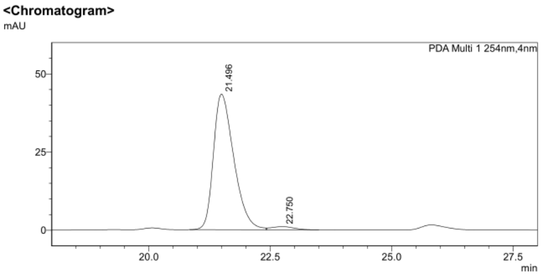

<Peak Table>
PDA Ch1 254nm

Peak#	Ret. Time	Area	Area%	Height	Height%
1	21.496	1269731	97.500	43389	97.681
2	22.750	32561	2.500	1030	2.319
Total		1302292	100.000	44419	100.000

4.13 Synthesis of ethyl (*R*, *E*)-7-(1,3-dioxoisoindolin-2-yl)-4-(2-oxooxazolidin-3-yl)oct-2-enoate (3an).

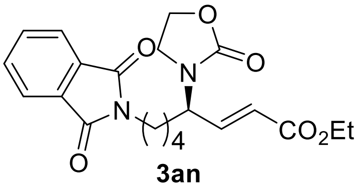

3an

The general procedure was followed using **1a** (8.7 mg, 0.10 mmol) and **2n** (62.6 mg, 0.20 mmol). After purification by column chromatography (PE/EtOAc = 1:1), **3an** (40.1 mg, 97%) was obtained as a colorless oil. $[\alpha]^{22}_{D}$ = -11.8 (*c* 0.25, acetone); ^{1}H NMR (400 MHz, $CDCl_3$) δ 7.78 – 7.70 (m, 2H), 7.70 – 7.60 (m, 2H), 6.74 (dd, *J* = 16.0, 6.0 Hz, 1H), 5.86 (d, *J* = 16.0 Hz, 1H), 4.39 (dd, *J* = 13.6, 6.8 Hz, 1H), 4.29 (t, *J* = 7.6 Hz, 2H), 4.11 (q, *J* = 7.2 Hz, 2H), 3.60 (t, *J* = 6.8 Hz, 2H), 3.51 – 3.38 (m, 2H), 1.74 – 1.59 (m, 4H), 1.38 – 1.26 (m, 2H), 1.21 (t, *J* = 7.2 Hz, 3H); ^{13}C NMR (100 MHz, $CDCl_3$) δ 168.32, 165.74, 157.97, 143.93, 133.97, 131.99, 123.23, 123.15, 62.14, 60.62, 53.64, 40.26, 37.27, 30.40, 27.99, 23.08, 14.16; Enantiomeric excess: 96%, determined by HPLC (Chiralpak ADH hexane/*i*-PrOH = 70/30; flow rate 0.8 ml/min; 25 ℃; 254 nm), first peak: t_R = 22.4 min, second peak: t_R = 30.2 min. HRMS (ESI) calcd. for $C_{21}H_{24}N_2NaO_6$ $[M+Na]^+$: 423.1527, found: 423.1521.

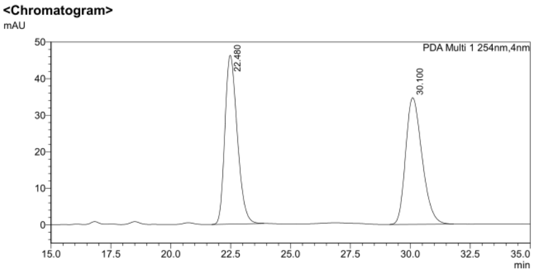

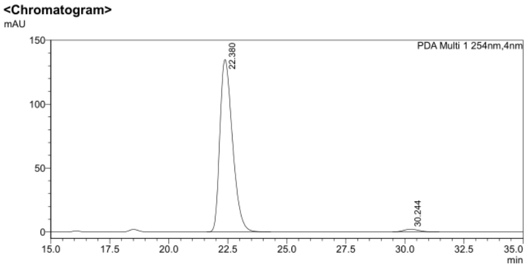

<Peak Table>
PDA Ch1 254nm

Peak#	Ret. Time	Area	Area%	Height	Height%
1	22.480	1660872	50.065	46177	57.121
2	30.100	1656543	49.935	34664	42.879
Total		3317415	100.000	80841	100.000

<Peak Table>
PDA Ch1 254nm

Peak#	Ret. Time	Area	Area%	Height	Height%
1	22.380	5029273	98.062	134974	98.424
2	30.244	99410	1.938	2161	1.576
Total		5128684	100.000	137135	100.000

4.14 Synthesis of methyl (*R*, *E*)-4-(2-oxooxazolidin-3-yl)hex-2-enoate (3ao).

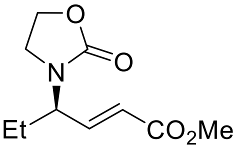

3ao

The general procedure was followed using **1a** (8.7 mg, 0.10 mmol) and **2o** (25.2 mg, 0.20 mmol). After purification by column chromatography (PE/EtOAc = 2:1), **3ao** (20.9 mg, 98%) was obtained as a colorless oil. $[\alpha]^{22}_D$ = -0.9 (*c* 0.25, acetone); ^{1}H NMR (400 MHz, $CDCl_3$) δ 6.83 (dd, *J* = 16.0, 6.0 Hz, 1H), 5.94 (dd, *J* = 16.0, 1.6 Hz, 1H), 4.45 – 4.39 (m, 1H), 4.36 (dd, *J* = 8.8, 7.2 Hz, 2H), 3.75 (s, 3H), 3.52 – 3.44 (m, 2H), 1.78 – 1.64 (m, 2H), 0.97 (t, *J* = 7.2 Hz, 3H); ^{13}C NMR (100 MHz, $CDCl_3$) δ 166.28, 158.03, 144.34, 122.83, 62.03, 55.32, 51.68, 40.26, 24.28, 10.50; Enantiomeric excess: 96%, determined by HPLC (Chiralpak IF hexane/*i*-PrOH = 70/30; flow rate 0.6 ml/min; 25 ℃; 190 nm), first peak: t_R = 20.3 min, second peak: t_R = 22.2 min. HRMS (ESI) calcd. for $C_{10}H_{15}NNaO_4$ $[M+Na]^+$: 236.0893, found: 236.0893.

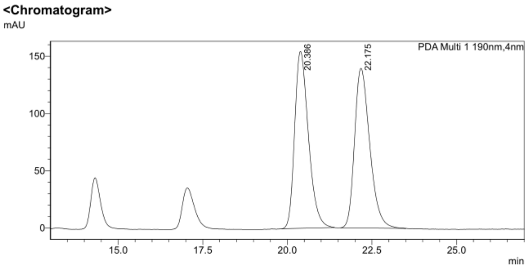

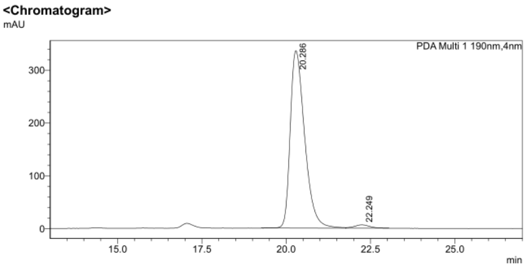

<Peak Table>
PDA Ch1 190nm

Peak#	Ret. Time	Area	Area%	Height	Height%
1	20.386	4367733	50.111	154569	52.561
2	22.175	4348411	49.889	139506	47.439
Total		8716143	100.000	294076	100.000

<Peak Table>
PDA Ch1 190nm

Peak#	Ret. Time	Area	Area%	Height	Height%
1	20.286	10010726	98.030	335831	98.150
2	22.249	201176	1.970	6330	1.850
Total		10211903	100.000	342161	100.000

4.15 Synthesis of tert-butyl (*R*, *E*)-4-(2-oxooxazolidin-3-yl)hex-2-enoate (3ap).

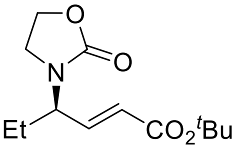

3ap

The general procedure was followed using **1a** (8.7 mg, 0.10 mmol) and **2p** (33.6 mg, 0.20 mmol). After purification by column chromatography (PE/EtOAc = 2:1), **3ap** (21.0 mg, 87%) was obtained as a colorless oil. $[\alpha]^{22}_{D}$ = -1.9 (*c* 0.25, acetone); ^{1}H NMR (400 MHz, $CDCl_3$) δ 6.72 (dd, *J* = 15.6, 6.0 Hz, 1H), 5.86 (dd, *J* = 15.6, 1.6 Hz, 1H), 4.43 – 4.34 (m, 3H), 3.54 – 3.44 (m, 2H), 1.78 – 1.60 (m, 2H), 1.49 (s, 9H), 0.97 (t, *J* = 7.2 Hz, 3H); ^{13}C NMR (100 MHz, $CDCl_3$) δ 165.26, 158.15, 142.83, 124.87, 80.93, 62.08, 55.20, 40.15, 28.07, 24.28, 10.61; Enantiomeric excess: 93%, determined by HPLC (Chiralpak IF hexane/*i*-PrOH = 80/20; flow rate 0.6 ml/min; 25 ℃; 210 nm), first peak: t_R = 16.6 min, second peak: t_R = 17.8 min. HRMS (ESI) calcd. for $C_{13}H_{21}NNaO_4$ $[M+Na]^+$: 278.1363, found: 278.1359.

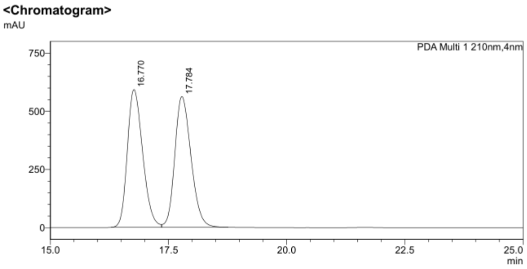

<Peak Table>
PDA Ch1 210nm

Peak#	Ret. Time	Area	Area%	Height	Height%
1	16.770	13375769	49.919	592107	51.316
2	17.784	13419278	50.081	561743	48.684
Total		26795047	100.000	1153850	100.000

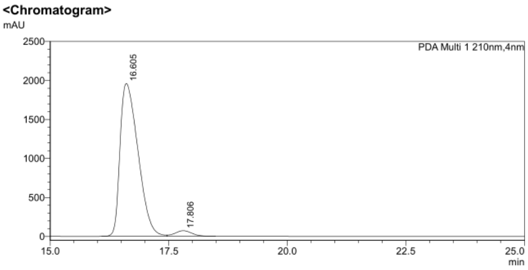

<Peak Table>
PDA Ch1 210nm

Peak#	Ret. Time	Area	Area%	Height	Height%
1	16.605	51739262	96.626	1961604	96.446
2	17.806	1806511	3.374	72294	3.554
Total		53545774	100.000	2033899	100.000

4.16 Synthesis of benzyl (*R*, *E*)-4-(2-oxooxazolidin-3-yl)hex-2-enoate (3aq).

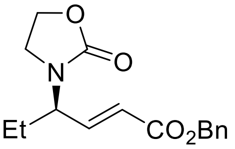

3aq

The general procedure was followed using **1a** (8.7 mg, 0.10 mmol) and **2q** (40.5 mg, 0.20 mmol). After purification by column chromatography (PE/EtOAc = 2:1), **3aq** (28.3 mg, 98%) was obtained as a colorless oil. $[\alpha]^{22}_{D}$ = -5.0 (*c* 0.25, acetone); ^{1}H NMR (400 MHz, $CDCl_3$) δ 7.42 – 7.30 (m, 5H), 6.87 (dd, *J* = 15.6, 5.6 Hz, 1H), 5.99

(dd, *J* = 15.6, 0.8 Hz, 1H), 5.19 (s, 2H), 4.45 – 4.38 (m, 1H), 4.38 – 4.33 (m,2H), 3.51 – 3.43 (m, 2H), 1.77 – 1.61 (m, 2H), 0.97 (t, *J* = 7.2 Hz, 3H); ^{13}C NMR (100 MHz, $CDCl_3$) δ 165.72, 158.13, 144.84, 135.68, 128.62, 128.38, 122.76, 66.56, 62.10, 55.28, 40.18, 24.21, 10.60; Enantiomeric excess: 97%, determined by HPLC (Chiralpak IF hexane/*i*-PrOH = 70/30; flow rate 0.6 ml/min; 25 ℃; 254 nm), first peak: t_R = 20.8 min, second peak: t_R = 22.5 min. HRMS (ESI) calcd. for $C_{16}H_{19}NNaO_4$ $[M+Na]^+$: 312.1206, found: 312.1209.

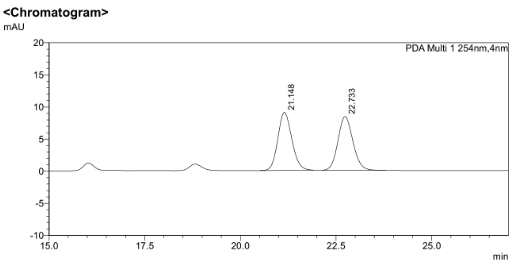

<Peak Table>
PDA Ch1 254nm

Peak#	Ret. Time	Area	Area%	Height	Height%
1	21.148	229382	50.205	9023	51.870
2	22.733	227506	49.795	8372	48.130
Total		456888	100.000	17395	100.000

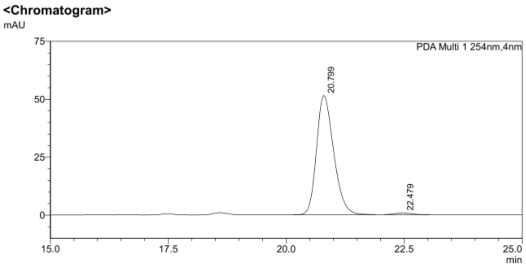

<Peak Table>
PDA Ch1 254nm

Peak#	Ret. Time	Area	Area%	Height	Height%
1	20.799	1306288	98.541	51477	98.494
2	22.479	19345	1.459	787	1.506
Total		1325633	100.000	52264	100.000

4.17 Synthesis of ethyl (*R*, *E*)-4-(2-oxooxazolidin-3-yl)hex-2-enoate (3aa).

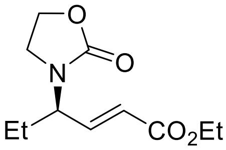

3aa

The general procedure was followed using **1a** (8.7 mg, 0.10 mmol) and **2a** (28.0 mg, 0.20 mmol). After purification by column chromatography (PE/EtOAc = 2:1), **3aa** (20.4 mg, 90%) was obtained as a colorless oil. $[\alpha]^{22}_D$ = -1.4 (*c* 0.25, acetone); ^{1}H NMR (400 MHz, $CDCl_3$) δ 6.80 (dd, *J* = 15.6, 5.6 Hz, 1H), 5.91 (dd, *J* = 15.6, 1.6 Hz, 1H), 4.49 – 4.28 (m, 3H), 4.18 (q, *J* = 6.8 Hz, 2H), 3.55 – 3.35 (m, 2H), 1.75 – 1.60 (m, 2H), 1.27 (t, *J* = 7.2 Hz, 3H), 0.95 (t, *J* = 7.2 Hz, 3H); ^{13}C NMR (100 MHz, $CDCl_3$) δ 165.94, 158.15, 144.10, 123.13, 62.11, 60.72, 55.25, 40.17, 24.23, 14.21, 10.60; Enantiomeric excess: 97%, determined by HPLC (Chiralpak IF hexane/*i*-PrOH = 70/30; flow rate 0.6 ml/min; 25 ℃; 190 nm), first peak: t_R = 17.5 min, second peak: t_R = 19.0 min. HRMS (ESI) calcd. for $C_{11}H_{17}NNaO_4$ $[M+Na]^+$: 250.1050, found: 250.1043.

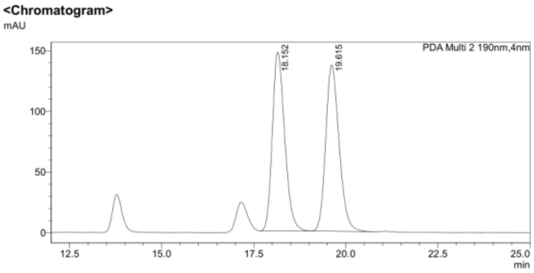

<Peak Table>

PDA Ch2 190nm

Peak#	Ret. Time	Area	Area%	Height	Height%
1	18.152	3440757	49.780	147395	51.800
2	19.615	3471149	50.220	137149	48.200
Total		6911906	100.000	284544	100.000

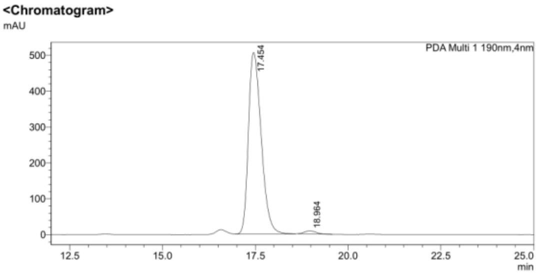

<Peak Table>

PDA Ch1 190nm

Peak#	Ret. Time	Area	Area%	Height	Height%
1	17.454	12112507	98.501	506566	98.330
2	18.964	184339	1.499	8605	1.670
Total		12296845	100.000	515171	100.000

4.18 Synthesis of ethyl (*R*, *E*)-4-(5,5-dimethyl-2-oxooxazolidin-3-yl)hex-2-enoate (3ba).

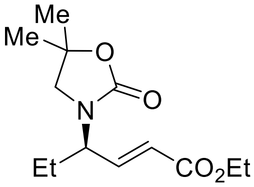

3ba

The general procedure was followed using **1b** (11.5 mg, 0.10 mmol) and **2a** (28.0 mg, 0.20 mmol). After purification by column chromatography (PE/EtOAc = 2:1), **3ba** (17.2 mg, 67%) was obtained as a colorless oil. $[\alpha]^{22}_{D}$ = -30.2 (*c* 0.10, acetone); ^{1}H NMR (400 MHz, $CDCl_3$) δ 6.80 (dd, *J* = 15.6, 6.0 Hz, 1H), 5.92 (d, *J* = 16.0 Hz, 1H), 4.45 – 4.36 (m, 1H), 4.19 (q, *J* = 7.2 Hz, 2H), 3.18 (q, *J* = 8.4 Hz, 2H), 1.71 – 1.59 (m, 2H), 1.46 (s, 6H), 1.28 (t, *J* = 7.2 Hz, 3H), 0.96 (t, *J* = 7.6 Hz, 3H); ^{13}C NMR (100 MHz, $CDCl_3$) δ 165.96, 157.24, 144.25, 123.18, 77.73, 60.67, 54.91, 52.29, 27.52, 27.38, 24.27, 14.20, 10.55; Enantiomeric excess: 97%, determined by HPLC (Chiralpak IF hexane/*i*-PrOH = 90/10; flow rate 0.5 ml/min; 25 °C; 254 nm), first peak: t_R = 52.2 min, second peak: t_R = 56.9 min. HRMS (ESI) calcd. for $C_{13}H_{21}NNaO_4$ $[M+Na]^+$: 278.1356, found: 278.1363.

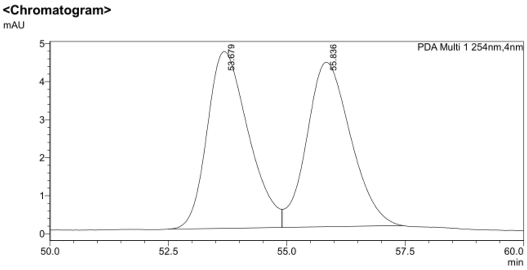

<Peak Table>

PDA Ch1 254nm

Peak#	Ret. Time	Area	Area%	Height	Height%
1	53.679	281728	50.248	4648	51.797
2	55.836	278943	49.752	4326	48.203
Total		560671	100.000	8974	100.000

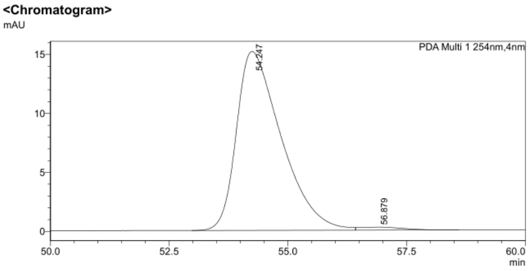

<Peak Table>

PDA Ch1 254nm

Peak#	Ret. Time	Area	Area%	Height	Height%
1	54.247	997129	98.434	15173	98.377
2	56.879	15867	1.566	250	1.623
Total		1012996	100.000	15423	100.000

4.19 Synthesis of ethyl (*R*, *E*)-4-((*R*)-5-methyl-2-oxooxazolidin-3-yl)hex-2-enoate (3ca).

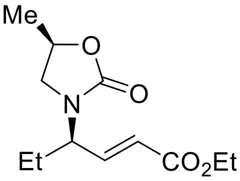

3ca

The general procedure was followed using **1c** (10.1 mg, 0.10 mmol) and **2a** (28.0 mg, 0.20 mmol). After purification by column chromatography (PE/EtOAc = 1:1), **3ca** (20.0 mg, 83%) was obtained as a colorless oil. $[\alpha]^{22}_{D}$ = 11.4 (*c* 0.25, acetone); ^{1}H NMR (400 MHz, $CDCl_3$) δ 6.79 (dd, *J* = 15.6, 6.0 Hz, 1H), 5.92 (d, *J* = 16.0 Hz, 1H), 4.70 – 4.60 (m, 1H), 4.37 (dd, *J* = 14.8, 6.4 Hz, 1H), 4.19 (q, *J* = 7.2 Hz, 2H), 3.53 (t, *J* = 8.0 Hz, 1H), 3.07 – 3.00 (m, 1H), 1.75 – 1.57 (m, 2H), 1.43 (d, *J* = 6.4 Hz, 3H), 1.28 (t, *J* = 7.2 Hz, 3H), 0.95 (t, *J* = 7.6 Hz, 3H); ^{13}C NMR (100 MHz, $CDCl_3$) δ 165.94, 157.73, 144.02, 123.31, 70.49, 60.68, 55.08, 46.95, 24.31, 20.67, 14.20, 10.56; Diastereoisomeric exces: > 99%, determined by HPLC (Chiralpak ID + ID hexane/*i*-PrOH = 95/5; flow rate 1.2 ml/min; 25 ℃; 190 nm), first peak: t_R = 225.8 min; HRMS (ESI) calcd. for $C_{12}H_{19}NNaO_4$ $[M+Na]^+$: 264.1206, found: 264.1204.

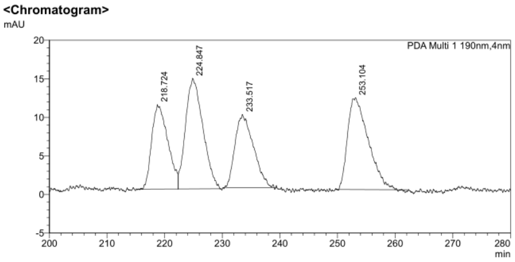

<Peak Table>

PDA Ch1 190nm

Peak#	Ret. Time	Area	Area%	Height	Height%
1	218.724	2134132	20.883	10987	23.454
2	224.847	2999082	29.347	14374	30.684
3	233.517	2118146	20.727	9534	20.354
4	253.104	2968003	29.043	11949	25.508
Total		10219363	100.000	46844	100.000

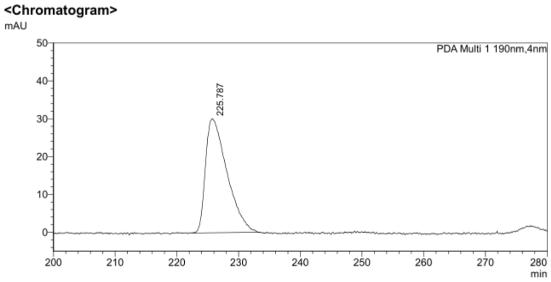

<Peak Table>

PDA Ch1 190nm

Peak#	Ret. Time	Area	Area%	Height	Height%
1	225.787	7289744	100.000	30133	100.000
Total		7289744	100.000	30133	100.000

4.20 Synthesis of ethyl (*R*, *E*)-4-((*S*)-5-methyl-2-oxooxazolidin-3-yl)hex-2-enoate (3da).

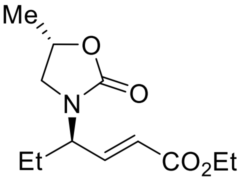

3da

The general procedure was followed using **1d** (10.1 mg, 0.10 mmol) and **2a** (28.0 mg, 0.20 mmol). After purification by column chromatography (PE/EtOAc = 1:1), **3da** (18.3 mg, 76%) was obtained as a colorless oil. $[\alpha]^{22}_{D}$ = -48.2 (*c* 0.10, acetone); ^{1}H NMR (400 MHz, $CDCl_3$) δ 6.80 (dd, *J* = 16.0, 6.0 Hz, 1H), 5.91 (dd, *J* = 16.0, 1.6 Hz, 1H), 4.73 – 4.59 (m, 1H), 4.46 – 4.34 (m, 1H), 4.18 (q, *J* = 7.2 Hz, 2H), 3.55 (t, *J* = 8.4 Hz, 1H), 3.01 (dd, *J* = 8.4, 6.9 Hz, 1H), 1.73 – 1.58 (m, 2H), 1.43 (d, *J* = 6.0 Hz, 3H), 1.27 (t, *J* = 7.2 Hz, 3H), 0.95 (t, *J* = 7.2 Hz, 3H); ^{13}C NMR (100 MHz, $CDCl_3$) δ 165.94, 157.72, 144.32, 122.97, 70.35, 60.67, 55.01, 46.95, 24.18, 20.78, 14.19, 10.54; Diastereoisomeric exces: 97%, determined by HPLC (Chiralpak ID + ID hexane/*i*-PrOH = 95/5; flow rate 1.2 ml/min; 25 ℃; 190 nm), first peak: t_R = 220.4 min, second peak: t_R = 229.9 min; HRMS (ESI) calcd. for $C_{12}H_{19}NNaO_4$ $[M+Na]^+$: 264.1206, found: 264.1201.

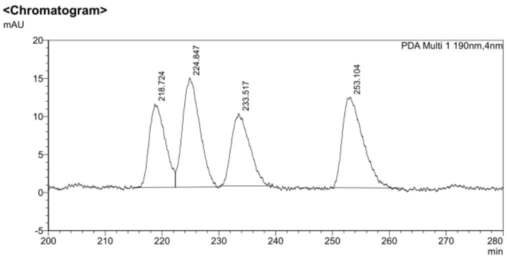

<Peak Table>

PDA Ch1 190nm

Peak#	Ret. Time	Area	Area%	Height	Height%
1	218.724	2134132	20.883	10987	23.454
2	224.847	2999082	29.347	14374	30.684
3	233.517	2118146	20.727	9534	20.354
4	253.104	2968003	29.043	11949	25.508
Total		10219363	100.000	46844	100.000

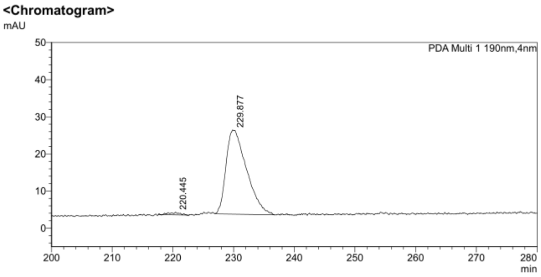

<Peak Table>

PDA Ch1 190nm

Peak#	Ret. Time	Area	Area%	Height	Height%
1	220.445	85373	1.577	719	3.073
2	229.877	5327334	98.423	22683	96.927
Total		5412707	100.000	23402	100.000

4.21 Synthesis of ethyl (*R*, *E*)-4-((*R*)-2-oxo-5-phenyloxazolidin-3-yl)hex-2-enoate (3ea).

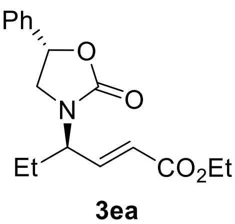

3ea

The general procedure was followed using **1e** (16.3 mg, 0.10 mmol) and **2a** (28.0 mg, 0.20 mmol). After purification by column chromatography (PE/EtOAc = 2:1), **3ea** (26.7 mg, 88%) was obtained as a colorless oil. $[\alpha]^{22}_{D}$ = 1.0 (*c* 0.25, acetone);^{1}H NMR (400 MHz, $CDCl_3$) δ 7.45 – 7.31 (m, 5H), 6.85 (dd, *J* = 16.0, 6.0 Hz, 1H), 5.97 (dd, *J* = 16.0, 1.6 Hz, 1H), 5.58 – 5.49 (m, 1H), 4.52 – 4.42 (m, 1H), 4.21 (q, *J* = 7.2 Hz,

2H), 3.84 (t, *J* = 8.8 Hz, 1H), 3.33 (dd, *J* = 8.4, 7.6 Hz, 1H), 1.72 – 1.57 (m, 2H), 1.30 (t, *J* = 7.2 Hz, 3H), 0.92 (t, *J* = 7.2 Hz, 3H); ^{13}C NMR (100 MHz, $CDCl_3$) δ 165.92, 157.61, 144.22, 138.60, 128.99, 128.91, 125.41, 123.11, 74.81, 60.73, 55.24, 47.98, 24.18, 14.22, 10.55; Diastereoisomeric exces: 98%, determined by HPLC (Chiralpak ODH hexane/*i*-PrOH = 90/10; flow rate 0.8 ml/min; 25 ℃; 254 nm), first peak: t_R = 20.5 min, second peak: t_R = 23.4 min; HRMS (ESI) calcd. for $C_{17}H_{21}NNaO_4$ $[M+Na]^+$: 326.1363, found: 326.1360.

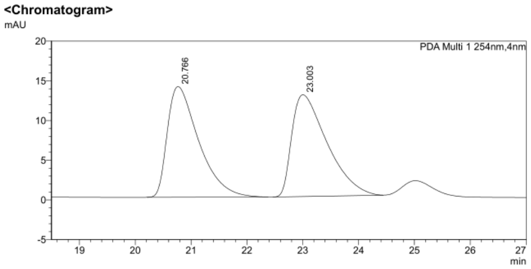

<Peak Table>
PDA Ch1 254nm

Peak#	Ret. Time	Area	Area%	Height	Height%
1	20.766	546542	49.215	13947	52.098
2	23.003	563985	50.785	12824	47.902
Total		1110527	100.000	26771	100.000

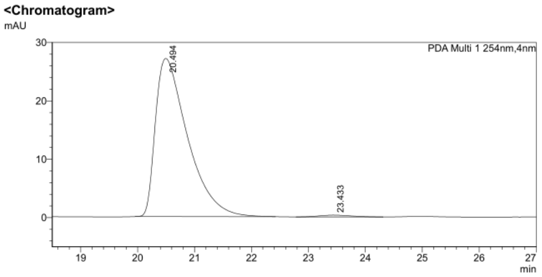

<Peak Table>
PDA Ch1 254nm

Peak#	Ret. Time	Area	Area%	Height	Height%
1	20.494	1065251	98.882	27113	98.870
2	23.433	12045	1.118	310	1.130
Total		1077296	100.000	27422	100.000

4.22 Synthesis of ethyl (4*R*,*E*)-4-(5-methyl-2-oxooxazolidin-3-yl)hex-2-enoate (3fa).

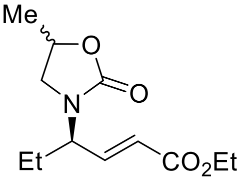

3fa

The general procedure was followed using **1f** (20.2 mg, 0.20 mmol) and **2a** (56.0 mg, 0.40 mmol). After purification by column chromatography (PE/EtOAc = 1:1), **3fa** (34.4 mg, 71%) was obtained as a colorless oil. $[\alpha]^{22}_D$ = -18.6 (*c* 0.25, acetone); ^{1}H NMR (400 MHz, $CDCl_3$) δ 6.80 (dd, *J* = 6.0, 3.6 Hz, 1H), 6.77 (dd, *J* = 6.0, 3.6 Hz, 1H), 5.92 (dd, *J* = 4.8, 1.6 Hz, 1H), 5.88 (dd, *J* = 4.8, 1.6 Hz, 1H), 4.71 – 4.59 (m, 2H), 4.42 – 4.32 (m, 2H), 4.18 (qd, *J* = 7.2, 1.6 Hz, 4H), 3.54 (dd, *J* = 16.0, 8.4 Hz, 2H), 3.06 – 2.97 (m, 2H), 1.72 – 1.59 (m, 4H), 1.43 (s, 3H), 1.41 (s, 3H), 1.27 (td, *J* = 7.2, 1.2 Hz, 7H), 0.94 (td, *J* = 7.6, 2.4 Hz, 7H); ^{13}C NMR (100 MHz, $CDCl_3$) δ 165.93 (s), 157.72 (s), 144.33 (s), 144.02 (s), 123.29 (s), 122.95 (s), 70.49 (s), 70.36 (s), 60.66 (s), 55.08 (s), 55.00 (s), 46.94 (d, *J* = 1.7 Hz), 24.30 (s), 24.17 (s), 20.77 (s), 20.66 (s),

14.18 (s), 10.55 (s); Diastereoisomeric ratio: 1: 1.1, enantiomeric excess: 97%, determined by HPLC (Chiralpak ID + ID hexane/*i*-PrOH = 95/5; flow rate 1.2 ml/min; 25 ℃; 190 nm), first peak: t_R = 236.9 min, second peak: t_R = 243.9 min, third peak: t_R = 255.1 min, fourth peak: t_R = 279.0 min; HRMS (ESI) calcd. for $C_{12}H_{19}NNaO_4$ $[M+Na]^+$: 264.1206, found: 264.1203.

<Chromatogram>

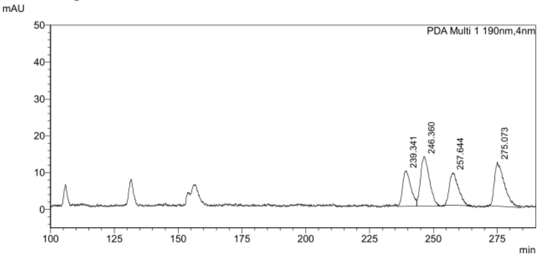

<Peak Table>

PDA Ch1 190nm

Peak#	Ret. Time	Area	Area%	Height	Height%
1	239.341	2150054	20.107	9716	22.037
2	246.360	3202484	29.950	13462	30.534
3	257.644	2159131	20.192	8925	20.242
4	275.073	3181202	29.751	11986	27.187
Total		10692871	100.000	44089	100.000

<Chromatogram>

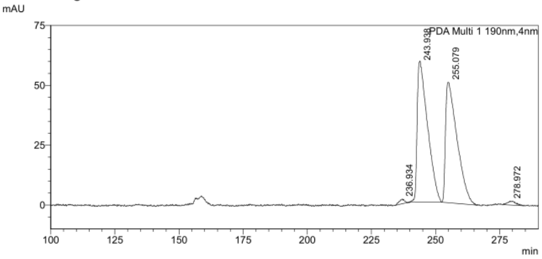

<Peak Table>

PDA Ch1 190nm

Peak#	Ret. Time	Area	Area%	Height	Height%
1	236.934	282988	0.859	2053	1.814
2	243.938	16541763	50.184	58999	52.151
3	255.079	15759958	47.812	50420	44.568
4	278.972	377756	1.146	1659	1.467
Total		32962464	100.000	113130	100.000

4.23 Synthesis of ethyl (*R*, *E*)-4-(2-oxothiazolidin-3-yl)hex-2-enoate (3ga).

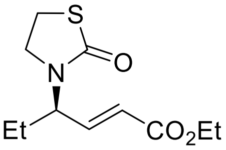

3ga

The general procedure was followed using **1g** (10.3 mg, 0.10 mmol) and **2a** (28.0 mg, 0.20 mmol). After purification by column chromatography (PE/EtOAc = 5:1), **3ga** (23.8 mg, 98%) was obtained as a colorless oil. $[\alpha]^{22}_D$ = -7.1 (*c* 0.25, acetone); ^{1}H NMR (400 MHz, $CDCl_3$) δ 6.82 (dd, *J* = 15.6, 5.6 Hz, 1H), 5.92 (dd, *J* = 16.0, 1.6 Hz, 1H), 4.76 – 4.64 (m, 1H), 4.26 – 4.15 (m, 2H), 3.57 – 3.50 (m, 2H), 3.36 – 3.21 (m, 2H), 1.78 – 1.62 (m, 2H), 1.30 (t, *J* = 7.2 Hz, 3H), 0.96 (t, *J* = 7.2 Hz, 3H); ^{13}C NMR (100 MHz, $CDCl_3$) δ 172.19, 165.95, 144.59, 122.98, 60.69, 55.44, 44.08, 26.03, 24.30, 14.21, 10.64; Enantiomeric excess: 97%, determined by HPLC (Chiralpak IE hexane/*i*-PrOH = 85/15; flow rate 0.6 ml/min; 25 ℃; 254 nm), first peak: t_R = 28.7 min, second peak: t_R = 32.0 min. HRMS (ESI) calcd. for $C_{11}H_{17}NNaO_4S$ $[M+Na]^+$: 266.0821, found: 266.0817.

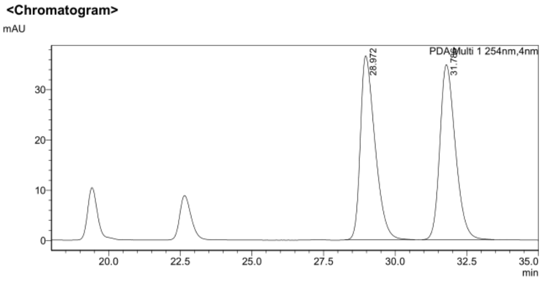

<Peak Table>

PDA Ch1 254nm

Peak#	Ret. Time	Area	Area%	Height	Height%
1	28.972	1305261	49.807	36620	51.268
2	31.786	1315373	50.193	34809	48.732
Total		2620634	100.000	71429	100.000

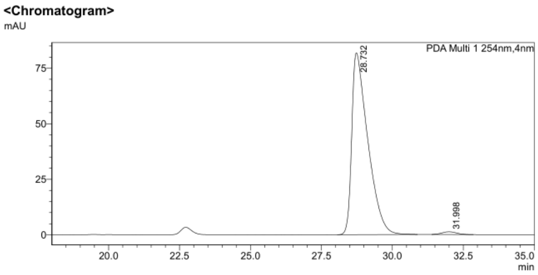

<Peak Table>

PDA Ch1 254nm

Peak#	Ret. Time	Area	Area%	Height	Height%
1	28.732	3223974	98.671	81863	98.549
2	31.998	43439	1.329	1205	1.451
Total		3267414	100.000	83068	100.000

4.24 Synthesis of ethyl (*R*, *E*)-4-(2-oxothiazolidin-3-yl)hept-2-enoate (3gc).

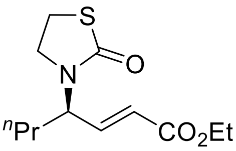

3gc

The general procedure was followed using **1g** (10.3 mg, 0.10 mmol) and **2c** (30.8 mg, 0.20 mmol). After purification by column chromatography (PE/EtOAc = 5:1), **3gc** (24.0 mg, 93%) was obtained as a colorless oil. $[\alpha]^{22}_{D}$ = -6.2 (*c* 0.10, acetone); ^{1}H NMR (400 MHz, $CDCl_3$) δ 6.80 (dd, *J* = 16.0, 5.6 Hz, 1H), 5.89 (dd, *J* = 16.0, 1.6 Hz, 1H), 4.82 – 4.73 (m, 1H), 4.19 (q, *J* = 7.2 Hz, 2H), 3.55 – 3.48 (m, 2H), 3.33 – 3.18 (m, 2H), 1.67 – 1.59 (m, 2H), 1.38 – 1.30 (m, 2H), 1.28 (t, *J* = 7.2 Hz, 3H), 0.94 (t, *J* = 7.6 Hz, 3H); ^{13}C NMR (100 MHz, $CDCl_3$) δ 172.03, 165.96, 144.81, 122.88, 60.67, 53.62, 44.13, 33.16, 26.01, 19.26, 14.21, 13.63; Enantiomeric excess: 96%, determined by HPLC (Chiralpak IF hexane/*i*-PrOH = 85/15; flow rate 0.6 ml/min; 25 ℃; 254 nm), first peak: t_R = 22.3 min, second peak: t_R = 24.9 min. HRMS (ESI) calcd. for $C_{12}H_{19}NNaO_3S$ $[M+Na]^+$: 280.0978, found: 280.0979.

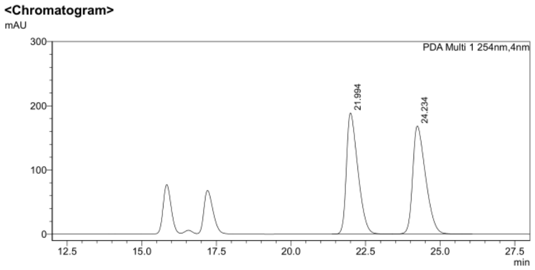

<Peak Table>

PDA Ch1 254nm

Peak#	Ret. Time	Area	Area%	Height	Height%
1	21.994	5048170	49.826	188907	52.841
2	24.234	5083406	50.174	168596	47.159
Total		10131577	100.000	357504	100.000

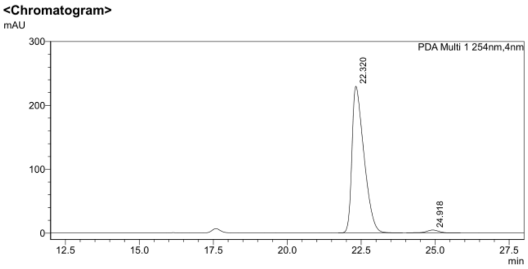

<Peak Table>

PDA Ch1 254nm

Peak#	Ret. Time	Area	Area%	Height	Height%
1	22.320	6492968	97.968	229785	98.067
2	24.918	134641	2.032	4528	1.933
Total		6627610	100.000	234314	100.000

4.25 Synthesis of ethyl (*R*, *E*)-4-(2-oxothiazolidin-3-yl)tetradec-2-enoate (3gd).

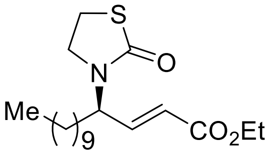

3gd

The general procedure was followed using **1g** (10.3 mg, 0.10 mmol) and **2d** (50.5 mg, 0.20 mmol). After purification by column chromatography (PE/EtOAc = 5:1), **3gd** (35.2 mg, 99%) was obtained as a colorless oil. $[\alpha]^{22}_{D}$ = -5.0 (*c* 0.25, acetone); ^{1}H NMR (400 MHz, $CDCl_3$) δ 6.79 (dd, *J* = 16.0, 5.6 Hz, 1H), 5.89 (d, *J* = 16.0 Hz, 1H), 4.79 – 4.70 (m, 1H), 4.18 (q, *J* = 7.2 Hz, 2H), 3.51 (t, *J* = 7.2 Hz, 2H), 3.31 – 3.18 (m, 2H), 1.67 – 1.54 (m, 2H), 1.33 – 1.19 (m, 19H), 0.86 (t, *J* = 7.2 Hz, 3H); ^{13}C NMR (100 MHz, $CDCl_3$) δ 172.00, 165.95, 144.83, 122.85, 60.65, 53.93, 44.14, 31.87, 31.11, 29.52 (d, *J* = 3.2 Hz), 29.39, 29.27, 29.15, 26.00 (d, *J* = 3.5 Hz), 22.65, 14.20, 14.08; Enantiomeric excess: 96%, determined by HPLC (Chiralpak IF hexane/*i*-PrOH = 85/15; flow rate 0.6 ml/min; 25 ℃; 254 nm), first peak: t_R = 19.1 min, second peak: t_R = 20.6 min. HRMS (ESI) calcd. for $C_{19}H_{33}NNaO_4S$ $[M+Na]^+$: 378.2073 found: 378.2065.

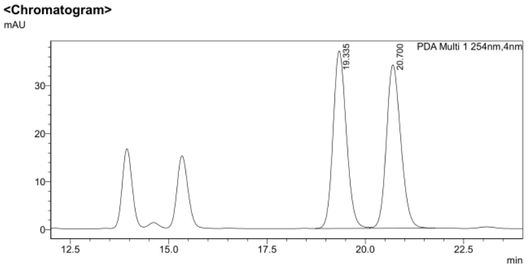

<Peak Table>
PDA Ch1 254nm

Peak#	Ret. Time	Area	Area%	Height	Height%
1	19.335	870335	50.004	36956	52.050
2	20.700	870186	49.996	34045	47.950
Total		1740522	100.000	71001	100.000

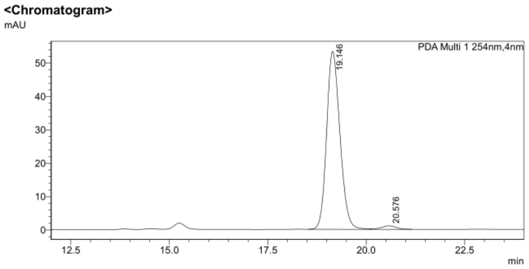

<Peak Table>
PDA Ch1 254nm

Peak#	Ret. Time	Area	Area%	Height	Height%
1	19.146	1252255	97.868	53339	98.088
2	20.576	27274	2.132	1040	1.912
Total		1279529	100.000	54378	100.000

4.26 Synthesis of ethyl (*R*, *E*)-5-methyl-4-(2-oxothiazolidin-3-yl)hex-2-enoate (3ge).

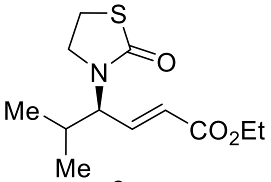

3ge

The general procedure was followed using **1g** (10.3 mg, 0.10 mmol) and **2e** (30.8 mg, 0.20 mmol). After purification by column chromatography (PE/EtOAc = 5:1), **3ge** (22.0 mg, 85%) was obtained as a colorless oil. $[\alpha]^{22}_{D}$ = -3.6 (*c* 0.25, acetone); ^{1}H NMR (400 MHz, $CDCl_3$) δ 6.84 (dd, *J* = 15.6, 7.6 Hz, 1H), 5.97 (dd, *J* = 15.6, 1.2 Hz, 1H), 4.37 – 4.28 (m, 1H), 4.20 (q, *J* = 7.2 Hz, 2H), 3.61 – 3.52 (m, 2H), 3.27 (t, *J* = 7.2 Hz, 2H), 1.98 – 1.86 (m, 1H), 1.29 (t, *J* = 7.2 Hz, 3H), 0.96 (dd, *J* = 6.4, 4.0 Hz, 6H); ^{13}C NMR (100 MHz, $CDCl_3$) δ 172.05, 166.01, 142.89, 124.71, 60.80, 60.70, 44.69, 29.64, 26.14, 19.83, 19.45, 14.21; Enantiomeric excess: 91%, determined by HPLC (Chiralpak IF hexane/*i*-PrOH = 85/15; flow rate 0.6 ml/min; 25 ℃; 254 nm), first peak: t_R = 19.3 min, second peak: t_R = 21. 3 min. HRMS (ESI) calcd. for $C_{12}H_{19}NNaO_3S$ $[M+Na]^+$: 280.0978, found: 280.0976.

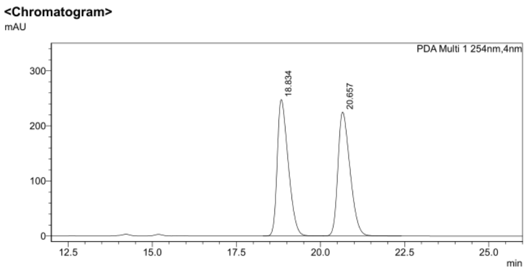

<Peak Table>
PDA Ch1 254nm

Peak#	Ret. Time	Area	Area%	Height	Height%
1	18.834	5644547	49.963	247900	52.392
2	20.657	5652889	50.037	225261	47.608
Total		11297436	100.000	473162	100.000

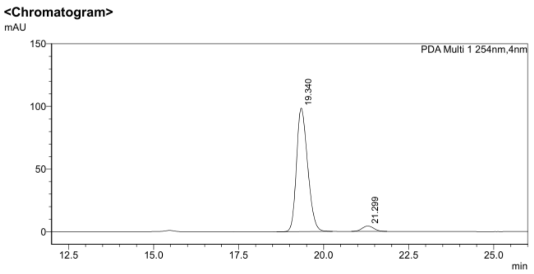

<Peak Table>
PDA Ch1 254nm

Peak#	Ret. Time	Area	Area%	Height	Height%
1	19.340	2250100	95.509	98634	95.779
2	21.299	105803	4.491	4347	4.221
Total		2355903	100.000	102981	100.000

4.27 Synthesis of ethyl (*R*, *E*)-4-(2-oxothiazolidin-3-yl)-6-phenylhex-2-enoate (3gf).

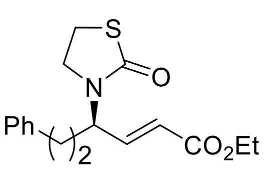

3gf

The general procedure was followed using **1g** (10.3 mg, 0.10 mmol) and **2f** (43.3 mg, 0.20 mmol). After purification by column chromatography (PE/EtOAc = 5:1), **3gf** (30.3 mg, 95%) was obtained as a colorless oil. $[\alpha]^{22}_{D}$ = -14.2 (*c* 0.25, acetone); ^{1}H NMR (400 MHz, $CDCl_3$) δ 7.32 – 7.25 (m, 2H), 7.23 – 7.14 (m, 3H), 6.83 (dd, *J* = 15.6, 5.6 Hz, 1H), 5.93 (d, *J* = 16.0 Hz, 1H), 4.83 (dd, *J* = 14.0, 6.8 Hz, 1H), 4.20 (q, *J* = 7.2 Hz, 2H), 3.57 – 3.45 (m, 2H), 3.31 – 3.11 (m, 2H), 2.74 – 2.57 (m, 2H), 2.00

(q, J = 7.6 Hz, 2H), 1.29 (t, J = 7.2 Hz, 3H); ^{13}C NMR (100 MHz, $CDCl_3$) δ 172.15, 165.85, 144.28, 140.54, 128.60, 128.31, 126.32, 123.30, 60.73, 53.88, 44.30, 32.80, 32.45, 25.94, 14.21; Enantiomeric excess: 95%, determined by HPLC (Chiralpak IF hexane/*i*-PrOH = 85/15; flow rate 0.6 ml/min; 25 ℃; 254 nm), first peak: t_R = 36.8 min, second peak: t_R = 42.4 min. HRMS (ESI) calcd. for $C_{17}H_{21}NNaO_3S$ $[M+Na]^+$: 342.1143, found: 342.1128.

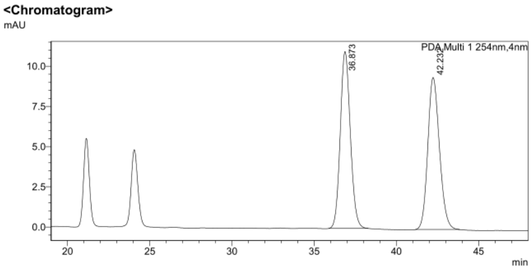

<Peak Table>
PDA Ch1 254nm

Peak#	Ret. Time	Area	Area%	Height	Height%
1	36.873	468598	50.124	11009	53.786
2	42.232	466281	49.876	9460	46.214
Total		934878	100.000	20469	100.000

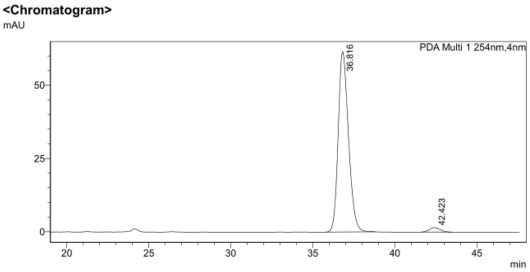

<Peak Table>
PDA Ch1 254nm

Peak#	Ret. Time	Area	Area%	Height	Height%
1	36.816	2679980	97.469	61509	97.624
2	42.423	69585	2.531	1497	2.376
Total		2749565	100.000	63006	100.000

4.28 Synthesis of 1-ethyl 8-methyl (*R*, *E*)-4-(2-oxothiazolidin-3-yl)oct-2-enedioate (3gg).

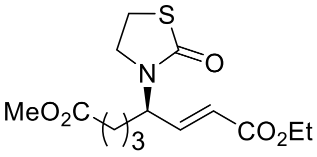

3gg

The general procedure was followed using **1g** (10.3 mg, 0.10 mmol) and **2g** (42.4 mg, 0.20 mmol). After purification by column chromatography (PE/EtOAc = 2:1), **3gg** (30.2 mg, 96%) was obtained as a colorless oil. $[\alpha]^{22}_D$ = 6.2 (c 0.10, acetone); ^{1}H NMR (400 MHz, $CDCl_3$) δ 6.78 (dd, J = 16.0, 5.6 Hz, 1H), 5.90 (dd, J = 15.6, 1.6 Hz, 1H), 4.80 – 4.72 (m, 1H), 4.18 (q, J = 6.8 Hz, 2H), 3.65 (s, 3H), 3.62 – 3.46 (m, 2H), 3.31 – 3.22 (m, 2H), 2.45 – 2.26 (m, 2H), 1.74 – 1.56 (m, 4H), 1.27 (t, J = 7.2 Hz, 3H); ^{13}C NMR (100 MHz, $CDCl_3$) δ 173.47, 172.26, 165.82, 144.30, 123.19, 60.72, 53.52, 51.63, 44.04, 33.00, 30.33, 26.01, 21.17, 14.20; Enantiomeric excess: 95%, determined by HPLC (Chiralpak IF hexane/*i*-PrOH = 92/08; flow rate 1.0 ml/min; 25 ℃; 190 nm), first peak: t_R = 83.6 min, second peak: t_R = 89.3 min. HRMS (ESI) calcd. for $C_{14}H_{21}NNaO_3S$ $[M+Na]^+$: 338.1033, found: 338.1027.

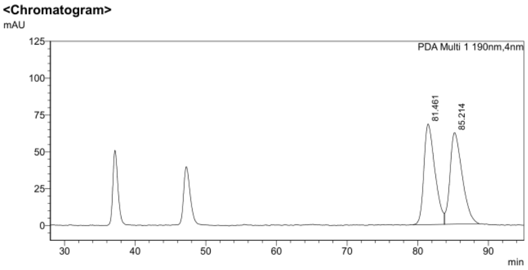

<Peak Table>
PDA Ch1 190nm

Peak#	Ret. Time	Area	Area%	Height	Height%
1	81.461	7423403	49.843	68325	52.389
2	85.214	7470076	50.157	62095	47.611
Total		14893480	100.000	130420	100.000

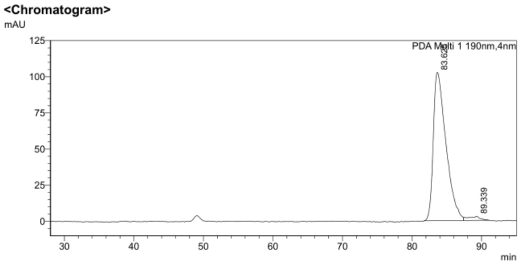

<Peak Table>
PDA Ch1 190nm

Peak#	Ret. Time	Area	Area%	Height	Height%
1	83.625	12938103	97.500	102486	97.283
2	89.339	331713	2.500	2862	2.717
Total		13269816	100.000	105348	100.000

4. General procedure for cascade reaction of other *N*-centered nucleophiles:

5.1 General procedure for variation of pyrrolidine-2,5-diones components

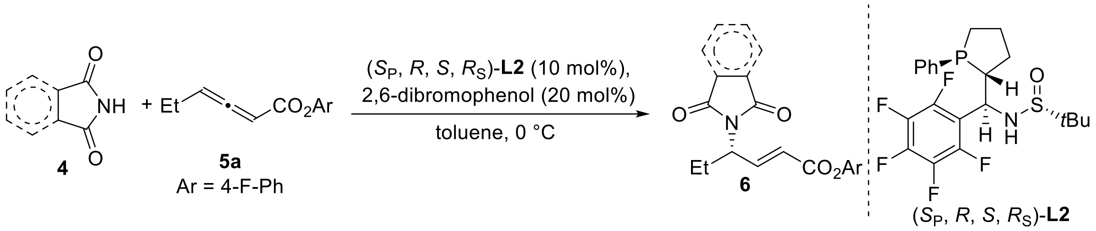

To a flame-dried glass tube with a magnetic stirring bar were added 3a,4,7,7a-tetrahydro-1H-isoindole-1,3(2H)-dione **4a** (14.7 mg, 0.10 mmol), 2,6-dibromophenol (5.1 mg, 0.02 mmol) and (S_P, *R*, *S*, R_S)-**L2** (4.6 mg, 0.01 mmol), followed by the addition of dry toluene (1.5 mL).[5] The above mixture was cooled to 0 °C, and then the allenoate **5a** (0.20 mmol) was slowly added via syringe at 0 °C under inert atmosphere. The reaction mixture was stirred at 0 °C for 12 h, and TLC show that the rection was completed. Then, the reaction system was warmed to room temperature, and toluene was removed under reduced pressure. The residue was purified by column chromatography on silica gel (petroleum ether/ethyl acetate = 6:1) to afford **6aa** (32.0 mg, 90% yield).

5.1.1 4-fluorophenyl (4*S*, *E*)-4-(1,3-dioxo-1,3,3a,4,7,7a-hexahydro-2H-isoindol-2-yl)hex-2-enoate (6aa).

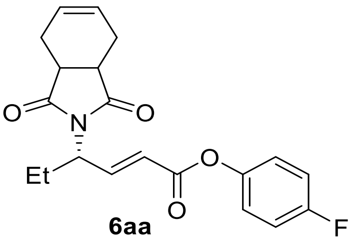

6aa

The general procedure was followed using **4a** (0.10 mmol) and **5a** (0.20 mmol). After purification by column chromatography (PE/EtOAc = 6:1), **6aa** (32.0 mg, 90%) was obtained as a colorless oil. $[\alpha]^{22}_{D}$ = -4.8 (*c* 0.25, acetone); ^{1}H NMR (400 MHz, $CDCl_3$) δ 7.21 (dd, *J* = 16.0, 6.4 Hz, 1H), 7.05 (d, *J* = 6.0 Hz, 4H), 5.99 (dd, *J* = 15.6, 1.6 Hz, 1H), 5.96 – 5.92 (m, 2H), 4.75 – 4.64 (m, 1H), 3.17 – 3.05 (m, 2H), 2.70 – 2.58 (m, 2H), 2.23 (dd, *J* = 14.8, 6.8 Hz, 2H), 2.17 – 2.02 (m, 1H), 1.95 – 1.82 (m, 1H), 0.86 (t, *J* = 7.6 Hz, 3H); ^{13}C NMR (100 MHz, $CDCl_3$) δ 179.61 (d, *J* = 6.5 Hz), 164.15, 161.38, 158.95, 146.31 (d, *J* = 2.8 Hz), 146.20, 127.97, 122.87 (d, *J* = 8.4 Hz), 121.76, 116.11, 115.88, 54.11, 38.95 (d, *J* = 12.3 Hz), 23.76, 23.60 (d, *J* = 2.5 Hz), 10.66; Enantiomeric excess: 92%, determined by HPLC (Chiralpak IC hexane/*i*-PrOH = 90/10; flow rate 0.8 ml/min; 25 ℃; 210 nm), first peak: t_R = 28.4 min, second peak: t_R = 38.6 min. HRMS (ESI) calcd. for $C_{20}H_{20}FNNaO_4$ $[M+Na]^+$: 380.1269, found: 380.1268.

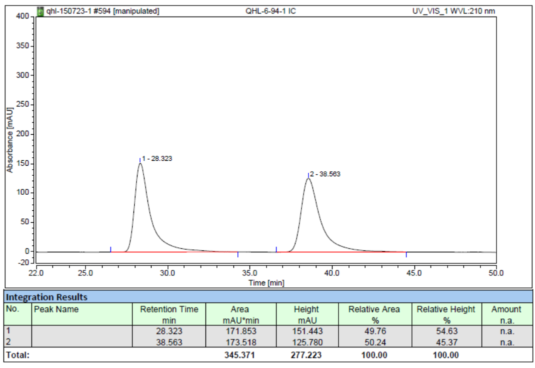

Integration Results

No.	Peak Name	Retention Time min	Area mAU*min	Height mAU	Relative Area %	Relative Height %	Amount n.a.
1		28.323	171.853	151.443	49.76	54.63	n.a.
2		38.563	173.518	125.780	50.24	45.37	n.a.
**Total:**			**345.371**	**277.223**	**100.00**	**100.00**	

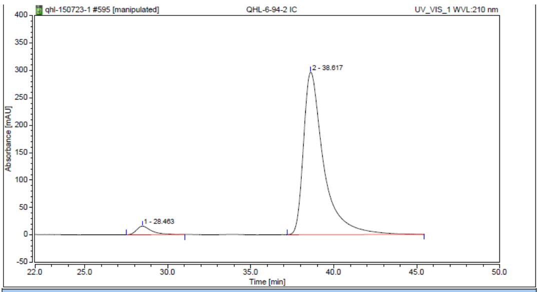

Integration Results

No.	Peak Name	Retention Time min	Area mAU*min	Height mAU	Relative Area %	Relative Height %	Amount n.a.
1		28.463	16.122	15.538	3.72	4.98	n.a.
2		38.617	417.778	296.450	96.28	95.02	n.a.
**Total:**			**433.899**	**311.989**	**100.00**	**100.00**	

5.1.2 4-fluorophenyl (4*S*, *E*)-4-(5,6-dimethyl-1,3-dioxo-1,3,3a,4,7,7a-hexahydro-2H-isoindol-2-yl)hex-2-enoate (6ba).

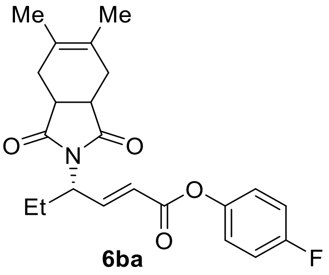

6ba

The general procedure was followed using **4b** (0.10 mmol) and **5a** (0.20 mmol). After purification by column chromatography (PE/EtOAc = 5:1), **6ba** (27.7 mg, 72%) was obtained. $[\alpha]^{22}_{D}$ = -9.2 (*c* 0.25, acetone); ^{1}H NMR (400 MHz, $CDCl_3$) δ 7.18 (dd, *J* = 16.0, 6.0 Hz, 1H), 7.05 (d, *J* = 6.4 Hz, 4H), 5.88 (dd, *J* = 15.6, 1.6 Hz, 1H), 4.73 – 4.61 (m, 1H), 3.12 – 3.00 (m, 2H), 2.49 (dd, *J* = 14.8, 5.2 Hz, 2H), 2.24 (d, *J* = 14.0 Hz, 2H), 2.18 – 2.02 (m, 1H), 1.93 – 1.81 (m, 1H), 1.69 (s, 6H), 0.84 (t, *J* = 7.6 Hz, 3H); ^{13}C NMR (100 MHz, $CDCl_3$) δ 179.80 (d, *J* = 9.9 Hz), 164.16, 161.41, 158.99, 146.48, 146.34 (d, *J* = 2.9 Hz), 127.44, 127.23, 122.86 (d, *J* = 8.5 Hz), 121.20, 116.16, 115.92, 54.02, 39.77 (d, *J* = 11.7 Hz), 30.86 (d, *J* = 5.9 Hz), 23.47, 19.22 (d, *J* = 3.2 Hz), 10.31; Enantiomeric excess: 88%, determined by HPLC (Chiralpak IC, hexane /*i*-PrOH = 90/10; flow rate 1.0 ml/min; 25 ℃; 254 nm), first peak: t_R = 13.4 min, second peak: t_R = 17.7 min. HRMS (ESI) calcd. for $C_{22}H_{24}FNNaO_4$ $[M+Na]^+$: 408.1582, found: 408.1581.

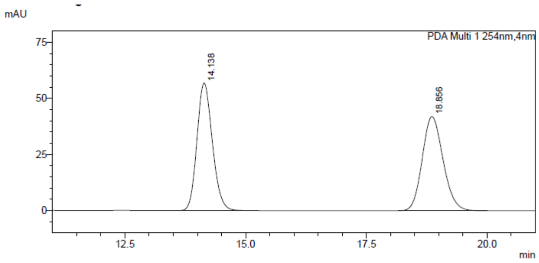

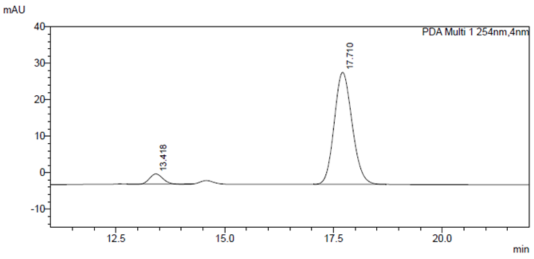

<Peak Table>

PDA Ch1 254nm

Peak#	Ret. Time	Area	Area%	Height	Height%
1	14.138	1260368	50.007	56798	57.506
2	18.856	1259995	49.993	41972	42.494
Total		2520363	100.000	98770	100.000

Peak#	Ret. Time	Area	Area%	Height	Height%
1	13.418	55849	5.974	2774	8.285
2	17.710	879037	94.026	30709	91.715
Total		934885	100.000	33483	100.000

5.1.3 4-fluorophenyl (4*S*, *E*)-4-(4,7-dimethyl-1,3-dioxo-1,3,3a,4,7,7a-hexahydro-2H-isoindol-2-yl)hex-2-enoate (6ca).

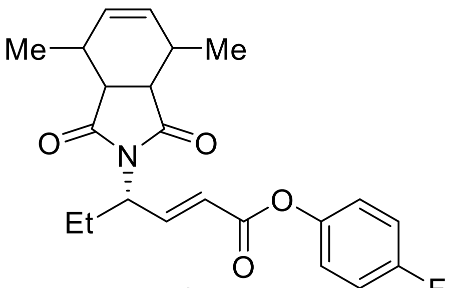

6ca

The general procedure was followed using **4c** (0.10 mmol) and **5a** (0.20 mmol). After purification by column chromatography (PE/EtOAc = 6:1), **6ca** (31.6 mg, 82%) was obtained. $[\alpha]^{22}_{D}$ = 2.8 (*c* 0.25, acetone); ^{1}H NMR (400 MHz, $CDCl_3$) δ 7.19 (dd, *J* = 16.0, 6.4 Hz, 1H), 7.08 – 7.01 (m, 4H), 5.98 (dd, *J* = 16.0, 1.6 Hz, 1H), 5.78 – 5.69 (m, 2H), 4.70 – 4.60 (m, 1H), 3.02 (p, *J* = 8.4 Hz, 2H), 2.50 – 2.39 (m, 2H), 2.13 – 2.01 (m, 1H), 1.92 – 1.80 (m, 1H), 1.46 (dd, *J* = 7.6, 2.0 Hz, 6H), 0.85 (t, *J* = 7.2 Hz, 3H); ^{13}C NMR (100 MHz, $CDCl_3$) δ 177.14 (d, *J* = 13.8 Hz), 164.23, 161.41, 158.98, 146.63, 146.36 (d, *J* = 2.8 Hz), 134.33, 122.91 (d, *J* = 8.4 Hz), 121.59, 116.13, 115.90, 53.63, 45.39 (d, *J* = 19.6 Hz), 31.03, 23.64, 16.71, 10.73; Enantiomeric excess: 90%, determined by HPLC (Chiralpak IC, hexane/*i*-PrOH = 80/20; flow rate 0.8 ml/min; 25 ℃; 254 nm), first peak: t_R = 12.7 min, second peak: t_R = 13.8 min. HRMS (ESI) calcd. for $C_{22}H_{24}FNNaO_4$ $[M+Na]^+$: 408.1582, found: 408.1584.

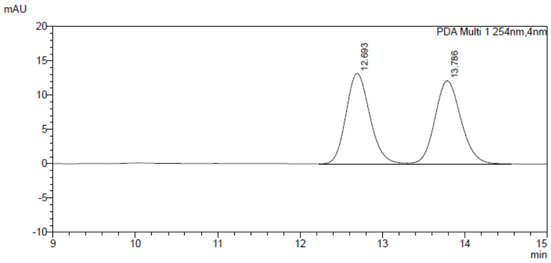

<Peak Table>
PDA Ch1 254nm

Peak#	Ret. Time	Area	Area%	Height	Height%
1	12.693	265418	49.948	13213	52.156
2	13.786	265971	50.052	12121	47.844
Total		531390	100.000	25333	100.000

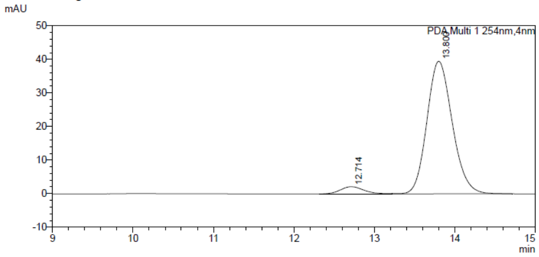

<Peak Table>
PDA Ch1 254nm

Peak#	Ret. Time	Area	Area%	Height	Height%
1	12.714	43225	4.749	2147	5.156
2	13.800	866947	95.251	39493	94.844
Total		910172	100.000	41640	100.000

5.1.4 4-fluorophenyl (4*S*, *E*)-4-(1,3-dioxooctahydro-2H-isoindol-2-yl)hex-2-enoate (6da).

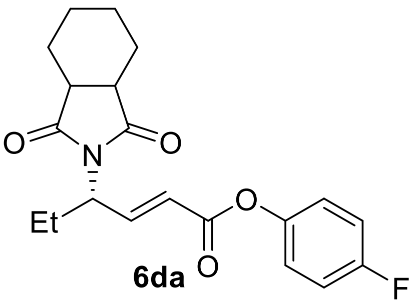

6da

The general procedure was followed using **4d** (0.10 mmol) and **5a** (0.20 mmol). After purification by column chromatography (PE/EtOAc = 6:1), **6da** (30.2 mg, 84%) was obtained. $[\alpha]^{22}_{D}$ = 14.8 (*c* 0.25, acetone); ^{1}H NMR (400 MHz, $CDCl_3$) δ 7.27 (dd, *J* = 14.0, 6.8 Hz, 1H), 7.13 – 6.99 (m, 4H), 6.06 (d, *J* = 16.0 Hz, 1H), 4.70 (dd, *J* = 15.2, 7.2 Hz, 1H), 2.96 – 2.82 (m, 2H), 2.18 – 2.06 (m, 1H), 2.03 – 1.93 (m, 1H), 1.93 – 1.83 (m, 2H), 1.83 – 1.69 (m, 2H), 1.55 – 1.38 (m, 4H), 0.93 (t, *J* = 7.2 Hz, 3H); ^{13}C NMR (100 MHz, $CDCl_3$) δ 179.16 (d, *J* = 4.1 Hz), 164.16, 161.42, 159.00, 146.35, 146.31, 122.90 (d, *J* = 8.5 Hz), 122.11, 116.15, 115.92, 53.54, 39.70, 23.99, 23.88, 21.74 (d, *J* = 0.8 Hz), 10.83; Enantiomeric excess: 91%, determined by HPLC (Chiralpak IC, hexane/*i*-PrOH = 90/10; flow rate 0.8 ml/min; 25 ℃; 210 nm), first peak: t_R = 24.2 min, second peak: t_R = 35.4 min. HRMS (ESI) calcd. for $C_{20}H_{22}FNNaO_4$ $[M+Na]^+$: 382.1425, found: 382.1433.

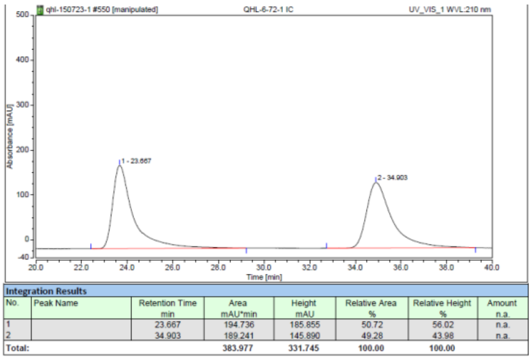

Integration Results

No.	Peak Name	Retention Time min	Area mAU*min	Height mAU	Relative Area %	Relative Height %	Amount n.a.
1		23.667	194.736	185.855	50.72	56.02	n.a.
2		34.903	189.241	145.890	49.28	43.98	n.a.
Total:			383.977	331.745	100.00	100.00	

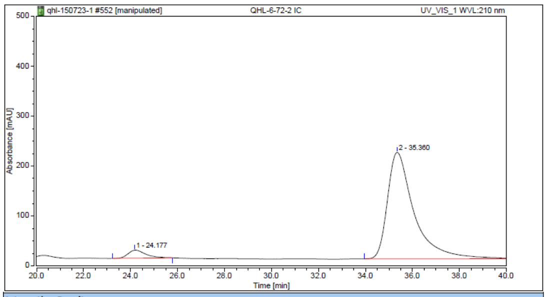

Integration Results

No.	Peak Name	Retention Time min	Area mAU*min	Height mAU	Relative Area %	Relative Height %	Amount n.a.
1		24.177	13.878	16.065	4.71	7.00	n.a.
2		35.360	280.853	213.442	95.29	93.00	n.a.
Total:			294.731	229.508	100.00	100.00	

5.1.5 4-Fluorophenyl (4*S*, *E*)-4-(1,3-dioxohexahydrocyclopenta[c]pyrrol-2(1H)-yl) hex- 2-enoate (6ea).

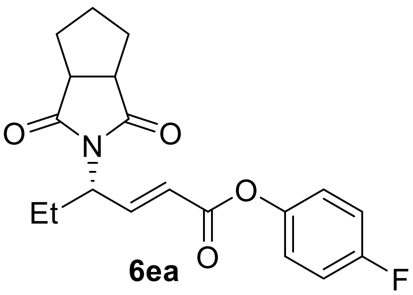

The general procedure was followed using **4e** (0.10 mmol) and **5a** (0.20 mmol). After purification by column chromatography (PE/EtOAc = 6:1), **6ea** (29.5 mg, 85%) was obtained. $[\alpha]^{22}_{D}$ = 12.4 (*c* 0.25, acetone); ^{1}H NMR (400 MHz, $CDCl_3$) δ 7.25 (dd, *J* = 16.0, 6.8 Hz, 1H), 7.10 – 7.02 (m, 4H), 6.03 (dd, *J* = 16.0, 1.2 Hz, 1H), 4.69 (q, *J* = 7.2 Hz, 1H), 3.17 (p, *J* = 8.7 Hz, 2H), 2.19 – 2.14 (m, 2H), 2.12 – 2.04 (m, 1H), 1.99 – 1.85 (m, 3H), 1.81 (dt, *J* = 19.2, 6.8 Hz, 1H), 1.38 – 1.28 (m, 1H), 0.88 (t, J = 7.2 Hz, 3H); ^{13}C NMR (100 MHz, $CDCl_3$) δ 177.17 (d, *J* = 9.3 Hz), 164.18, 161.38, 158.96, 146.31, 146.28, 134.72 (d, *J* = 7.0 Hz), 122.88 (d, *J* = 8.5 Hz), 122.08, 116.13, 115.90, 53.77, 52.28, 45.57 (d, *J* = 11.3 Hz), 45.04 (d, *J* = 2.1 Hz), 23.82, 10.86; Enantiomeric excess: 90%, determined by HPLC (Chiralpak IC, hexane/*i*-PrOH = 90/10; flow rate 0.8 ml/min; 25 ℃; 210 nm), first peak: t_R = 31.8 min, second peak: t_R = 48.8 min. HRMS (ESI) calcd. for $C_{19}H_{20}FNNaO_4$ $[M+Na]^+$: 368.1269, found: 368.1266.

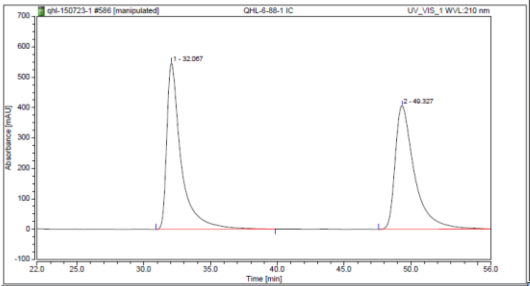

Integration Results

No.	Peak Name	Retention Time min	Area mAU*min	Height mAU	Relative Area %	Relative Height %	Amount n.a.
1		32.067	676.909	546.495	49.99	57.34	n.a.
2		49.327	677.089	406.620	50.01	42.66	n.a.
**Total:**			**1353.998**	**953.114**	**100.00**	**100.00**	

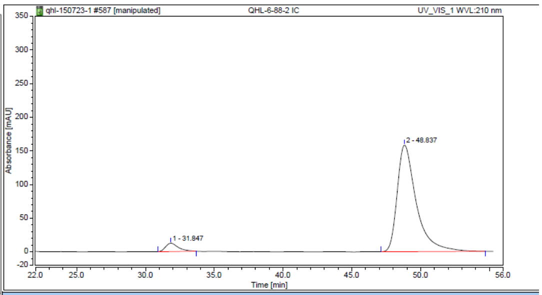

Integration Results

No.	Peak Name	Retention Time min	Area mAU*min	Height mAU	Relative Area %	Relative Height %	Amount n.a.
1		31.847	13.264	12.409	4.95	7.26	n.a.
2		48.837	254.722	158.559	95.05	92.74	n.a.
**Total:**			**267.986**	**170.968**	**100.00**	**100.00**	

5.1.6 4-fluorophenyl (4*S*, *E*)-4-((3a*R*,7a*S*)-1,3-dioxooctahydro-2H-4,7-methanoiso- indol-2-yl)hex-2-enoate (6fa).

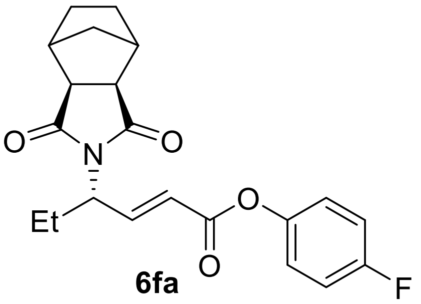

6fa

The general procedure was followed using **4f** (0.10 mmol) and **5a** (0.20 mmol). After purification by column chromatography (PE/EtOAc = 4:1), **6fa** (34.0 mg, 91%) was obtained. $[\alpha]^{22}_{D}$ = 20.0 (*c* 0.25, acetone); ^{1}H NMR (400 MHz, $CDCl_3$) δ 7.25 (dd, *J* = 16.0, 7.6 Hz, 1H), 7.09 – 6.99 (m, 4H), 6.06 (dd, *J* = 15.6, 1.2 Hz, 1H), 4.66 (q, *J* = 7.2 Hz, 1H), 2.72 (s, 2H), 2.64 – 2.58 (m, 2H), 2.00 (pd, *J* = 14.2, 7.4 Hz, 2H), 1.67 (d, *J* = 8.0 Hz, 2H), 1.34 (dd, *J* = 9.6, 2.0 Hz, 2H), 1.25 (dd, *J* = 12.8, 3.2 Hz, 1H), 1.15 (d, *J* = 11.2 Hz, 1H), 0.90 (t, *J* = 7.6 Hz, 3H); ^{13}C NMR (100 MHz, $CDCl_3$) δ 13C NMR (101 MHz, CDCl3) δ 178.38 (d, *J* = 3.0 Hz), 164.11, 161.38, 158.95, 146.27 (d, *J* = 2.9 Hz), 146.02, 122.87 (d, *J* = 8.4 Hz), 122.45, 116.12, 115.89, 54.03, 48.38 (d, *J* = 5.0 Hz), 39.80, 33.27, 27.96 (d, *J* = 6.2 Hz), 23.91, 10.92; Enantiomeric excess: 90%, determined by HPLC (Chiralpak IC, hexane/*i*-PrOH = 90/10; flow rate 0.8 ml/min; 25 ℃; 210 nm), first peak: t_R = 27.9 min, second peak: t_R = 36.0 min. HRMS (ESI) calcd. for $C_{21}H_{22}FNNaO_4$ $[M+Na]^+$: 394.1425, found: 394.1424.

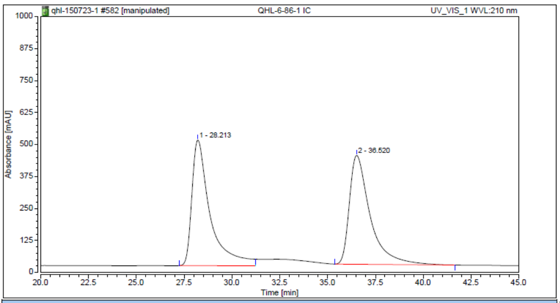

Integration Results

No.	Peak Name	Retention Time min	Area mAU*min	Height mAU	Relative Area %	Relative Height %	Amount n.a.
1		28.213	545.295	491.357	50.42	53.57	n.a.
2		36.520	536.215	425.932	49.58	46.43	n.a.
**Total:**			**1081.510**	**917.290**	**100.00**	**100.00**	

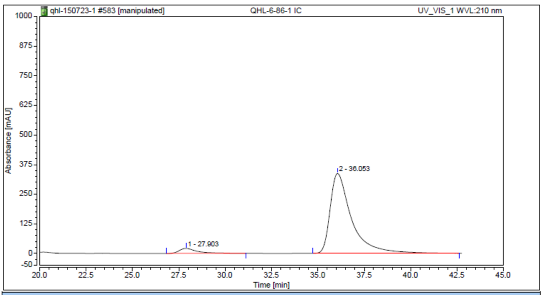

Integration Results

No.	Peak Name	Retention Time min	Area mAU*min	Height mAU	Relative Area %	Relative Height %	Amount n.a.
1		27.903	23.485	21.168	4.96	5.91	n.a.
2		36.053	449.782	337.293	95.04	94.09	n.a.
**Total:**			**473.268**	**358.461**	**100.00**	**100.00**	

5.1.7 4-fluorophenyl (4*S*, *E*)-4-((3a*R*,7a*S*)-1,3-dioxo-1,3,3a,4,7,7a-hexahydro-2H-4,7- methanoisoindol-2-yl)hex-2-enoate (6ga).

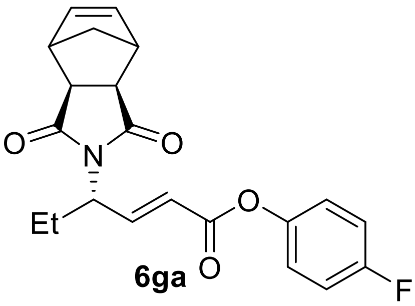

6ga

The general procedure was followed using **4g** (0.10 mmol) and **5a** (0.20 mmol). After purification by column chromatography (PE/EtOAc = 4:1), **6ga** (28.4 mg, 77%) was obtained. $[\alpha]^{22}_{D}$ = 20.0 (*c* 0.25, acetone); ^{1}H NMR (400 MHz, $CDCl_3$) δ 7.16 (dd, *J* = 16.0, 6.8 Hz, 1H), 7.11 – 7.00 (m, 4H), 6.15 (s, 2H), 6.01 (dd, *J* = 16.0, 1.2 Hz, 1H), 4.54 (q, *J* = 7.2 Hz, 1H), 3.41 (s, 2H), 3.35 – 3.23 (m, 2H), 2.03 – 1.80 (m, 2H), 1.74 (d, *J* = 8.8 Hz, 1H), 1.54 (d, *J* = 8.8 Hz, 1H), 0.87 (t, *J* = 7.6 Hz, 3H); ^{13}C NMR (100 MHz, $CDCl_3$) δ 179.83, 164.14, 161.39, 158.97, 146.28 (d, *J* = 2.9 Hz), 146.18, 122.88 (d, *J* = 8.5 Hz), 122.11, 116.14, 115.90, 54.02, 44.95 (d, *J* = 15.3 Hz), 30.58 (d, *J* = 2.1 Hz), 24.80, 23.80, 10.74; Enantiomeric excess: 91%, determined by HPLC (Chiralpak OD-H, hexane/*i*-PrOH = 90/10; flow rate 0.8 ml/min; 25 ℃; 210 nm), first peak: t_R = 21.7 min, second peak: t_R = 28.3 min. HRMS (ESI) calcd. for $C_{21}H_{20}FNNaO_4$ $[M+Na]^+$: 392.1269, found: 392.1267.

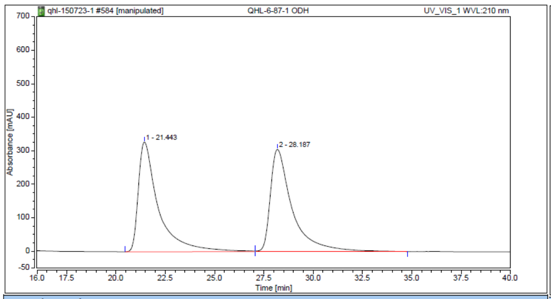

Integration Results

No.	Peak Name	Retention Time min	Area mAU*min	Height mAU	Relative Area %	Relative Height %	Amount n.a.
1		21.443	389.125	327.623	49.76	51.76	n.a.
2		28.187	392.901	305.402	50.24	48.24	n.a.
Total:			782.026	633.025	100.00	100.00	

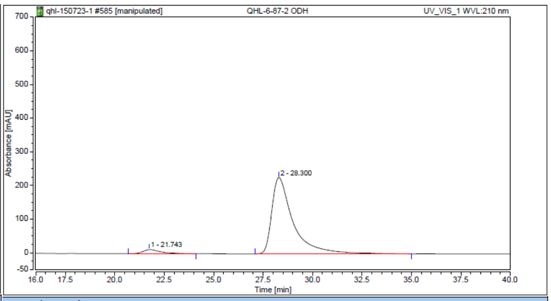

Integration Results

No.	Peak Name	Retention Time min	Area mAU*min	Height mAU	Relative Area %	Relative Height %	Amount n.a.
1		21.743	12.678	12.224	4.28	5.12	n.a.
2		28.300	283.454	226.752	95.72	94.88	n.a.
Total:			296.132	238.976	100.00	100.00	

5.1.8 4-fluorophenyl (4*S*, *E*)-4-((3a*R*,7a*S*)-1,3-dioxo-1,3,3a,4,7,7a-hexahydro-2H-4,7- epoxyisoindol-2-yl)hex-2-enoate (6ha).

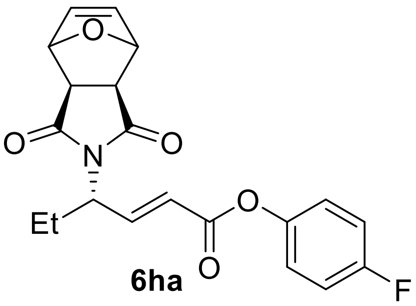

6ha

The general procedure was followed using **4h** (0.10 mmol) and **5a** (0.20 mmol). After purification by column chromatography (PE/EtOAc = 3:1), **6ha** (26.4 mg, 78%) was obtained. $[\alpha]^{22}_{D}$ = 4.0 (*c* 0.25, acetone); ^{1}H NMR (400 MHz, $CDCl_3$) δ 7.23 (dd, *J* = 16.0, 6.4 Hz, 1H), 7.11 – 7.00 (m, 4H), 6.53 (s, 2H), 6.06 (dd, *J* = 16.0, 1.2 Hz, 1H), 5.31 (s, 2H), 4.74 – 4.65 (m, 1H), 2.87 (q, *J* = 6.4 Hz, 2H), 2.20 – 2.06 (m, 1H), 1.98 – 1.85 (m, 1H), 0.92 (t, *J* = 7.6 Hz, 3H); ^{13}C NMR (100 MHz, $CDCl_3$) δ 175.80 (d, *J* = 4.2 Hz), 164.22, 161.43, 159.00, 146.35 (d, *J* = 2.9 Hz), 145.96, 136.54 (d, *J* = 4.2 Hz), 122.92 (d, *J* = 8.4 Hz), 121.90, 116.15, 115.91, 81.09 (d, *J* = 2.0 Hz), 54.23, 47.23 (d, *J* = 16.0 Hz), 23.92, 10.60; Enantiomeric excess: 95%, determined by HPLC (Chiralpak IC, hexane/*i*-PrOH = 70/30; flow rate 0.8 ml/min; 25 ℃; 254 nm), first peak: t_R = 12.2 min, second peak: t_R = 13.8 min. HRMS (ESI) calcd. for $C_{20}H_{18}FNNaO_5$ $[M+Na]^+$: 394.1061, found: 394.1067.

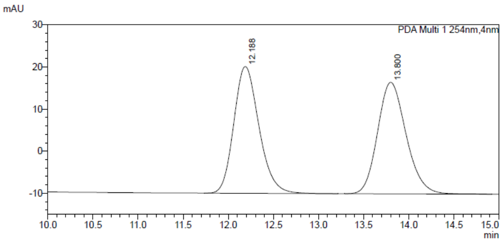

<Peak Table>

PDA Ch1 254nm

Peak#	Ret. Time	Area	Area%	Height	Height%
1	12.188	585218	50.100	30113	53.214
2	13.800	582877	49.900	26475	46.786
Total		1168095	100.000	56588	100.000

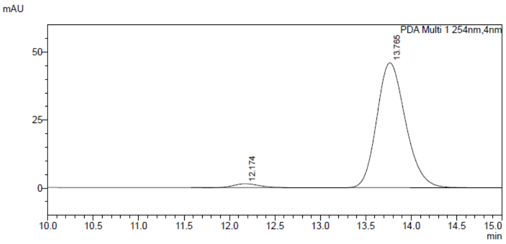

<Peak Table>

PDA Ch1 254nm

Peak#	Ret. Time	Area	Height	Area%	Height%
1	12.174	27801	1428	2.682	3.020
2	13.765	1008832	45869	97.318	96.980
Total		1036632	47297	100.000	100.000

5.1.9 4-fluorophenyl (4*S*, *E*)-4-((3a*R*,7a*S*)-1,3-dioxo-1,3,3a,4,7,7a-hexahydro-2H -4,7- ethanoisoindol-2-yl)hex-2-enoate (6ia).

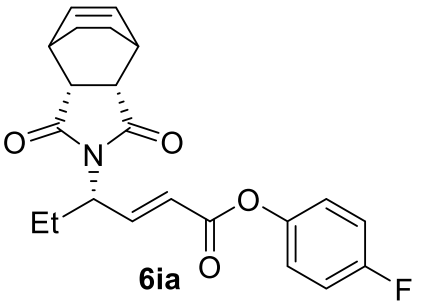

6ia

The general procedure was followed using **4i** (0.10 mmol) and **5a** (0.20 mmol). After purification by column chromatography (PE/EtOAc = 7:1), **6ia** (28.4 mg, 73%) was obtained. $[\alpha]^{22}_{D}$ = 3.6 (*c* 0.25, acetone); ^{1}H NMR (400 MHz, $CDCl_3$) δ 7.18 (dd, *J* = 16.0, 6.4 Hz, 1H), 7.09 – 7.01 (m, 4H), 6.26 – 6.18 (m, 2H), 6.00 (dd, *J* = 16.0, 1.2 Hz, 1H), 4.62 (dd, *J* = 14.4, 6.4 Hz, 1H), 3.17 (s, 2H), 2.90 – 2.81 (m, 2H), 2.12 – 1.97 (m, 1H), 1.94 – 1.81 (m, 1H), 1.61 (d, *J* = 7.2 Hz, 2H), 1.40 (d, *J* = 7.6 Hz, 2H), 0.87 (t, *J* = 7.6 Hz, 3H); ^{13}C NMR (100 MHz, $CDCl_3$) δ 178.40 (d, *J* = 11.7 Hz), 164.20, 146.34, 132.59 (d, *J* = 2.1 Hz), 122.89 (d, *J* = 8.4 Hz), 121.81, 116.13, 115.90, 53.78, 43.99 (d, *J* = 11.0 Hz), 31.70, 23.81, 23.63 (d, *J* = 5.1 Hz), 10.81; Enantiomeric excess: 92%, determined by HPLC (Chiralpak IC, hexane/*i*-PrOH = 90/10; flow rate 1.0 ml/min; 25 ℃; 254 nm), first peak: t_R = 18.1 min, second peak: t_R = 19.4 min. HRMS (ESI) calcd. For $C_{22}H_{22}FNNaO_4$ $[M+Na]^+$: 406.1425, found: 406.1427.

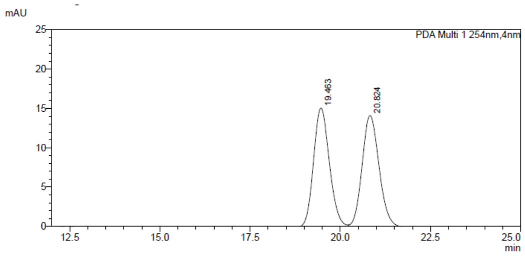

<Peak Table>

PDA Ch1 254nm

Peak#	Ret. Time	Area	Area%	Height	Height%
1	19.463	464157	49.909	15136	51.605
2	20.824	465854	50.091	14194	48.395
Total		930011	100.000	29330	100.000

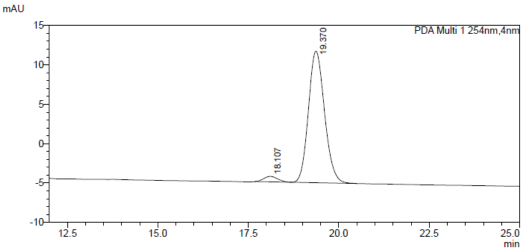

<Peak Table>

PDA Ch1 254nm

Peak#	Ret. Time	Area	Area%	Height	Height%
1	18.107	18757	3.501	698	4.010
2	19.370	516980	96.499	16698	95.990
Total		535737	100.000	17396	100.000

5.1.10 4-fluorophenyl (4*S*, *E*)-4-((3a*R*,6a*S*)-1,3-dioxo-3,3a,4,4a,5,5a,6,6a-octahydro-4,6- ethenocyclopropa[f]isoindol-2(1H)-yl)hex-2-enoate (6ja).

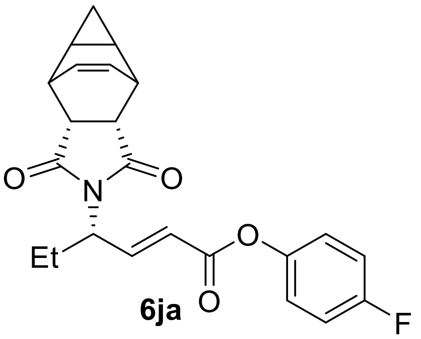

6ja

The general procedure was followed using **4j** (0.10 mmol) and **5a** (0.20 mmol). After purification by column chromatography (PE/EtOAc = 5:1), **6ja** (26.8 mg, 68%) was obtained. $[\alpha]^{22}_{D}$ = 2.8 (*c* 0.25, acetone); ^{1}H NMR (400 MHz, $CDCl_3$) δ 7.17 (dd, *J* = 16.0, 6.4 Hz, 1H), 7.06 (d, *J* = 6.4 Hz, 4H), 6.00 (dd, *J* = 15.6, 1.2 Hz, 1H), 5.83 – 5.76 (m, 2H), 4.65 – 4.58 (m, 1H), 3.41 (d, *J* = 2.0 Hz, 2H), 3.03 – 2.96 (m, 2H), 2.10 – 1.97 (m, 1H),1.94 – 1.82 (m, 1H), 1.14 – 1.08 (m, 2H), 0.87 (t, *J* = 7.6Hz, 3H), 0.33 – 0.27 (m, 1H), 0.26 – 0.20 (m, 1H); ^{13}C NMR (100 MHz, $CDCl_3$) δ 178.04 (d, *J* = 10.1 Hz), 164.22, 161.42, 146.38, 146.33 (d, *J* = 2.9 Hz), 127.95 (d, *J* = 3.8 Hz), 122.91 (d, *J* = 8.5 Hz), 121.82, 116.16, 115.92, 53.68, 45.09 (d, *J* = 10.8 Hz), 33.48, 23.85, 10.84, 9.80 (d, *J* = 5.4 Hz), 4.73; Enantiomeric excess: 94%, determined by HPLC (Chiralpak IC, hexane/*i*-PrOH = 80/20; flow rate 0.8 ml/min; 25 ℃; 210 nm), first peak: t_R = 19.1 min, second peak: t_R = 24.8 min. HRMS (ESI) calcd. For $C_{23}H_{22}FNNaO_4$ $[M+Na]^+$: 418.1425, found: 418.1425.

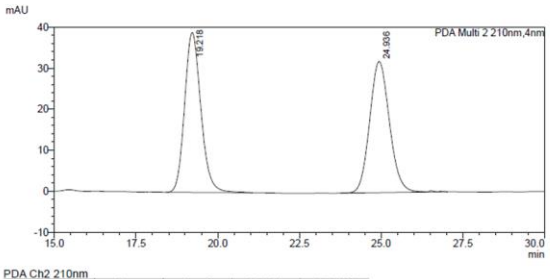

PDA Ch2 210nm

Peak#	Ret. Time	Area	Area%	Height	Height%
1	19.218	1350000	50.028	38942	54.944
2	24.936	1348490	49.972	31934	45.056
Total		2698490	100.000	70876	100.000

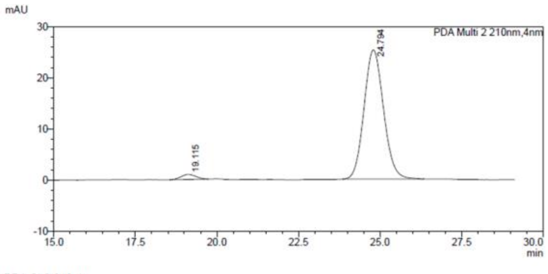

PDA Ch2 210nm

Peak#	Ret. Time	Area	Area%	Height	Height%
1	19.115	30873	2.854	989	3.767
2	24.794	1051007	97.146	25257	96.233
Total		1081879	100.000	26245	100.000

5.1.11 4-fluorophenyl (4*S*, *E*)-4-(2,5-dioxopyrrolidin-1-yl)hex-2-enoate (6ka).

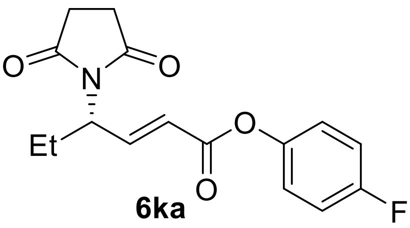

6ka

The general procedure was followed using **4k** (0.10 mmol) and **5a** (0.20 mmol). After purification by column chromatography (PE/EtOAc = 3:1), **6ka** (24.8 mg, 81%) was obtained. $[\alpha]^{22}_D$ = 10.0 (*c* 0.25, acetone); ^{1}H NMR (400 MHz, $CDCl_3$) δ 7.29 (dd, *J* = 15.6, 6.8 Hz, 1H), 7.11 – 7.02 (m, 4H), 6.07 (d, *J* = 16.0 Hz, 1H), 4.74 (dd, *J* = 15.2, 8.0 Hz, 1H), 2.75 (s, 4H), 2.17 – 1.93 (m, 2H), 0.92 (t, *J* = 7.6 Hz, 3H); ^{13}C NMR (100 MHz, $CDCl_3$) δ 176.58, 164.13, 161.46, 159.03, 146.32 (d, *J* = 2.9 Hz), 145.92, 122.89 (d, *J* = 8.4 Hz), 122.50, 116.19, 115.96, 54.08, 28.04, 23.82, 10.78; Enantiomeric excess: 87%, determined by HPLC (Chiralpak IC, hexane/*i*-PrOH = 70/30; flow rate 0.8 ml/min; 25 ℃; 254 nm), first peak: t_R = 15.1 min, second peak: t_R = 18.9 min. HRMS (ESI) calcd. For $C_{16}H_{16}FNNaO_4$ $[M+Na]^+$: 328.0956, found: 328.0951.

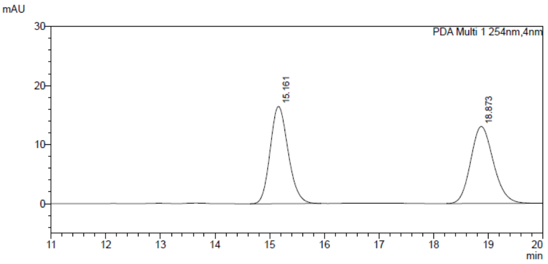

<Peak Table>

PDA Ch1 254nm

Peak#	Ret. Time	Area	Area%	Height	Height%
1	15.161	377075	49.965	16472	55.818
2	18.873	377600	50.035	13039	44.182
Total		754676	100.000	29511	100.000

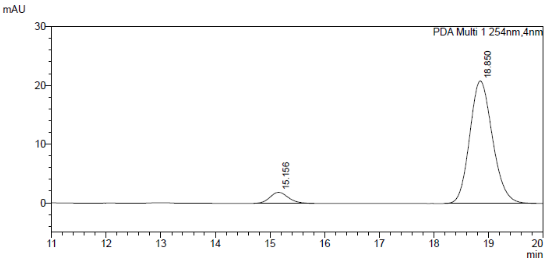

<Peak Table>

PDA Ch1 254nm

Peak#	Ret. Time	Area	Area%	Height	Height%
1	15.156	43282	6.684	1874	8.238
2	18.850	604254	93.316	20868	91.762
Total		647535	100.000	22742	100.000

5.1.12 4-fluorophenyl (4*S*, *E*)-4-(1,3-dioxoisoindolin-2-yl)hex-2-enoate (6la).

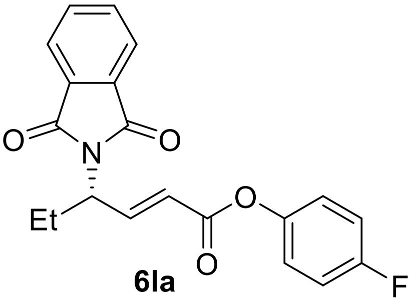

6la

The general procedure was followed using **4l** (0.10 mmol) and **5a** (0.25 mmol). After purification by column chromatography (PE/EtOAc = 6:1), **6la** (30.0 mg, 81%) was obtained. $[\alpha]^{22}_{D}$ = 3.5 (*c* 0.25, acetone); ^{1}H NMR (400 MHz, $CDCl_3$) δ 7.90 – 7.83 (m, 2H), 7.79 – 7.72 (m, 2H), 7.37 (dd, *J* = 15.6, 6.4 Hz, 1H), 7.11 – 6.99 (m, 4H), 6.11 (dd, *J* = 16.0, 1.6 Hz, 1H), 4.89 (dd, *J* = 14.8, 6.8 Hz, 1H), 2.31 – 2.17 (m, 1H), 2.12 – 2.00 (m, 1H), 0.97 (t, *J* = 7.2 Hz, 3H); ^{13}C NMR (100 MHz, $CDCl_3$) δ 167.79, 164.20, 161.41, 158.99, 146.77, 146.32 (d, *J* = 2.8 Hz), 134.24, 131.63, 123.45, 122.88 (d, *J* = 8.5 Hz), 121.95, 116.15, 115.91, 53.37, 24.64, 10.88; Enantiomeric excess: 90%, determined by HPLC (Chiralpak IC, hexane/*i*-PrOH = 90/10; flow rate 0.8 ml/min; 25 ℃; 210 nm), first peak: t_R = 15.2 min, second peak: t_R = 24.2 min. HRMS (ESI) calcd. For $C_{20}H_{16}FNNaO_4$ $[M+Na]^+$: 376.0956, found: 376.0954.

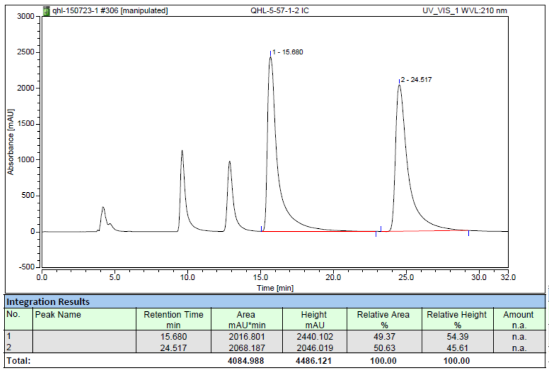

Integration Results

No.	Peak Name	Retention Time min	Area mAU*min	Height mAU	Relative Area %	Relative Height %	Amount n.a.
1		15.680	2016.801	2440.102	49.37	54.39	n.a.
2		24.517	2068.187	2046.019	50.63	45.61	n.a.
**Total:**			**4084.988**	**4486.121**	**100.00**	**100.00**	

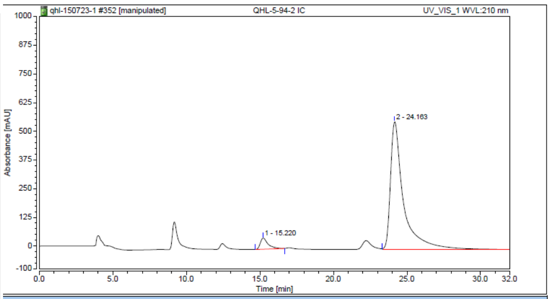

Integration Results

No.	Peak Name	Retention Time min	Area mAU*min	Height mAU	Relative Area %	Relative Height %	Amount n.a.
1		15.220	29.796	48.784	4.92	8.05	n.a.
2		24.163	576.102	557.000	95.08	91.95	n.a.
**Total:**			**605.898**	**605.784**	**100.00**	**100.00**	

5.1.13 4-fluorophenyl (4*S*, *E*)-4-(1,3-dioxo-1,3,3a,4,7,7a-hexahydro-2H-isoindol-2-yl)pent-2-enoate (6ab).

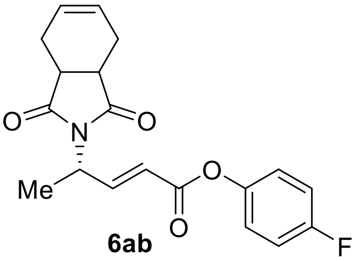

6ab

The general procedure was followed using **4a** (0.1 mmol) and **5b** (0.2 mmol). After purification by column chromatography (PE/EtOAc = 4:1), **6ab** (24.0 mg, 70%) was obtained. $[\alpha]^{22}_{D}$ = 18.8 (*c* 0.25, acetone); ^{1}H NMR (400 MHz, $CDCl_3$) δ 7.16 (dd, *J* = 16.0, 5.2 Hz, 1H), 7.05 (d, *J* = 6.4 Hz, 4H), 5.98 (dd, *J* = 16.0, 1.6 Hz, 1H), 5.95 – 5.90 (m, 2H), 5.00 – 4.89 (m, 1H), 3.15 – 3.02 (m, 2H), 2.64 (d, *J* = 16.4 Hz, 2H),

2.31 – 2.15 (m, 2H), 1.53 (d, *J* = 7.2 Hz, 3H); ^{13}C NMR (100 MHz, $CDCl_3$) δ 179.35 (d, *J* = 7.2 Hz), 164.19, 161.39, 158.97, 147.39, 146.30 (d, *J* = 2.8 Hz), 127.86, 122.90 (d, *J* = 8.5 Hz), 121.10, 116.14, 115.91, 47.61, 39.00, 23.59 (d, *J* = 3.4 Hz), 16.78; Enantiomeric excess: 93%, determined by HPLC (Chiralpak IC, hexane/ *i*-PrOH = 90/10; flow rate 0.8 ml/min; 25 ℃; 210 nm), first peak: t_R = 32.6 min, second peak: t_R = 41.9 min. HRMS (ESI) calcd. For $C_{19}H_{18}FNNaO_4$ $[M+Na]^+$: 366.1112, found: 366.1107.

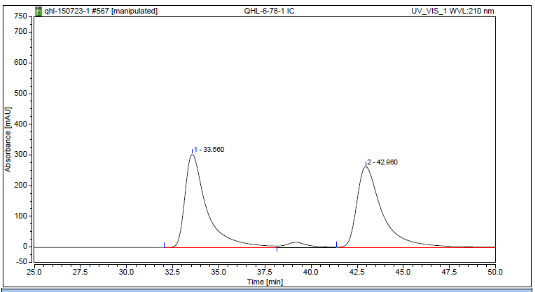

Integration Results

No.	Peak Name	Retention Time min	Area mAU*min	Height mAU	Relative Area %	Relative Height %	Amount n.a.
1		33.560	416.532	303.689	49.64	53.52	n.a.
2		42.960	422.573	263.737	50.36	46.48	n.a.
Total:			839.104	567.425	100.00	100.00	

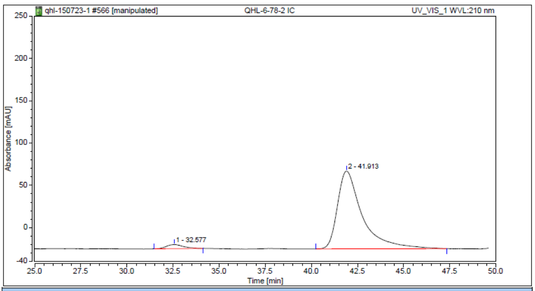

Integration Results

No.	Peak Name	Retention Time min	Area mAU*min	Height mAU	Relative Area %	Relative Height %	Amount n.a.
1		32.577	5.045	4.845	3.38	5.00	n.a.
2		41.913	144.116	91.971	96.62	95.00	n.a.
Total:			149.161	96.816	100.00	100.00	

5.1.14 4-fluorophenyl (4*S*, *E*)-4-(1,3-dioxo-1,3,3a,4,7,7a-hexahydro-2H-isoindol-2-yl)hept-2-enoate (6ac).

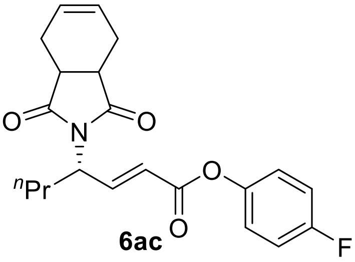

The general procedure was followed using **4a** (0.1 mmol) and **5c** (0.2 mmol). After purification by column chromatography (PE/EtOAc = 4:1), **6ac** (33.0 mg, 89%) was obtained. $[\alpha]^{22}_D$ = 6.4 (*c* 0.25, acetone); ^{1}H NMR (400 MHz, $CDCl_3$) δ 7.21 (dd, *J* = 15.6, 6.0 Hz, 1H), 7.10 – 7.00 (m, 4H), 5.98 (dd, *J* = 16.0, 1.6 Hz, 1H), 5.96 – 5.88 (m, 2H), 4.85 – 4.73 (m, 1H), 3.16 – 3.05 (m, 2H), 2.70 – 2.58 (m, 2H), 2.23 (dd, *J* = 14.8, 6.4 Hz, 2H), 2.15 – 2.03 (m, 1H), 1.85 – 1.73 (m, 1H), 1.29 – 1.18 (m, 2H), 0.91 (t, *J* = 7.6 Hz, 3H); ^{13}C NMR (100 MHz, $CDCl_3$) δ 179.58 (d, *J* = 11.3 Hz), 164.17, 161.40, 158.97, 146.42, 146.32 (d, *J* = 2.8 Hz), 127.95, 122.89 (d, *J* = 8.4 Hz), 121.65, 116.13, 115.90, 52.27, 38.97 (d, *J* = 10.8 Hz), 32.42, 23.61, 19.32, 13.36;

Enantiomeric excess: 91%, determined by HPLC (Chiralpak OD-H, hexane/ *i*-PrOH = 90/10; flow rate 0.8 ml/min; 25 ℃; 210 nm), first peak: t_R = 37.0 min, second peak: t_R = 50.8 min. HRMS (ESI) calcd. For $C_{21}H_{22}FNNaO_4$ $[M+Na]^+$: 394.1425, found: 394.1431.

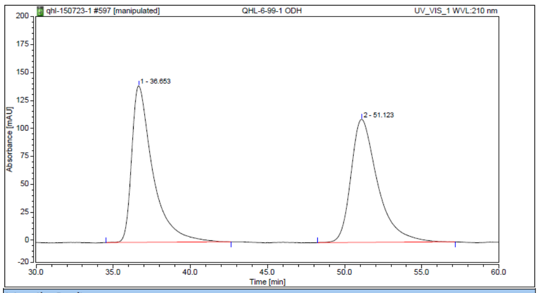

Integration Results

No.	Peak Name	Retention Time min	Area mAU*min	Height mAU	Relative Area %	Relative Height %	Amount n.a.
1		36.653	221.645	140.117	50.06	55.98	n.a.
2		51.123	221.085	110.191	49.94	44.02	n.a.
Total:			442.730	250.307	100.00	100.00	

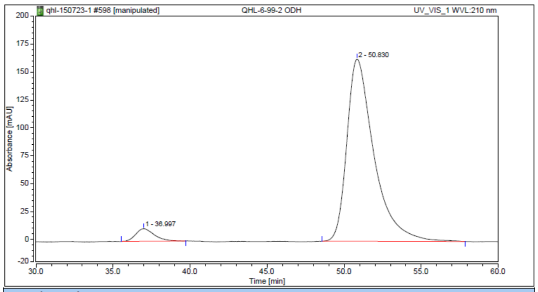

Integration Results

No.	Peak Name	Retention Time min	Area mAU*min	Height mAU	Relative Area %	Relative Height %	Amount n.a.
1		36.997	15.530	11.306	4.46	6.48	n.a.
2		50.830	333.009	163.245	95.54	93.52	n.a.
Total:			348.539	174.551	100.00	100.00	

5.1.15 4-fluorophenyl (4*S*, *E*)-4-(1,3-dioxo-1,3,3a,4,7,7a-hexahydro-2H-isoindol-2-yl)tetradec-2-enoate (6ad).

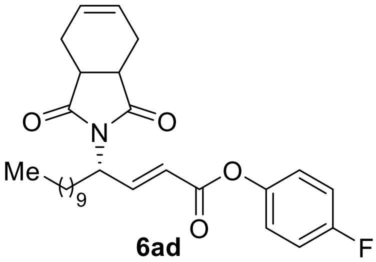

6ad

The general procedure was followed using **4a** (0.1 mmol) and **5d** (0.2 mmol). After purification by column chromatography (PE/EtOAc = 4:1), **6ad** (38.9 mg, 83%) was obtained. $[\alpha]^{22}_D$ = 7.6 (*c* 0.25, acetone); ^{1}H NMR (400 MHz, $CDCl_3$) δ 7.21 (dd, *J* = 15.6, 6.4 Hz, 1H), 7.05 (d, *J* = 6.4 Hz, 4H), 5.98 (dd, *J* = 15.6, 1.2 Hz, 1H), 5.96 – 5.90 (m, 2H), 4.82 – 4.71 (m, 1H), 3.17 – 3.04 (m, 2H), 2.65 (dd, *J* = 10.4, 4.8 Hz, 2H), 2.23 (dd, *J* = 14.8, 6.8 Hz, 2H), 2.16 – 2.01 (m, 1H), 1.89 – 1.75 (m, 1H), 1.31 – 1.17 (m, 16H), 0.87 (t, *J* = 6.4 Hz, 3H); ^{13}C NMR (100 MHz, $CDCl_3$) δ 179.62 (d, *J* = 10.1 Hz), 164.22, 161.40, 158.97, 146.49, 146.30 (d, *J* = 2.9 Hz), 128.00, 122.90 (d, *J* = 8.5 Hz), 121.61, 116.15, 115.92, 52.59, 38.97 (d, *J* = 12.1 Hz), 31.86, 30.39, 29.52, 29.45, 29.39, 29.28, 28.87, 26.08, 23.64, 22.65, 14.10; Enantiomeric excess: 91%, determined by HPLC (Chiralpak OD-H, hexane/*i*-PrOH = 90/10; flow rate 0.8 ml/min;

25 ℃; 210 nm), first peak: t_R = 28.9 min, second peak: t_R = 35.9 min. HRMS (ESI) calcd. For $C_{28}H_{36}FNNaO_4$ $[M+Na]^+$: 492.2521, found: 492.2521.

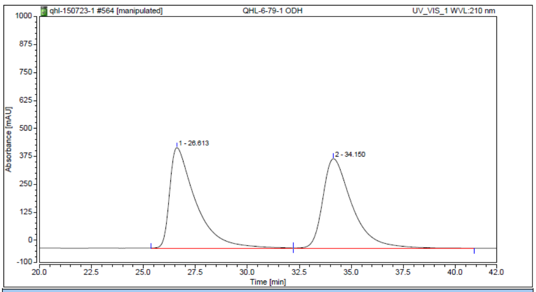

Integration Results							
No.	Peak Name	Retention Time min	Area mAU*min	Height mAU	Relative Area %	Relative Height %	Amount n.a.
1		26.613	654.098	449.932	49.96	52.90	n.a.
2		34.150	655.163	400.617	50.04	47.10	n.a.
Total:			1309.261	850.550	100.00	100.00	

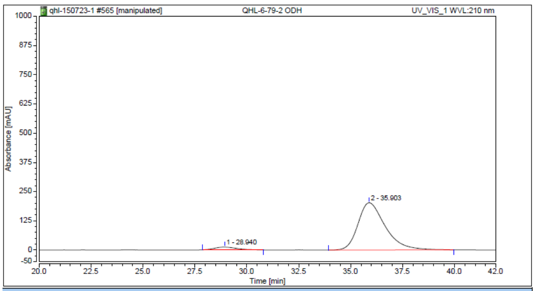

Integration Results							
No.	Peak Name	Retention Time min	Area mAU*min	Height mAU	Relative Area %	Relative Height %	Amount n.a.
1		28.940	14.310	12.060	4.44	5.63	n.a.
2		35.903	307.716	202.192	95.56	94.37	n.a.
Total:			322.026	214.252	100.00	100.00	

5.1.16 4-fluorophenyl (4*S*, *E*)-4-(1,3-dioxo-1,3,3a,4,7,7a-hexahydro-2H-isoindol-2-yl)-5-methylhex-2-enoate (6ae).

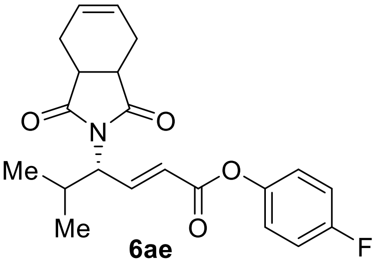

6ae

The general procedure was followed using **4a** (0.1 mmol) and **5e** (0.2 mmol). After purification by column chromatography (PE/EtOAc = 5:1), **6ae** (27.7 mg, 75%) was obtained. $[\alpha]^{22}_D$ = 13.6 (*c* 0.25, acetone); ^{1}H NMR (400 MHz, $CDCl_3$) δ 7.33 (dd, *J* = 16.0, 8.4 Hz, 1H), 7.12 – 7.00 (m, 4H), 6.04 (dd, *J* = 15.6, 0.4 Hz, 1H), 5.98 – 5.89 (m, 2H), 4.35 – 4.25 (m, 1H), 3.15 – 3.05 (m, 2H), 2.64 (d, *J* = 14.0 Hz, 2H), 2.53 (qd, *J* = 13.2, 6.8 Hz, 1H), 2.23 (dd, *J* = 14.8, 7.2 Hz, 2H), 0.97 (d, *J* = 6.4 Hz, 3H), 0.79 (d, *J* = 6.8 Hz, 3H); ^{13}C NMR (100 MHz, $CDCl_3$) δ 179.62 (d, *J* = 11.7 Hz), 164.06, 161.39, 158.96, 146.30 (d, *J* = 2.8 Hz), 145.09, 128.03 (d, *J* = 2.2 Hz), 123.54, 122.87 (d, *J* = 8.4 Hz), 116.12, 115.89, 59.60, 39.04, 38.80, 28.21, 23.59 (d, *J* = 7.5 Hz), 20.16, 19.44; Enantiomeric excess: 93%, determined by HPLC (Chiralpak OD-H, hexane/ *i*-PrOH = 90/10; flow rate 0.8 ml/min; 25 ℃; 210 nm), first peak: t_R = 19.3 min, second peak: t_R = 22.9 min. HRMS (ESI) calcd. For $C_{21}H_{22}FNNaO_4$ $[M+Na]^+$: 394.1425, found: 394.1427.

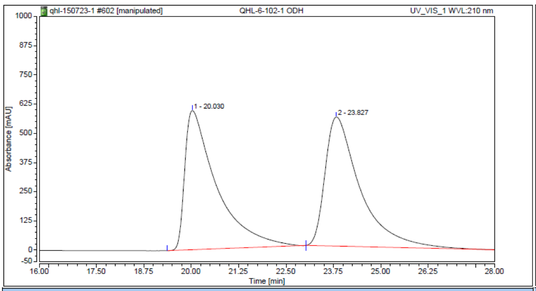

Integration Results

No.	Peak Name	Retention Time min	Area mAU*min	Height mAU	Relative Area %	Relative Height %	Amount n.a.
1		20.030	578.771	596.204	49.41	51.88	n.a.
2		23.827	592.542	553.048	50.59	48.12	n.a.
**Total:**			**1171.312**	**1149.252**	**100.00**	**100.00**	

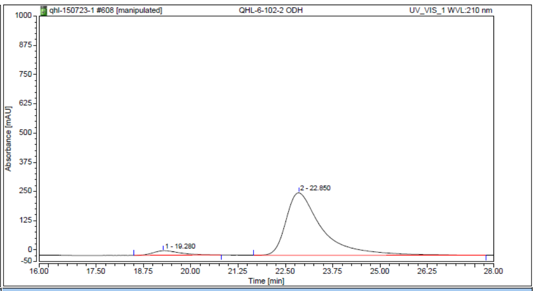

Integration Results

No.	Peak Name	Retention Time min	Area mAU*min	Height mAU	Relative Area %	Relative Height %	Amount n.a.
1		19.280	15.785	19.150	5.06	6.68	n.a.
2		22.850	296.389	267.541	94.94	93.32	n.a.
**Total:**			**312.175**	**286.691**	**100.00**	**100.00**	

5.1.17 4-fluorophenyl (4*S*, *E*)-4-cyclopentyl-4-(1,3-dioxo-1,3,3a,4,7,7a-hexahydro -2H-isoindol-2-yl)but-2-enoate (6af).

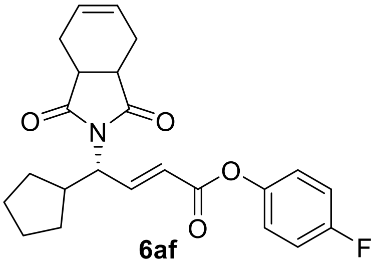

The general procedure was followed using **4a** (0.1 mmol) and **5f** (0.2 mmol). After purification by column chromatography (PE/EtOAc = 5:1), **6af** (39.4 mg, 98%) was obtained. $[\alpha]^{22}_D$ = -6.0 (*c* 0.25, acetone); ^{1}H NMR (400 MHz, $CDCl_3$) δ 7.27 (dd, *J* = 16.0, 7.6 Hz, 1H), 7.09 – 7.02 (m, 4H), 5.99 (dd, *J* = 15.6, 0.8 Hz, 1H), 5.96 – 5.92 (m, 2H), 4.46 (dd, *J* = 10.0, 7.2 Hz, 1H), 3.17 – 3.05 (m, 2H), 2.86 – 2.72 (m, 1H), 2.65 (dd, *J* = 10.8, 4.4 Hz, 2H), 2.23 (dd, *J* = 14.8, 6.8 Hz, 2H), 1.93 – 1.78 (m, 1H), 1.72 – 1.58 (m, 3H), 1.57 – 1.48 (m, 2H), 1.23 – 1.15 (m, 1H), 1.09 – 0.97 (m, 1H); ^{13}C NMR (100 MHz, $CDCl_3$) δ 179.67 (d, *J* = 11.4 Hz), 164.22, 161.40, 158.97, 146.31 (d, *J* = 2.9 Hz), 145.52, 127.99, 122.89 (d, *J* = 8.4 Hz), 122.29, 116.14, 115.90, 58.04, 39.83, 39.04, 38.84, 30.80, 30.16, 25.37, 24.77, 23.62 (d, *J* = 4.2 Hz); Enantiomeric excess: 88%, determined by HPLC (Chiralpak OD-H, hexane/*i*-PrOH = 90/10; flow rate 0.8 ml/min; 25 ℃; 210 nm), first peak: t_R = 19.4 min, second peak: t_R = 23.0 min. HRMS (ESI) calcd. For $C_{23}H_{24}FNNaO_4$ $[M+Na]^+$: 420.1581, found: 420.1582.

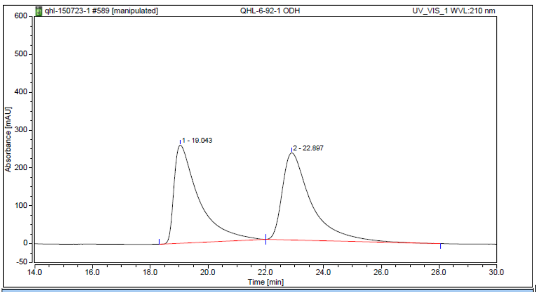

Integration Results

No.	Peak Name	Retention Time min	Area mAU*min	Height mAU	Relative Area %	Relative Height %	Amount n.a.
1		19.043	257.788	259.497	49.15	52.97	n.a.
2		22.897	266.665	230.413	50.85	47.03	n.a.
Total:			524.453	489.910	100.00	100.00	

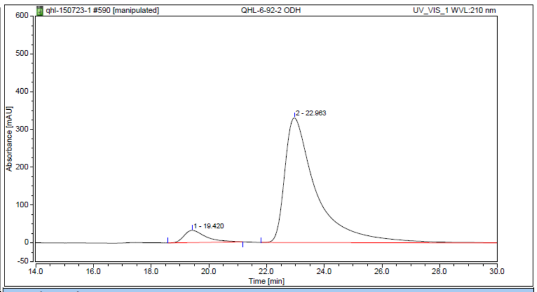

Integration Results

No.	Peak Name	Retention Time min	Area mAU*min	Height mAU	Relative Area %	Relative Height %	Amount n.a.
1		19.420	28.049	32.306	6.27	8.92	n.a.
2		22.963	419.638	329.909	93.73	91.08	n.a.
Total:			447.687	362.216	100.00	100.00	

5.1.18 4-fluorophenyl (4*S*, *E*)-4-cyclohexyl-4-(1,3-dioxo-1,3,3a,4,7,7a-hexahydro-2H-isoindol-2-yl)but-2-enoate (6ag).

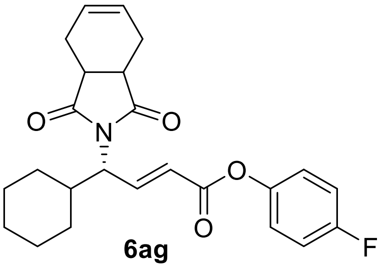

6ag

The general procedure was followed using **4a** (0.1 mmol) and **5g** (0.2 mmol). After purification by column chromatography (PE/EtOAc = 5:1), **6ag** (22.0 mg, 54%) was obtained. $[\alpha]^{22}_{D}$ = 10.8 (*c* 0.25, acetone); ^{1}H NMR (400 MHz, $CDCl_3$) δ 7.34 (dd, *J* = 15.6, 8.4 Hz, 1H), 7.13 – 7.03 (m, 4H), 6.02 (d, *J* = 15.6 Hz, 1H), 5.97 – 5.89 (m, 2H), 4.46 – 4.32 (m, 1H), 3.15 – 3.04 (m, 2H), 2.63 (dd, *J* = 15.6, 2.4 Hz, 2H), 2.27 – 2.21 (m, 2H), 1.82 – 1.64 (m, 4H), 1.46 (d, *J* = 12.6 Hz, 1H), 1.28 – 1.21 (m, 2H), 1.18 – 1.08 (m, 2H), 0.97 – 0.87 (m, 1H), 0.87 – 0.73 (m, 1H); ^{13}C NMR (100 MHz, $CDCl_3$) δ 179.67 (d, *J* = 11.1 Hz), 164.06, 161.40, 158.97, 146.32 (d, *J* = 2.9 Hz), 144.99, 127.98 (d, *J* = 4.6 Hz), 123.59, 122.88 (d, *J* = 8.5 Hz), 116.13, 115.89, 58.38, 39.01, 38.81, 36.74, 30.62, 29.40, 25.94, 25.36 (d, *J* = 4.6 Hz), 23.59 (d, *J* = 7.8 Hz); Enantiomeric excess: 89%, determined by HPLC (Chiralpak OD-H, hexane/*i*-PrOH = 90/10; flow rate 0.8 ml/min; 25 ℃; 210 nm), first peak: t_R = 17.9 min, second peak: t_R = 21.1 min. HRMS (ESI) calcd. For $C_{24}H_{26}FNNaO_4$ $[M+Na]^+$: 434.1738, found: 434.1736.

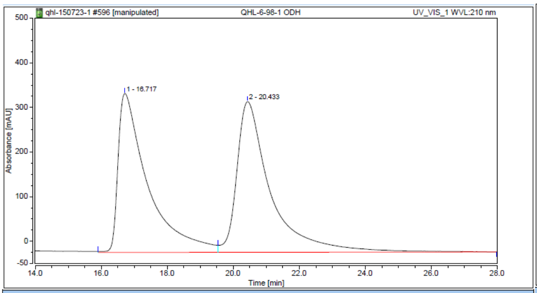

Integration Results

No.	Peak Name	Retention Time min	Area mAU*min	Height mAU	Relative Area %	Relative Height %	Amount n.a.
1		16.717	371.433	356.773	48.05	51.36	n.a.
2		20.433	401.626	337.861	51.95	48.64	n.a.
**Total:**			**773.060**	**694.634**	**100.00**	**100.00**	

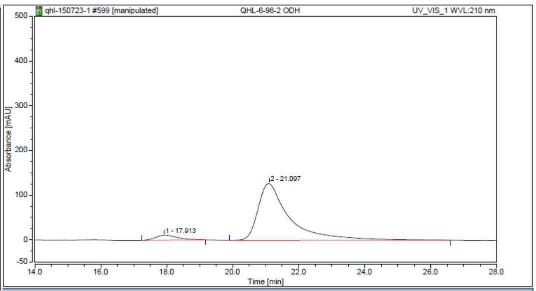

Integration Results

No.	Peak Name	Retention Time min	Area mAU*min	Height mAU	Relative Area %	Relative Height %	Amount n.a.
1		17.913	8.308	10.848	5.58	7.88	n.a.
2		21.097	140.466	126.799	94.42	92.12	n.a.
**Total:**			**148.774**	**137.647**	**100.00**	**100.00**	

5.1.19 4-fluorophenyl (4*S*, *E*)-7-chloro-4-(1,3-dioxo-1,3,3a,4,7,7a-hexahydro-2H-isoindol-2-yl)hept-2-enoate (6ah).

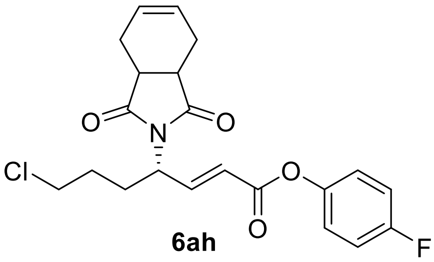

6ah

The general procedure was followed using **4a** (0.1 mmol) and **5h** (0.2 mmol). After purification by column chromatography (PE/EtOAc = 5:1), **6ah** (29.1 mg, 80%) was obtained. $[\alpha]^{22}_{D}$ = 10.8 (*c* 0.25, acetone); ^{1}H NMR (400 MHz, $CDCl_3$) δ 7.20 (dd, *J* = 16.0, 6.4 Hz, 1H), 7.09 – 7.02 (m, 4H), 6.03 (dd, *J* = 15.6, 1.2 Hz, 1H), 5.99 – 5.91 (m, 2H), 4.84 – 4.74 (m, 1H), 3.51 (t, *J* = 6.4 Hz, 2H), 3.17 – 3.08 (m, 2H), 2.71 – 2.58 (m, 2H), 2.22 (dd, *J* = 14.8, 6.4 Hz, 2H), 2.06 – 1.96 (m, 1H), 1.72 – 1.65 (m, 2H), 1.22 (dt, *J* = 19.6, 7.2 Hz, 1H); ^{13}C NMR (100 MHz, $CDCl_3$) δ 179.52 (d, *J* = 7.8 Hz), 164.00, 161.42, 158.99, 146.26 (d, *J* = 2.8 Hz), 145.58, 128.07 (d, *J* = 4.3 Hz), 122.85 (d, *J* = 8.5 Hz), 122.22, 116.16, 115.92, 51.71, 43.70, 39.01 (d, *J* = 13.5 Hz), 28.99, 27.70, 23.63; Enantiomeric excess: 88%, determined by HPLC (Chiralpak IC, hexane/*i*-PrOH = 90/10; flow rate 0.8 ml/min; 25 ℃; 210 nm), first peak: t_R = 35.2 min, second peak: t_R = 42.6 min. HRMS (ESI) calcd. For $C_{21}H_{21}ClFNNaO_4$ $[M+Na]^+$: 428.1042, found: 428.1035.

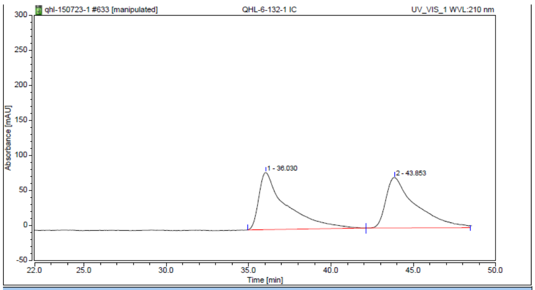

Integration Results

No.	Peak Name	Retention Time min	Area mAU*min	Height mAU	Relative Area %	Relative Height %	Amount n.a.
1		36.030	157.851	81.619	51.00	53.10	n.a.
2		43.853	151.659	72.103	49.00	46.90	n.a.
Total:			309.509	153.722	100.00	100.00	

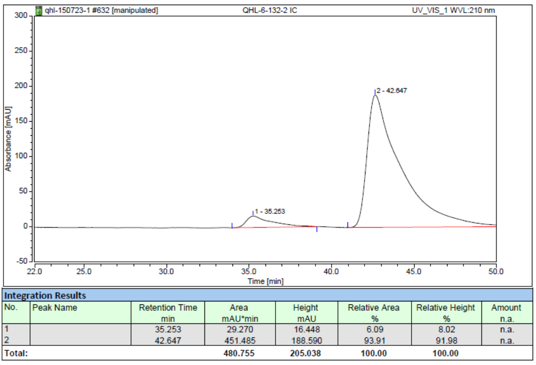

Integration Results

No.	Peak Name	Retention Time min	Area mAU*min	Height mAU	Relative Area %	Relative Height %	Amount n.a.
1		35.253	29.270	16.448	6.09	8.02	n.a.
2		42.647	451.485	188.590	93.91	91.98	n.a.
Total:			480.755	205.038	100.00	100.00	

5.1.20 4-fluorophenyl (4*S*, *E*)-8-bromo-4-(1,3-dioxo-1,3,3a,4,7,7a-hexahydro-2H-isoindol-2-yl)oct-2-enoate (6ai).

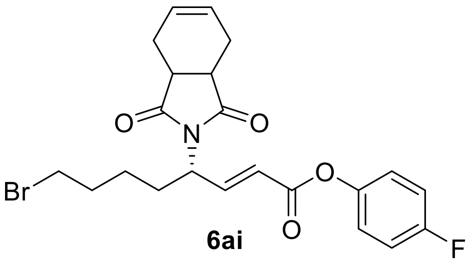

6ai

The general procedure was followed using **4a** (0.1 mmol) and **5i** (0.2 mmol). After purification by column chromatography (PE/EtOAc = 6:1), **6ai** (35.0 mg, 75%) was obtained. $[\alpha]^{22}_{D}$ = 5.2 (*c* 0.25, acetone); ^{1}H NMR (400 MHz, $CDCl_3$) δ 7.20 (dd, *J* = 15.6, 6.4 Hz, 1H), 7.12 – 7.00 (m, 4H), 6.00 (dd, *J* = 16.0, 1.2 Hz, 1H), 5.98 – 5.93 (m, 2H), 4.84 – 4.72 (m, 1H), 3.42 – 3.31 (m, 2H), 3.17 – 3.08 (m, 2H), 2.65 (dd, *J* = 10.4, 4.8 Hz, 2H), 2.29 – 2.20 (m, 2H), 2.19 – 2.10 (m, 1H), 1.93 – 1.72 (m, 3H), 1.43 – 1.31 (m, 2H); ^{13}C NMR (100 MHz, $CDCl_3$) δ 179.57 (d, *J* = 9.1 Hz), 164.07, 161.42, 158.99, 146.29 (d, *J* = 2.9 Hz), 145.91, 128.05, 122.87 (d, *J* = 8.4 Hz), 121.99, 116.15, 115.92, 99.95, 52.24, 39.01 (d, *J* = 11.6 Hz), 33.22, 31.75, 29.53, 24.62, 23.61; Enantiomeric excess: 92%, determined by HPLC (Chiralpak IC, hexane/*i*-PrOH = 90/10; flow rate 0.8 ml/min; 25 ℃; 254 nm), first peak: t_R = 37.4 min, second peak: t_R = 43.6 min. HRMS (ESI) calcd. For $C_{22}H_{23}BrFNNaO_4$ $[M+Na]^+$: 486.0687, found: 486.0680.

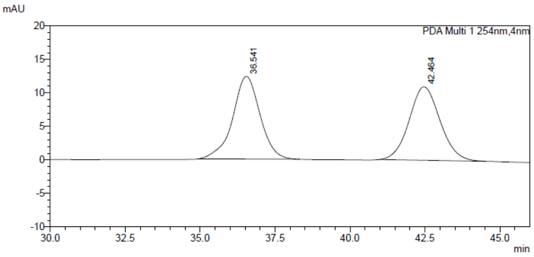

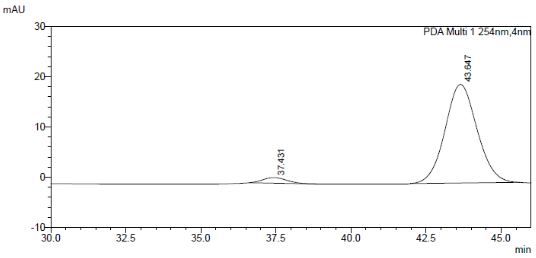

<Peak Table>

PDA Ch1 254nm

Peak#	Ret. Time	Area	Area%	Height	Height%
1	36.541	785881	49.969	12350	52.957
2	42.464	786863	50.031	10971	47.043
Total		1572744	100.000	23321	100.000

PDA Ch1 254nm

Peak#	Ret. Time	Area	Area%	Height	Height%
1	37.431	59824	3.943	1105	5.331
2	43.647	1457385	96.057	19633	94.669
Total		1517209	100.000	20739	100.000

5.1.21 1-(4-fluorophenyl)-7-methyl-(4*S*, *E*)-4-(1,3-dioxo-1,3,3a,4,7,7a-hexahydro-2H-isoindol-2-yl)hept-2-enedioate (6aj).

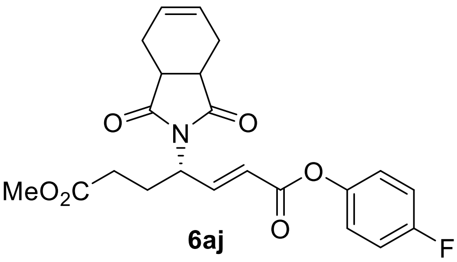

6aj

The general procedure was followed using **4a** (0.1 mmol) and **5j** (0.2 mmol). After purification by column chromatography (PE/EtOAc = 5:1), **6aj** (34.0 mg, 82%) was obtained. $[\alpha]^{22}_{D}$ = 13.6 (*c* 0.25, acetone); ^{1}H NMR (400 MHz, $CDCl_3$) δ 7.18 (dd, *J* = 15.6, 6.0 Hz, 1H), 7.05 (d, *J* = 6.4 Hz, 4H), 6.02 (dd, *J* = 15.6, 1.6 Hz, 1H), 5.99 – 5.91 (m, 2H), 4.87 – 4.80 (m, 1H), 3.66 (s, 3H), 3.18 – 3.06 (m, 2H), 2.64 (d, *J* = 17.6 Hz, 2H), 2.44 – 2.34 (m, 1H), 2.28 – 2.17 (m, 5H); ^{13}C NMR (100 MHz, $CDCl_3$) δ 179.45 (d, *J* = 12.5 Hz), 172.38, 163.95, 161.40, 158.97, 146.25 (d, *J* = 2.8 Hz), 145.34, 128.06 (d, *J* = 8.2 Hz), 122.85 (d, *J* = 8.5 Hz), 122.33, 116.13, 115.89, 51.78 (d, *J* = 0.6 Hz), 51.56, 38.99 (d, *J* = 8.8 Hz), 30.34, 25.56, 23.62, 23.59 (d, *J* = 6.0 Hz); Enantiomeric excess: 91%, determined by HPLC (Chiralpak IE, hexane/*i*-PrOH = 70/30; flow rate 0.8 ml/min; 25 ℃; 190 nm), first peak: t_R = 31.0 min, second peak: t_R = 34.2 min. HRMS (ESI) calcd. For $C_{22}H_{22}FNNaO_6$ $[M+Na]^+$: 438.1323, found: 438.1322.

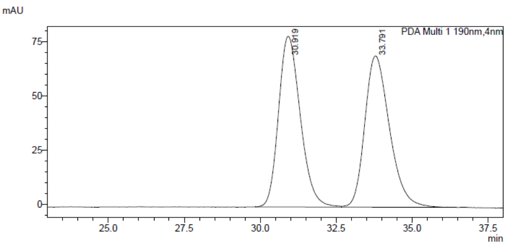

PDA Ch1 190nm

Peak#	Ret. Time	Area	Area%	Height	Height%
1	30.919	3921824	49.865	78548	53.030
2	33.791	3943100	50.135	69572	46.970
Total		7864923	100.000	148120	100.000

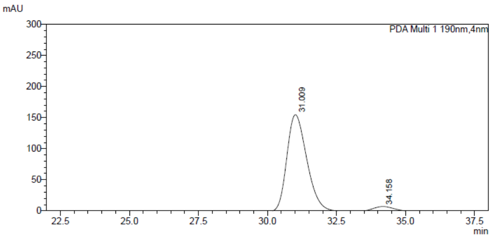

PDA Ch1 190nm

Peak#	Ret. Time	Area	Area%	Height	Height%
1	31.009	7784759	95.209	155535	95.408
2	34.158	391770	4.791	7486	4.592
Total		8176529	100.000	163021	100.000

5.1.22 4-fluorophenyl (4*S*, *E*)-4-(1,3-dioxo-1,3,3a,4,7,7a-hexahydro-2H-isoindol-2-yl)-6-phenylhex-2-enoate (6ak).

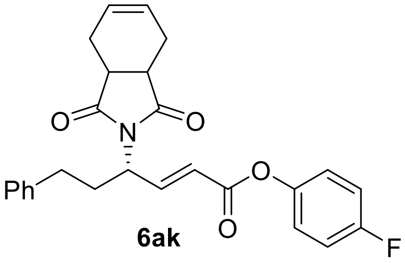

6ak

The general procedure was followed using **4a** (0.1 mmol) and **5k** (0.2 mmol). After purification by column chromatography (PE/EtOAc = 5:1), **6ak** (35.1 mg, 81%) was obtained. $[\alpha]^{22}_{D}$ = -6.4 (*c* 0.25, acetone); ^{1}H NMR (400 MHz, $CDCl_3$) δ 7.26 – 7.21 (m, 2H), 7.19 – 7.12 (m, 3H), 7.09 (d, *J* = 7.2 Hz, 2H), 7.01 (d, *J* = 6.4 Hz, 4H), 5.98 – 5.85 (m, 3H), 4.85 – 4.75 (m, 1H), 3.00 – 2.86 (m, 2H), 2.63 – 2.57 (m, 2H), 2.55 – 2.49 (m, 2H), 2.20 – 2.15 (m, 1H), 2.15 – 2.06 (m, 2H); ^{13}C NMR (100 MHz, $CDCl_3$) δ 179.58 (d, *J* = 11.1 Hz), 164.08, 161.38, 158.96, 146.28 (d, *J* = 2.8 Hz) 146.09, 140.26, 128.47, 128.42, 128.39 (d, *J* = 6.6 Hz), 128.27, 128.06 (d, *J* = 4.9 Hz), 126.23, 125.97, 122.86 (d, *J* = 8.5 Hz), 121.79, 116.12, 115.88, 52.46, 38.94 (d, *J* = 4.4 Hz), 32.64, 31.53, 23.57 (d, *J* = 3.8 Hz); Enantiomeric excess: 85%, determined by HPLC (Chiralpak IE, hexane/*i*-PrOH = 80/20; flow rate 0.8 ml/min; 25 ℃; 254 nm), first peak: t_R = 16.9 min, second peak: t_R = 18.8 min. HRMS (ESI) calcd. For $C_{26}H_{24}FNNaO_4$ $[M+Na]^+$: 456.1582, found: 456.1584.

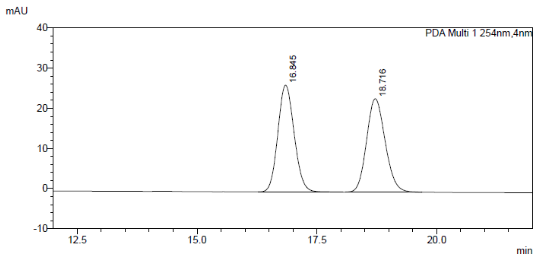

<Peak Table>
PDA Ch1 254nm

Peak#	Ret. Time	Area	Area%	Height	Height%
1	16.845	628781	50.079	26585	53.352
2	18.716	626789	49.921	23245	46.648
Total		1255570	100.000	49830	100.000

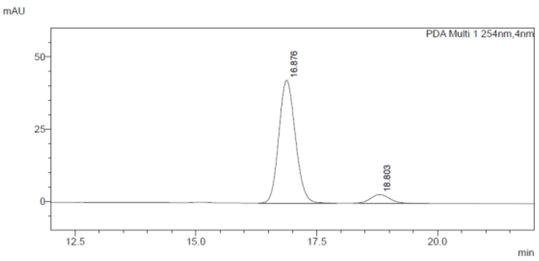

<Peak Table>
PDA Ch1 254nm

Peak#	Ret. Time	Area	Area%	Height	Height%
1	16.876	1007083	92.545	42447	93.379
2	18.803	81125	7.455	3010	6.621
Total		1088207	100.000	45457	100.000

5.1.23 4-fluorophenyl (2*E*, 4*S*, 8*E*)-4-(1,3-dioxo-1,3,3a,4,7,7a-hexahydro-2H-isoindol-2-yl)-10-methylundeca-2,8-dienoate (6al).

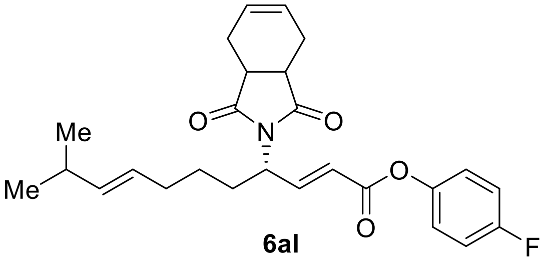

The general procedure was followed using **4a** (0.1 mmol) and **5l** (0.2 mmol). After purification by column chromatography (PE/EtOAc = 5:1), **6al** (29.0 mg, 68%) was obtained. $[\alpha]^{22}_{D}$ = 17.2 (*c* 0.25, acetone); ^{1}H NMR (400 MHz, $CDCl_3$) δ 7.20 (dd, *J* = 15.6, 6.0 Hz, 1H), 7.06 (d, *J* = 6.0 Hz, 4H), 5.99 (dd, *J* = 15.6, 1.2 Hz, 1H), 5.95 – 5.92 (m, 2H), 5.47 – 5.21 (m, 2H), 4.83 – 4.72 (m, 1H), 3.16 – 3.06 (m, 2H), 2.65 (d, *J* = 15.6 Hz, 2H), 2.28 – 2.17 (m, 3H), 2.16 – 2.04 (m, 1H), 2.03 – 1.91 (m, 2H), 1.87 – 1.76 (m, 1H), 1.28 – 1.22 (m, 2H), 0.96 (d, *J* = 6.4 Hz, 6H); ^{13}C NMR (100 MHz, $CDCl_3$) δ 179.60 (d, *J* = 9.6 Hz), 164.20, 161.43, 159.00, 146.40, 146.35 (d, *J* = 2.9 Hz), 138.56, 128.03, 125.88, 122.91 (d, *J* = 8.5 Hz), 121.69, 116.16, 115.92, 52.42, 39.01 (d, *J* = 12.4 Hz), 31.60, 30.97, 29.76, 25.99, 23.65, 22.61; Enantiomeric excess: 90%, determined by HPLC (Chiralpak OD-H, hexane/*i*-PrOH = 85/15; flow rate 0.8 ml/min; 25 ℃; 210 nm), first peak: t_R = 19.0 min, second peak: t_R = 30.0 min. HRMS (ESI) calcd. For $C_{26}H_{30}FNNaO_4$ $[M+Na]^+$: 462.2057, found: 462.2051.

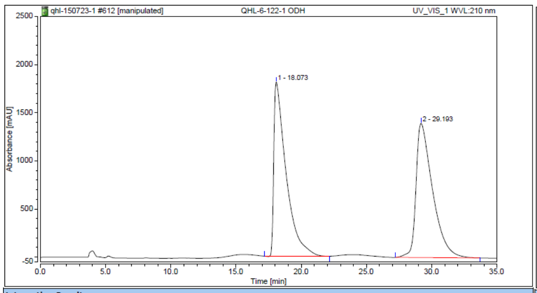

Integration Results

No.	Peak Name	Retention Time min	Area mAU*min	Height mAU	Relative Area %	Relative Height %	Amount n.a.
1		18.073	1969.778	1810.421	49.00	56.47	n.a.
2		29.193	2050.239	1395.679	51.00	43.53	n.a.
Total:			4020.017	3206.100	100.00	100.00	

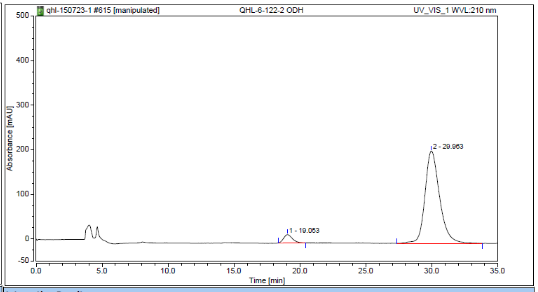

Integration Results

No.	Peak Name	Retention Time min	Area mAU*min	Height mAU	Relative Area %	Relative Height %	Amount n.a.
1		19.053	14.462	18.651	5.16	8.25	n.a.
2		29.963	266.038	207.558	94.84	91.75	n.a.
Total:			280.500	226.209	100.00	100.00	

5.1.24 4-fluorophenyl-(2*E*, 4*S*, 11*E*)-4-(1,3-dioxo-1,3,3a,4,7,7a-hexahydro-2H-isoindol-2-yl)icosa-2,11-dienoate (6am).

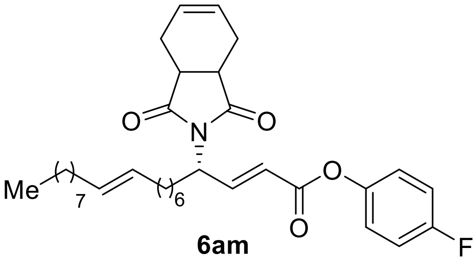

6am

The general procedure was followed using **4a** (0.1 mmol) and **5m** (0.2 mmol). After purification by column chromatography (PE/EtOAc = 5:1), **6am** (47.4 mg, 86%) was obtained. $[\alpha]^{22}_{D}$ = 6.0 (*c* 0.25, acetone); ^{1}H NMR (400 MHz, $CDCl_3$) δ 7.21 (dd, *J* = 16.0, 6.4 Hz, 1H), 7.05 (d, *J* = 6.0 Hz, 4H), 5.99 (dd, *J* = 15.6, 1.2 Hz, 1H), 5.96 – 5.89 (m, 2H), 5.40 – 5.29 (m, 2H), 4.77 (dt, *J* = 10.0, 6.0 Hz, 1H), 3.16 – 3.06 (m, 2H), 2.65 (d, *J* = 15.2 Hz, 2H), 2.23 (dd, *J* = 14.4, 6.0 Hz, 2H), 2.16 – 2.06 (m, 1H),2.06 – 1.92 (m, 4H), 1.87 – 1.75 (m, 1H), 1.38 – 1.19 (m, 20H), 0.87 (t, *J* = 6.4 Hz, 3H); ^{13}C NMR (100 MHz, $CDCl_3$) δ 179.58 (d, *J* = 10.1 Hz), 164.17, 161.41, 158.98, 146.44, 146.34 (d, *J* = 2.8 Hz), 130.07, 129.55, 127.99, 122.89 (d, *J* = 8.5 Hz), 121.66, 116.13, 115.89, 52.57, 38.97 (d, *J* = 12.0 Hz), 31.86, 30.40, 29.72, 29.65 (d, *J* = 0.9 Hz), 29.55 (d, *J* = 2.4 Hz), 29.48, 29.44, 29.03, 28.79, 27.14 (d, *J* = 10.5 Hz), 26.06, 23.62 (d, *J* = 1.5 Hz), 22.59 (d, *J* = 10.7 Hz), 14.08; Enantiomeric excess: 90%, determined by HPLC (Chiralpak IB, hexane/*i*-PrOH = 90/10; flow rate 0.8 ml/min; 25 ℃; 254 nm), first peak: t_R = 20.4 min, second peak: t_R = 22.7 min. HRMS (ESI) calcd. For $C_{34}H_{46}FNNaO_4$ $[M+Na]^+$: 574.3303, found: 574.3323.

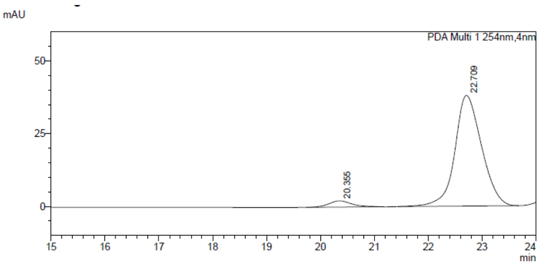

Peak#	Ret. Time	Area	Area%	Height	Height%
1	20.355	63834	4.644	2136	5.316
2	22.709	1310701	95.356	38047	94.684
Total		1374535	100.000	40183	100.000

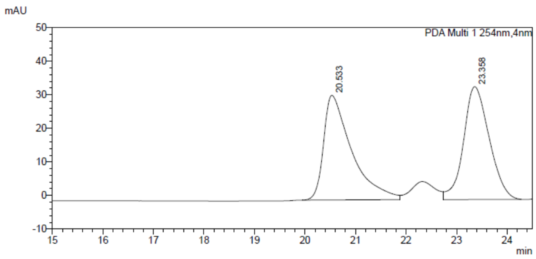

<Peak Table>

PDA Ch1 254nm

Peak#	Ret. Time	Area	Area%	Height	Height%
1	20.533	1204648	50.360	31175	48.117
2	23.358	1187412	49.640	33615	51.883
Total		2392059	100.000	64790	100.000

5.1.25 4-fluorophenyl (4*S*, *E*)-4-(1,3-dioxo-1,3,3a,4,7,7a-hexahydro-2H-isoindol-2-yl)trideca-2,12-dienoate (6an).

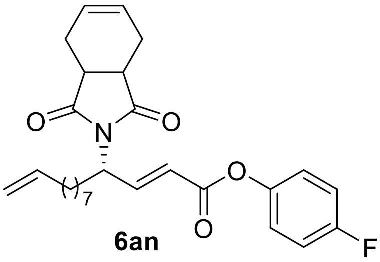

6an

The general procedure was followed using **4a** (0.1 mmol) and **5n** (0.2 mmol). After purification by column chromatography (PE/EtOAc = 6:1), **6an** (34.8mg, 77%) was obtained. $[\alpha]^{22}_{D}$ = 7.2 (*c* 0.25, acetone); ^{1}H NMR (400 MHz, $CDCl_3$) δ 7.21 (dd, *J* = 15.6, 6.0 Hz, 1H), 7.05 (d, *J* = 6.4 Hz, 4H), 5.98 (dd, *J* = 15.6, 1.2 Hz, 1H), 5.96 – 5.90 (m, 2H), 5.80 (ddt, *J* = 17.2, 10.4, 6.8 Hz, 1H), 4.95 (ddd, *J* = 18.8, 17.2, 1.6 Hz, 2H), 4.82 – 4.71 (m, 1H), 3.17 – 3.05 (m, 2H), 2.65 (dd, *J* = 10.4, 4.4 Hz, 2H), 2.23 (dd, *J* = 14.8, 6.8 Hz, 2H), 2.14 – 2.06 (m, 1H), 2.03 (dd, *J* = 14.4, 6.8 Hz, 2H), 1.86 – 1.74 (m, 1H), 1.36 – 1.17 (m, 10H); ^{13}C NMR (100 MHz, $CDCl_3$) δ 179.60 (d, *J* = 10.0 Hz), 164.19, 161.39, 158.96, 146.44, 146.30 (d, *J* = 2.8 Hz), 139.04, 127.99, 122.88 (d, *J* = 8.4 Hz), 121.63, 116.13, 115.90, 114.19, 52.56, 38.96 (d, *J* = 12.1 Hz), 33.71, 30.37, 29.20, 28.87, 28.78, 26.04, 23.62 (d, *J* = 1.7 Hz); Enantiomeric excess: 90%, determined by HPLC (Chiralpak OD-H, hexane/*i*-PrOH = 90/10; flow rate 0.8 ml/min; 25 ℃; 210 nm), first peak: t_R = 36.6 min, second peak: t_R = 42.8 min. HRMS (ESI) calcd. For $C_{27}H_{32}FNNaO_4$ $[M+Na]^+$: 476.2208, found: 476.2219.

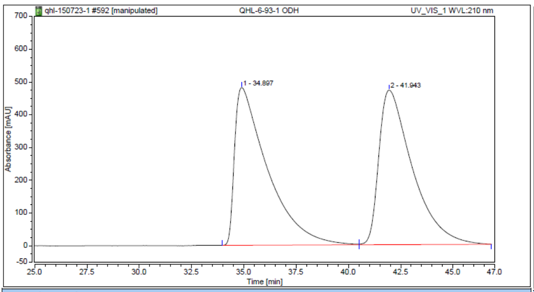

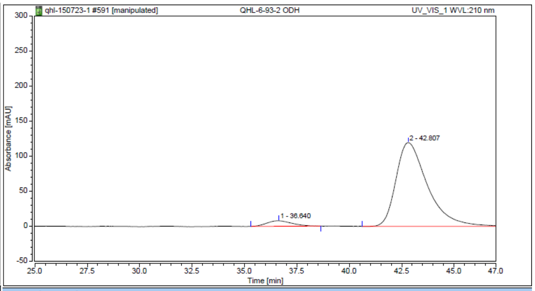

Integration Results

No.	Peak Name	Retention Time min	Area mAU*min	Height mAU	Relative Area %	Relative Height %	Amount n.a.
1		34.897	879.860	481.537	50.12	50.52	n.a.
2		41.943	875.728	471.693	49.88	49.48	n.a.
Total:			1755.588	953.230	100.00	100.00	

Integration Results

No.	Peak Name	Retention Time min	Area mAU*min	Height mAU	Relative Area %	Relative Height %	Amount n.a.
1		36.640	11.037	7.917	5.00	6.21	n.a.
2		42.807	209.718	119.641	95.00	93.79	n.a.
Total:			220.754	127.558	100.00	100.00	

5.1.26 ethyl (S,E)-4-(1,3-dioxoisoindolin-2-yl)hex-2-enoate (6lo).

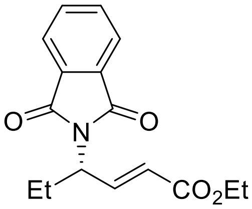

6lo

The general procedure was followed using **4l** (1.0 mmol) and **2a** (2.0 mmol). After purification by column chromatography (PE/EtOAc = 5:1), **6lo** (258.1mg, 90%) was obtained. $[\alpha]^{22}_{D}$ = -7.9 (c 0.20, $CHCl_3$); ^{1}H NMR (400 MHz, $CDCl_3$) δ 7.89 – 7.78 (m, 2H), 7.78 – 7.64 (m, 2H), 7.14 (dd, J = 15.8, 6.8 Hz, 1H), 5.90 (dd, J = 16.0, 1.6 Hz, 1H), 4.82 – 4.74 (m, 1H), 4.15 (q, J = 7.1 Hz, 2H), 2.27 – 2.08 (m, 1H), 2.08 – 1.92 (m, 1H), 1.24 (t, J = 7.2 Hz, 3H), 0.91 (t, J = 7.2 Hz, 3H).; ^{13}C NMR (101 MHz, $CDCl_3$) δ 167.79, 165.86, 144.22, 134.14, 131.72, 123.37, 123.11, 60.56, 53.43, 24.71, 14.18, 10.88; Enantiomeric excess: 88%, determined by HPLC (Chiralpak AD-H, hexane/i-PrOH = 95/05; flow rate 0.8 ml/min; 25 ℃; 290 nm), first peak: t_R = 17.2 min, second peak: t_R = 19.7 min. HRMS (ESI) calcd. For $C_{16}H_{17}NNaO_4$ $[M+Na]^+$: 310.1050, found: 310.1055.

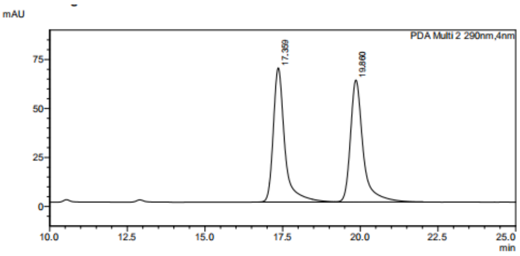

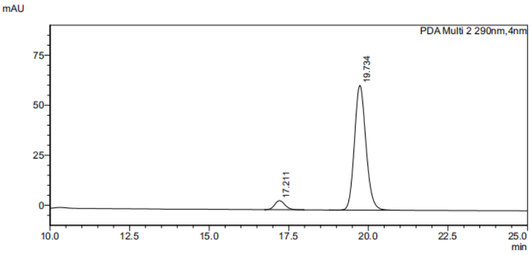

<Peak Table>
PDA Ch2 290nm

Peak#	Ret. Time	Area	Area%	Height	Height%
1	17.359	1765733	49.970	68479	52.364
2	19.860	1767885	50.030	62295	47.636
Total		3533618	100.000	130774	100.000

<Peak Table>
PDA Ch2 290nm

Peak#	Ret. Time	Area	Area%	Height	Height%
1	17.211	101904	6.095	4546	6.795
2	19.734	1570061	93.905	62355	93.205
Total		1671965	100.000	66901	100.000

5.2 General procedure for variation of $TsNH_2$ components.

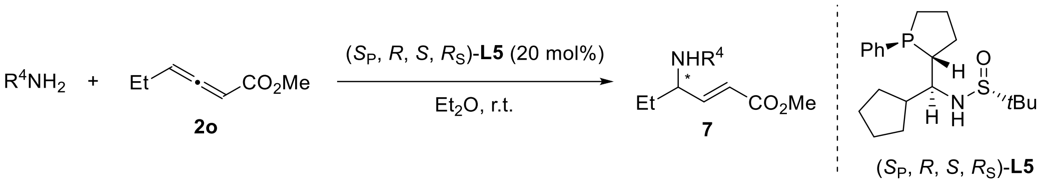

To a flame-dried glass tube with a magnetic stirring bar were added $TsNH_2$ (17.1 mg, 0.10 mmol) and (S_P, R, S, R_S)-**L5** (7.4 mg, 0.02 mmol), followed by the addition of dry Et_2O (1.5 mL).[5] Then the allenoate **2o** (25.2 mg, 0.20 mmol) was slowly added via syringe at room temperature under inert atmosphere. The reaction mixture was stirred for 48 h, and TLC show that the rection was completed. Then Et_2O was removed under reduced pressure. The residue was purified by column chromatography on silica gel (petroleum ether/ethyl acetate = 2:1) to afford **7ao** (17.2 mg, 58% yield).

5.2.1 Methyl (*E*)-4-((4-methylphenyl)sulfonamido)hex-2-enoate (7ao).

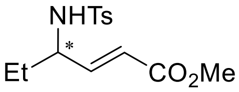

7ao

$[\alpha]^{22}_D$ = 40.2 (*c* 0.33, $CHCl_3$); **^{1}H NMR** (500 MHz, $CDCl_3$) δ 7.73 (d, *J* = 7.0 Hz, 2H), 7.31 – 7.26 (m, 2H), 6.60 (dd, *J* = 15.5, 6.5 Hz, 1H), 5.77 (d, *J* = 15.5 Hz, 1H), 5.02 (d, *J* = 7.5 Hz, 1H), 3.88 – 3.79 (m, 1H), 3.69 (s, 3H), 2.41 (s, 3H), 1.59 – 1.50 (m, 2H), 0.83 (t, *J* = 7.0 Hz, 3H); **^{13}C NMR** (125 MHz, $CDCl_3$) δ 166.29, 146.76, 143.60, 137.63, 129.68, 127.16, 121.69, 56.02, 51.64, 28.07, 21.51, 9.71; Enantiomeric excess: 87%, determined by HPLC (Chiralpak IF hexane/*i*-PrOH = 75/25; flow rate 0.7 ml/min; 25 ℃; 254 nm), first peak: t_R = 14.9 min, second peak: t_R = 18.7 min. HRMS (ESI) calcd. for $C_{14}H_{19}NNaO_4S$ $[M+Na]^+$: 320.0927, found: 320.0919.

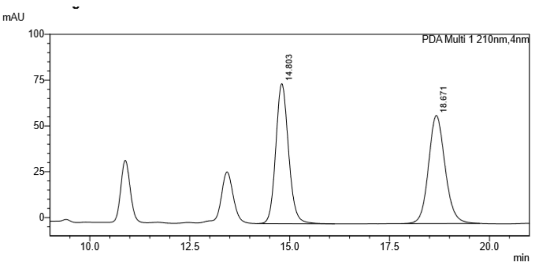

<Peak Table>
PDA Ch1 210nm

Peak#	Ret. Time	Area	Area%	Height	Height%
1	14.803	1699247	49.978	76411	56.461
2	18.671	1700760	50.022	58922	43.539
Total		3400007	100.000	135333	100.000

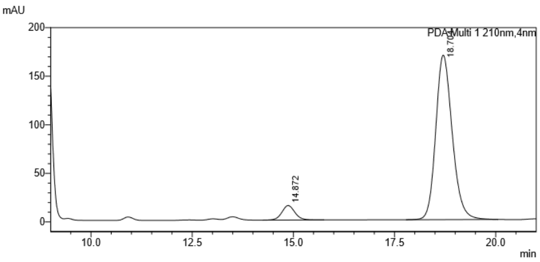

<Peak Table>
PDA Ch1 210nm

Peak#	Ret. Time	Area	Area%	Height	Height%
1	14.872	333072	6.477	14901	8.088
2	18.704	4809146	93.523	169338	91.912
Total		5142218	100.000	184239	100.000

5.2.2 Methyl (*E*)-4-(phenylsulfonamido)hex-2-enoate (7bo).

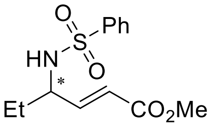

7bo

The general procedure was followed using $PhSO_2NH_2$ (0.10 mmol) and **2o** (0.20 mmol). After purification by column chromatography (PE/EtOAc = 2:1), **7bo** (17.6 mg, 62%) was obtained. $[\alpha]^{22}_D$ = 33.3 (*c* 0.33, $CHCl_3$); **^{1}H NMR** (500 MHz, $CDCl_3$) δ 7.88 – 7.84 (m, 2H), 7.58 – 7.54 (m, 1H), 7.53 – 7.47 (m, 2H), 6.60 (dd, *J* = 15.5, 6.5 Hz, 1H), 5.81 – 5.74 (m, 1H), 5.15 – 5.05 (m, 1H), 3.91 – 3.83 (m, 1H), 3.68 (s, 3H), 1.59 – 1.50 (m, 2H), 0.83 (t, *J* = 7.5 Hz, 3H); **^{13}C NMR** (125 MHz, $CDCl_3$) δ 166.26, 146.65, 140.65, 132.74, 129.11, 127.07, 121.75, 56.10, 51.67, 28.09, 9.71. Enantiomeric excess: 86%, determined by HPLC (Chiralpak IF hexane/*i*-PrOH = 75/25; flow rate 0.7 ml/min; 25 ℃; 254 nm), first peak: tR = 13.7 min, second peak: tR = 17.1 min. HRMS (ESI) calcd. for $C_{13}H_{17}NNaO_4S$ $[M+Na]^+$: 306.0770, found: 306.0764.

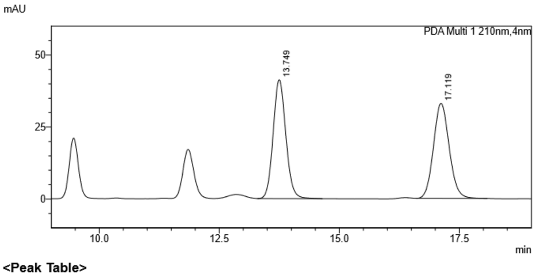

<Peak Table>
PDA Ch1 210nm

Peak#	Ret. Time	Area	Area%	Height	Height%
1	13.749	760149	50.281	41259	55.553
2	17.119	751642	49.719	33010	44.447
Total		1511791	100.000	74269	100.000

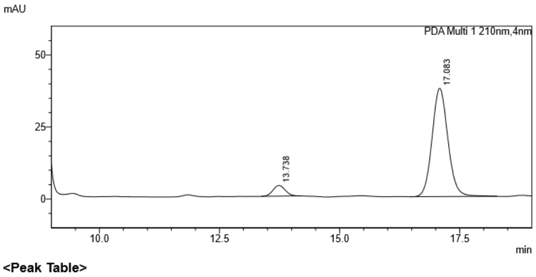

<Peak Table>
PDA Ch1 210nm

Peak#	Ret. Time	Area	Area%	Height	Height%
1	13.738	66488	7.164	3726	9.027
2	17.083	861647	92.836	37550	90.973
Total		928135	100.000	41276	100.000

5.3 General procedure for $(BocNH)_2$.

Di-tert-butyl (*E*)-1-(6-(benzyloxy)-6-oxohex-4-en-3-yl)hydrazine-1,2-dicarboxylate (9).

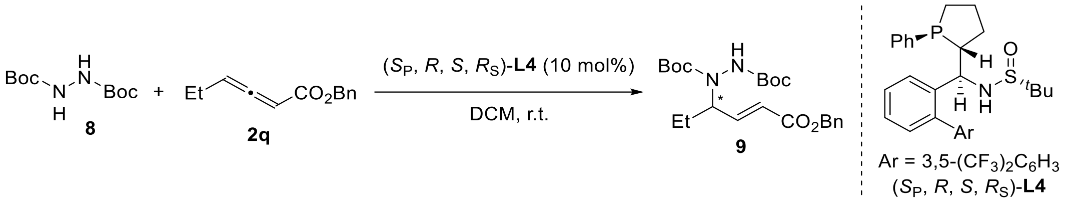

To a flame-dried glass tube with a magnetic stirring bar were added $(BocNH)_2$ **8** (23.2 mg, 0.10 mmol) and (S_P, *R*, *S*, R_S)-**L4** (5.9 mg, 0.01 mmol), followed by the addition of dry DCM (1.5 mL).[5] Then the allenoate **2q** (40.4 mg, 0.20 mmol) was slowly added via syringe at room temperature under inert atmosphere. The reaction mixture was stirred for 48 h, and TLC show that the rection was completed. Then DCM was removed under reduced pressure. The residue was purified by column chromatography on silica gel (petroleum ether/ethyl acetate = 5:1) to afford **9** (38.2 mg, 88% yield). $[\alpha]^{22}_D = 6.44$ (*c* 0.33, $CHCl_3$); **^{1}H NMR** (300 MHz, $CDCl_3$) δ 7.39 – 7.32 (m, 5H), 6.93 (dd, *J* = 15.6, 6.6 Hz, 1H), 6.24 (s, 1H), 5.96 (d, *J* = 15.6 Hz, 1H), 5.18 (s, 2H), 4.69 (s, 1H), 1.52 – 1.36 (m, 20H), 0.99 – 0.88 (m, 3H); **^{13}C NMR** (125 MHz, $CDCl_3$) δ 166.11, 154.77, 146.36, 135.91, 128.41 (d, *J* = 34.1 Hz), 122.17, 81.66, 66.32, 28.20, 24.34, 10.78. Enantiomeric excess: 83%, determined by HPLC (Chiralpak IF hexane/*i*-PrOH = 85/15; flow rate 0.8 ml/min; 25 ℃; 254 nm), first peak: tR = 16.8 min, second peak: tR = 18.7 min. HRMS (ESI) calcd. for $C_{23}H_{34}N_2NaO_6$ $[M+Na]^+$: 457.2309, found: 457.2299.

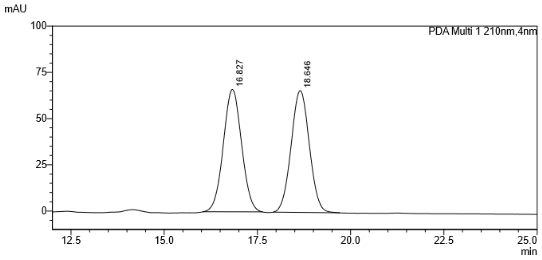

<Peak Table>

PDA Ch1 210nm

Peak#	Ret. Time	Area	Area%	Height	Height%
1	16.827	2259479	50.204	66255	50.142
2	18.646	2241160	49.796	65880	49.858
Total		4500640	100.000	132135	100.000

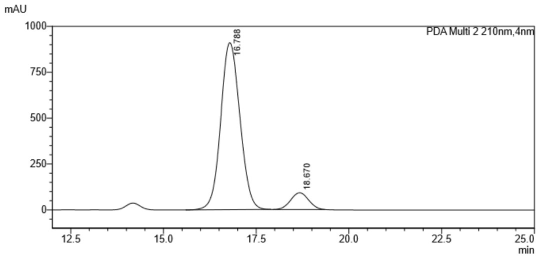

<Peak Table>

PDA Ch2 210nm

Peak#	Ret. Time	Area	Area%	Height	Height%
1	16.788	32841497	91.429	910554	90.914
2	18.670	3078839	8.571	91005	9.086
Total		35920337	100.000	1001559	100.000

5.4 General procedure for pyrazole.

Ethyl (*E*)-4-(1H-pyrazol-1-yl)pent-2-enoate

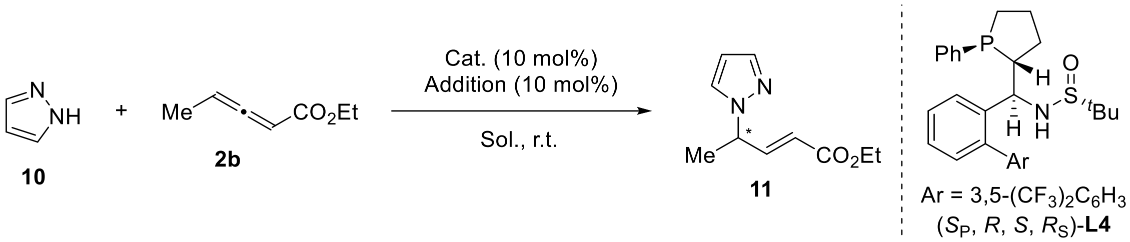

To a flame-dried glass tube with a magnetic stirring bar were added pyrazole **10** (6.8 mg, 0.10 mmol) and (S_P, R, S, R_S)-**L4** (5.9 mg, 0.01 mmol), followed by the addition of dry diaxane (1.5 mL) and CH_3COOH (0.60 mg, 0.01 mmol). Then the allenoate **2b** (37.8 mg, 0.30 mmol) was slowly added via syringe at room temperature under inert atmosphere. The reaction mixture was stirred for 24 h, and TLC show that the rection was completed. Then dioxane was removed under reduced pressure. The residue was purified by column chromatography on silica gel (petroleum ether/ethyl acetate = 2:1) to afford **11** (16.5 mg, 85% yield). $[\alpha]^{22}_D$ = -1.44 (c 0.50, $CHCl_3$); ^{1}H NMR (300 MHz, $CDCl_3$) δ 7.54 (d, J = 1.5 Hz, 1H), 7.42 (d, J = 2.1 Hz, 1H), 7.04 (dd, J = 15.6, 5.4 Hz, 1H), 6.29 (t, J = 2.1 Hz, 1H), 5.64 (dd, J = 15.6, 1.5 Hz, 1H), 5.18 – 5.01 (m, 1H), 4.16 (q, J = 7.2 Hz, 2H), 1.69 (d, J = 6.9 Hz, 3H), 1.25 (t, J = 7.2 Hz, 3H); ^{13}C NMR (100 MHz, $CDCl_3$) δ 165.79, 146.90, 139.39, 127.32, 121.82, 105.69, 60.52, 57.79, 19.58, 14.06; Enantiomeric excess: 95%, determined by HPLC (Chiralpak ODH, hexane/i-PrOH = 90/10; flow rate 0.8 ml/min; 25 ℃; 210 nm), first peak: tR = 10.6 min, second peak: tR = 11.9 min.

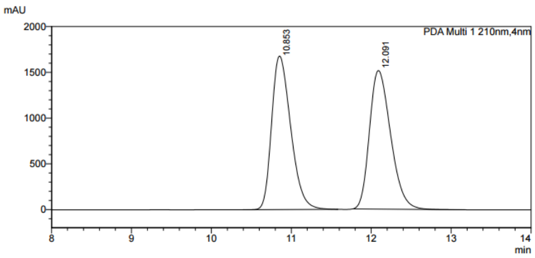

<Peak Table>

PDA Ch1 210nm

Peak#	Ret. Time	Area	Area%	Height	Height%
1	10.853	27924162	49.961	1677730	52.549
2	12.091	27968019	50.039	1514938	47.451
Total		55892181	100.000	3192668	100.000

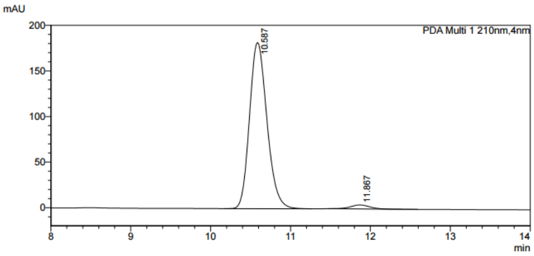

<Peak Table>

PDA Ch1 210nm

Peak#	Ret. Time	Area	Area%	Height	Height%
1	10.587	2766079	97.415	182049	97.699
2	11.867	73407	2.585	4288	2.301
Total		2839486	100.000	186336	100.000

5. Experimental procedure general datum and HPLC spectra for the transformations of 3ga, 6aa and 6lo.

6.1 Hydrolysis of 3ga:[6]

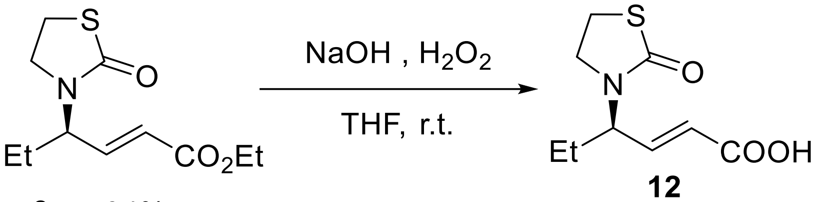

3ga, 94% *ee* → **12**, 73% yield, 94% *ee*

30% H_2O_2 (0.10 mL) and aqoeous NaOH (0.40 mmol, 0.50 mL H_2O) were added to a solution of **3ga** (49.0 mg, 0.20 mmol) in THF (1.0 mL) at 0 ℃. The reaction mixture was stirred at room temperature for 2 h until the disappearance of **3ga** as indicated by TLC, quenched with saturated sodium thiosulfate solution (5.0 mL), stirred for another 15 min, acidified with 2 N HCl, extracted with EtOAc (3 × 5 mL), dried over Na_2SO_4, filtered, concentrated, and purified by flash chromatography (silica gel, DCM/MeOH = 10/1 as eluent) to give acid **12** (31.3 mg, 73%). $[\alpha]^{22}_D$ = -2.6 (*c* 0.25, acetone); ^{1}H NMR (400 MHz, $CDCl_3$) δ 10.05 (s, 1H), 6.91 (dd, *J* = 16.0, 5.6 Hz, 1H), 5.91 (dd, *J* = 15.6, 0.8 Hz, 1H), 4.75 – 4.66 (m, 1H), 3.54 (t, *J* = 7.2 Hz, 2H), 3.37 – 3.21 (m, 2H), 1.81 – 1.60 (m, 2H), 0.95 (t, *J* = 7.2 Hz, 3H); ^{13}C NMR (101 MHz, $CDCl_3$) δ 172.70, 170.45, 147.04, 122.31, 55.53, 44.23, 26.06, 24.20, 10.60; Enantiomeric excess (determined by transformed to methyl ester) : 94%, determined by HPLC (Chiralpak IF hexane/*i*-PrOH = 85/15; flow rate 0.6 ml/min; 25 ℃; 254 nm), first peak: t_R = 32.0 min, second peak: t_R = 36.5 min. HRMS (ESI) calcd. for $C_9H_{13}NNaO_3S$ $[M+Na]^+$: 238.0508, found: 238.0503.

<Chromatogram>

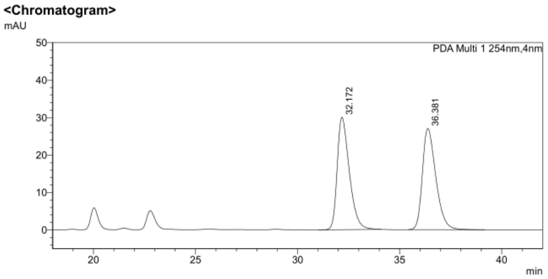

<Peak Table>

PDA Ch1 254nm

Peak#	Ret. Time	Area	Area%	Height	Height%
1	32.172	1241944	50.020	30029	52.608
2	36.381	1240962	49.980	27052	47.392
Total		2482906	100.000	57081	100.000

<Chromatogram>

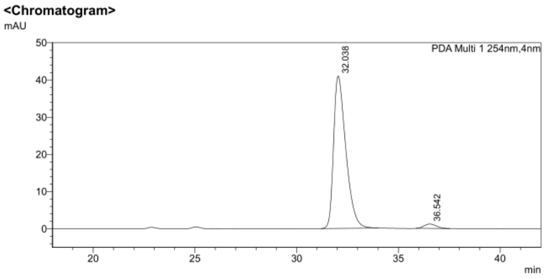

<Peak Table>

PDA Ch1 254nm

Peak#	Ret. Time	Area	Area%	Height	Height%
1	32.038	1719002	97.185	40992	97.196
2	36.542	49787	2.815	1182	2.804
Total		1768789	100.000	42174	100.000

6.2 Synthesis of 13:

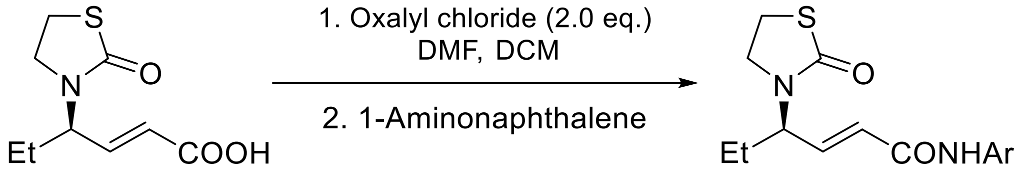

12, 94% *ee*

13
Ar = 1-naphthyl
94% yield, 95% *ee*

A dichloromethane solution of **12** (43.0 mg, 0.20 mmol) and N,N-Dimethylformamide (10 µL) was stirred at room temperature. Oxalyl chloride (34 µL, 0.40 mmol) was slowly added to the solution and stirred for 1h until the disappearance of **12** as indicated by TLC. The solvent was removed under reduced pressure. The crude product was dissolved by dichloromethane, and a dichloromethane solution of 1-aminonaphthalene (43 mg, 0.30 mmol) was added and the mixture was stirred for another 2h. The solvent was removed under reduced pressure and the resulting solid was purified by flash column chromatography in petroleum ether/EtOAc (3:1) to afford **13** (64 mg, 94%) as a white solid. $[\alpha]^{22}_{D}$ = -3.0 (*c* 0.25, acetone); ^{1}H NMR (400 MHz, $CDCl_3$) δ 8.66 (s, 1H), 7.93 (dd, *J* = 6.4, 3.6 Hz, 1H), 7.91 – 7.86 (m, 1H), 7.83 (s, 1H), 7.67 (d, *J* = 8.0 Hz, 1H), 7.49 – 7.40 (m, 3H), 6.82 (dd, *J* = 15.6, 6.4 Hz, 1H), 6.35 (d, *J* = 15.2 Hz, 1H), 4.68 (d, *J* = 6.0 Hz, 1H), 3.57 – 3.39 (m, 2H), 3.32 – 3.13 (m, 2H), 1.65 – 1.52 (m, 2H), 0.86 (t, *J* = 6.8 Hz, 4H); ^{13}C NMR (100 MHz, $CDCl_3$) δ 172.71, 164.20, 141.26, 134.10, 132.53, 128.53, 127.45, 126.10 (d, *J* = 20.3 Hz), 125.64, 121.40 (d, *J* = 24.4 Hz), 55.88, 44.07, 26.16, 24.85, 10.65; Enantiomeric excess: 95%, determined by HPLC (Chiralpak IF hexane/*i*-PrOH = 60/40; flow rate 0.8 ml/min; 25 ℃; 190 nm), first peak: t_R = 10.8 min, second peak: t_R = 13.7 min. HRMS (ESI) calcd. for $C_{19}H_{20}N_2NaO_2S$ $[M+Na]^+$: 363.1138, found: 363.1142.

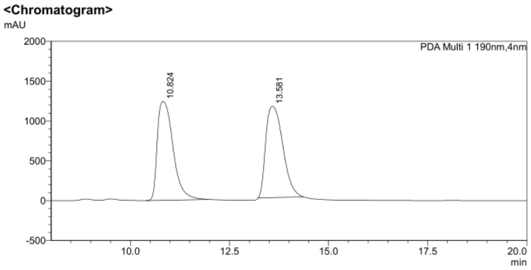

<Peak Table>

PDA Ch1 190nm

Peak#	Ret. Time	Area	Area%	Height	Height%
1	10.824	34362921	49.973	1242494	52.003
2	13.581	34400234	50.027	1146777	47.997
Total		68763155	100.000	2389271	100.000

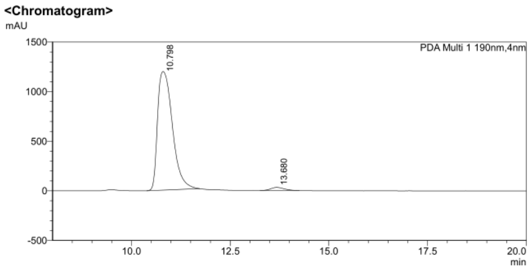

<Peak Table>

PDA Ch1 190nm

Peak#	Ret. Time	Area	Area%	Height	Height%
1	10.798	31311924	97.599	1195936	97.427
2	13.680	770151	2.401	31580	2.573
Total		32082075	100.000	1227516	100.000

6.3 Synthesis of 14:[7]

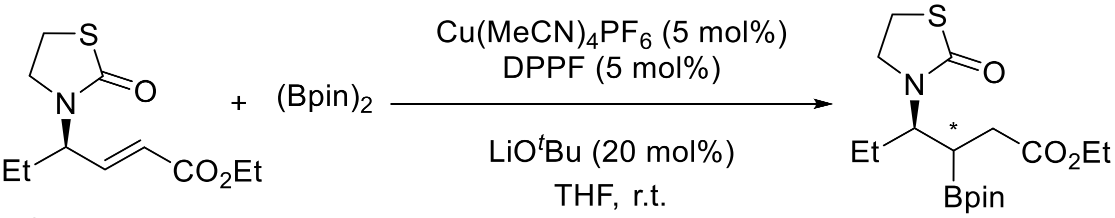

3ga, 97% *ee*

14
94% yield, 98% *ee*
dr = 5:1

A solution of $Cu(MeCN)_4PF_6$ (4.0 mg, 0.01 mmol) and DPPF (5.0 mg, 0.01 mmol) in THF (1.0 mL) was stirred at room temperature for 30 minutes, **3ga** (49.0 mg, 0.20 mmol) was slowly added to the reaction followed by $(Bpin)_2$ (76.0 mg, 0.30 mmol) and the mixture stirred for an additional 30 minutes. Then 0.04 mmol lithium *tert*-butoxide (1.0 M in hexane) was added and the solution was stirred for another 2h. The solvent was removed under reduced pressure and the resulting solid was purified by flash column chromatography in petroleum ether/EtOAc (5:1) to afford **14** (46.0 mg, 94%, *dr* = 5:1) as a colorless oil. $[\alpha]^{22}_{D}$ = 27.9 (*c* 0.25, acetone); ^1H NMR (400 MHz, $CDCl_3$) δ 4.12 – 4.03 (m, 2H), 4.04 – 3.97 (m, 1H), 3.78 – 3.71 (m, 1H), 3.45 – 3.36 (m, 1H), 3.31 – 3.22 (m, 1H), 3.16 – 3.09 (m, 1H), 2.52 – 2.34 (m, 2H), 1.72 – 1.61 (m, 1H), 1.61 – 1.53 (m, 1H), 1.44 – 1.34 (m, 1H), 1.23 – 1.18 (m, 15H), 0.82 (t, *J* = 7.2 Hz, 3H); ^{13}C NMR (100 MHz, $CDCl_3$) δ 173.58, 171.85, 83.50, 60.48, 56.27, 44.01, 33.90, 25.97, 24.95, 24.80, 24.53, 14.23, 10.69; Enantiomeric excess: 98%, determined by HPLC (Chiralpak IC, hexane/i-PrOH = 93/7; flow rate 0.8 ml/min; 25 ℃; 210 nm), first peak: t_R = 38.4 min, second peak: t_R = 41.2 min, third peak: t_R =

50.1 min. HRMS (ESI) calcd. For $C_{17}H_{30}BNNaO_5S$ $[M+Na]^+$: 394.1833, found: 394.1829.

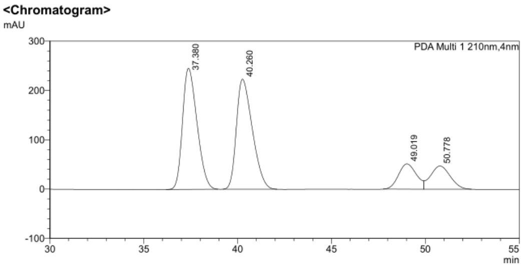

<Peak Table>
PDA Ch1 210nm

Peak#	Ret. Time	Area	Area%	Height	Height%
1	37.380	13205063	39.748	245582	43.319
2	40.260	13181797	39.678	223149	39.362
3	49.019	3400295	10.235	51234	9.037
4	50.778	3434851	10.339	46945	8.281
Total		33222006	100.000	566911	100.000

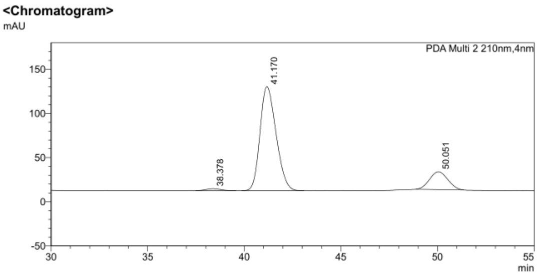

<Peak Table>
PDA Ch2 210nm

Peak#	Ret. Time	Area	Height	Area%	Conc.	Height%
1	38.378	119487	2253	1.445	0.000	1.607
2	41.170	6830545	117809	82.597	0.000	84.035
3	50.051	1319675	20129	15.958	0.000	14.358
Total		8269706	140191	100.000		100.000

6.4 Reduction of the double bond of 3aa:

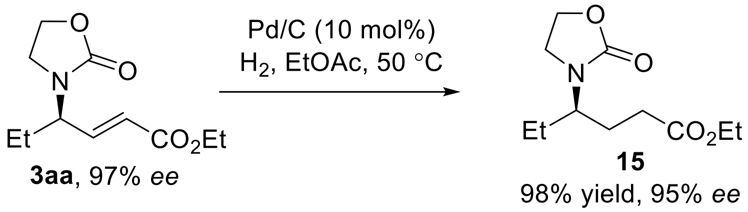

3aa, 97% *ee*

15

98% yield, 95% *ee*

A suspension of **3aa** (22.7 mg, 0.10 mmol) and 10% palladium on carbon (15.5 mg) in EtOAc (2.0 mL) was maintained under an atmosphere of hydrogen gas for 8 h at 50 °C. The insoluble solids were removed by filtration and the filtrate was concentrated to provide product **15** (22.5 mg, 98%) without any purification. $[\alpha]^{22}_D$ = 1.04 (*c* 0.50, $CHCl_3$); ^1H NMR (300 MHz, $CDCl_3$) δ 4.34 – 4.17 (m, 2H), 4.04 (q, *J* = 6.9 Hz, 2H), 3.70 – 3.54 (m, 1H), 3.46 – 3.27 (m, 2H), 2.36 – 2.13 (m, 2H), 1.82 – 1.63 (m, 2H), 1.56 – 1.34 (m, 2H), 1.17 (t, *J* = 7.2 Hz, 3H), 0.83 (t, *J* = 7.2 Hz, 3H); ^{13}C NMR (100 MHz, $CDCl_3$) δ 172.94, 158.46, 61.87, 60.35, 54.67, 39.24, 31.08, 27.02, 25.27, 13.99, 10.54; Enantiomeric excess: 95%, determined by HPLC (Chiralpak IF, hexane/*i*-PrOH = 80/20; flow rate 0.7 ml/min; 25 °C; 210 nm), first peak: t_R = 18.1 min, second peak: t_R = 19.3 min. HRMS (ESI) calcd. For $C_{11}H_{19}NNaO_4$ $[M+Na]^+$: 252.1209, found: 252.1206.

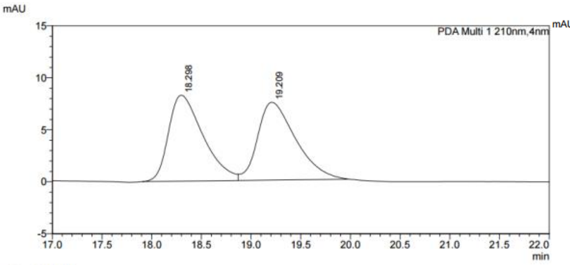

<Peak Table>
PDA Ch1 210nm

Peak#	Ret. Time	Area	Area%	Height	Height%
1	18.298	200357	50.083	8271	52.464
2	19.209	199693	49.917	7494	47.536
Total		400050	100.000	15765	100.000

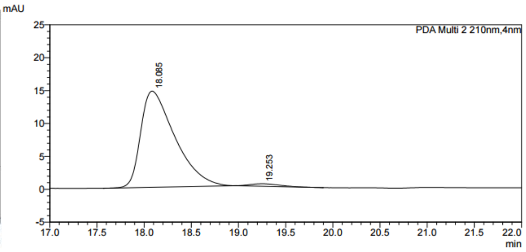

<Peak Table>
PDA Ch2 210nm

Peak#	Ret. Time	Area	Area%	Height	Height%
1	18.085	369763	97.774	14613	97.428
2	19.253	8419	2.226	386	2.572
Total		378182	100.000	14999	100.000

6.5 Ring opening of 15:

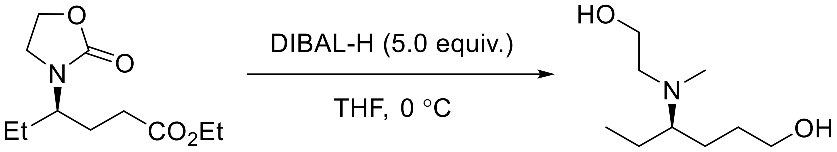

To a flame-dried glass tube with a magnetic stirring bar was added **15** (229.2 mg, 1.0 mmol) in THF (3.0 mL), stirring at 0 ℃ for 10 minutes. DIBAL-H (5.0 mmol, 1.5 mol/L in toluene) was slowly added to the reaction and the solution was stirred for another 1h at the same temperature. Potassium sodium tartrate saturated solution was added to the solution and the reaction mixture was stirred overnight, extracted with EtOAc (3 × 10 mL), dried over Na_2SO_4, filtered, concentrated, and purified by flash chromatography (silica gel, DCM/MeOH = 10/1 as eluent) to give **16** (131.5 mg, 75%). $[\alpha]^{22}_D$ = -22.10 (*c* 0.20, $CHCl_3$); ^1H NMR (300 MHz, $CDCl_3$) δ 5.16 (s, 2H), 3.83 – 3.49 (m, 4H), 2.93 – 2.77 (m, 1H), 2.76 – 2.62 (m, 1H), 2.62 – 2.47 (m, 1H), 2.41 (s, 3H), 1.88 – 1.49 (m, 5H), 1.31 – 1.20 (m, 1H), 0.97 (t, *J* = 7.4 Hz, 3H); ^{13}C NMR (101 MHz, $CDCl_3$) δ 66.07, 61.43, 57.17, 54.63, 35.59, 29.94, 27.29, 20.17, 10.89; HRMS (ESI) calcd. For $C_9H_{22}NO_2$ $[M+H]^+$: 176.1645, found: 176.1644.

6.6 Synthesis of 17:

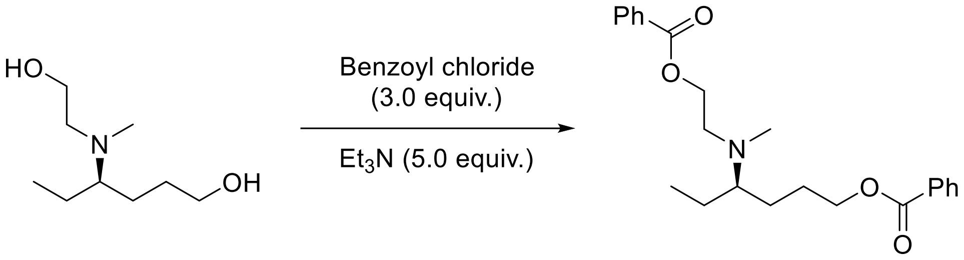

A dichloromethane solution of **16** (87.6 mg, 0.50 mmol) and 0.3 mL Et_3N was stirred at room temperature. Benzoyl chloride (210.9 mg, 1.50 mmol) was slowly added to the solution and stirred for 30 minutes until the disappearance of **16** as indicated by TLC. The solvent was removed under reduced pressure and the resulting solid was purified by flash column chromatography in petroleum ether/EtOAc (2:1) to afford **17** (153.4 mg, 80%). $[\alpha]^{22}_D$ = -8.23 (*c* 0.40, $CHCl_3$); ^1H NMR (400 MHz, $CDCl_3$) δ 8.06 – 7.99 (m, 4H), 7.57 – 7.48 (m, 2H), 7.41 (dd, *J* = 16.8, 7.8 Hz, 4H), 4.37 (t, *J* = 6.0 Hz, 2H), 4.27 (t, *J* = 6.8 Hz, 2H), 2.90 – 2.75 (m, 2H), 2.40 (dt, *J* = 13.6, 6.4 Hz, 1H), 2.32 (s, 3H), 1.92 – 1.71 (m, 2H), 1.55 – 1.45 (m, 2H), 1.33 – 1.20 (m, 2H), 0.90 (t, *J* = 7.2 Hz, 3H); ^{13}C NMR (101 MHz, $CDCl_3$) δ 166.65, 132.79 (d, *J* = 3.8 Hz), 130.49 (d, *J* = 10.5 Hz), 129.54 (d, *J* = 3.3 Hz), 128.31, 65.47, 65.21, 63.55, 52.31, 37.19, 26.68, 26.33, 22.44, 11.90; Enantiomeric excess: 92%, determined by HPLC (Chiralpak IF, hexane/*i*-PrOH = 98/02; flow rate 1.0 ml/min; 25 ℃; 210 nm), first peak: t_R = 13.2 min, second peak: t_R = 13.8 min. HRMS (ESI) calcd. For $C_{23}H_{30}NO_4$ $[M+H]^+$: 384.2169, found: 384.2170.

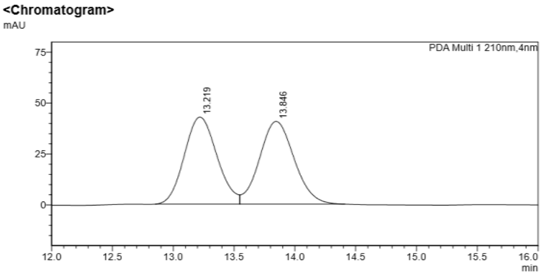

<Peak Table>
PDA Ch1 210nm

Peak#	Ret. Time	Area	Area%	Height	Height%
1	13.219	800287	49.768	42716	51.237
2	13.846	807751	50.232	40653	48.763
Total		1608039	100.000	83370	100.000

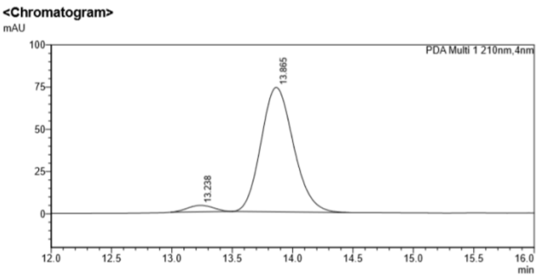

<Peak Table>
PDA Ch1 210nm

Peak#	Ret. Time	Area	Area%	Height	Height%
1	13.238	57120	3.975	3788	4.889
2	13.865	1379698	96.025	73699	95.111
Total		1436818	100.000	77487	100.000

6.7 Selective epoxidation of 6aa:[8]

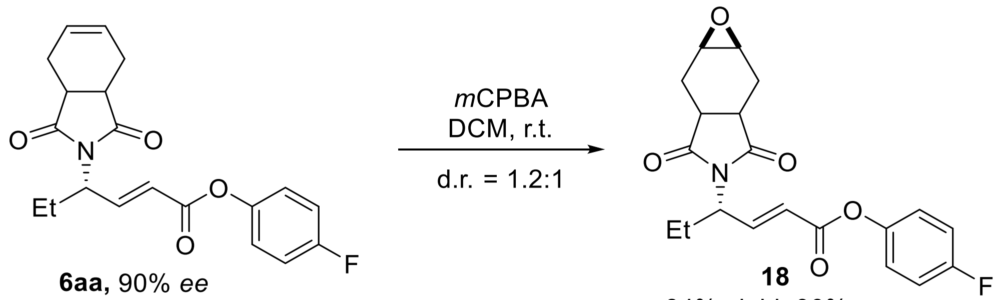

6aa, 90% *ee*

18 84% yield, 90% *ee*

A dichloromethane solution of **6aa** (50 mg; 0.14 mmol) and m-chloroperbenzoic acid (48 mg; 0.28 mmol) was stirred at room temperature for 4h until the disappearance of **6aa** as indicated by TLC. Saturated sodium thiosulfate solution (5 mL) was added and the mixture was stirred for an additional 15 minutes. The organic layer was separated and the aqueous phase was extracted with dichloromethane (3x10 mL). Combined organic layers were washed with saturated sodium bicarbonate, followed by brine, and dried over anhydrous Na_2SO_4. The solvent was removed under reduced pressure and the resulting solid was purified by flash column chromatography in petroleum ether/EtOAc (3:1) to give **18** (84%, 90% *ee*) of white product. $[\alpha]^{22}_{D}$ = 6.0 (*c* 0.25, acetone); ^{1}H NMR (400 MHz, $CDCl_3$) δ 7.25 (dd, *J* = 16.0, 6.4 Hz, 1H), 7.06 (d, *J* = 6.4 Hz, 4H), 6.04 (dd, *J* = 15.6, 0.8 Hz, 1H), 4.74 – 4.66 (m, 1H), 3.27 (s, 2H), 3.02 – 2.92 (m, 2H), 2.70 – 2.56 (m, 2H), 2.11 – 2.03 (m, 1H), 2.00 – 1.88 (m, 3H), 1.62 (d, *J* = 6.0 Hz, 1H), 0.89 (t, *J* = 7.6 Hz, 3H); ^{13}C NMR (100 MHz, $CDCl_3$) δ 179.09, 164.08, 161.45, 159.02, 146.29 (d, *J* = 2.8 Hz), 145.87, 122.87 (d, *J* = 8.4 Hz), 122.41, 116.18, 115.94, 53.71, 49.00, 35.14, 23.85, 23.20, 10.69; Enantiomeric excess: 90%, determined by HPLC (Chiralpak AD-3, hexane/*i*-PrOH = 70/30; flow rate 0.8 ml/min; 25 ℃; 254 nm), first peak: t_R = 23.5 min, second peak: t_R = 35.1 min. HRMS (ESI) calcd. For $C_{20}H_{20}FNNaO_5$ $[M+Na]^+$: 396.1218, found: 396.1224.

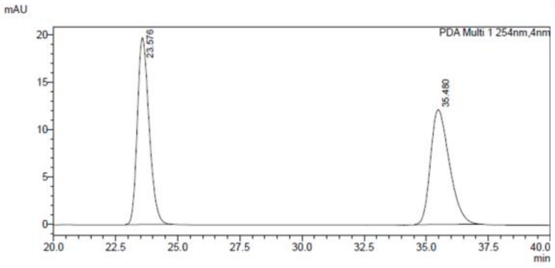

PDA Ch1 254nm

Peak#	Ret. Time	Area	Area%	Height	Height%
1	23.576	651758	50.343	19772	61.969
2	35.480	642888	49.657	12134	38.031
Total		1294646	100.000	31905	100.000

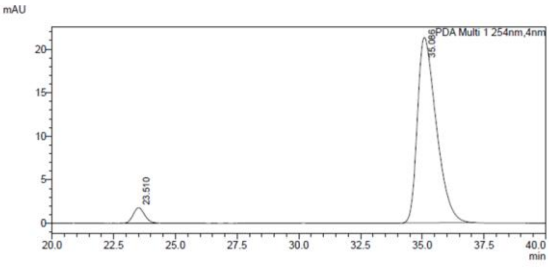

PDA Ch1 254nm

Peak#	Ret. Time	Area	Area%	Height	Height%
1	23.510	56176	4.706	1763	7.647
2	35.086	1137548	95.294	21295	92.353
Total		1193724	100.000	23058	100.000

6.8 Synthesis of 19:[9]

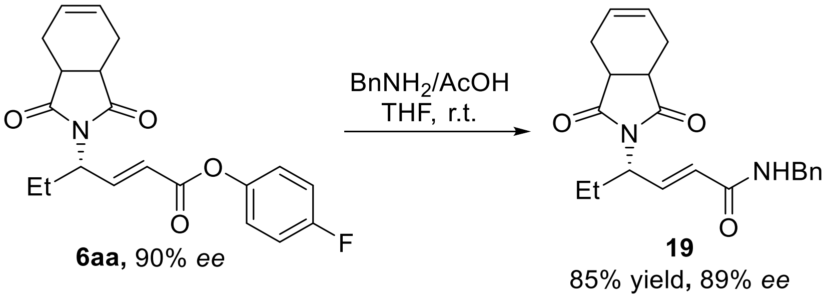

BnNH$_2$ (75 mg, 0.7 mmol) and AcOH (42 mg, 0.7 mmol) were added to a solution of **6aa** (50 mg, 0.14 mmol) in THF (1.0 mL). The reaction mixture was stirred at room temperature for 8 h until the disappearance of **6aa** as indicated by TLC, concentrated, and purified by flash chromatography (silica gel, petroleum ether /EtOAc = 2/1 as eluent) to give acid **19** (85%, 89% *ee*). $[\alpha]^{22}_D$ = -0.8 (*c* 0.25, acetone); ^{1}H NMR (400 MHz, CDCl$_3$) δ 7.35 – 7.24 (m, 5H), 6.93 (dd, *J* = 15.6, 7.2 Hz, 1H), 6.09 (s, 1H), 5.93 – 5.88 (m, 2H), 5.86 (d, *J* = 15.6 Hz, 1H), 4.56 (dd, *J* = 16.0, 7.2 Hz, 1H), 4.46 (d, *J* = 5.6 Hz, 2H), 3.08 – 2.98 (m, 2H), 2.58 (d, *J* = 14.8 Hz, 2H), 2.19 (dd, *J* = 14.8, 6.8 Hz, 2H), 2.11 – 1.96 (m, 1H), 1.86 – 1.72 (m, 1H), 0.79 (t, *J* = 7.6 Hz, 3H); ^{13}C NMR (100 MHz, CDCl$_3$) δ 179.67 (d, *J* = 1.6 Hz), 164.85, 139.62, 137.97, 128.63, 127.85, 127.48, 125.46, 54.36, 43.62, 38.83 (d, *J* = 16.3 Hz), 23.80, 23.53 (d, *J* = 2.3 Hz), 10.65; Enantiomeric excess: 89%, determined by HPLC (Chiralpak IC, hexane/*i*-PrOH = 70/30; flow rate 0.8 ml/min; 25 ℃; 254 nm), first peak: tR = 20.7 min, second peak: tR = 27.0 min. HRMS (ESI) calcd. For $C_{21}H_{24}N_2NaO_3$ [M+Na]$^+$: 375.1679, found: 375.1677.

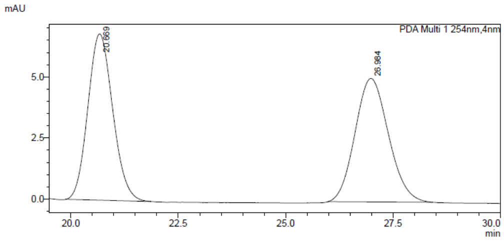

<Peak Table>

PDA Ch1 254nm

Peak#	Ret. Time	Area	Height	Conc.	Unit	Mark	Name
1	20.669	274273	6807	0.000			
2	26.984	273618	5051	0.000			
Total		547890	11857				

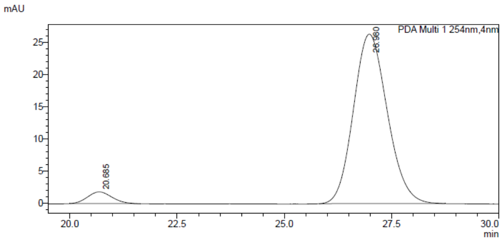

<Peak Table>

PDA Ch1 254nm

Peak#	Ret. Time	Area	Area%	Height	Height%
1	20.685	72309	4.754	1822	6.466
2	26.980	1448713	95.246	26349	93.534
Total		1521022	100.000	28171	100.000

6.9 Reduction of the double bond of 6lo:

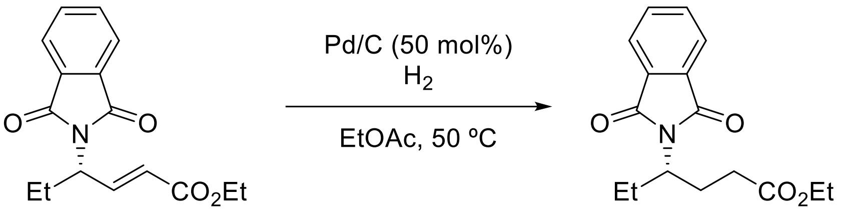

6lo, 88% *ee*   **20**, 96%, 88% *ee*

A suspension of **6lo** (143 mg, 0.50 mmol) and 50% palladium on carbon (70 mg) in EtOAc (10 mL) was maintained under an atmosphere of hydrogen gas for 8 h at 50 °C. The insoluble solids were removed by filtration and the filtrate was concentrated to provide product **20** (136.0 mg, 96%) without any purification. $[\alpha]^{22}_{D}$ = -4.4 (*c* 0.20, $CHCl_3$); ^1H NMR (500 MHz, $CDCl_3$) δ 7.86 – 7.77 (m, 2H), 7.87 – 7.77 (m, 2H), 7.74 – 7.68 (m, 2H), 7.74 – 7.69 (m, 2H), 4.13 (dq, *J* = 15.5, 5.0 Hz, 1H), 4.08 – 3.98 (m, 2H), 2.46 – 2.33 (m, 1H), 2.31 – 2.22 (m, 2H), 2.14 – 2.03 (m, 2H), 1.85 – 1.74 (m, 1H), 1.18 (t, *J* = 7.1 Hz, 3H), 0.86 (t, *J* = 7.4 Hz, 3H); ^{13}C NMR (126 MHz, $CDCl_3$) δ 172.69, 168.62, 133.94, 131.73, 123.14, 60.44, 53.16, 31.50, 27.32, 25.44, 14.11, 11.02; Enantiomeric excess: 88%, determined by HPLC (Chiralpak AD-H, hexane/*i*-PrOH = 95/05; flow rate 0.8 ml/min; 25 ℃; 210 nm), first peak: t_R = 12.4 min, second peak: t_R = 13.0 min. HRMS (ESI) calcd. For $C_{16}H_{19}NNaO_4$ $[M+Na]^+$: 312.1206, found: 312.1210.

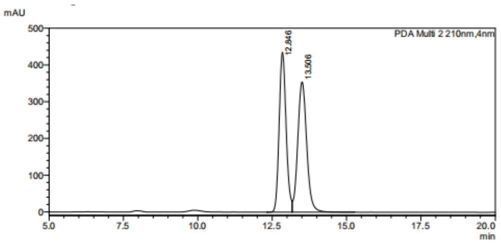

<Peak Table>

PDA Ch2 210nm

Peak#	Ret. Time	Area	Area%	Height	Height%
1	12.846	7089985	48.829	435074	55.101
2	13.506	7430165	51.171	354526	44.899
Total		14520150	100.000	789600	100.000

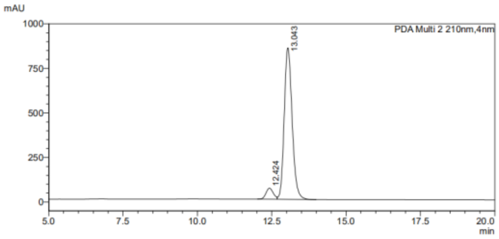

<Peak Table>

PDA Ch2 210nm

Peak#	Ret. Time	Area	Area%	Height	Height%
1	12.424	1044851	6.207	61478	6.739
2	13.043	15788290	93.793	850830	93.261
Total		16833140	100.000	912308	100.000

6.10 Acidic deprotection of 20:[10]

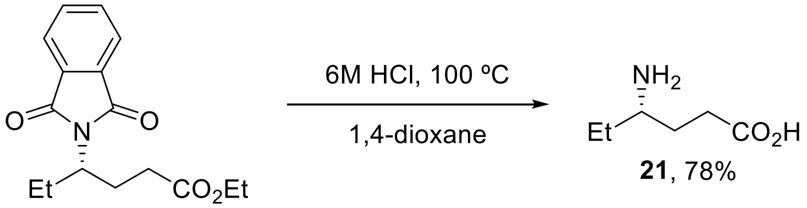

20   **21**, 78%

A solution of compound **20** (136 mg, 0.47 mmol) in dioxane (1 mL) and 6 M HCl (6 mL) is refluxed for 12 h. After cooling to room temperature, the mixture is washed with EtOAc. The aqueous phase is concentrated in vacuo to afford the product **21** (48.1 mg, 78%). $[\alpha]^{22}_{D}$ = -3.7 (*c* 0.10, acetone); ^{1}H NMR (400 MHz, D_2O) δ 3.27 (p, *J* = 6.4 Hz, 1H), 2.53 (t, *J* = 7.6 Hz, 2H), 2.02 – 1.86 (m, 2H), 1.79 – 1.68 (m, 1H), 1.68 – 1.58 (m, 1H), 0.97 (t, *J* = 7.6 Hz, 3H); ^{13}C NMR (101 MHz, D_2O) δ 177.07, 52.46, 29.63, 26.56, 24.71, 8.51. HRMS (ESI) calcd. For $C_6H_{14}NO_2$ $[M+H]^+$: 132.1019, found: 132.1015.

6.8 Synthesis of 22:[11]

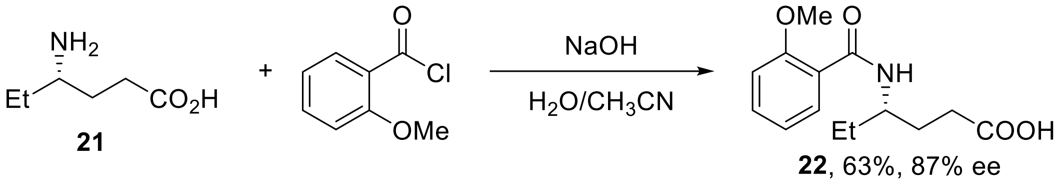

To a vigorously stirred solution containing amino acid **21** (26 mg, 0.2 mmol) and NaOH (0.8 mmol) in 2 mL H_2O/CH_3CN (3:1) at 0 ℃ was added *o*-methoxybenzoyl chloride (0.24 mmol). The reaction was allowed to warm to room temperature and then proceeded for 4 h, then cooled to 0 ℃ and acidified to pH = 1-2 with 2 N HCl, and was extracted into EtOAc (3×10 mL), dried over Na_2SO_4, filtered, concentrated, and purified by flash chromatography (silica gel, DCM/MeOH = 20/1 as eluent) to give **22** (32.9 mg, 63%). $[\alpha]^{22}_{D}$ = -5.0 (*c* 0.20, acetone); ^{1}H NMR (400 MHz, $CDCl_3$) δ 8.26 – 8.08 (m, 1H), 7.75 (d, *J* = 8.4 Hz, 1H), 7.47 – 7.41 (m, 1H), 7.10 – 7.04 (m, 1H), 6.97 (d, *J* = 8.4 Hz, 1H), 4.18 – 4.05 (m, 1H), 3.96 (s, 3H), 2.43 (t, *J* = 7.6 Hz, 2H), 2.02 – 1.92 (m, 1H), 1.81 – 1.71 (m, 1H), 1.71 – 1.61 (m, 1H), 1.59 – 1.47 (m, 1H), 0.97 (t, *J* = 7.6 Hz, 3H); ^{13}C NMR (101 MHz, $CDCl_3$) δ (101 MHz, CDCl3) δ 177.39, 165.94, 157.51, 132.96, 132.37, 121.42, 121.22, 111.39, 56.03, 56.00, 50.56, 31.49, 30.57, 28.27, 10.20; Enantiomeric excess: 87%, determined by HPLC (Chiralpak AD-H, hexane/*i*-PrOH = 85/15; flow rate 0.8 ml/min; 25 ℃; 254 nm), first peak: t_R = 23.5 min, second peak: t_R = 28.6 min. HRMS (ESI) calcd. For $C_{14}H_{19}NNaO_4$ $[M+Na]^+$: 288.1206, found: 288.1221.

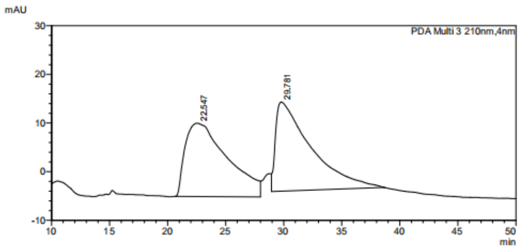

<Peak Table>

PDA Ch3 210nm

Peak#	Ret. Time	Area	Area%	Height	Height%
1	22.547	3809193	49.478	15069	45.149
2	29.781	3889502	50.522	18308	54.851
Total		7698694	100.000	33377	100.000

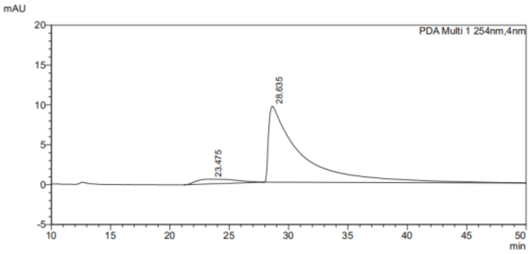

<Peak Table>

PDA Ch1 254nm

Peak#	Ret. Time	Area	Area%	Height	Height%
1	23.475	132844	6.859	594	5.879
2	28.635	1803896	93.141	9516	94.121
Total		1936740	100.000	10110	100.000

6. The X-ray structure of compound (*R*P, *S*, *S*, *R*S)-L2 borane complex, (*S*P, *R*, *S*, *R*S)-L2 borane complex , 6ba and 13:

7.1 The X-ray structure of compound (*R*P, *S*, *S*, *R*S)-L2 borane complex

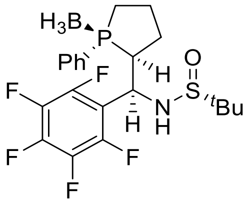

(R_P, *S*, *S*, R_S)-**L2** borane complex

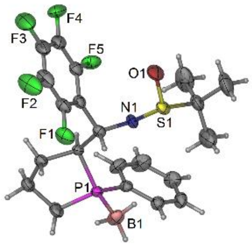

Bond precision:	C-C = 0.0045 A		Wavelength=0.71073
Cell:	a=6.5464(2)	b=17.3658(6)	c=21.0198(7)
	alpha=90	beta=96.370(1)	gamma=90
Temperature:	173 K		

	Calculated	Reported
Volume	2374.85(14)	2374.85(14)
Space group	P 21	P2(1)
Hall group	P 2yb	?
Moiety formula	C21 H25 B F5 N O P S	?
Sum formula	C21 H25 B F5 N O P S	C21 H25 B F5 N O P S
Mr	476.26	476.26
Dx,g cm-3	1.332	1.332
Z	4	4
Mu (mm-1)	0.255	0.255
F000	988.0	988.0
F000'	989.48	
h,k,lmax	7,20,25	7,20,25
Nref	8359[4330]	8322
Tmin,Tmax	0.912,0.931	0.914,0.932
Tmin'	0.912	

Correction method= # Reported T Limits: Tmin=0.914 Tmax=0.932
AbsCorr = MULTI-SCAN

Data completeness= 1.92/1.00 Theta(max)= 25.010

R(reflections)= 0.0373(7464) wR2(reflections)= 0.0912(8322)

S = 1.036 Npar= 583

7.2 The X-ray structure of compound (*S*P, *R*, *S*, *R*S)-L2 borane complex.

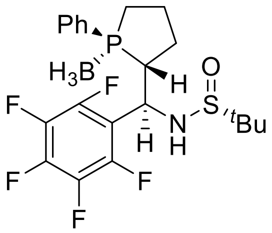

(S_P, *R*, *S*, R_S)-**L2** borane complex

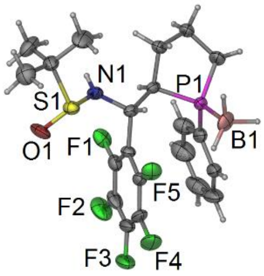

Bond precision: C-C = 0.0110 A Wavelength=0.71073

Cell: a=17.1138(14) b=6.6266(5) c=20.6037(16)
alpha=90 beta=93.452(3) gamma=90

Temperature: 173 K

	Calculated	Reported
Volume	2332.4(3)	2332.3(3)
Space group	P 21	P2(1)
Hall group	P 2yb	?
Moiety formula	C21 H26 B F5 N O P S	?
Sum formula	C21 H26 B F5 N O P S	C21 H26 B F5 N O P S
Mr	477.27	477.27
Dx,g cm-3	1.359	1.359
Z	4	4
Mu (mm-1)	0.260	0.260
F000	992.0	992.0
F000'	993.48	
h,k,lmax	20,7,24	20,7,24
Nref	8196[4489]	7869
Tmin,Tmax	0.942,0.964	0.928,0.965
Tmin'	0.927	

Correction method= # Reported T Limits: Tmin=0.928 Tmax=0.965
AbsCorr = MULTI-SCAN

Data completeness= 1.75/0.96 Theta(max)= 25.000

R(reflections)= 0.0714(6051) wR2(reflections)= 0.1800(7869)

S = 1.081 Npar= 583

7.3 The X-ray structure of compound 6ba.

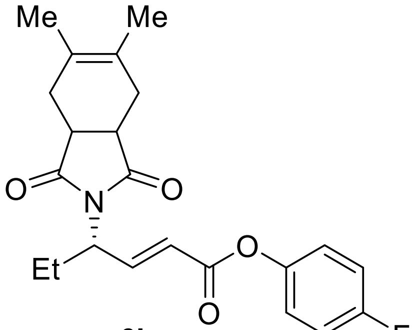

6ba

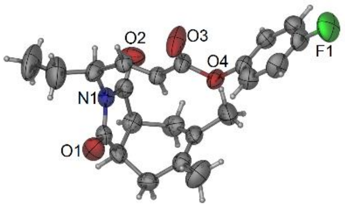

Bond precision: C-C = 0.0080 A Wavelength=1.54184

Cell:	a=11.4515(2)	b=13.8216(2)	c=13.0859(2)
	alpha=90	beta=95.239(2)	gamma=90

Temperature: 293 K

	Calculated	Reported
Volume	2062.56(6)	2062.56(6)
Space group	P 21/c	P 1 21/c 1
Hall group	-P 2ybc	-P 2ybc
Moiety formula	C22 H24 F N O4	C22 H24 F N O4
Sum formula	C22 H24 F N O4	C22 H24 F N O4
Mr	385.42	385.42
Dx, g cm-3	1.241	1.241
Z	4	4
Mu (mm-1)	0.753	0.753
F000	816.0	816.0
F000'	818.69	
h, k, lmax	13, 16, 15	13, 16, 15
Nref	3690	3604
Tmin, Tmax	0.756, 0.854	0.731, 1.000
Tmin'	0.713	

Correction method= # Reported T Limits: Tmin=0.731 Tmax=1.000 AbsCorr = MULTI-SCAN

Data completeness= 0.977 Theta(max)= 67.043

R(reflections)= 0.1146(3315) wR2(reflections)= 0.3195(3604)

S = 1.079 Npar= 257

7.4 The X-ray structure of compound 13.

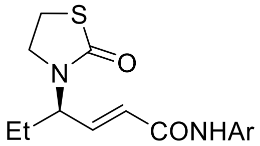

13

Ar = 1-naphthyl

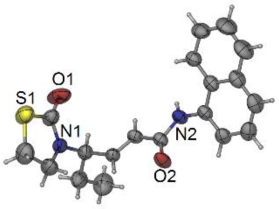

Bond precision:	C-C = 0.0097 A		Wavelength=1.54184
Cell:	a=7.2656(3)	b=9.5413(3)	c=13.0878(4)
	alpha=90	beta=100.119(3)	gamma=90
Temperature: 293 K			

	Calculated	Reported
Volume	893.18(5)	893.18(5)
Space group	P 21	P 1 21 1
Hall group	P 2yb	P 2yb
Moiety formula	C19 H20 N2 O2 S	C19 H20 N2 O2 S
Sum formula	C19 H20 N2 O2 S	C19 H20 N2 O2 S
Mr	340.43	340.43
Dx, g cm-3	1.266	1.266
Z	2	2
Mu (mm-1)	1.712	1.712
F000	360.0	360.0
F000'	361.62	
h,k,lmax	8,11,15	8,11,15
Nref	3193[1701]	3162
Tmin,Tmax	0.544,0.641	0.553,1.000
Tmin'	0.464	

Correction method= # Reported T Limits: Tmin=0.553 Tmax=1.000 AbsCorr = MULTI-SCAN

Data completeness= 1.86/0.99 Theta(max)= 67.074

R(reflections)= 0.0766(2544) wR2(reflections)= 0.2144(3162)

S = 1.040 Npar= 218

7. References:

[1]. a) W. Tang, X. Zhang, *Angew. Chem. Int. Ed.* **2002**, *41*, 1612; b) X.-M. Sun, K. Manabe, W. W.-L. Lam, N. Shiraishi, J. Kobayashi, S. Kobayashi, *Chem. Eur. J.* **2005**, *11*, 361; c) A. Haynes, G. L. Williams, *Organometallics* **2007**, *26*, 713.

[2] I. Franzoni, L. Guénée, C. Mazet, *Tetrahedron* **2014**, *70*, 4181.

[3] Y. He, R. J. Hinklin, J. Chang, L. L. Kiessling, *Org. Lett.* **2004**, *6*, 4479.

[4] T. Wang, Z. Yu, D. L. Hoon, C. Phee, Y. Lan, Y. Lu, *J. Am. Chem. Soc.* **2016**, *138*, 265.

[5] a) S. W. Smith, G. C. Fu, *J. Am. Chem. Soc.* **2009**, *131*, 14231; b) R. Sinisi, J. Sun, G. C. Fu, *Proc. Natl. Acad. Sci. U. S. A.* **2010**, *107*, 20652; c) R. J. Lundgren, A. Wilsily, N. Marion, C. Ma, Y. K. Chung, G. C. Fu, *Angew. Chem. Int. Ed.* **2013**, *52*, 2525; d) M. Kalek, G. C. Fu, *J. Am. Chem. Soc*. **2015**, *137*, 9438.

[6] S. L. Wiskur, G. C. Fu, *J. Am. Chem. Soc*. **2005**, *127*, 6176.

[7] Z. Liu, H.-Q. Ni, T. Zeng, K. M. Engle, *J. Am. Chem. Soc*. **2018**, *140*, 3223.

[8] K. N. Houk, Y. Lin, F. K. Brown, *J. Am. Chem. Soc*. **1986**, *108*, 554.

[9] J. Li, W. Chang, W. Ren, J. Dai, Y. Shi, *Org. Lett.* **2016**, *18*, 5456.

[10] M. Nasopoulou, D. Georgiadis, M. Matziari, V. Dive, A. Yiotakis, *J. Org. Chem*. **2007**, *72*, 7222.

[11] B. M. Trost, C. Lee, *J. Am. Chem. Soc.* **2001**, *123*, 12191.

8. NMR spectra:

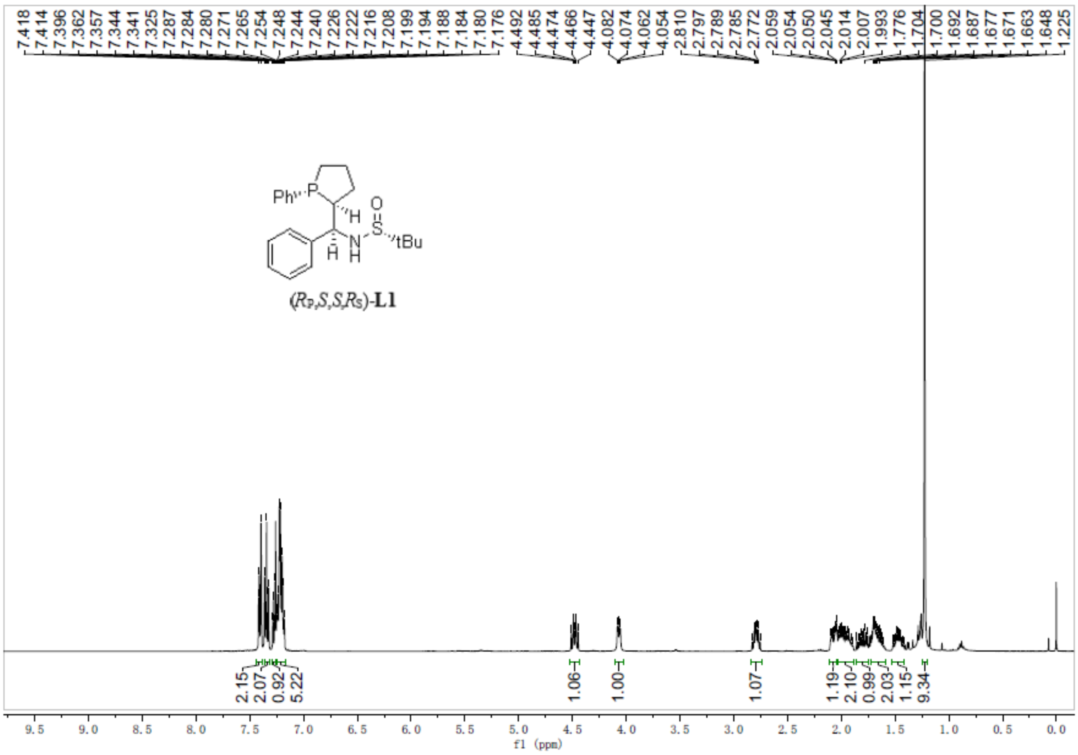

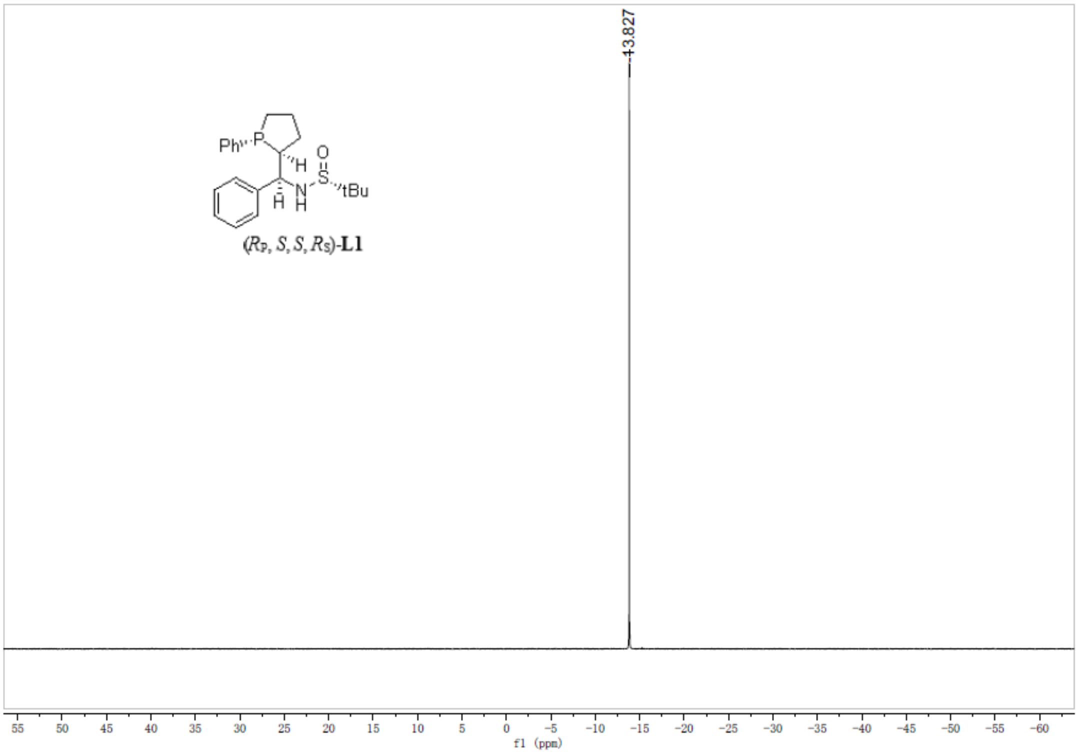

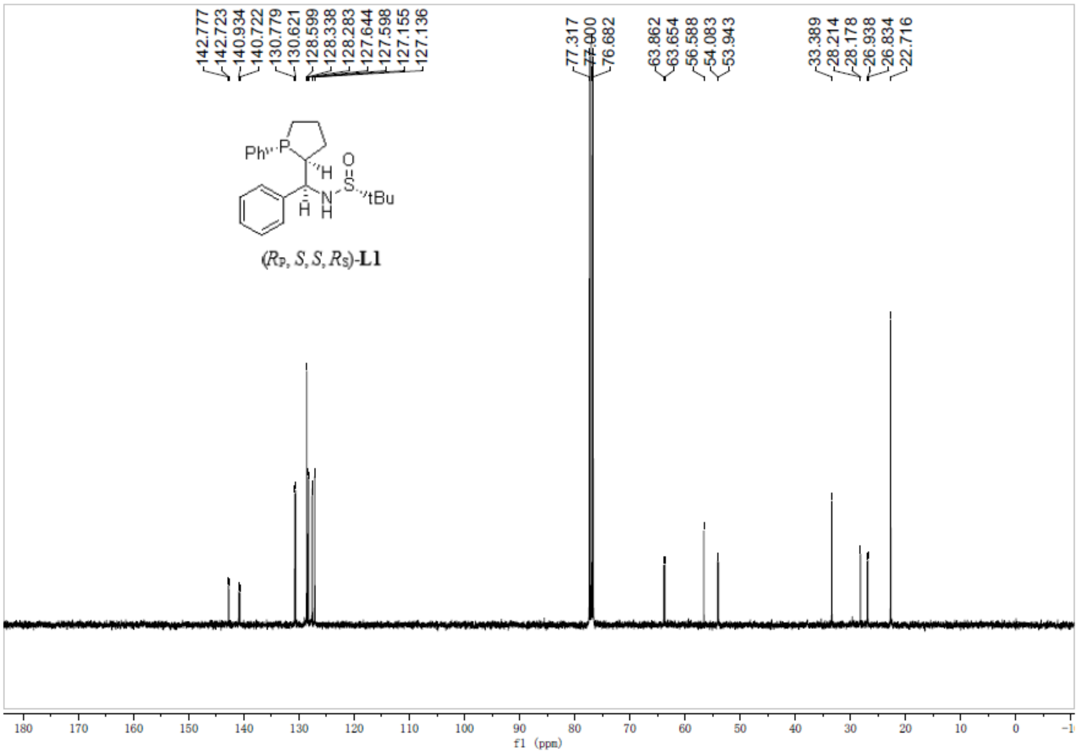
142.777
142.723
140.934
140.722
130.779
130.621
128.599
128.338
128.283
127.644
127.598
127.155
127.136
77.317
77.000
76.682
63.862
63.654
56.588
54.083
53.943
33.389
28.214
28.178
26.938
26.834
22.716
Ph P H O S N tBu H H
(RP, S, S, RS)-L1
f1 (ppm)

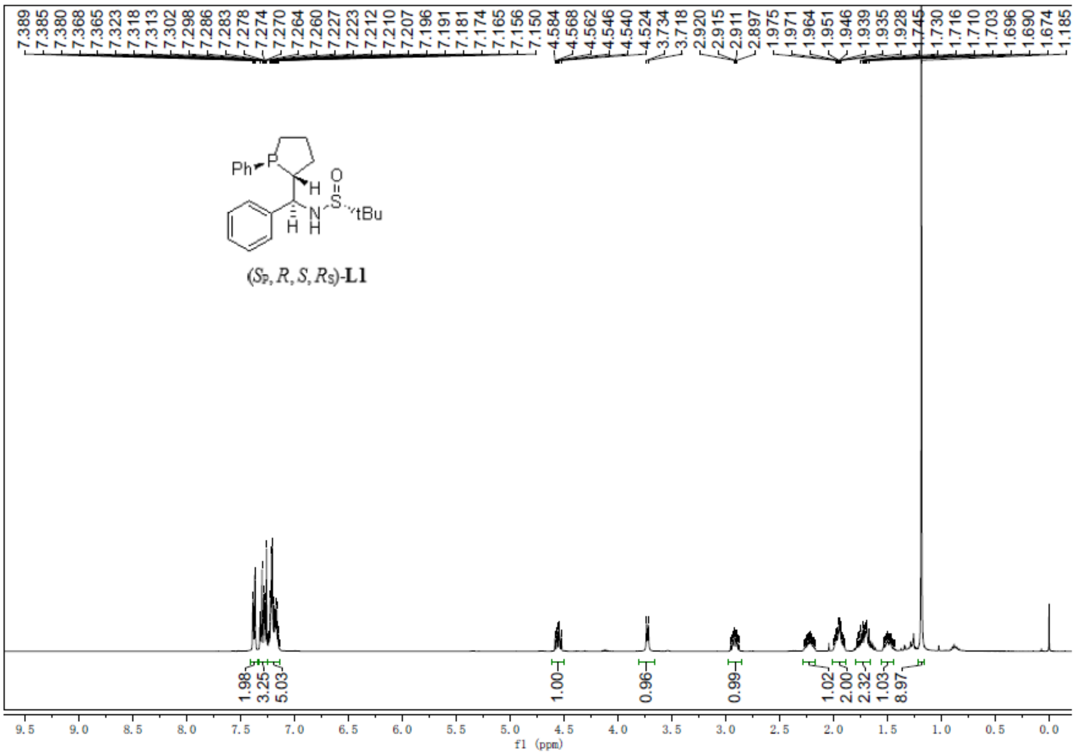
7.389
7.385
7.380
7.368
7.365
7.323
7.318
7.313
7.302
7.298
7.286
7.283
7.278
7.274
7.270
7.264
7.260
7.227
7.223
7.212
7.210
7.207
7.196
7.191
7.181
7.174
7.165
7.156
7.150
4.584
4.568
4.562
4.546
4.540
4.524
3.734
3.718
2.920
2.915
2.911
2.897
1.975
1.971
1.964
1.951
1.946
1.939
1.935
1.928
1.745
1.730
1.716
1.710
1.703
1.696
1.690
1.674
1.185
Ph P H O S N tBu H H
(SP, R, S, RS)-L1
1.98
3.25
5.03
1.00
0.96
0.99
1.02
2.00
2.32
1.03
8.97
f1 (ppm)

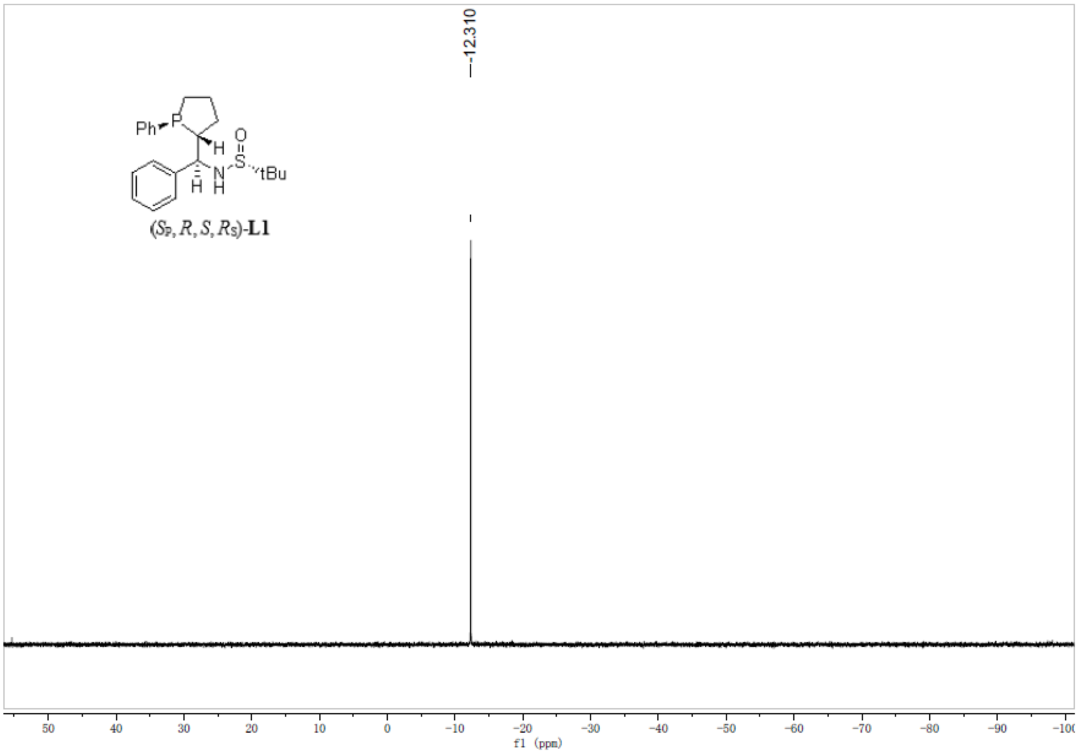
-12.310
Ph
P
H
O
N
S
tBu
H
H
(SP, R, S, RS)-L1
f1 (ppm)

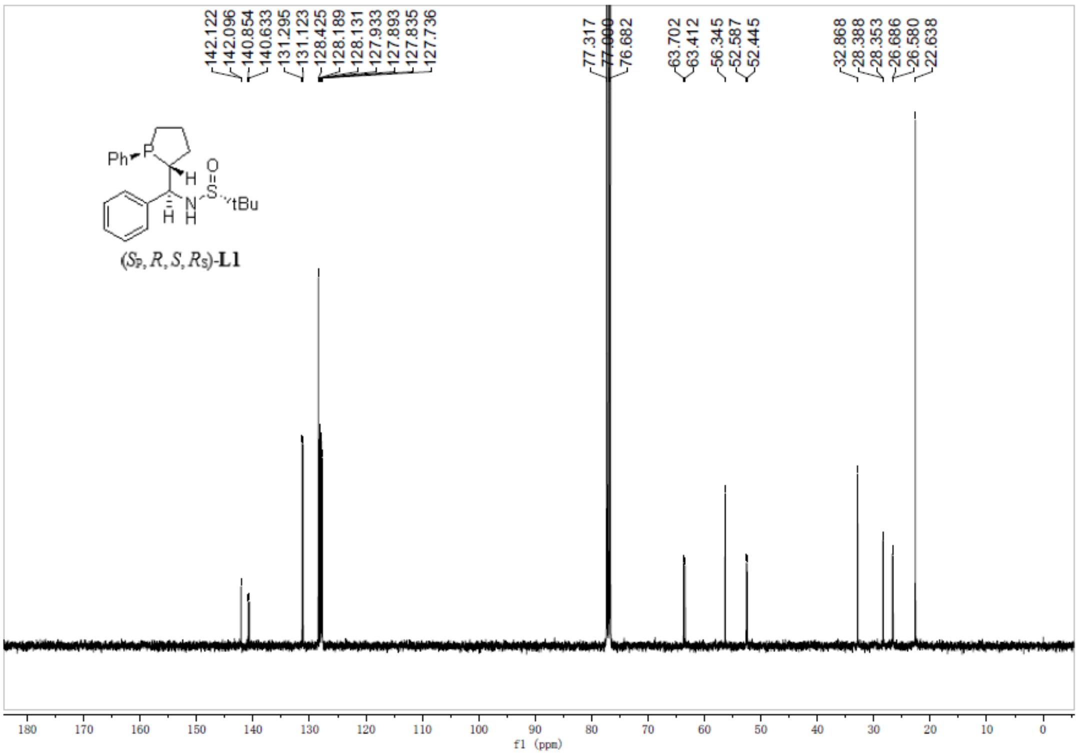
142.122
142.096
140.854
140.633
131.295
131.123
128.425
128.189
128.131
127.933
127.893
127.835
127.736
77.317
76.682
63.702
63.412
56.345
52.587
52.445
32.868
28.388
28.353
26.686
26.580
22.638
Ph
P
H
O
N
S
tBu
H
H
(SP, R, S, RS)-L1
f1 (ppm)

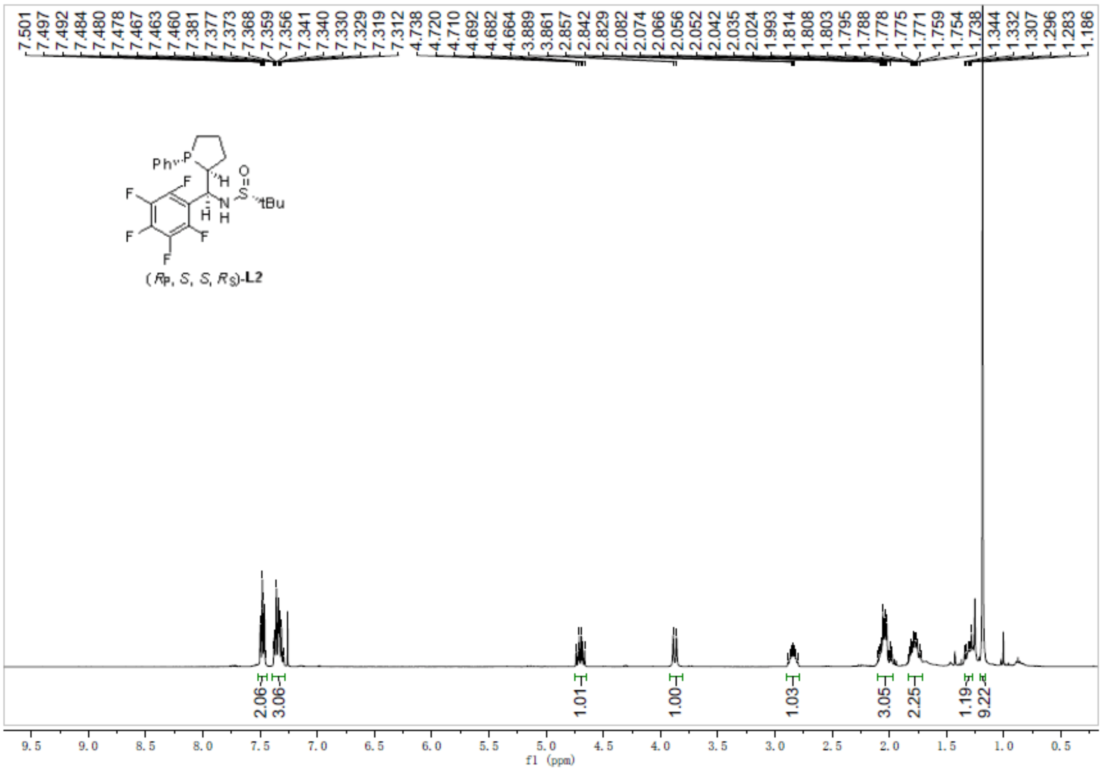
7.501
7.497
7.492
7.484
7.480
7.478
7.467
7.463
7.460
7.381
7.377
7.373
7.368
7.359
7.356
7.341
7.340
7.330
7.329
7.319
7.312
4.738
4.720
4.710
4.692
4.682
4.664
3.889
3.861
2.857
2.842
2.829
2.082
2.074
2.066
2.056
2.052
2.042
2.035
2.024
1.993
1.814
1.808
1.803
1.795
1.788
1.778
1.775
1.771
1.759
1.754
1.738
1.344
1.332
1.307
1.296
1.283
1.186
Ph
P
H
O
S
F
N
tBu
F
H
H
F
F
F
(*R*P, *S*, *S*, *R*S)-**L2**
2.06
3.06
1.01
1.00
1.03
3.05
2.25
1.19
9.22
f1 (ppm)

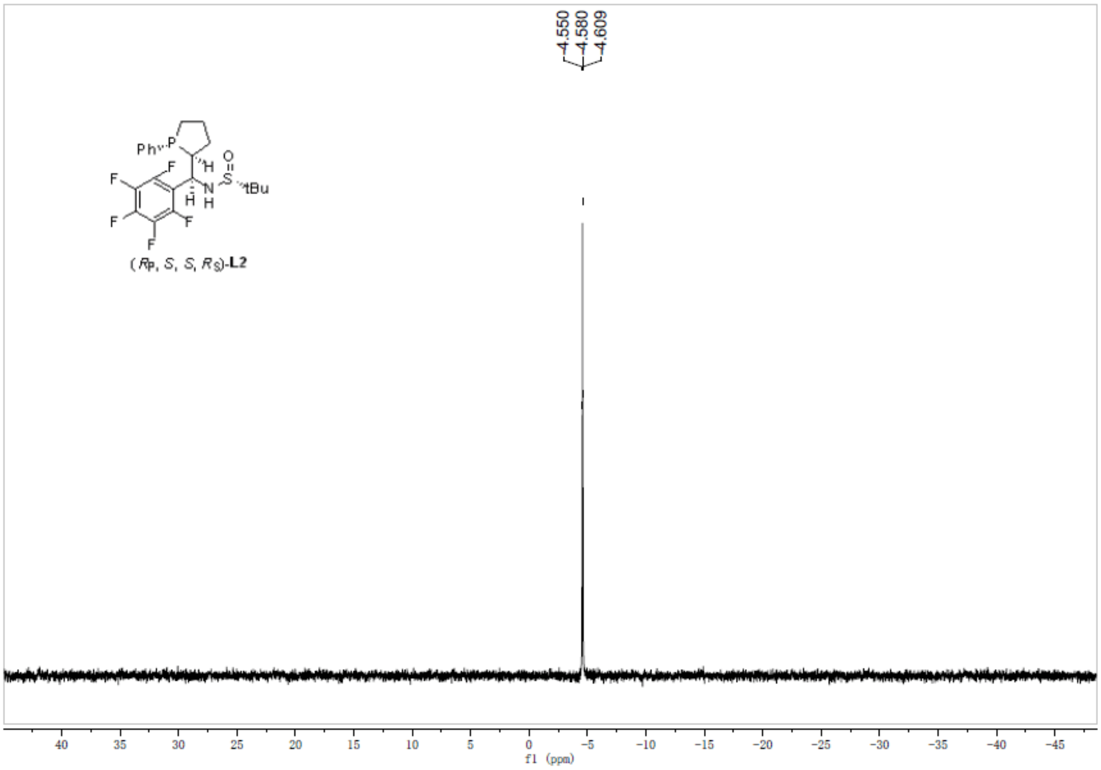
-4.550
-4.580
-4.609
Ph
P
H
O
S
F
N
tBu
F
H
H
F
F
F
(*R*P, *S*, *S*, *R*S)-**L2**
f1 (ppm)

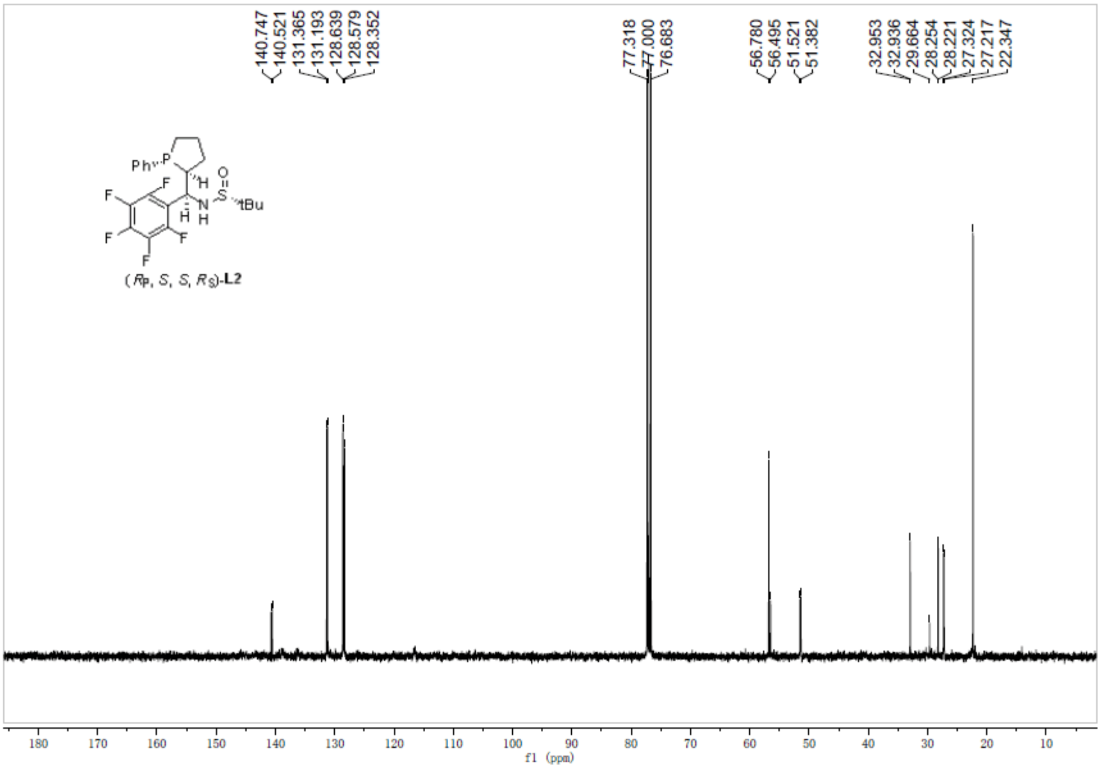
140.747
140.521
131.365
131.193
128.639
128.579
128.352
77.318
77.000
76.683
56.780
56.495
51.521
51.382
32.953
32.936
29.664
28.254
28.221
27.324
27.217
22.347
(R_P, S, S, R_S)-L2
f1 (ppm)

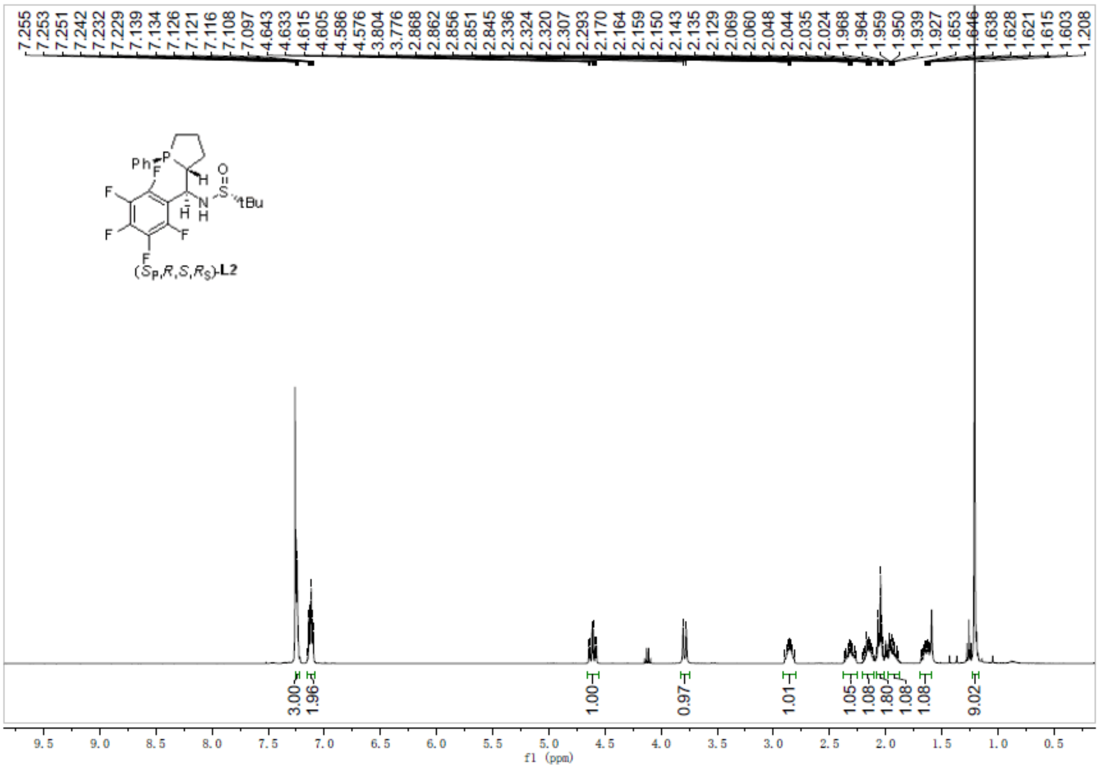
(S_P,R,S,R_S)-L2
3.00
1.96
1.00
0.97
1.01
1.05
1.08
1.80
1.08
1.08
9.02
f1 (ppm)

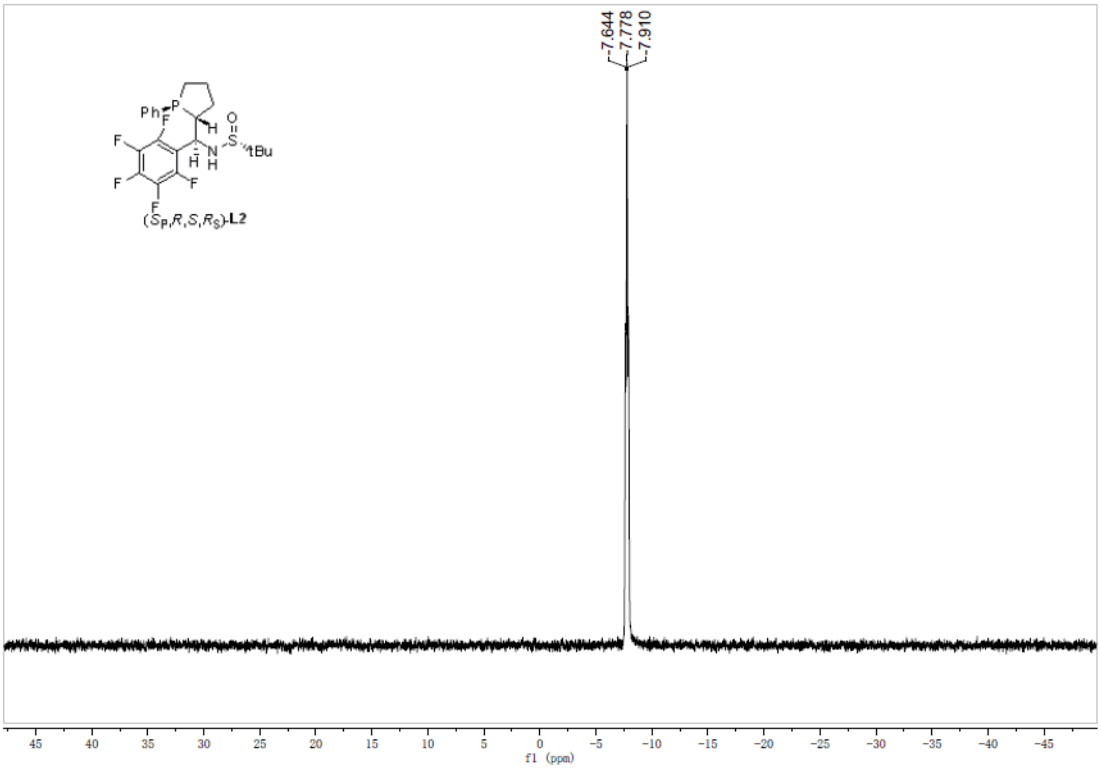

-7.644
-7.778
-7.910
Ph
P
F
H
O
S
N
tBu
F
H
F
F
F
F
(S_P,R,S,R_S)-L2
f1 (ppm)

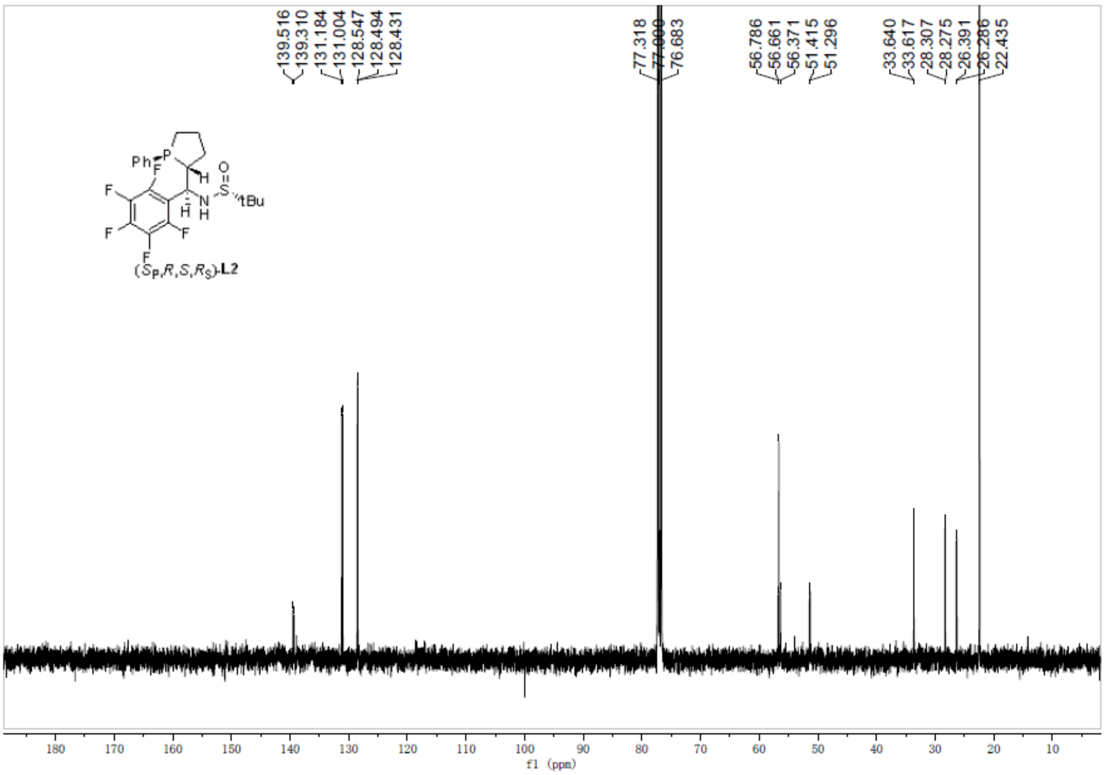

139.516
139.310
131.184
131.004
128.547
128.494
128.431
77.318
76.683
56.786
56.661
56.371
51.415
51.296
33.640
33.617
28.307
28.275
26.391
22.435
(S_P,R,S,R_S)-L2
f1 (ppm)

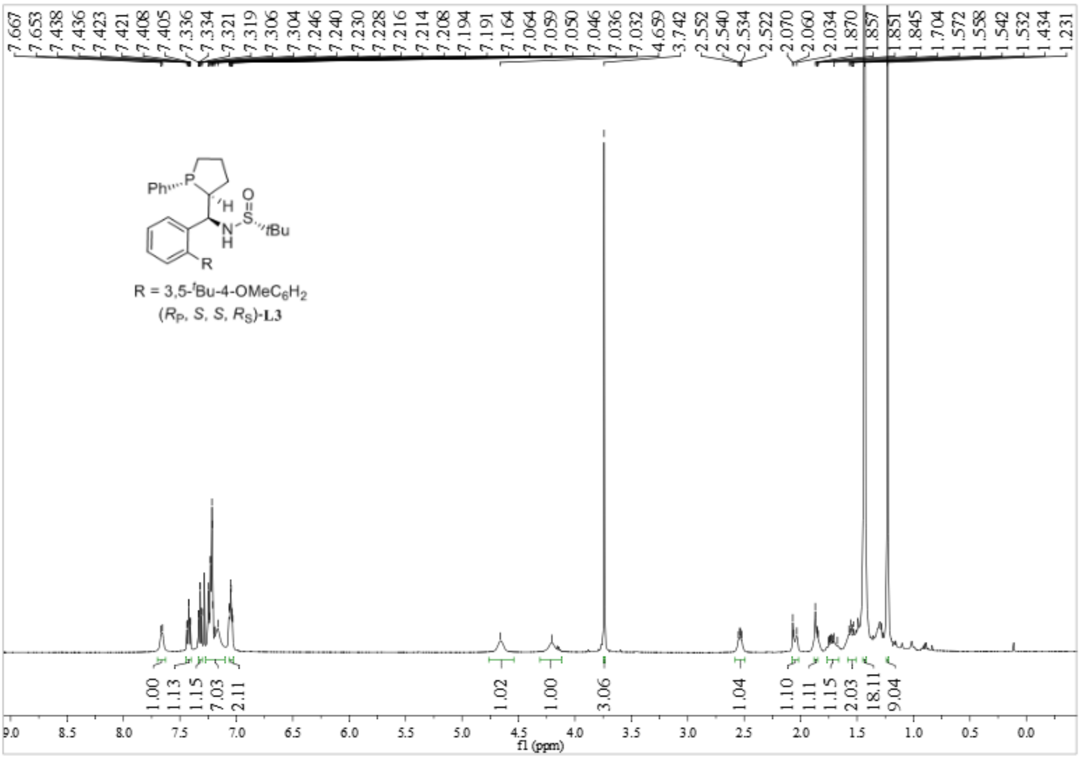
7.667
7.653
7.438
7.436
7.423
7.421
7.408
7.405
7.336
7.334
7.321
7.319
7.306
7.304
7.246
7.240
7.230
7.228
7.216
7.214
7.208
7.194
7.191
7.164
7.064
7.059
7.050
7.046
7.036
7.032
4.659
3.742
2.552
2.540
2.534
2.522
2.070
2.060
2.034
1.870
1.857
1.851
1.845
1.704
1.572
1.558
1.542
1.532
1.434
1.231
Ph
P
H
O
S
N
H
tBu
R
R = 3,5-^{t}Bu-4-OMeC$_6$H$_2$
(R_P, S, S, R_S)-**L3**
1.00
1.13
1.15
7.03
2.11
1.02
1.00
3.06
1.04
1.10
1.11
1.15
2.03
18.11
9.04
f1 (ppm)

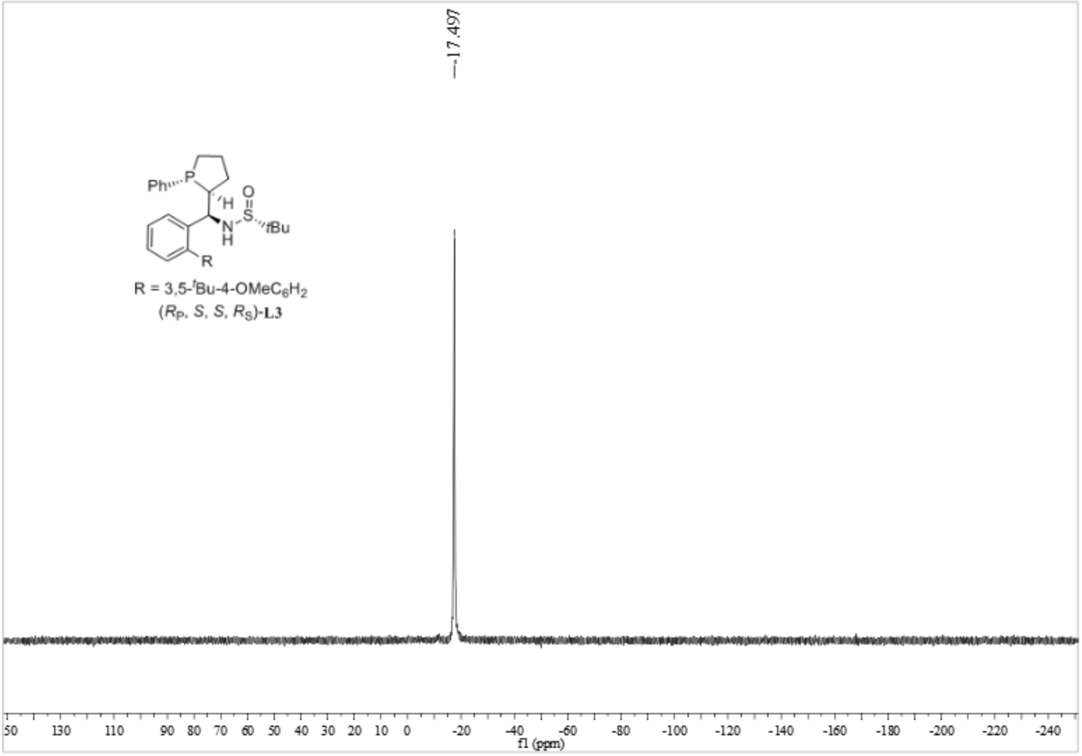
-17.497
Ph
P
H
O
S
N
H
tBu
R
R = 3,5-^{t}Bu-4-OMeC$_6$H$_2$
(R_P, S, S, R_S)-**L3**
f1 (ppm)

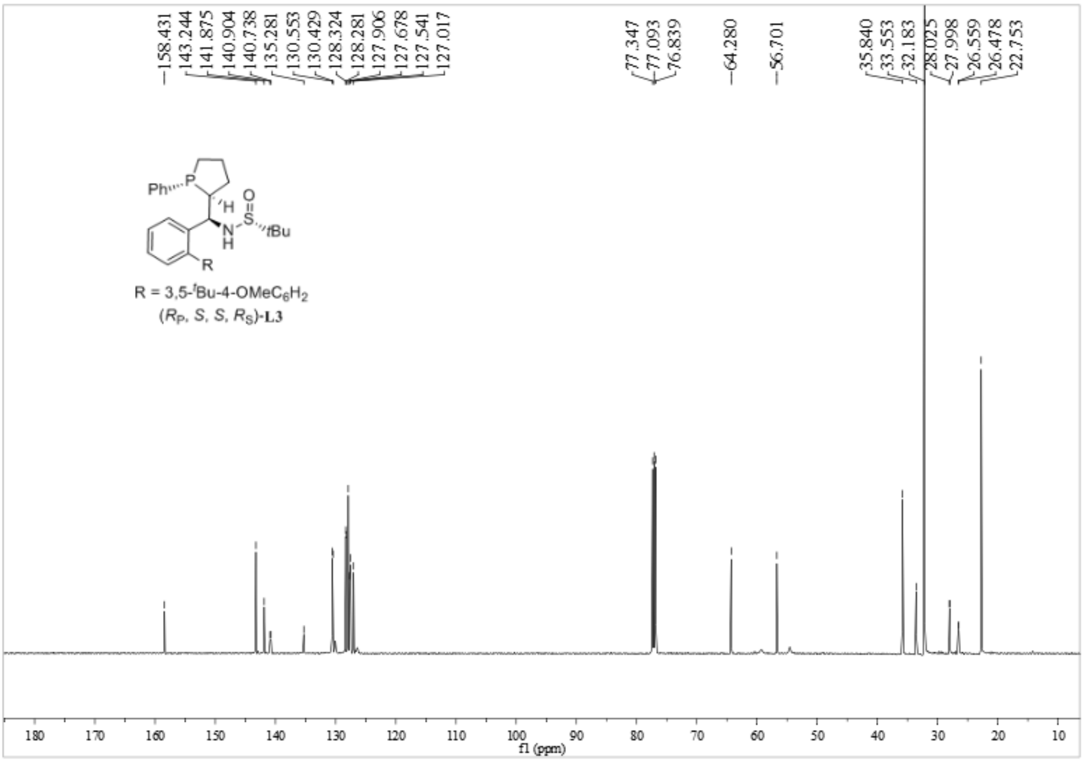
158.431 143.244 141.875 140.904 140.738 135.281 130.553 130.429 128.324 128.281 127.906 127.678 127.541 127.017
77.347 77.093 76.839
64.280
56.701
35.840 33.553 32.183 28.025 27.998 26.559 26.478 22.753
Ph
P
H
O
S
N
H
tBu
R
R = 3,5-^{t}Bu-4-OMeC$_6$H$_2$
(R_P, S, S, R_S)-L3
f1 (ppm)

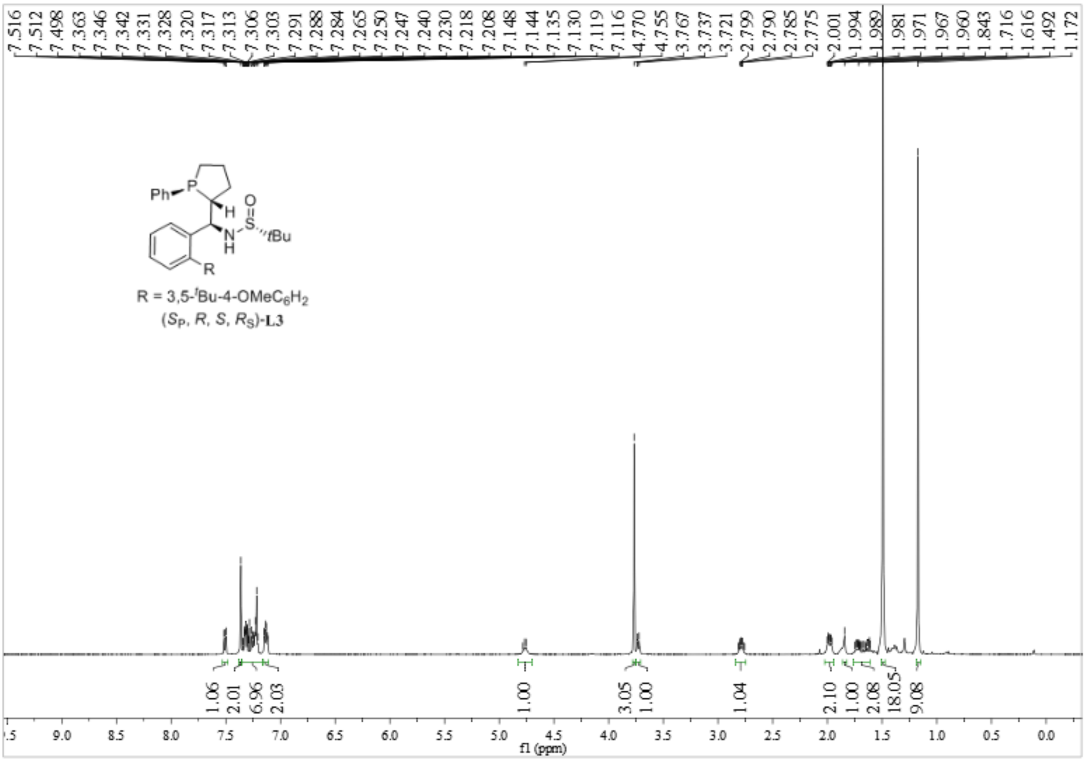
7.516 7.512 7.498 7.363 7.346 7.342 7.331 7.328 7.320 7.317 7.313 7.306 7.303 7.291 7.288 7.284 7.265 7.250 7.247 7.240 7.230 7.218 7.208 7.148 7.144 7.135 7.130 7.119 7.116 4.770 4.755 3.767 3.737 3.721 2.799 2.790 2.785 2.775 2.001 1.994 1.989 1.981 1.971 1.967 1.960 1.843 1.716 1.616 1.492 1.172
Ph
P
H
O
S
N
H
tBu
R
R = 3,5-^{t}Bu-4-OMeC$_6$H$_2$
(S_P, R, S, R_S)-L3
1.06 2.01 6.96 2.03
1.00
3.05 1.00
1.04
2.10 1.00 2.08 18.05 9.08
f1 (ppm)

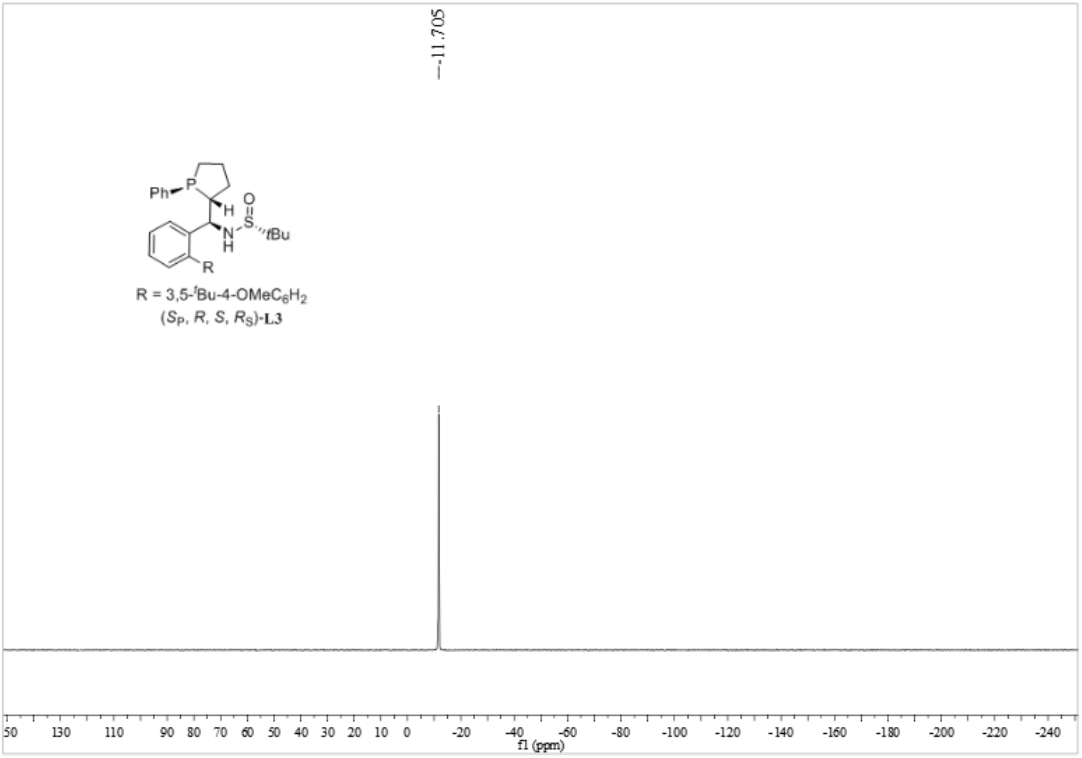

-11.705
Ph
P
H
O
S
N
H
tBu
R
R = 3,5-tBu-4-OMeC6H2
(SP, R, S, RS)-L3
f1 (ppm)

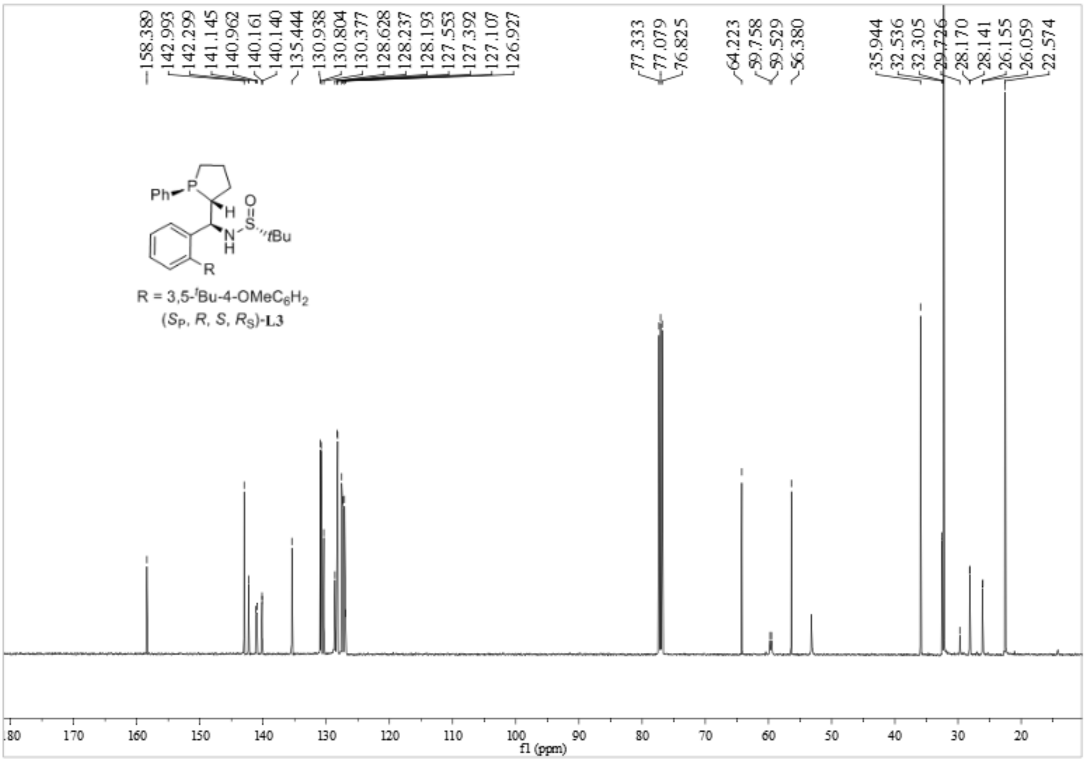

158.389
142.993
142.299
141.145
140.962
140.161
140.140
135.444
130.938
130.804
130.377
128.628
128.237
128.193
127.553
127.392
127.107
126.927
77.333
77.079
76.825
64.223
59.758
59.529
56.380
35.944
32.536
32.305
29.726
28.170
28.141
26.155
26.059
22.574
Ph
P
H
O
S
N
H
tBu
R
R = 3,5-tBu-4-OMeC6H2
(SP, R, S, RS)-L3
f1 (ppm)

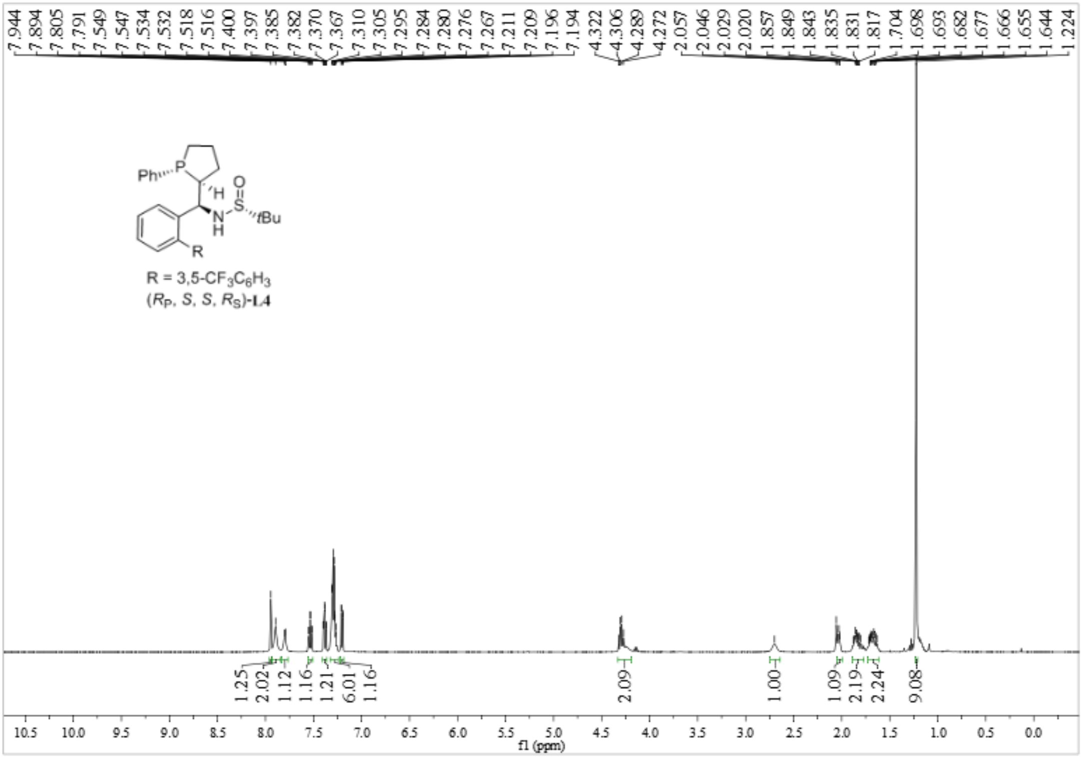
R = 3,5-CF3C6H3
(RP, S, S, RS)-L4
f1 (ppm)

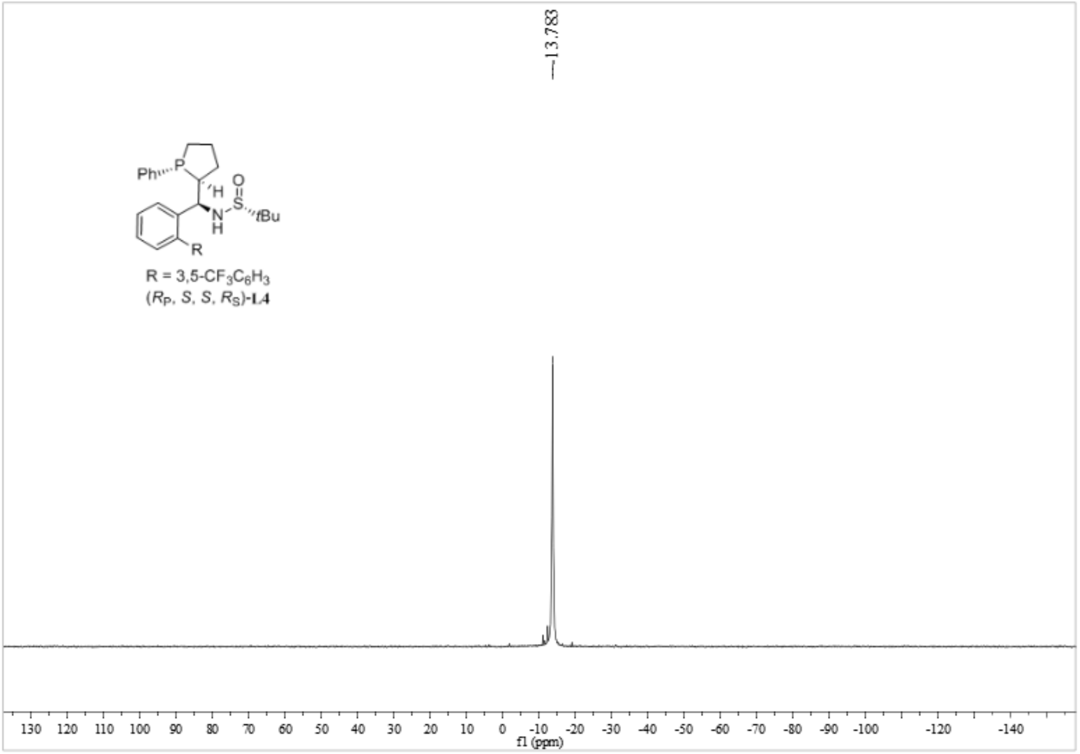
-13.783
R = 3,5-CF3C6H3
(RP, S, S, RS)-L4
f1 (ppm)

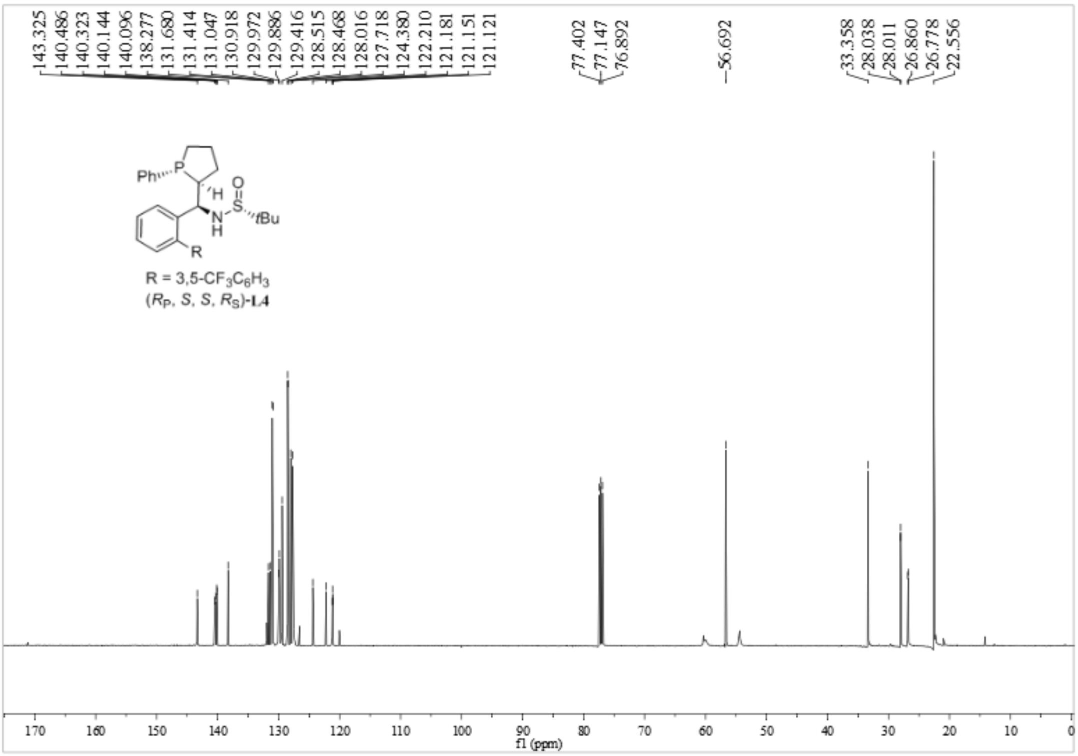
143.325
140.486
140.323
140.144
140.096
138.277
131.680
131.414
131.047
130.918
129.972
129.886
129.416
128.515
128.468
128.016
127.718
124.380
122.210
121.181
121.151
121.121
77.402
77.147
76.892
56.692
33.358
28.038
28.011
26.860
26.778
22.556
Ph
P
H
O
S
N
H
tBu
R
R = 3,5-CF3C6H3
(RP, S, S, RS)-L4
f1 (ppm)

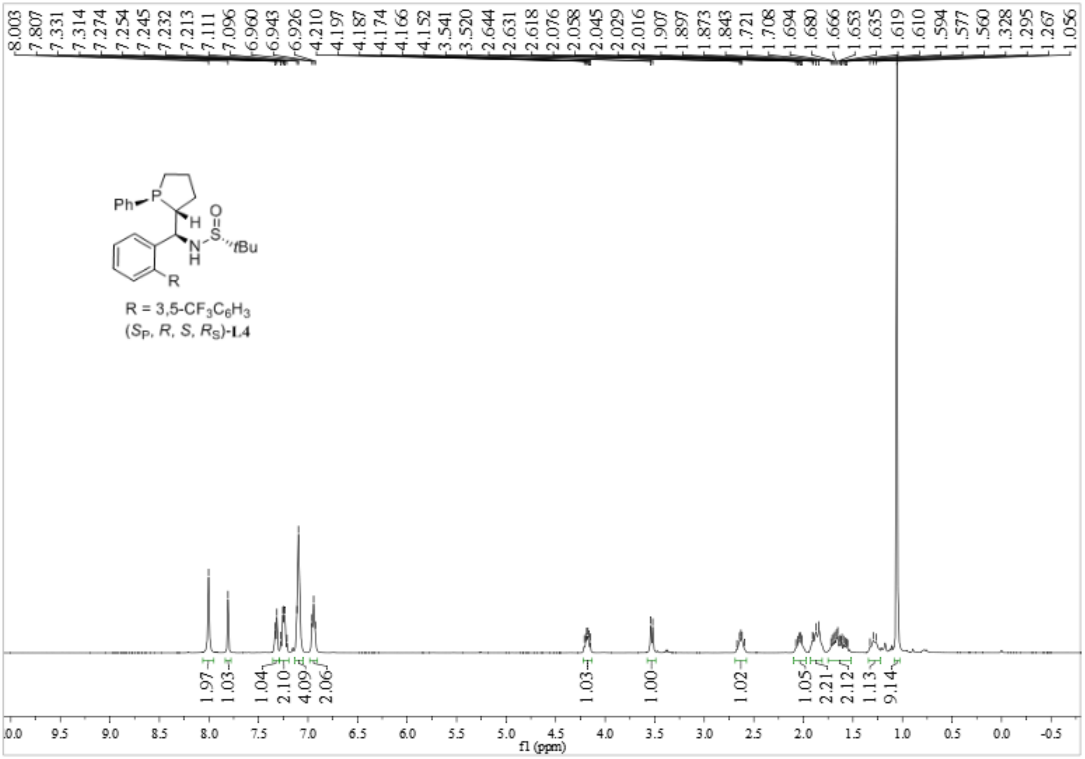
8.003
7.807
7.331
7.314
7.274
7.254
7.245
7.232
7.213
7.111
7.096
6.960
6.943
6.926
4.210
4.197
4.187
4.174
4.166
4.152
3.541
3.520
2.644
2.631
2.618
2.076
2.058
2.045
2.029
2.016
1.907
1.897
1.873
1.843
1.721
1.708
1.694
1.680
1.666
1.653
1.635
1.619
1.610
1.594
1.577
1.560
1.328
1.295
1.267
1.056
Ph
P
H
O
S
N
H
tBu
R
R = 3,5-CF3C6H3
(SP, R, S, RS)-L4
1.97
1.03
1.04
2.10
4.09
2.06
1.03
1.00
1.02
1.05
2.21
2.12
1.13
9.14
f1 (ppm)

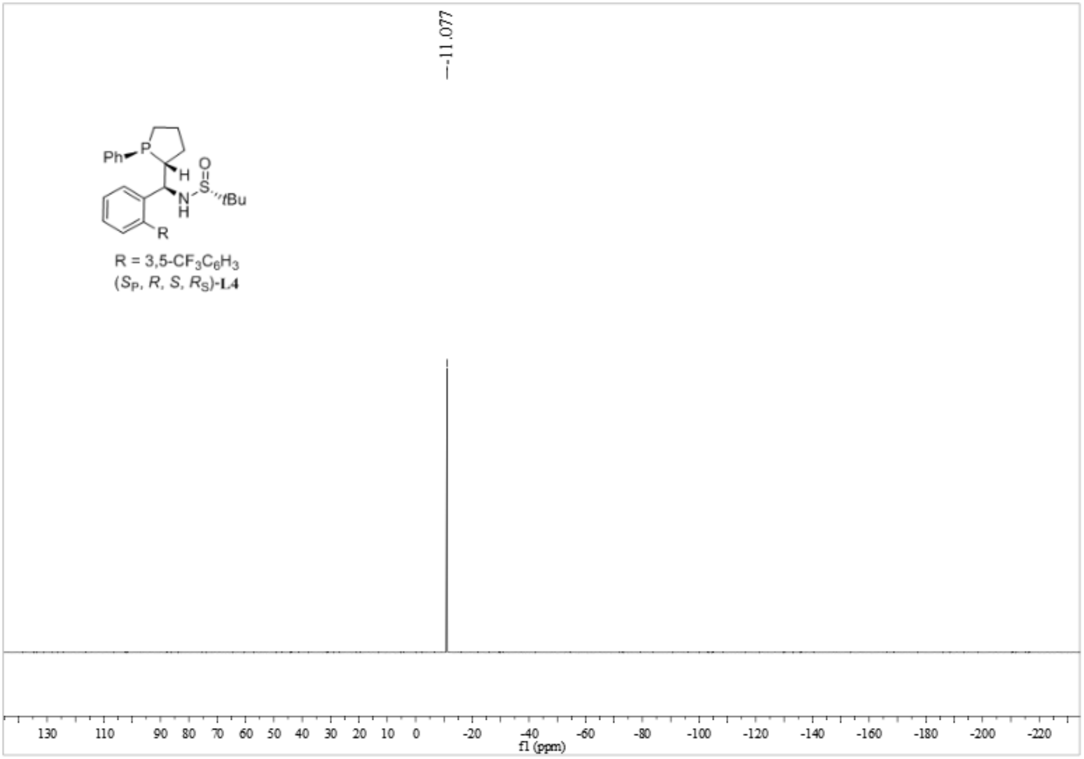
--11.077
Ph
P
H
O
S
N
H
tBu
R
R = 3,5-CF3C6H3
(SP, R, S, RS)-L4
fl (ppm)

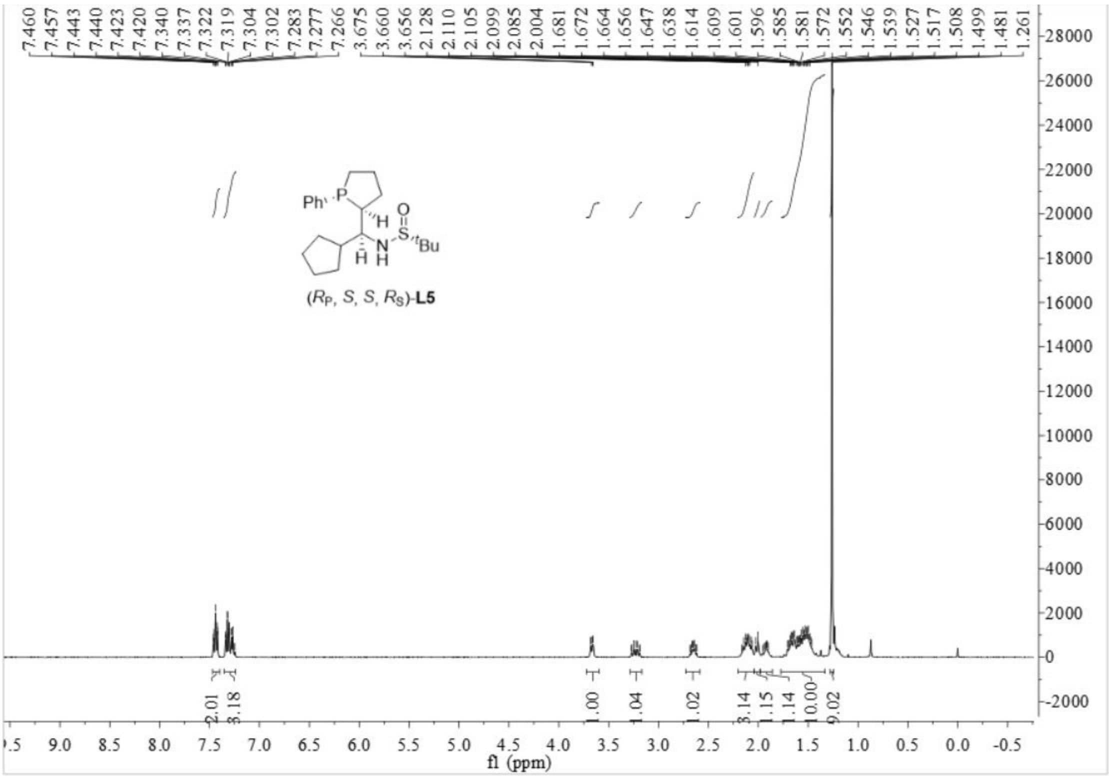
Ph
P
H
O
S
N
H
H
tBu
(RP, S, S, RS)-L5
fl (ppm)

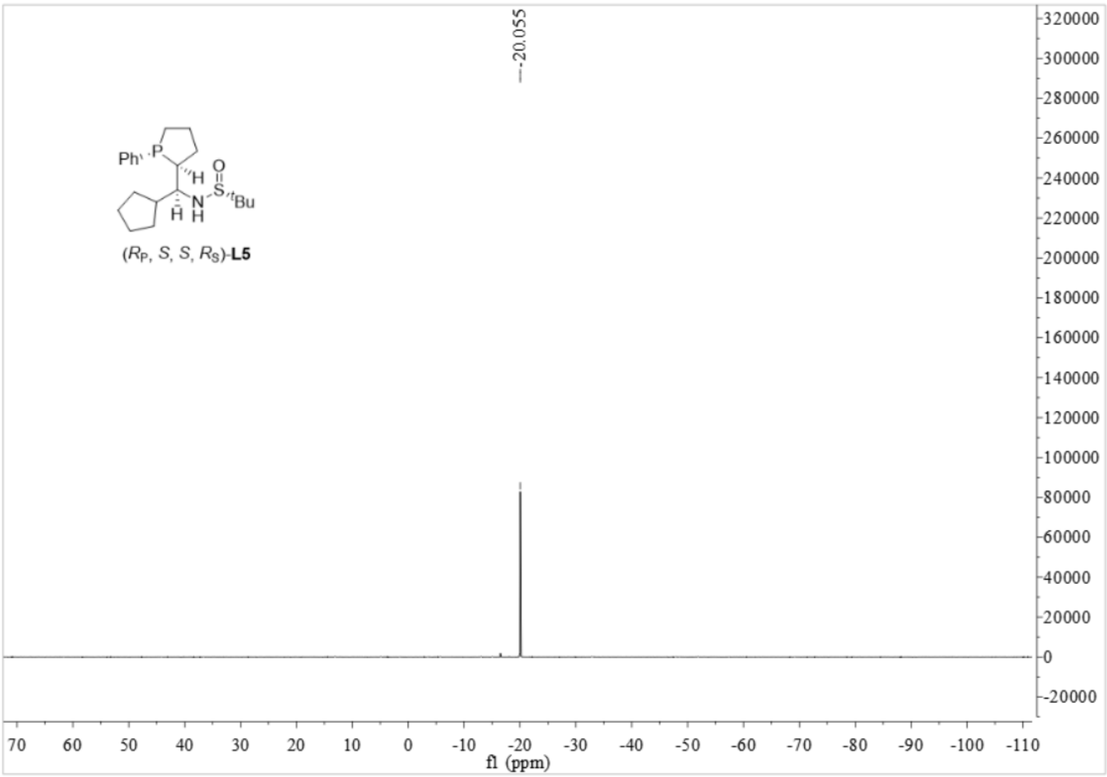
-20.055
Ph
P
H
O
N
S
tBu
H
H
(RP, S, S, RS)-L5
f1 (ppm)

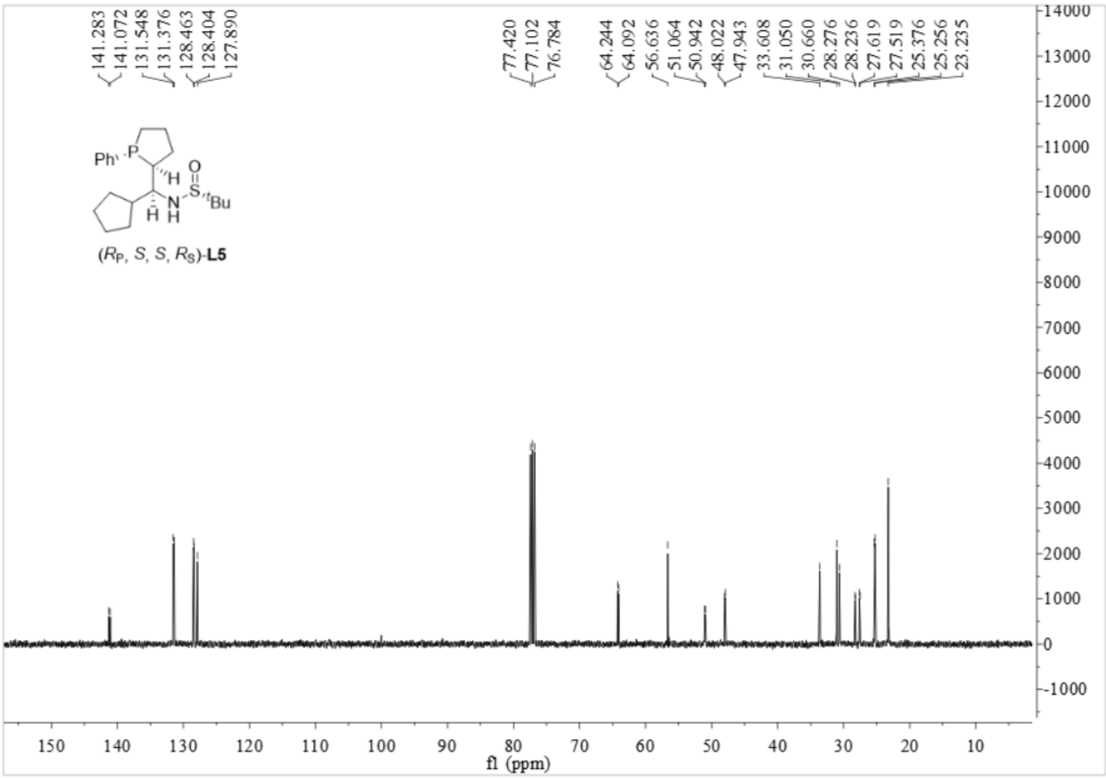
141.283
141.072
131.548
131.376
128.463
128.404
127.890
77.420
77.102
76.784
64.244
64.092
56.636
51.064
50.942
48.022
47.943
33.608
31.050
30.660
28.276
28.236
27.619
27.519
25.376
25.256
23.235
Ph
P
H
O
N
S
tBu
H
H
(RP, S, S, RS)-L5
f1 (ppm)

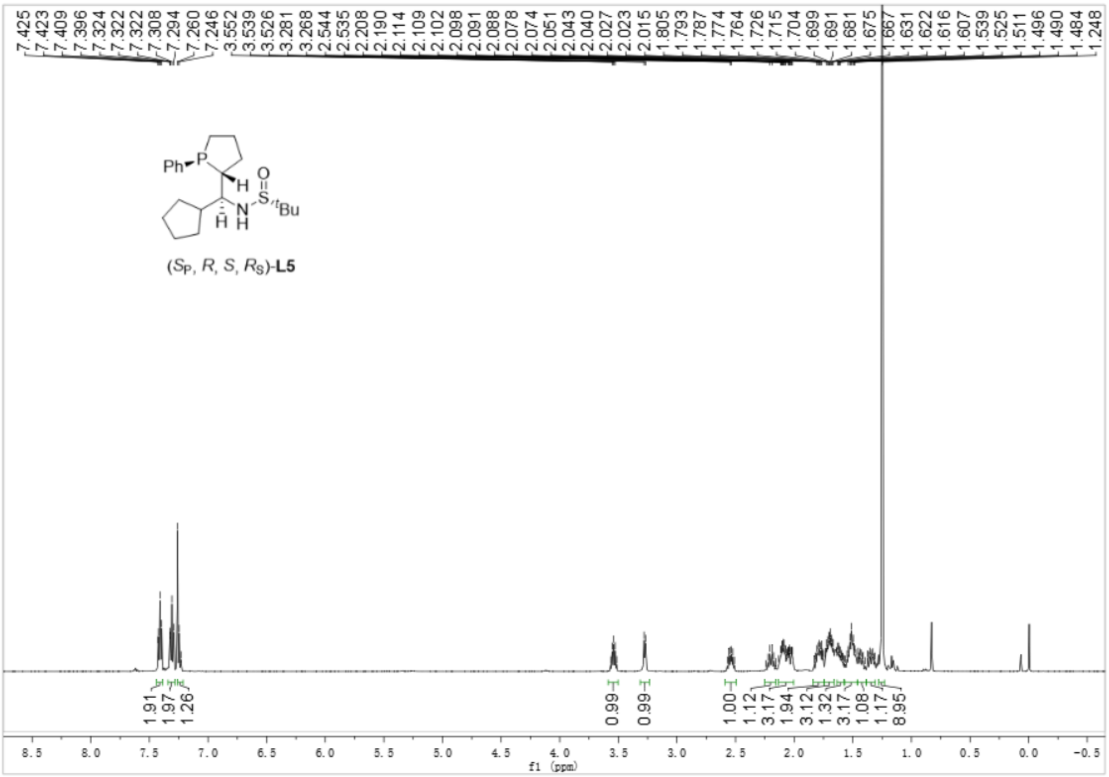
7.425
7.423
7.409
7.396
7.324
7.322
7.322
7.308
7.294
7.260
7.246
3.552
3.539
3.526
3.281
3.268
2.544
2.535
2.208
2.190
2.114
2.109
2.102
2.098
2.091
2.088
2.078
2.074
2.051
2.043
2.040
2.027
2.023
2.015
1.805
1.793
1.787
1.774
1.764
1.726
1.715
1.704
1.699
1.691
1.681
1.675
1.667
1.631
1.622
1.616
1.607
1.539
1.525
1.511
1.496
1.490
1.484
1.248
Ph
P
H
O
S
N
tBu
H
H
(SP, R, S, RS)-L5
1.91
1.97
1.26
0.99
0.99
1.00
1.12
3.17
1.94
3.12
1.32
3.17
1.08
1.17
8.95
f1 (ppm)

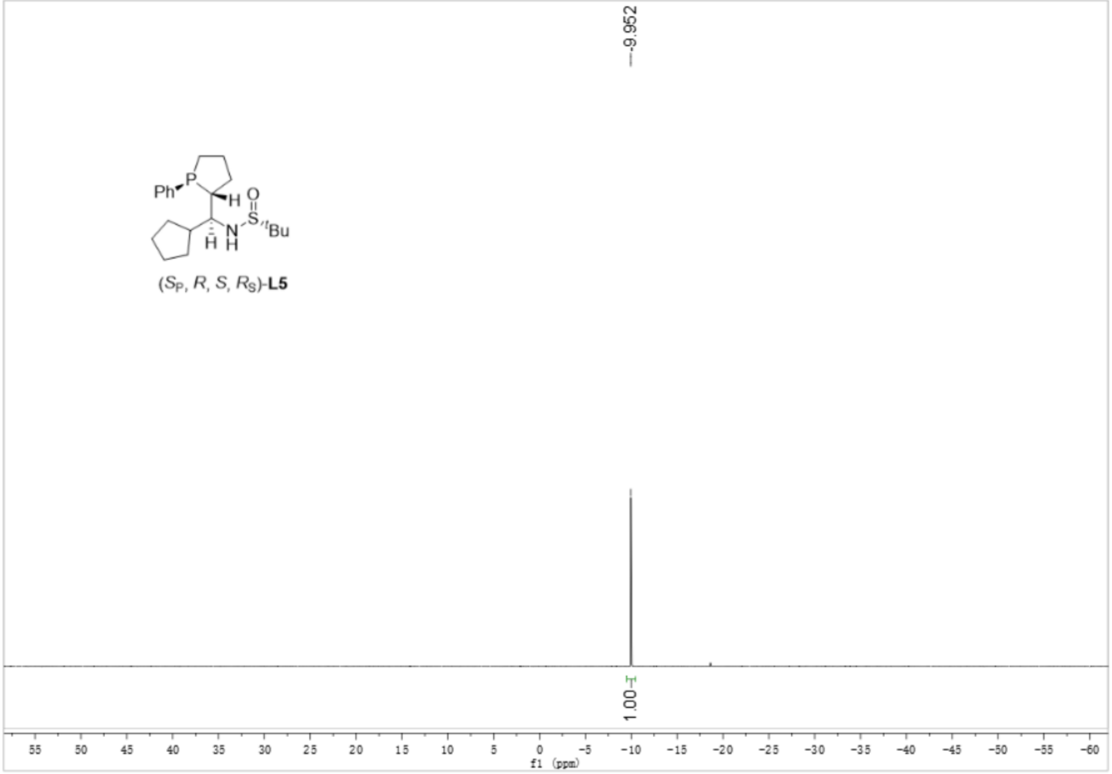
-9.952
Ph
P
H
O
S
N
tBu
H
H
(SP, R, S, RS)-L5
1.00
f1 (ppm)

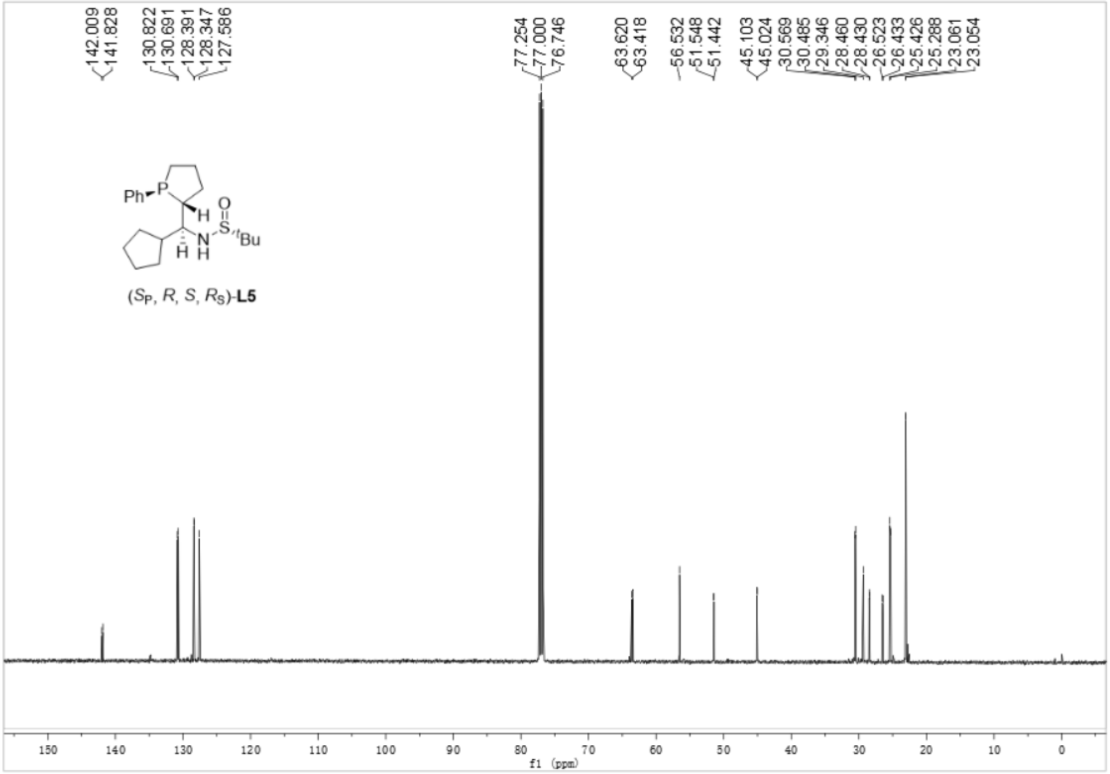
(SP, R, S, RS)-L5
f1 (ppm)

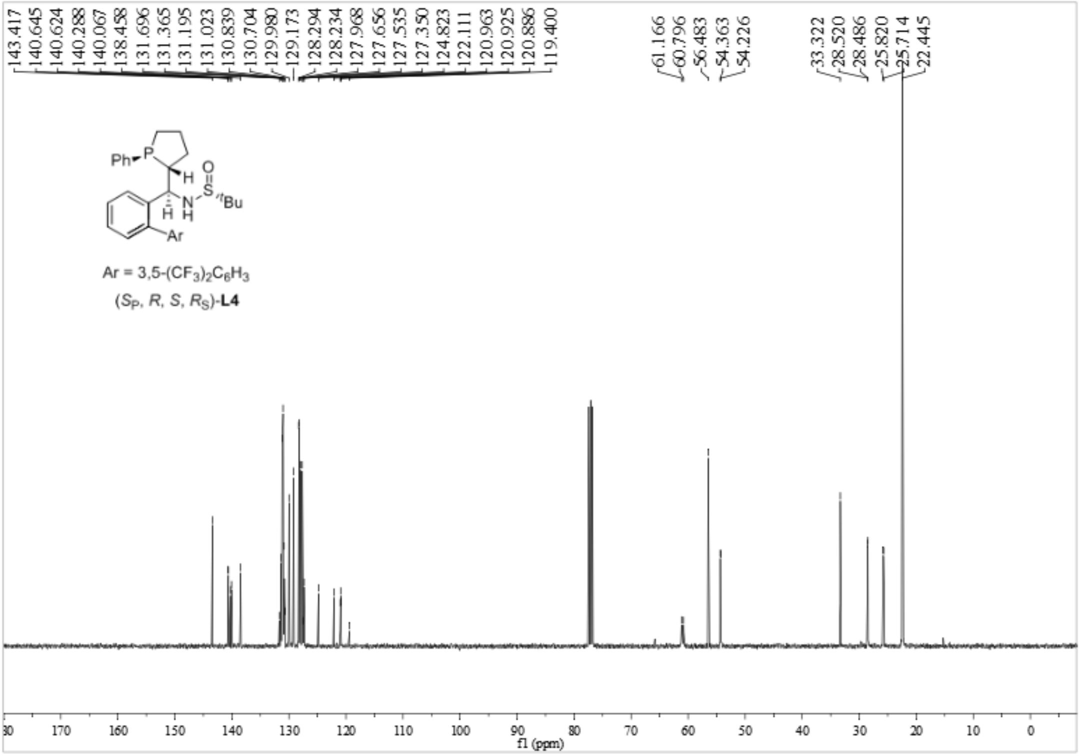
Ar = 3,5-(CF3)2C6H3
(SP, R, S, RS)-L4
f1 (ppm)

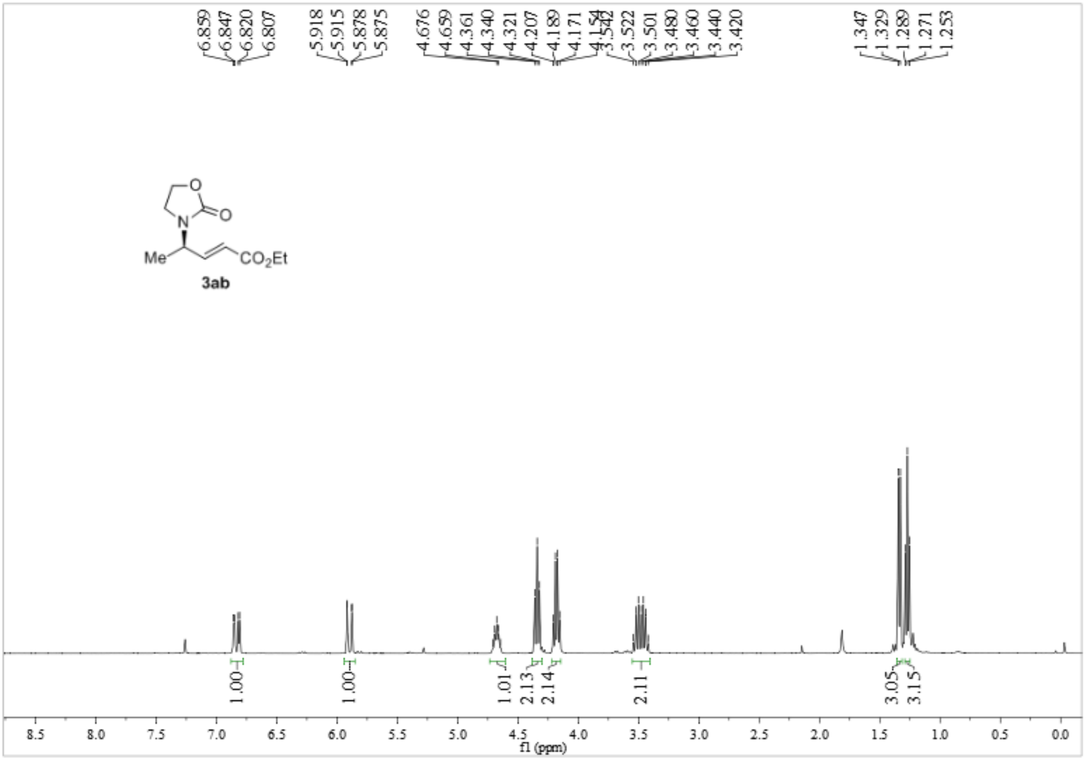
6.859
6.847
6.820
6.807
5.918
5.915
5.878
5.875
4.676
4.659
4.361
4.340
4.321
4.207
4.189
4.171
4.154
3.542
3.522
3.501
3.480
3.460
3.440
3.420
1.347
1.329
1.289
1.271
1.253
Me
N
O
O
CO2Et
3ab
1.00
1.00
1.01
2.13
2.14
2.11
3.05
3.15
f1 (ppm)

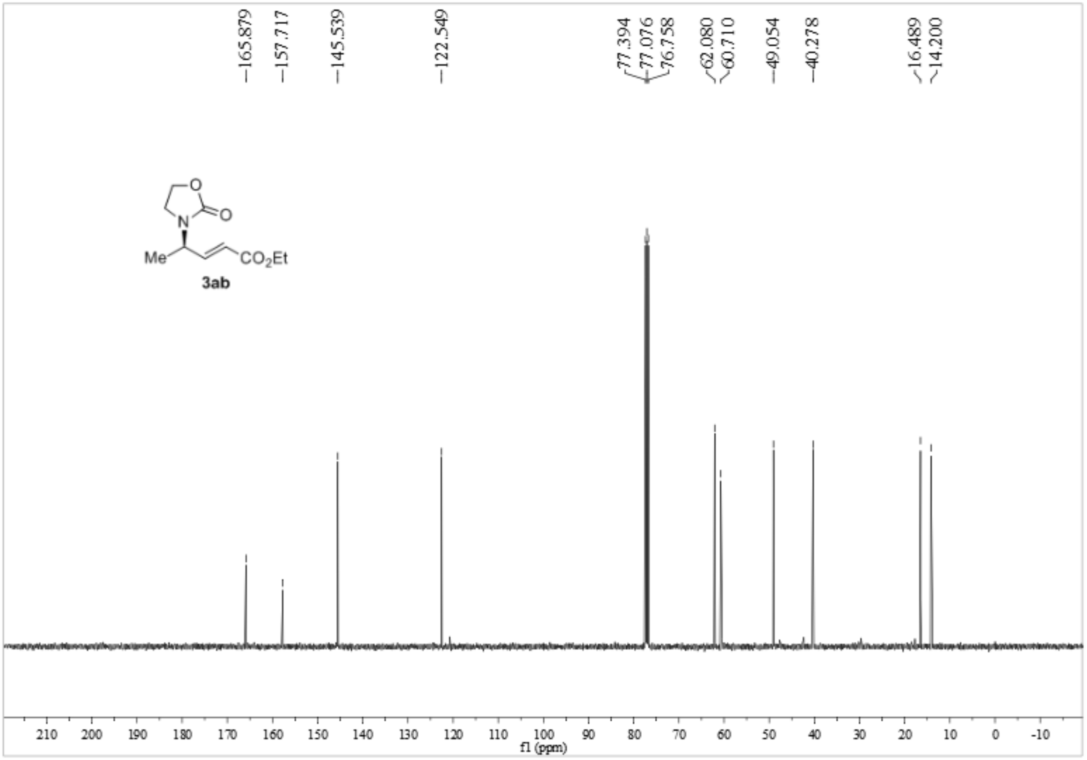
165.879
157.717
145.539
122.549
77.394
77.076
76.758
62.080
60.710
49.054
40.278
16.489
14.200
Me
N
O
O
CO2Et
3ab
f1 (ppm)

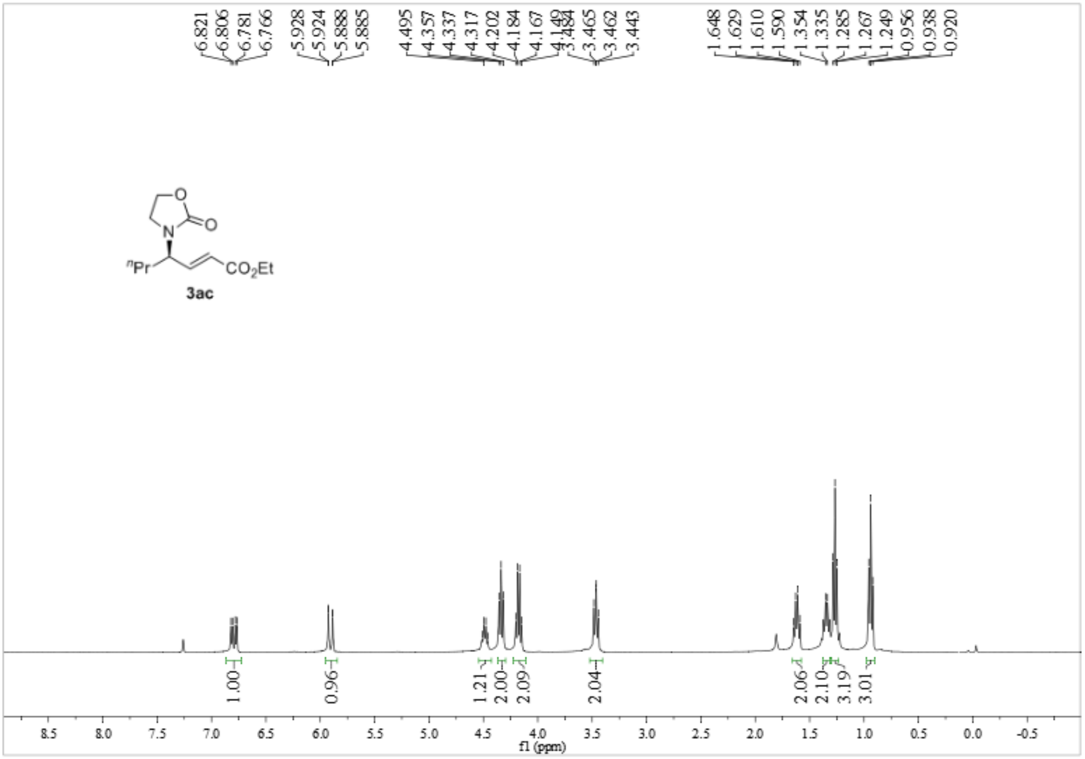
6.821
6.806
6.781
6.766
5.928
5.924
5.888
5.885
4.495
4.357
4.337
4.317
4.202
4.184
4.167
4.149
3.484
3.465
3.462
3.443
1.648
1.629
1.610
1.590
1.354
1.335
1.285
1.267
1.249
0.956
0.938
0.920
3ac
1.00
0.96
1.21
2.00
2.09
2.04
2.06
2.10
3.19
3.01
f1 (ppm)

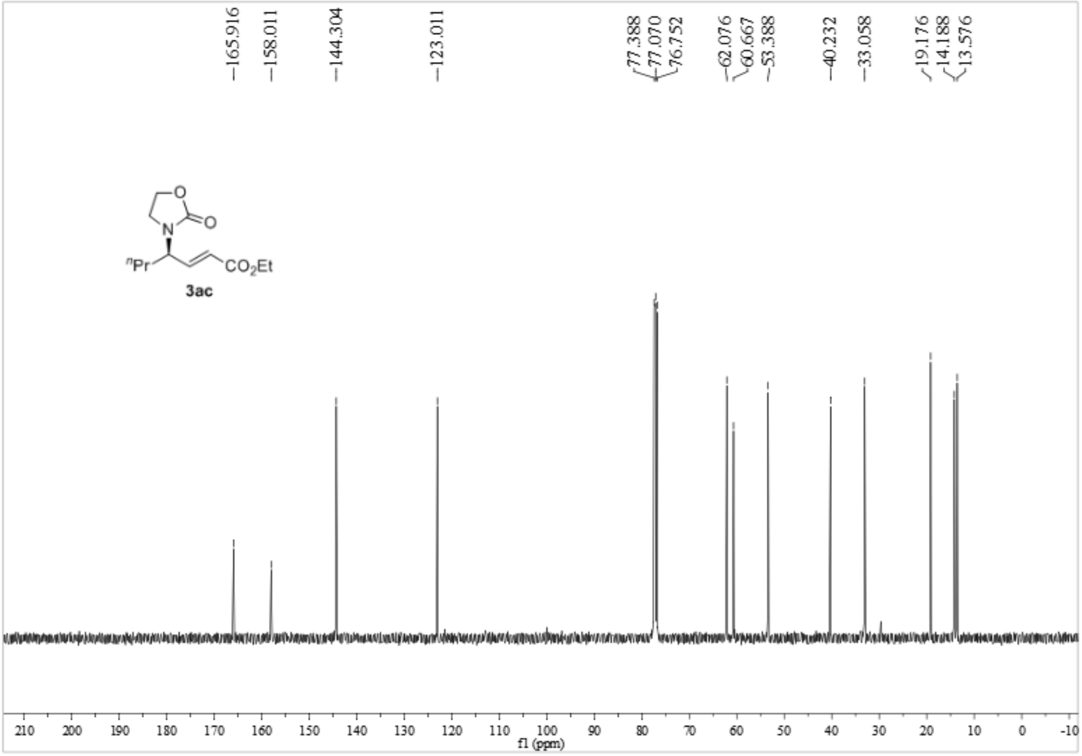
165.916
158.011
144.304
123.011
77.388
77.070
76.752
62.076
60.667
53.388
40.232
33.058
19.176
14.188
13.576
3ac
f1 (ppm)

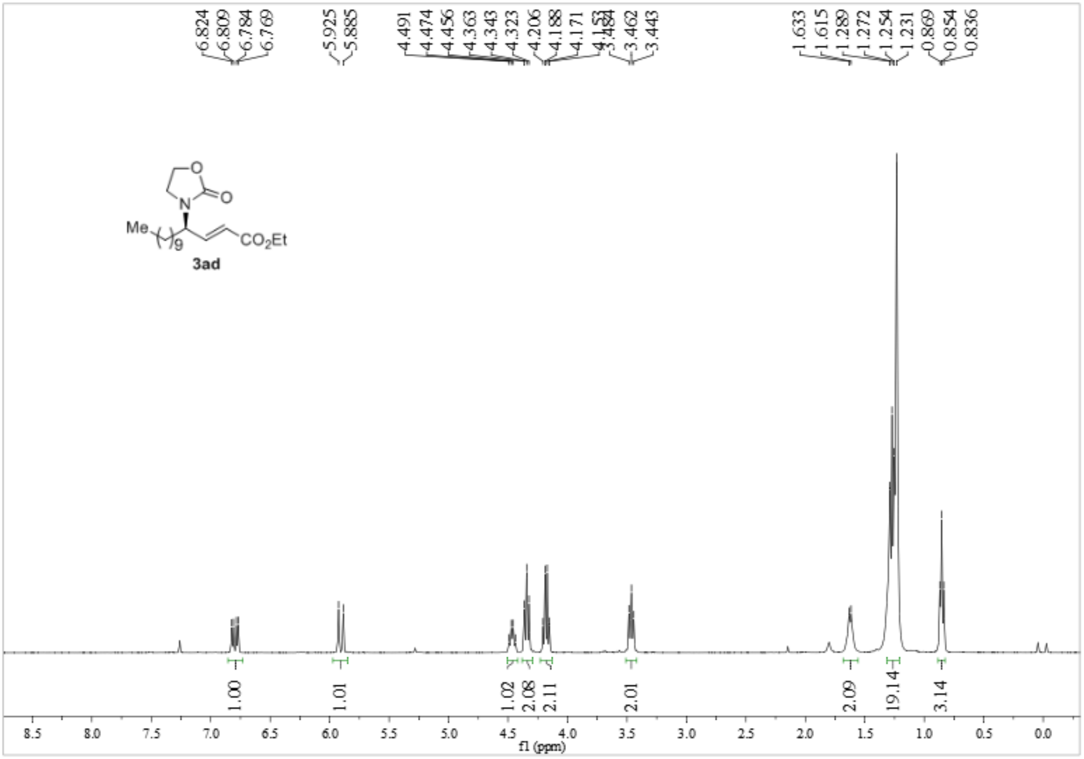

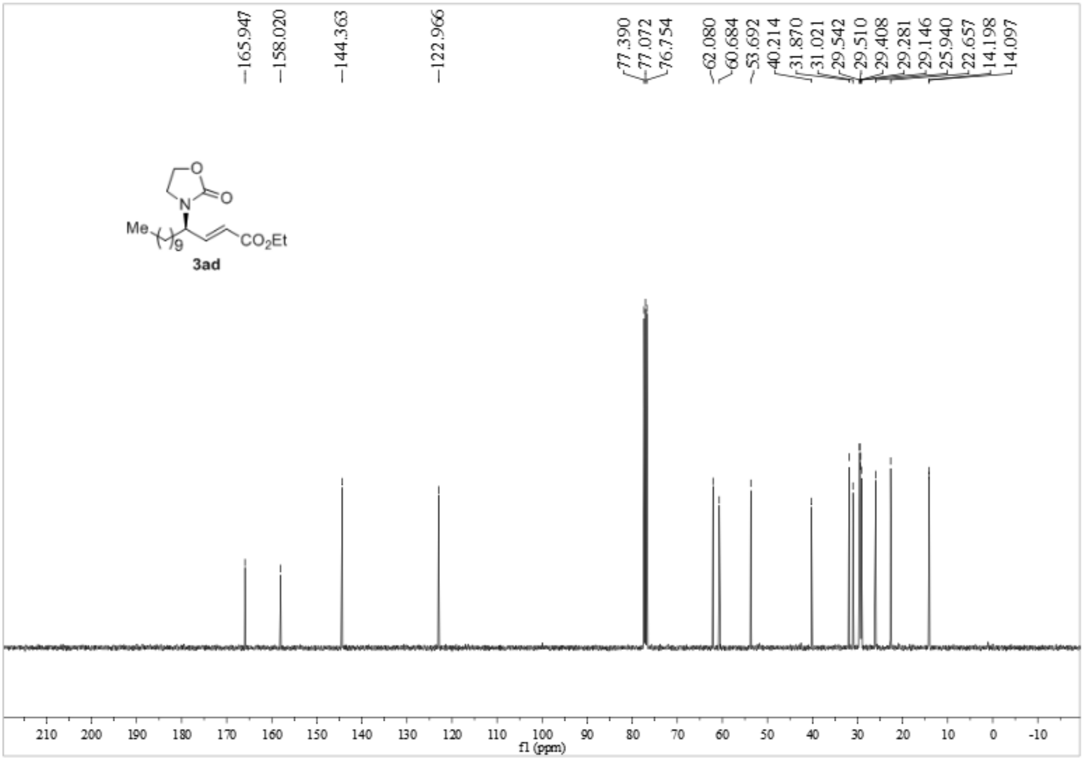

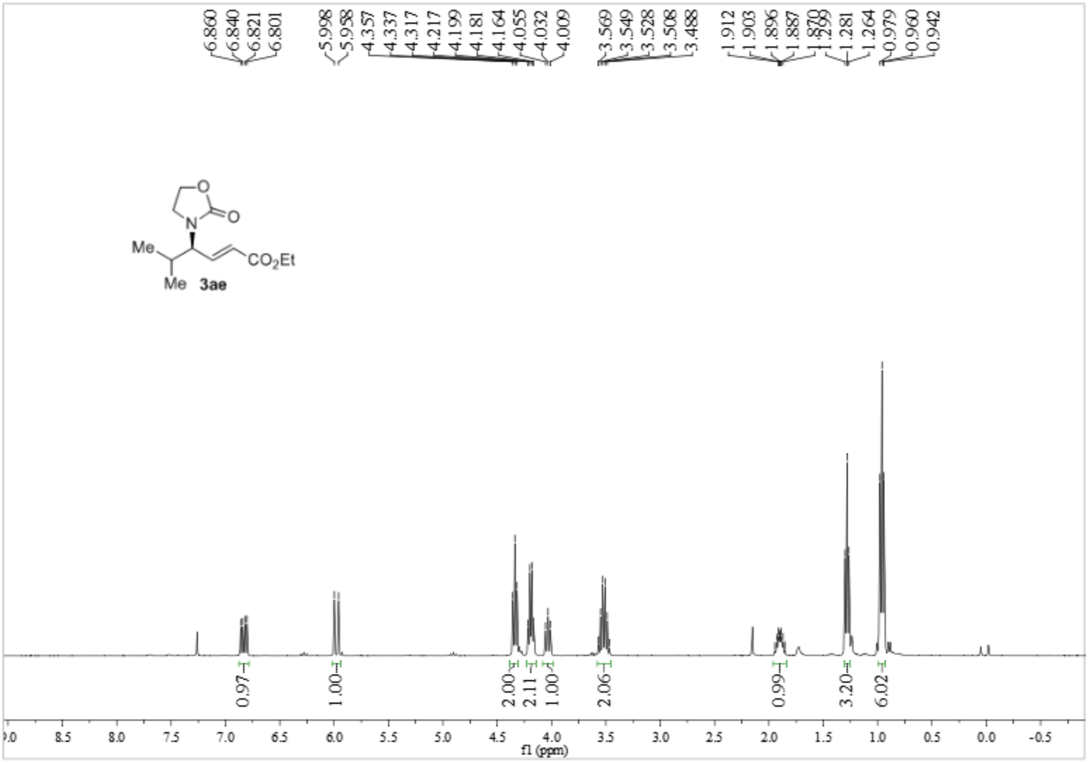

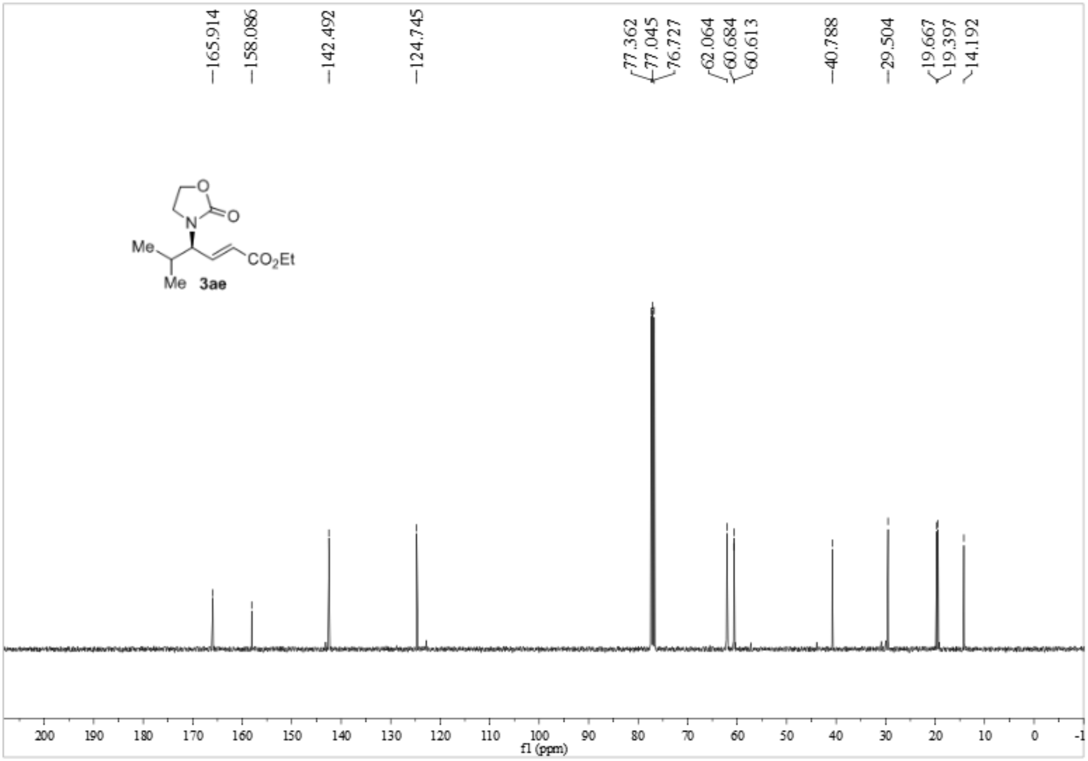

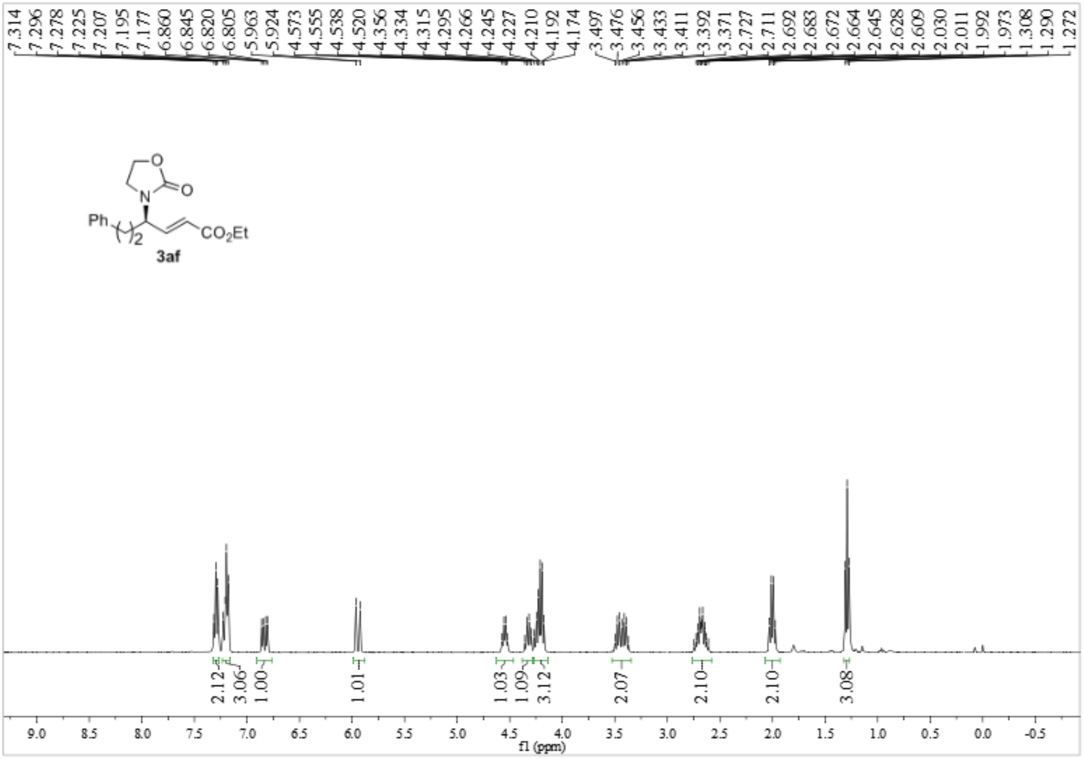
7.314 7.296 7.278 7.225 7.207 7.195 7.177 6.860 6.845 6.820 6.805 5.963 5.924 4.573 4.555 4.538 4.520 4.356 4.334 4.315 4.295 4.266 4.245 4.227 4.210 4.192 4.174 3.497 3.476 3.456 3.433 3.411 3.392 3.371 2.727 2.711 2.692 2.683 2.672 2.664 2.645 2.628 2.609 2.030 2.011 1.992 1.973 1.308 1.290 1.272
Ph
N
O
O
CO_2Et
2
3af
2.12 3.06 1.00 1.01 1.03 1.09 3.12 2.07 2.10 2.10 3.08
9.0 8.5 8.0 7.5 7.0 6.5 6.0 5.5 5.0 4.5 4.0 3.5 3.0 2.5 2.0 1.5 1.0 0.5 0.0 -0.5
f1 (ppm)

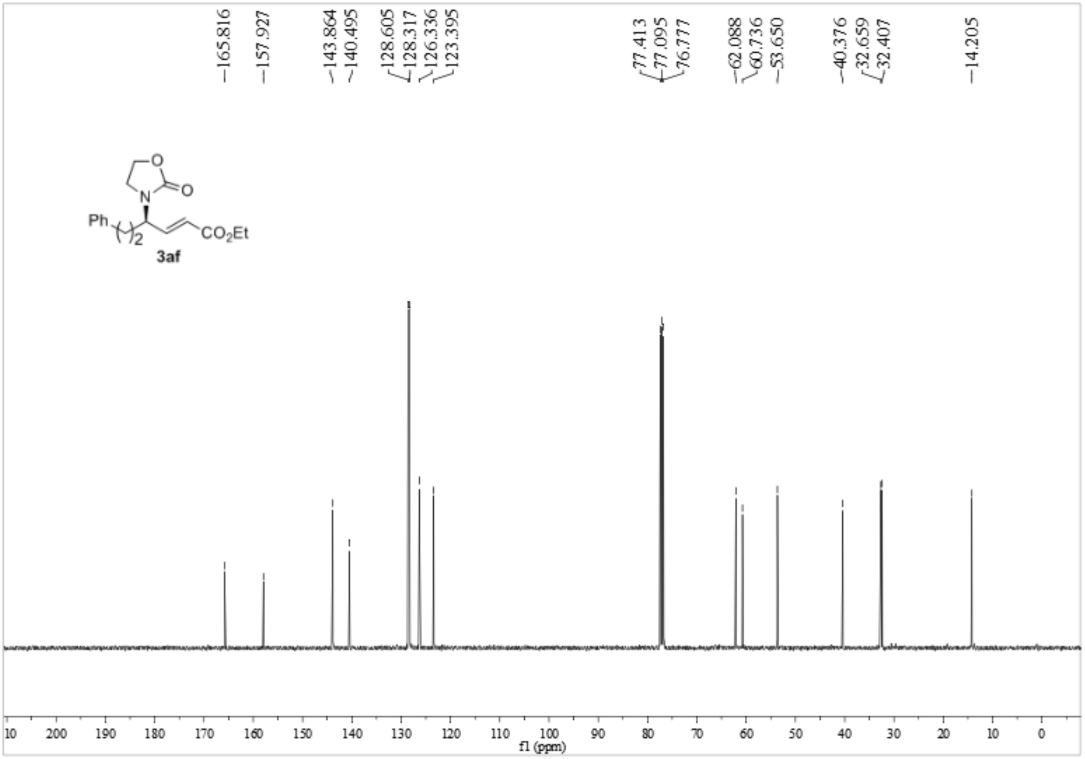
165.816 157.927 143.864 140.495 128.605 128.317 126.336 123.395 77.413 77.095 76.777 62.088 60.736 53.650 40.376 32.659 32.407 14.205
Ph
N
O
O
CO_2Et
2
3af
10 200 190 180 170 160 150 140 130 120 110 100 90 80 70 60 50 40 30 20 10 0
f1 (ppm)

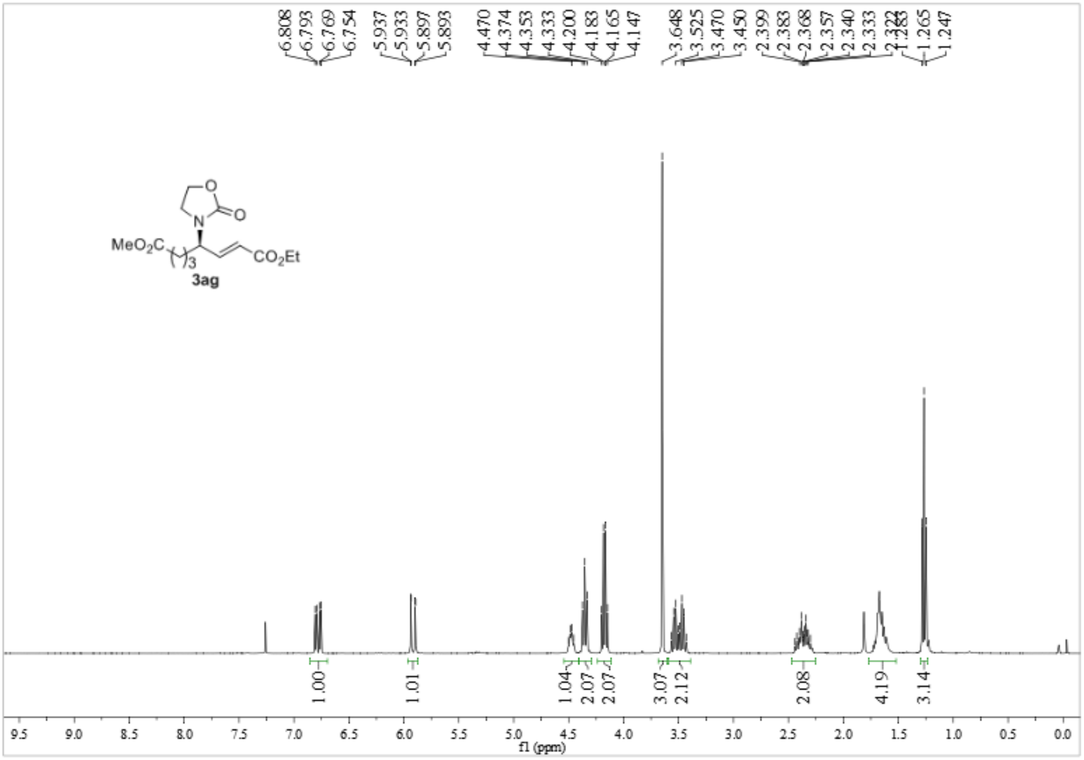
6.808
6.793
6.769
6.754
5.937
5.933
5.897
5.893
4.470
4.374
4.353
4.333
4.200
4.183
4.165
4.147
3.648
3.525
3.470
3.450
2.399
2.383
2.368
2.357
2.340
2.333
2.322
1.265
1.247
MeO2C
CO2Et
N
O
O
3
3ag
1.00
1.01
1.04
2.07
2.07
3.07
2.12
2.08
4.19
3.14
f1 (ppm)

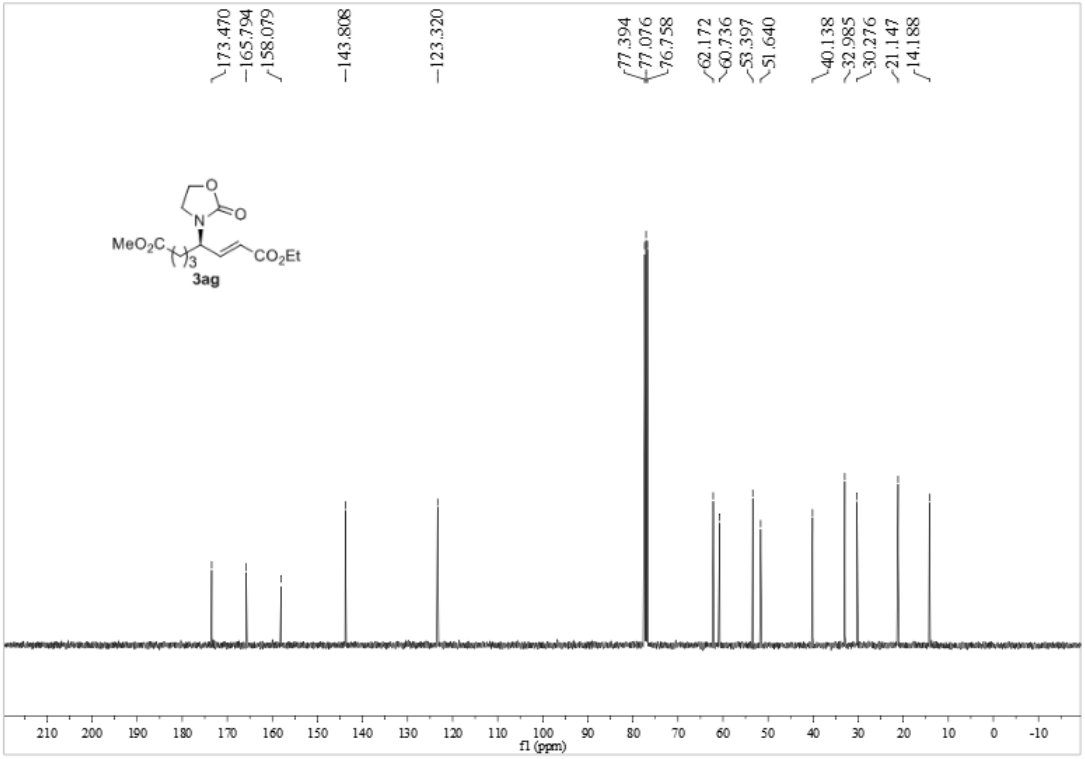
173.470
165.794
158.079
143.808
123.320
77.394
77.076
76.758
62.172
60.736
53.397
51.640
40.138
32.985
30.276
21.147
14.188
MeO2C
CO2Et
N
O
O
3
3ag
f1 (ppm)

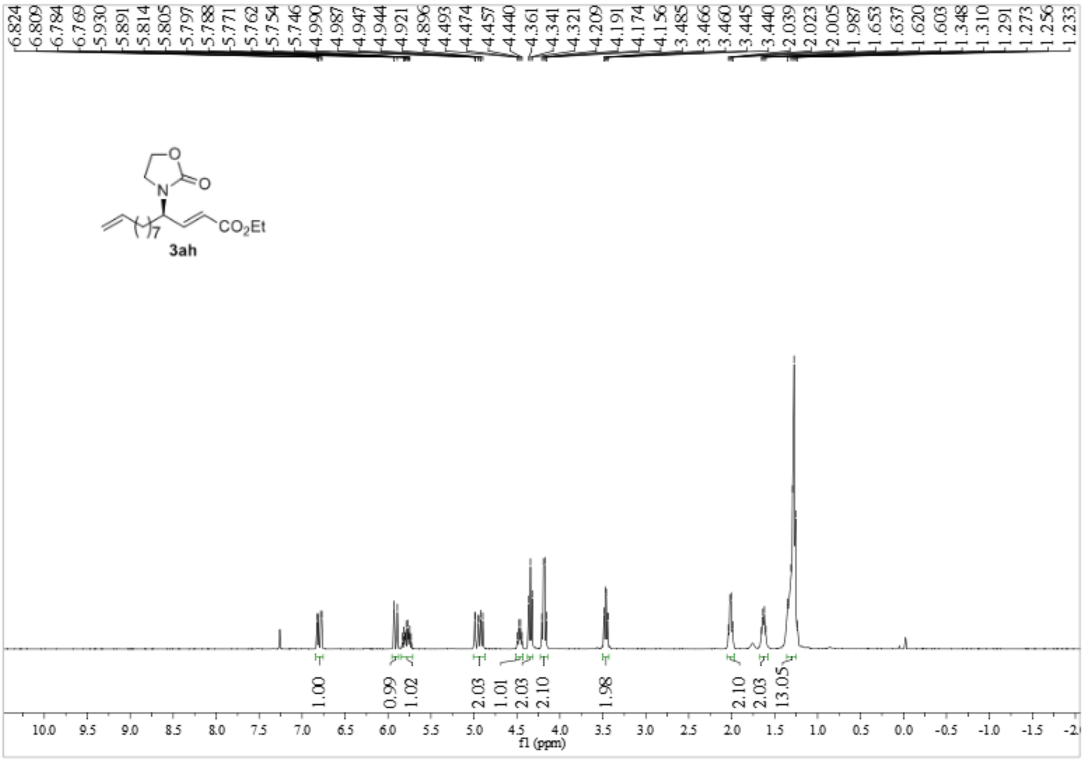

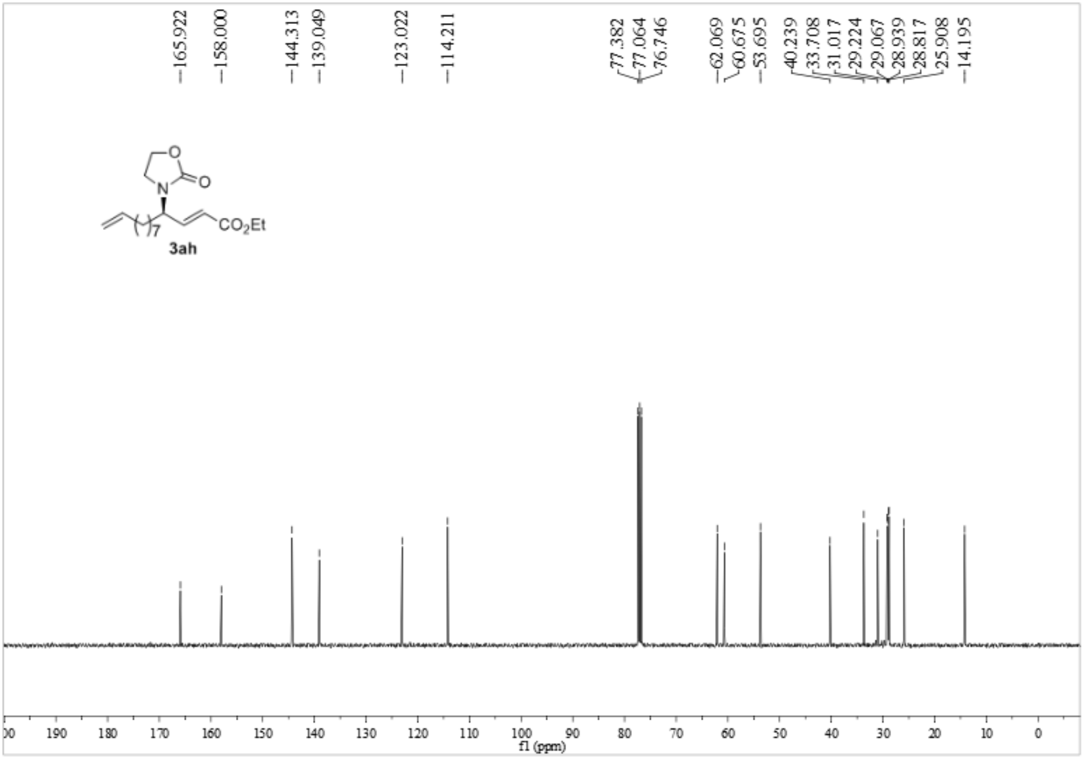

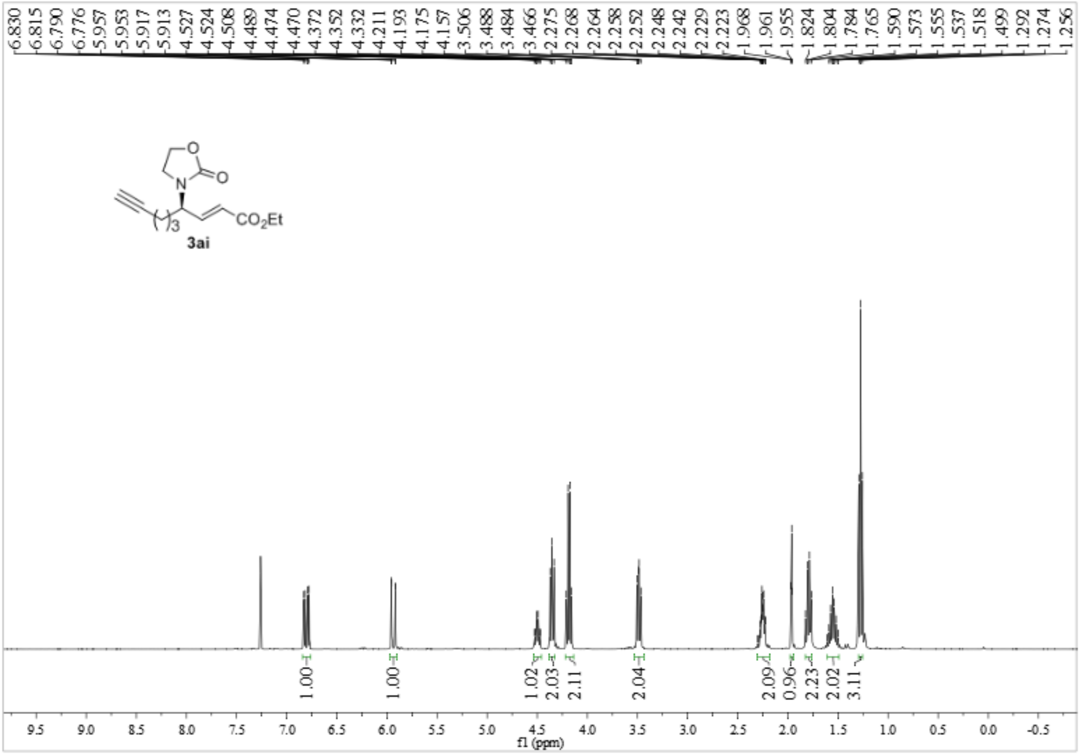

6.830
6.815
6.790
6.776
5.957
5.953
5.917
5.913
4.527
4.524
4.508
4.489
4.474
4.470
4.372
4.352
4.332
4.211
4.193
4.175
4.157
3.506
3.488
3.484
3.466
2.275
2.268
2.264
2.258
2.252
2.248
2.242
2.229
2.223
1.968
1.961
1.955
1.824
1.804
1.784
1.765
1.590
1.573
1.555
1.537
1.518
1.499
1.292
1.274
1.256
CO2Et
3ai
1.00
1.00
1.02
2.03
2.11
2.04
2.09
0.96
2.23
2.02
3.11
f1 (ppm)

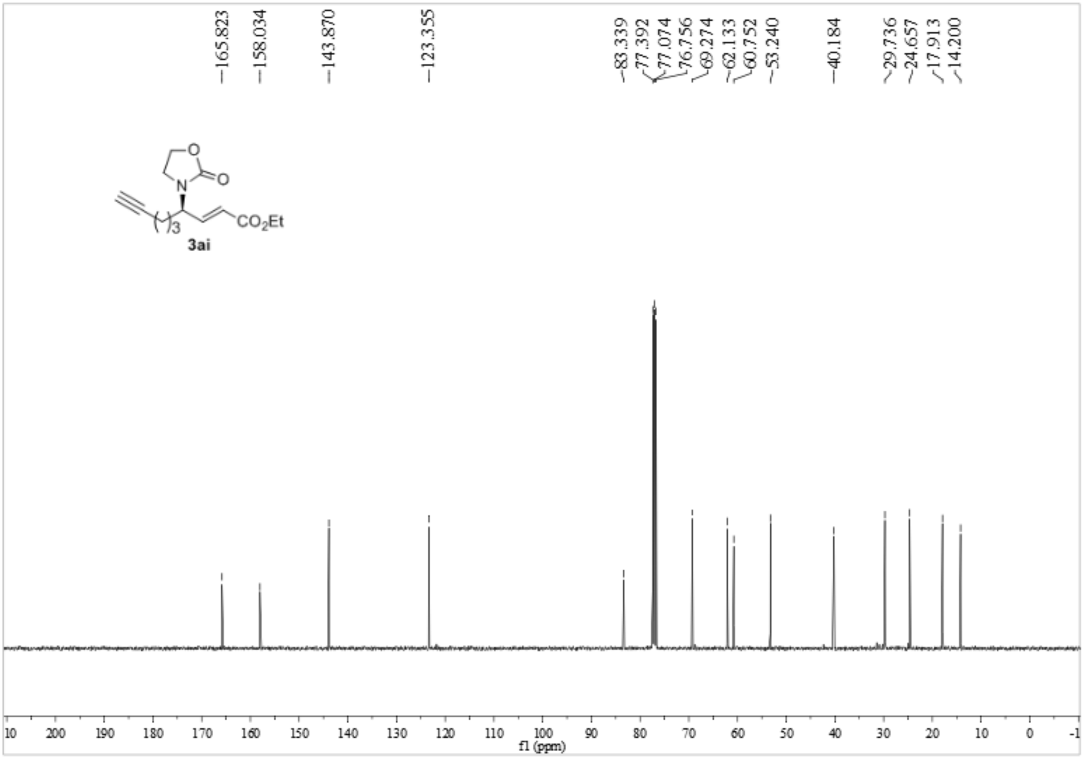

165.823
158.034
143.870
123.355
83.339
77.392
77.074
76.756
69.274
62.133
60.752
53.240
40.184
29.736
24.657
17.913
14.200
CO2Et
3ai
f1 (ppm)

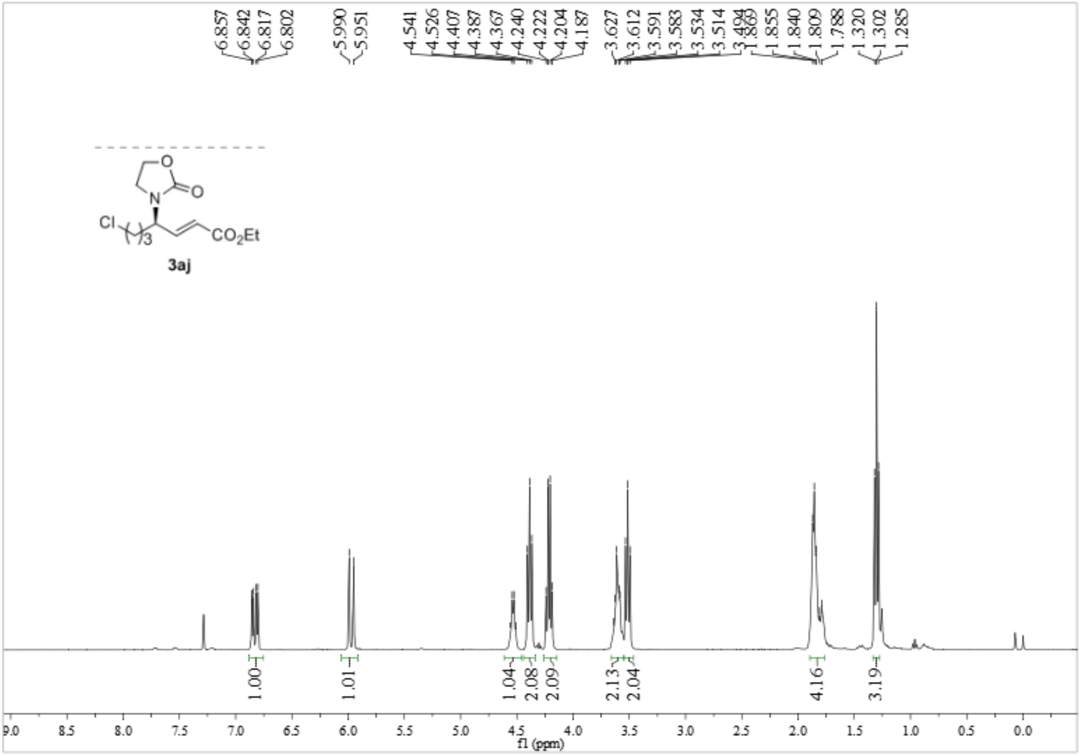

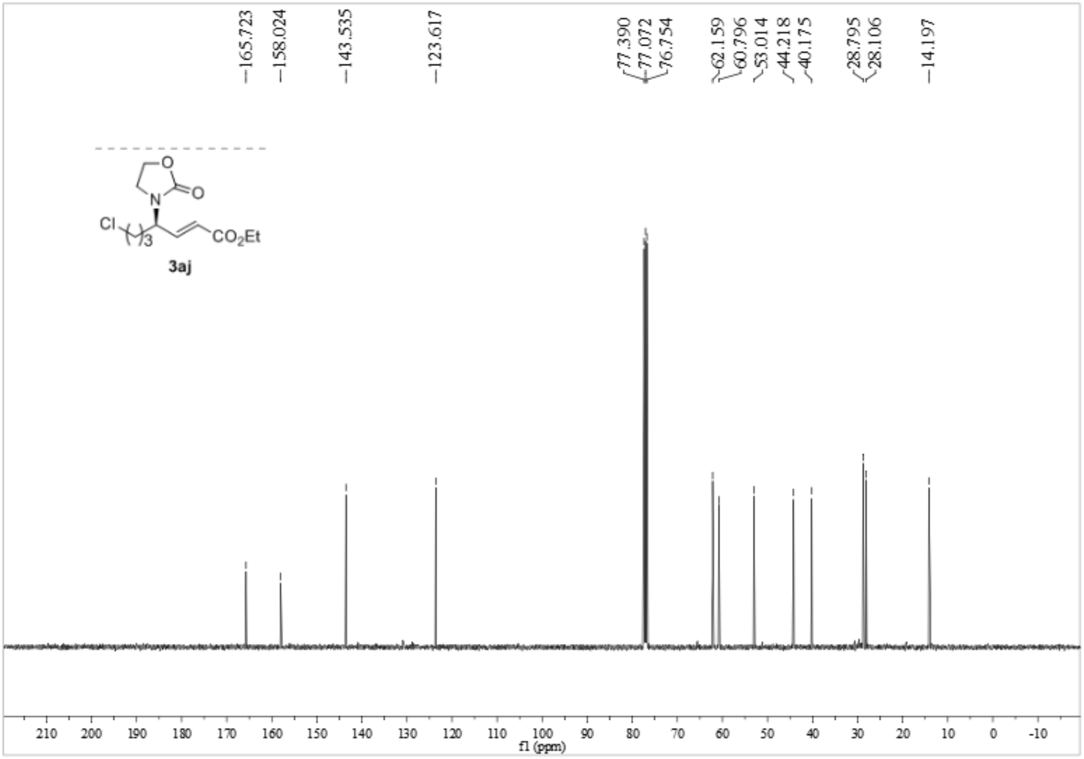

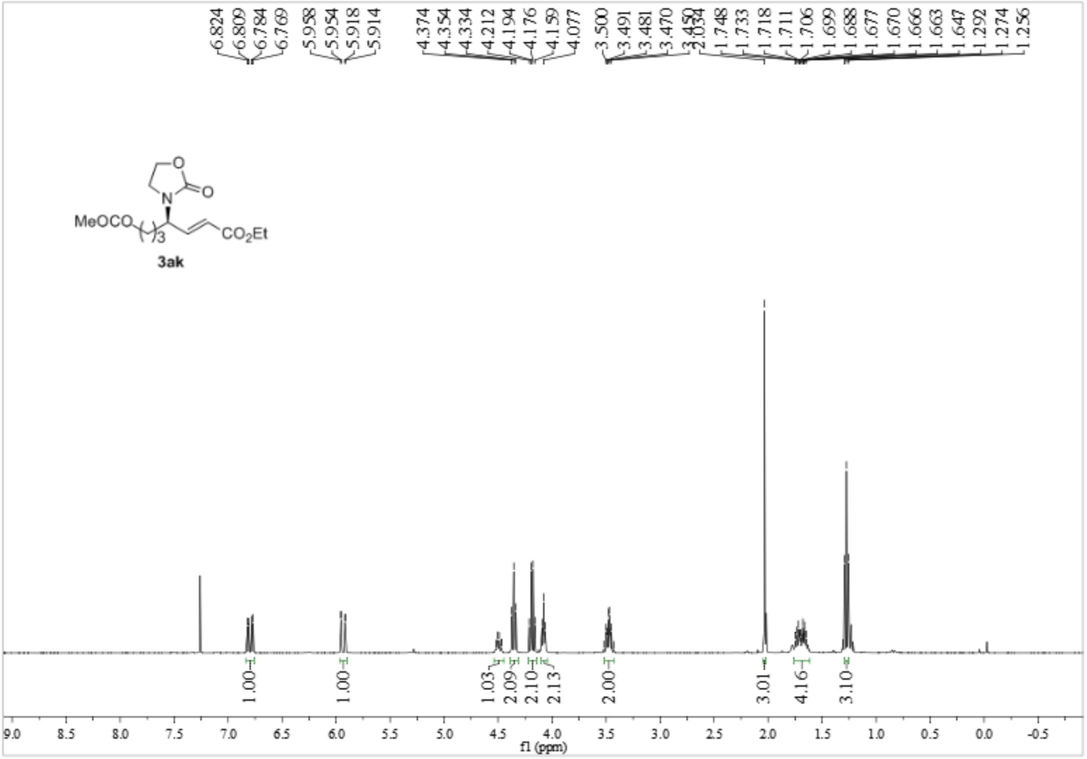

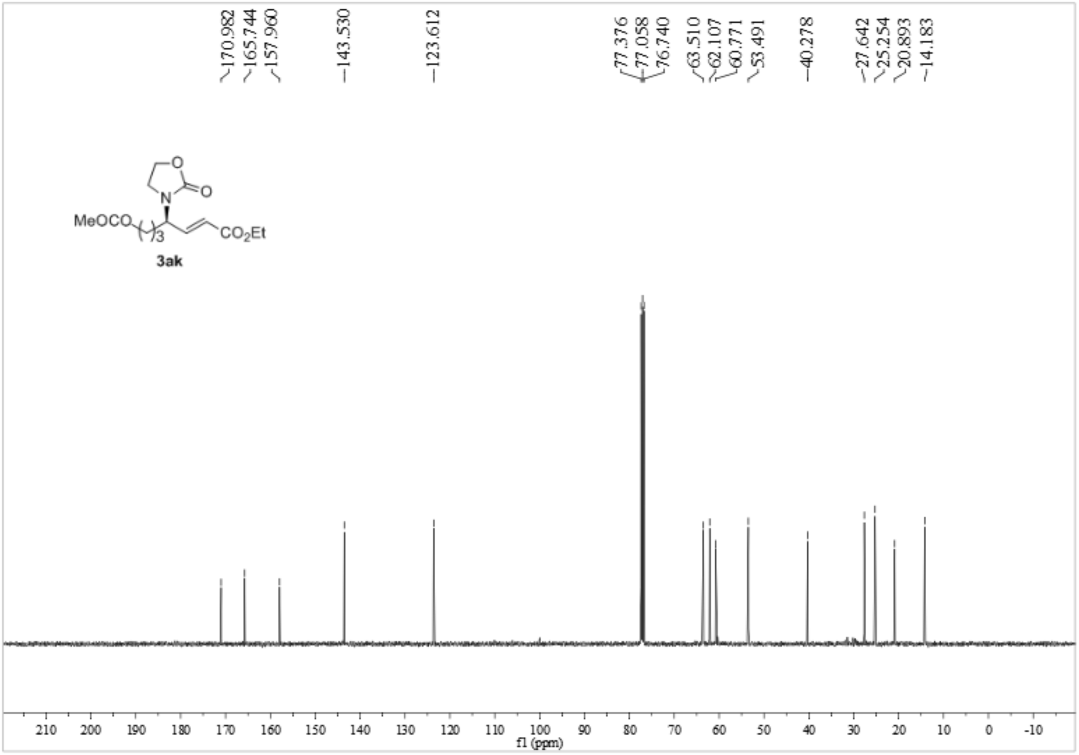

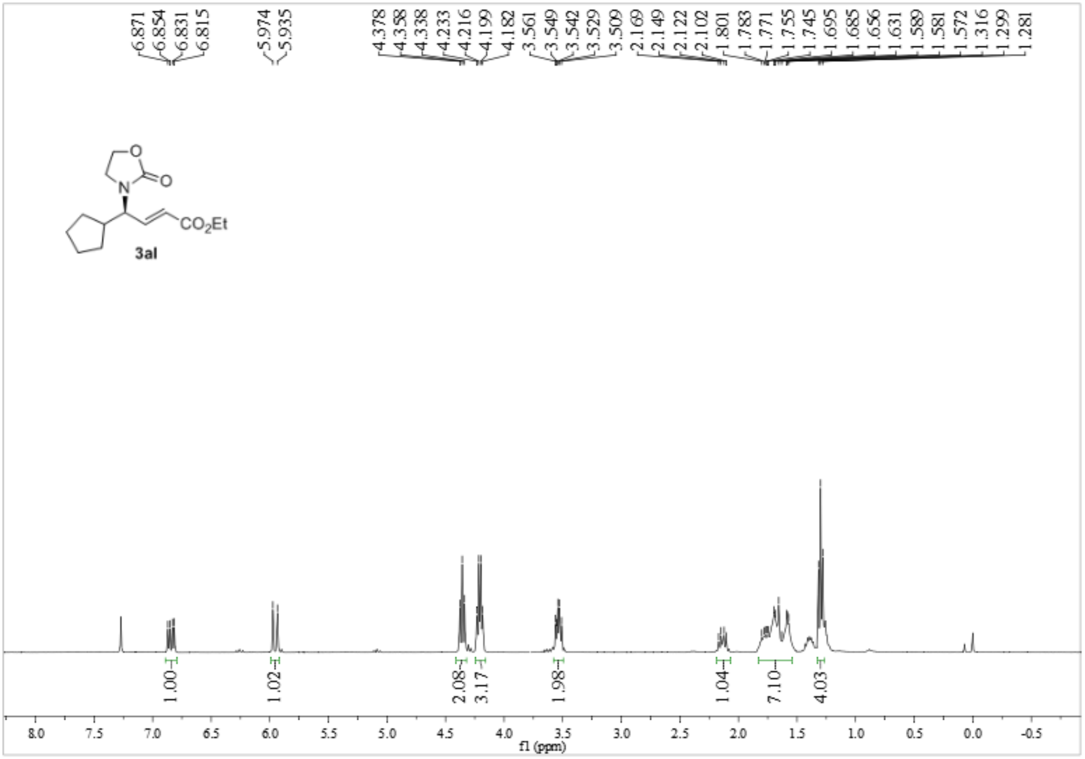
6.871
6.854
6.831
6.815
5.974
5.935
4.378
4.358
4.338
4.233
4.216
4.199
4.182
3.561
3.549
3.542
3.529
3.509
2.169
2.149
2.122
2.102
1.801
1.783
1.771
1.755
1.745
1.695
1.685
1.656
1.631
1.589
1.581
1.572
1.316
1.299
1.281
O
N
O
CO2Et
3al
1.00
1.02
2.08
3.17
1.98
1.04
7.10
4.03
f1 (ppm)

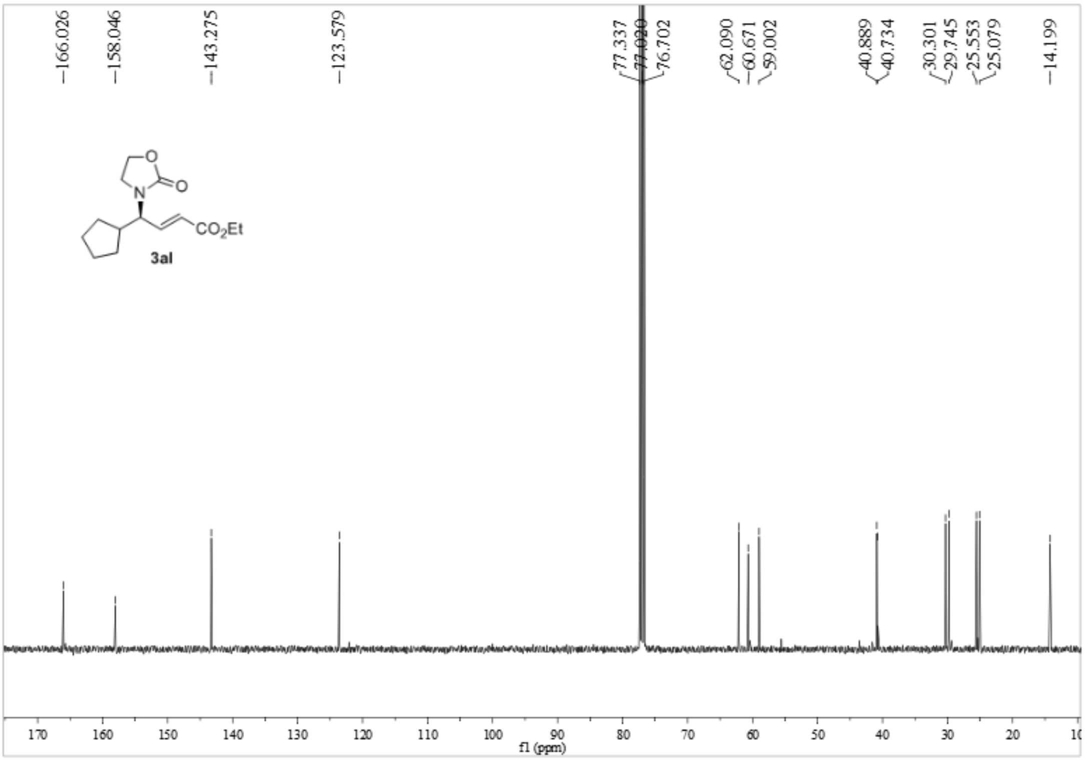
166.026
158.046
143.275
123.579
77.337
77.020
76.702
62.090
60.671
59.002
40.889
40.734
30.301
29.745
25.553
25.079
14.199
O
N
O
CO2Et
3al
f1 (ppm)

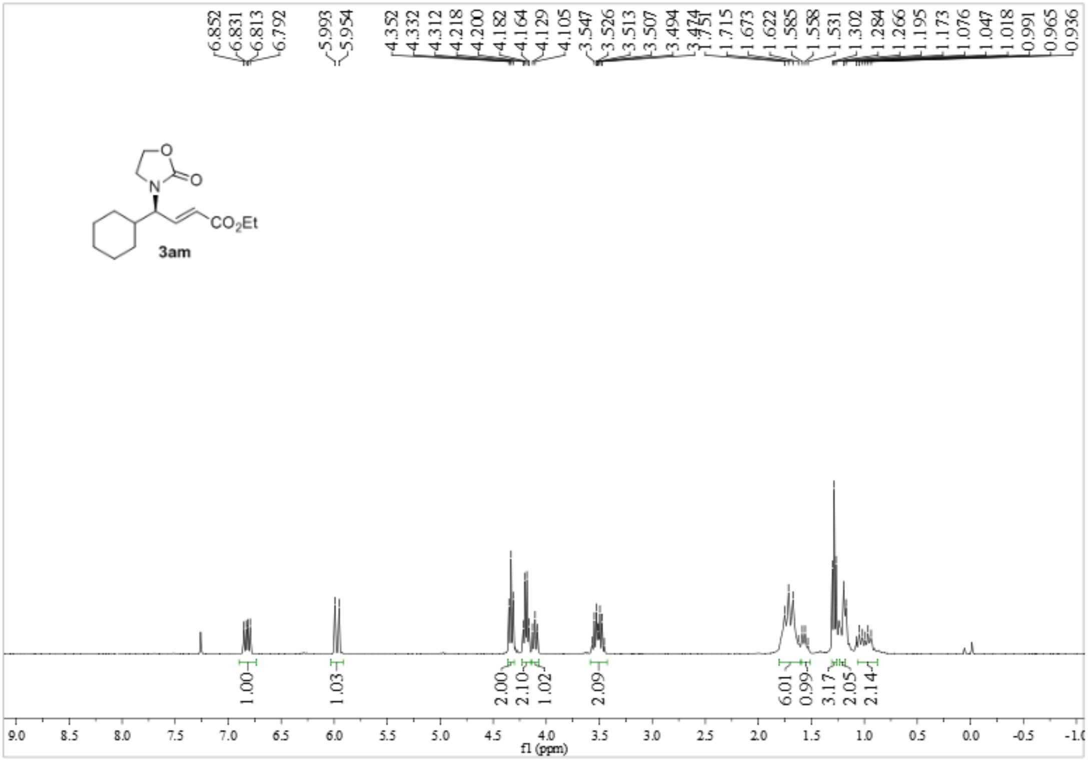

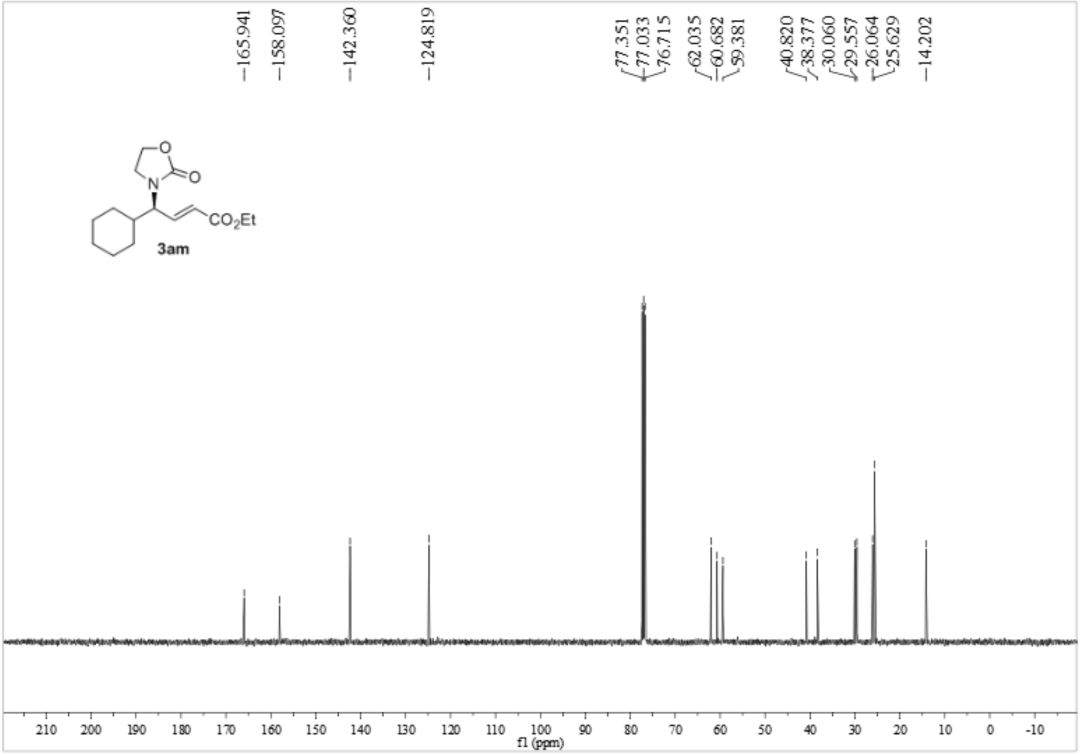

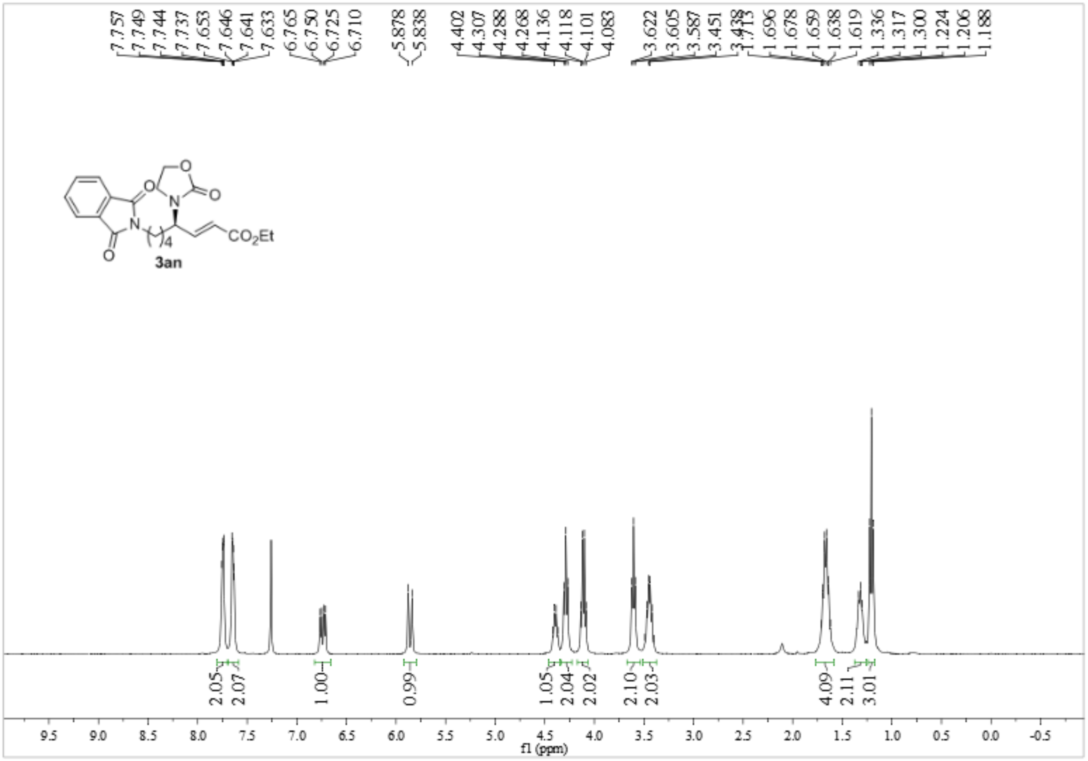
3an
f1 (ppm)

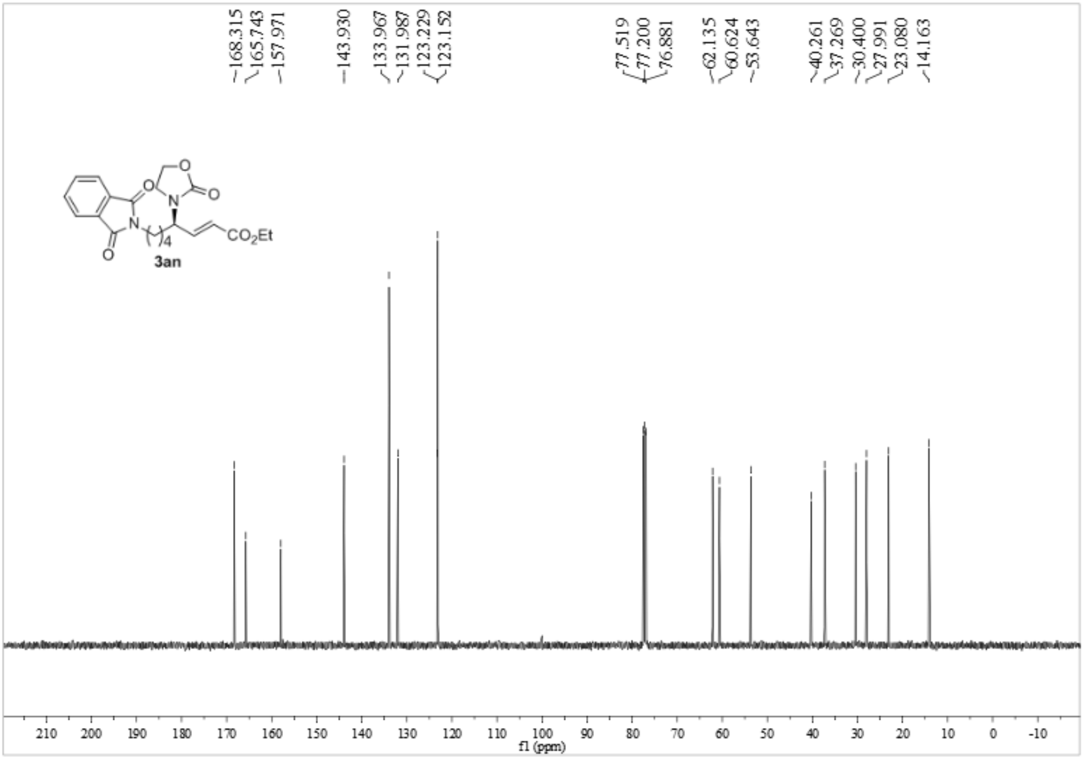
3an
f1 (ppm)

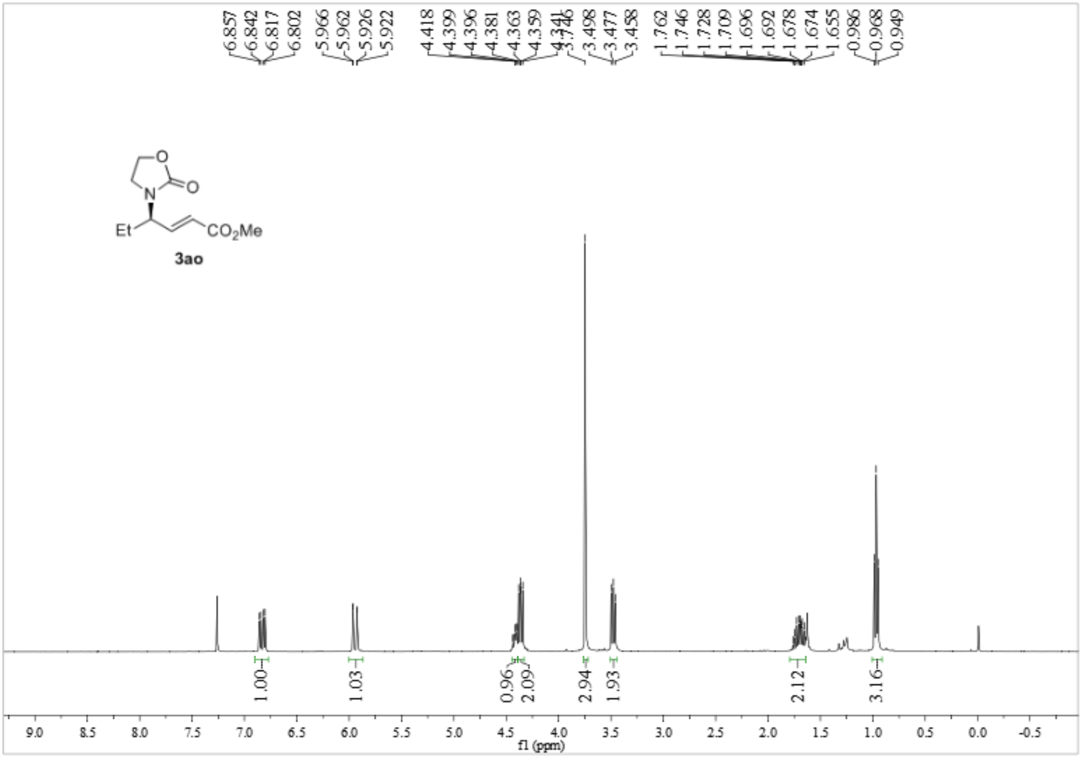
6.857
6.842
6.817
6.802
5.966
5.962
5.926
5.922
4.418
4.399
4.396
4.381
4.363
4.359
4.341
3.746
3.498
3.477
3.458
1.762
1.746
1.728
1.709
1.696
1.692
1.678
1.674
1.655
0.986
0.968
0.949
Et
CO2Me
3ao
1.00
1.03
0.96
2.09
2.94
1.93
2.12
3.16
f1 (ppm)

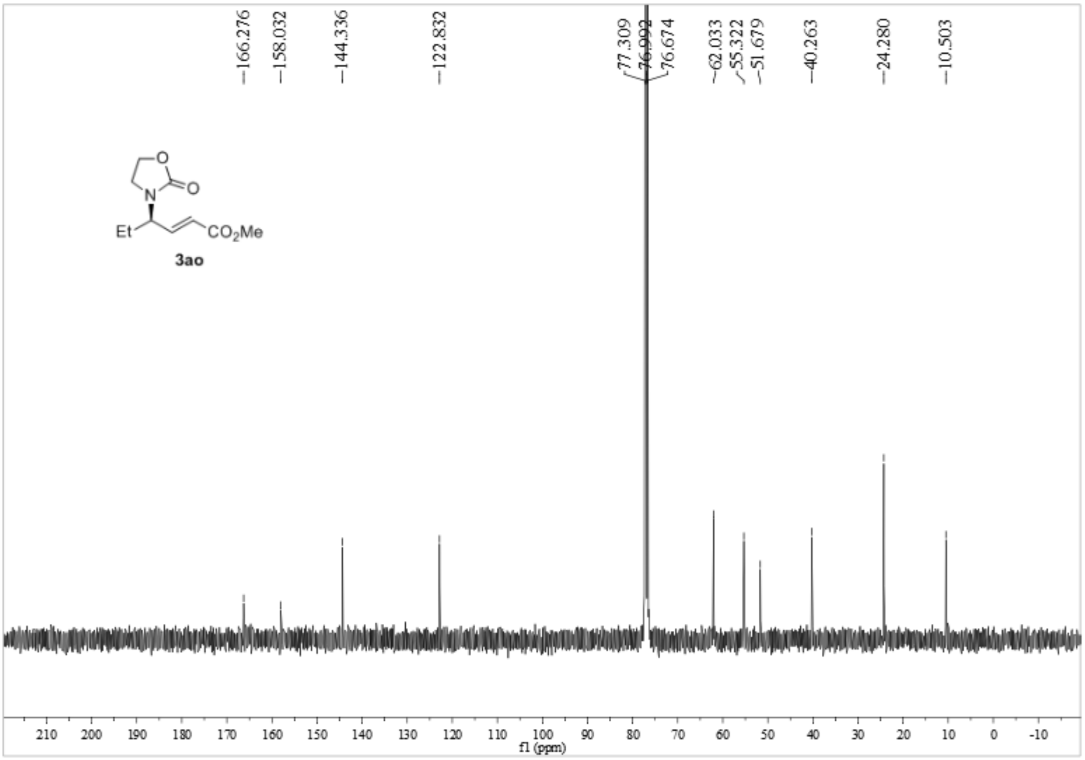
166.276
158.032
144.336
122.832
77.309
76.992
76.674
62.033
55.322
51.679
40.263
24.280
10.503
Et
CO2Me
3ao
f1 (ppm)

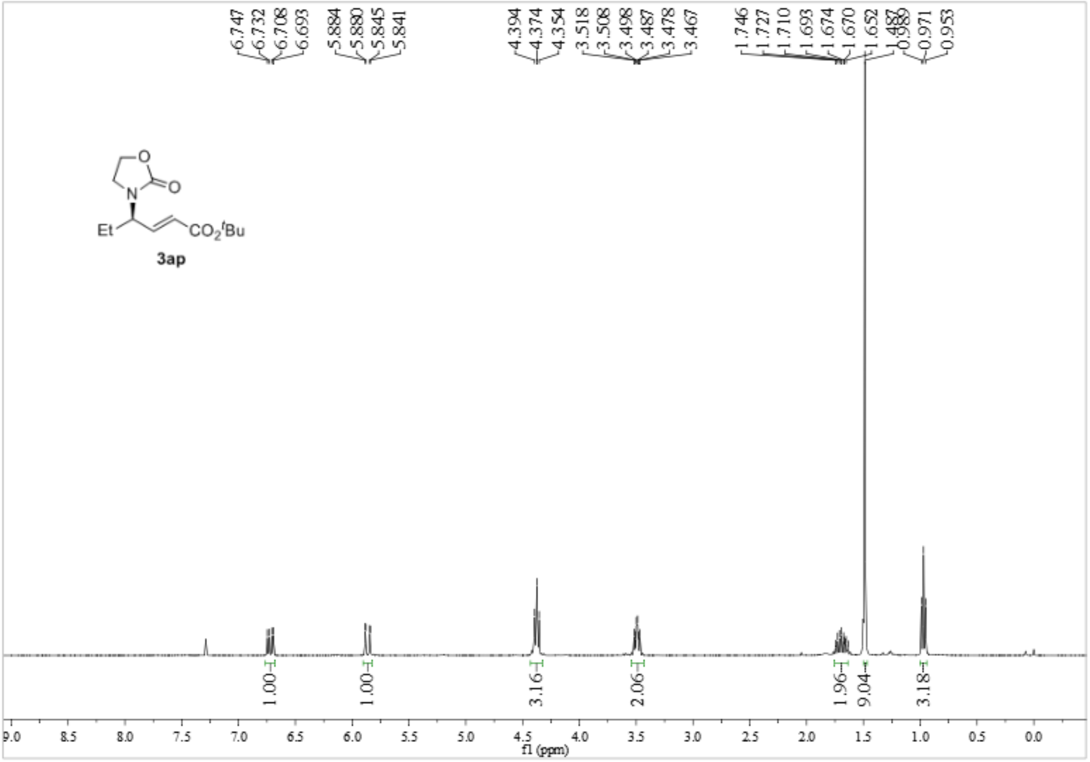
6.747
6.732
6.708
6.693
5.884
5.880
5.845
5.841
4.394
4.374
4.354
3.518
3.508
3.498
3.487
3.478
3.467
1.746
1.727
1.710
1.693
1.674
1.670
1.652
1.487
0.971
0.953
3ap
1.00
1.00
3.16
2.06
1.96
9.04
3.18
f1 (ppm)

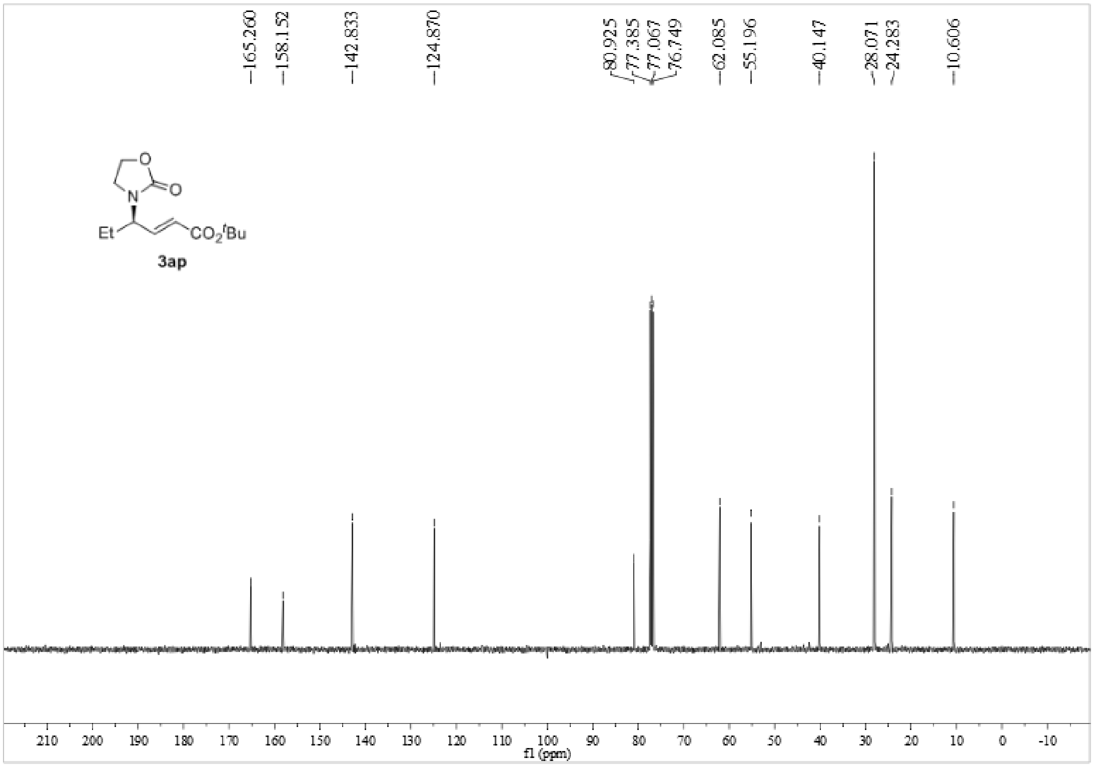
165.260
158.152
142.833
124.870
80.925
77.385
77.067
76.749
62.085
55.196
40.147
28.071
24.283
10.606
3ap
f1 (ppm)

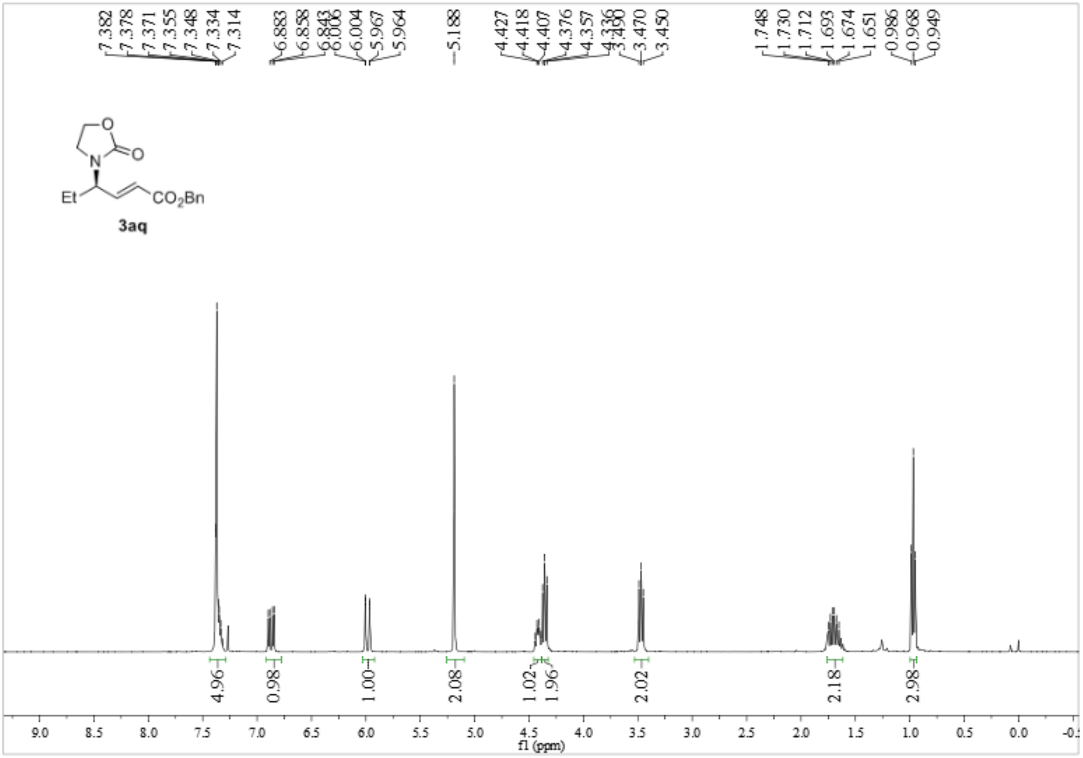
7.382
7.378
7.371
7.355
7.348
7.334
7.314
6.883
6.858
6.843
6.006
6.004
5.967
5.964
5.188
4.427
4.418
4.407
4.376
4.357
4.336
3.490
3.470
3.450
1.748
1.730
1.712
1.693
1.674
1.651
0.986
0.968
0.949
Et
CO_2Bn
3aq
4.96
0.98
1.00
2.08
1.02
1.96
2.02
2.18
2.98
f1 (ppm)

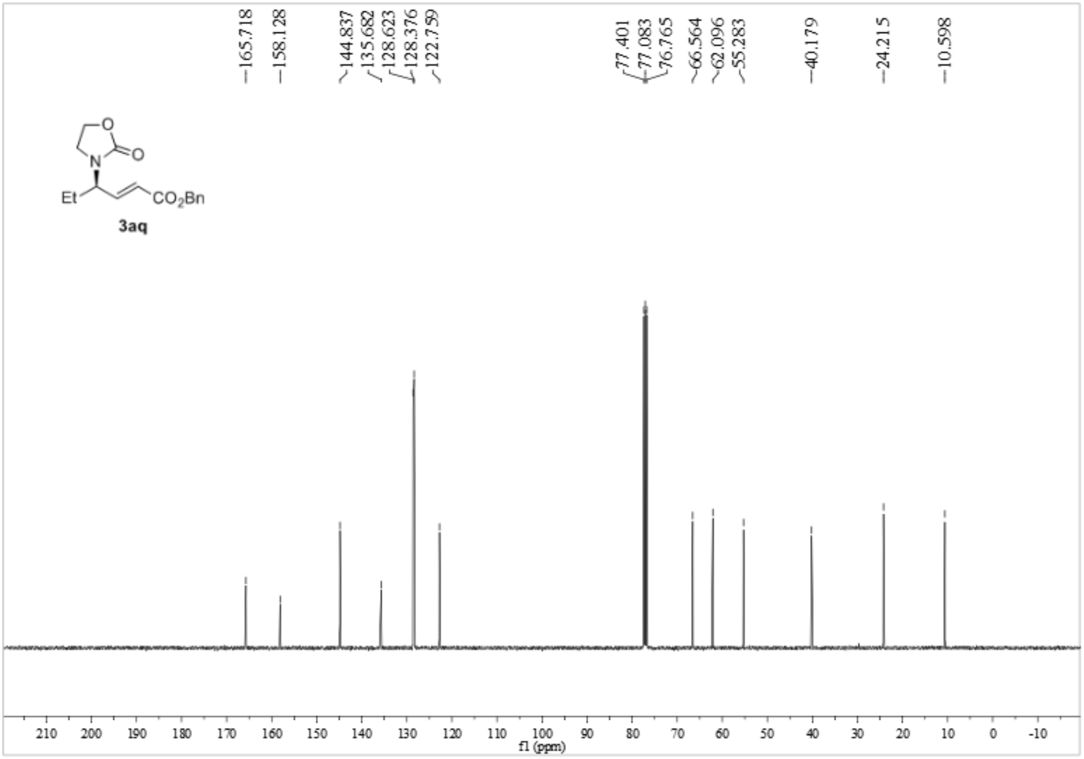
165.718
158.128
144.837
135.682
128.623
128.376
122.759
77.401
77.083
76.765
66.564
62.096
55.283
40.179
24.215
10.598
Et
CO_2Bn
3aq
f1 (ppm)

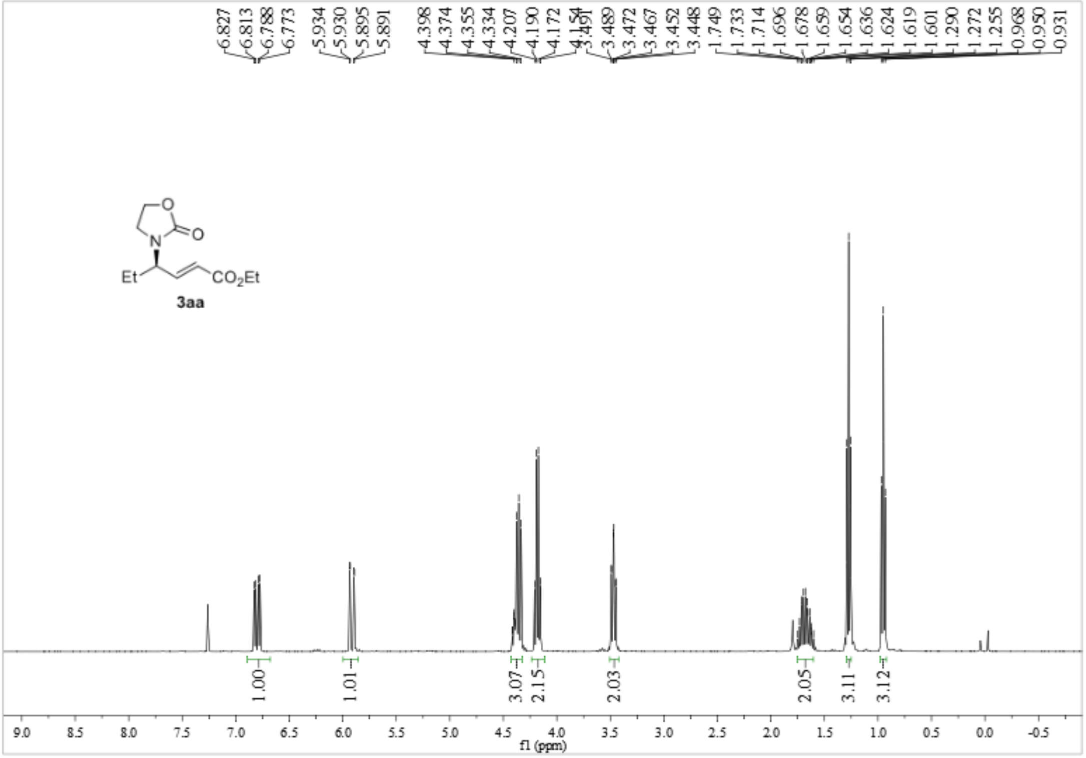
3aa
CO2Et
Et
f1 (ppm)

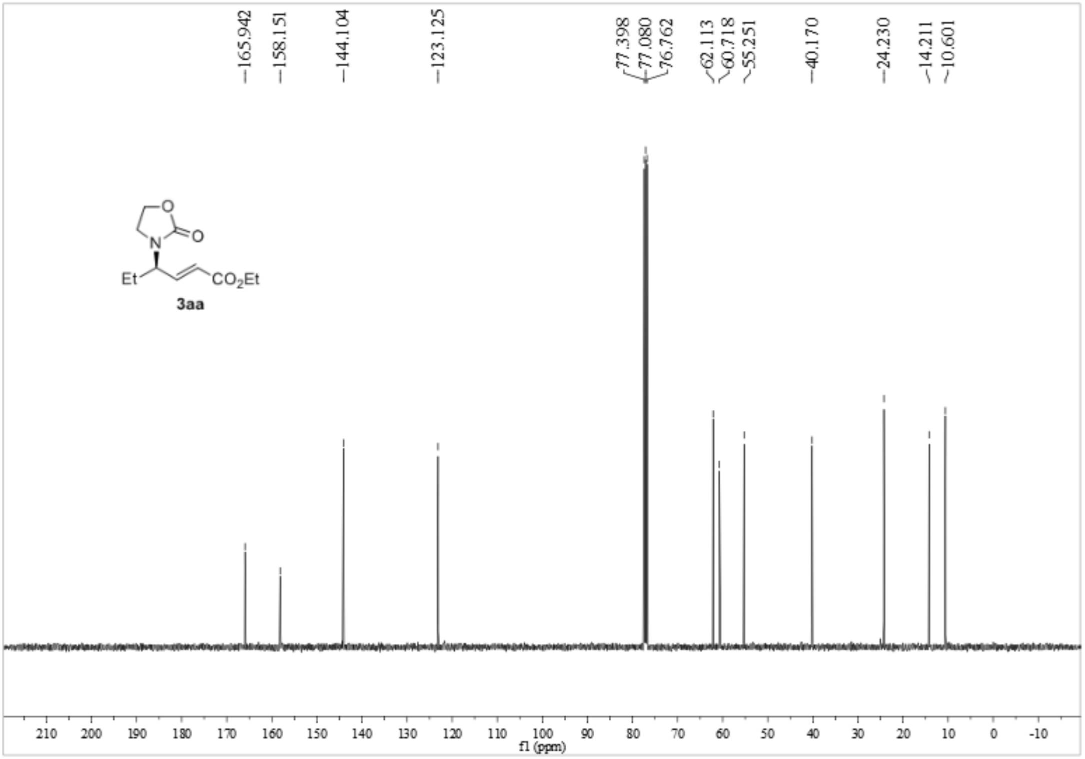
3aa
CO2Et
Et
f1 (ppm)

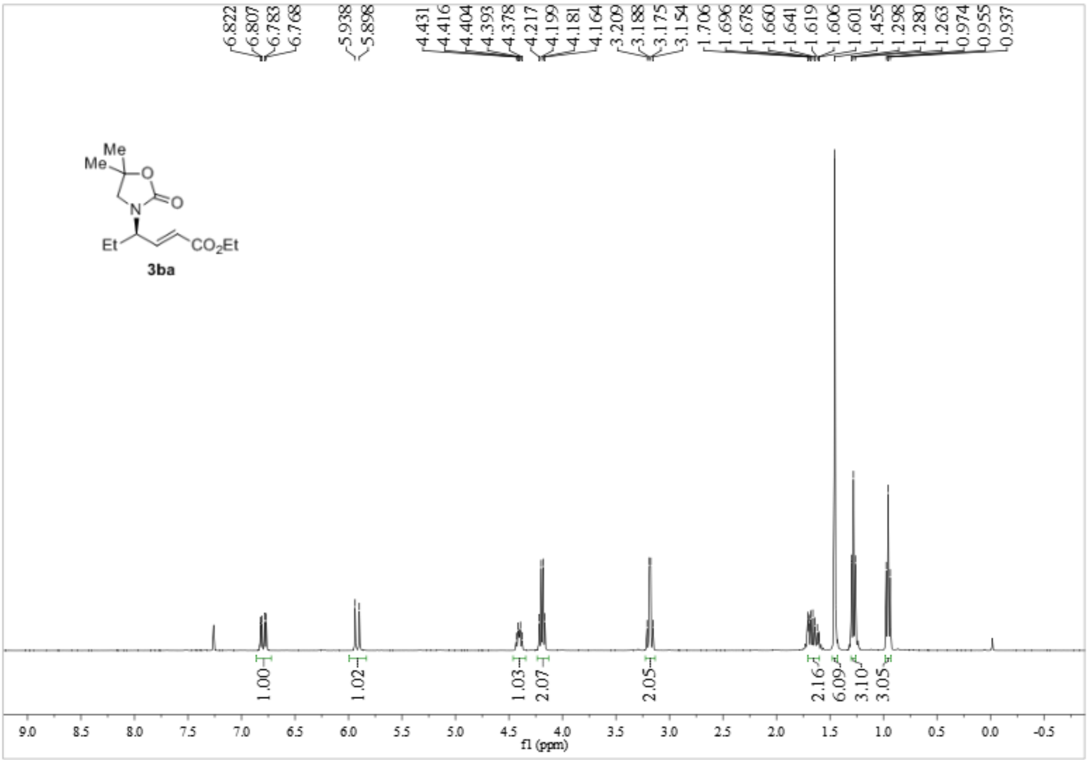

6.822
6.807
6.783
6.768
5.938
5.898
4.431
4.416
4.404
4.393
4.378
4.217
4.199
4.181
4.164
3.209
3.188
3.175
3.154
1.706
1.696
1.678
1.660
1.641
1.619
1.606
1.601
1.455
1.298
1.280
1.263
0.974
0.955
0.937
Me
Me
O
N
O
Et
CO2Et
3ba
1.00
1.02
1.03
2.07
2.05
2.16
6.09
3.10
3.05
9.0 8.5 8.0 7.5 7.0 6.5 6.0 5.5 5.0 4.5 4.0 3.5 3.0 2.5 2.0 1.5 1.0 0.5 0.0 -0.5
f1 (ppm)

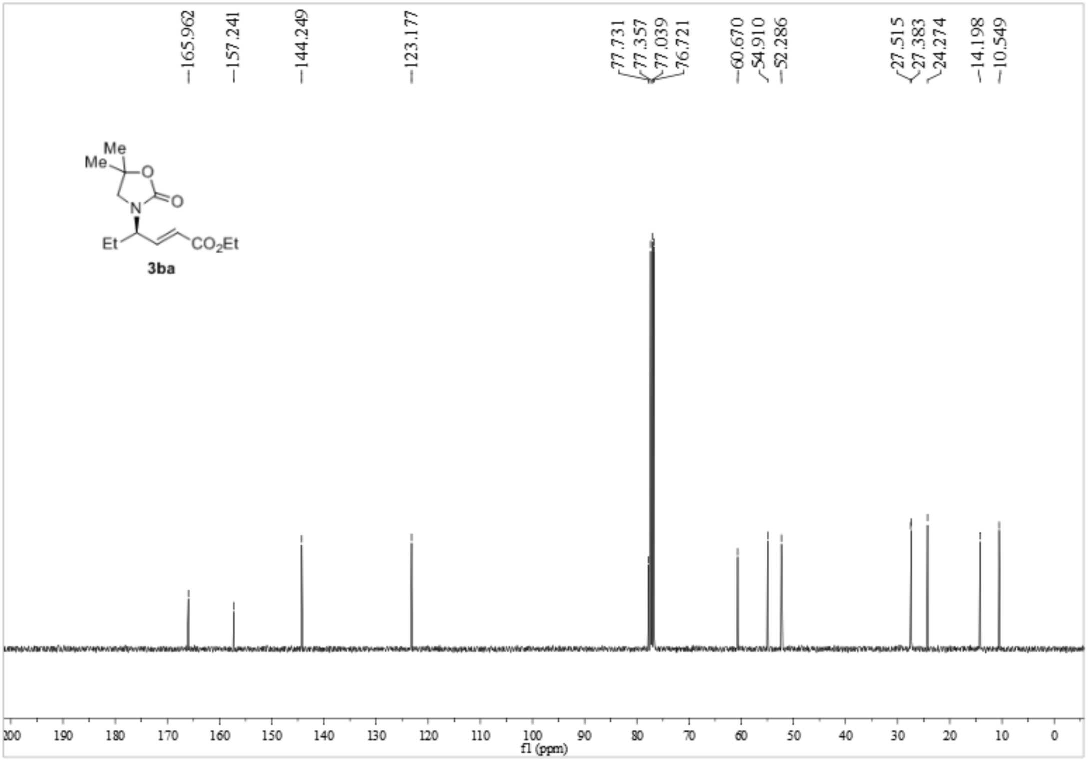

165.962
157.241
144.249
123.177
77.731
77.357
77.039
76.721
60.670
54.910
52.286
27.515
27.383
24.274
14.198
10.549
Me
Me
O
N
O
Et
CO2Et
3ba
200 190 180 170 160 150 140 130 120 110 100 90 80 70 60 50 40 30 20 10 0
f1 (ppm)

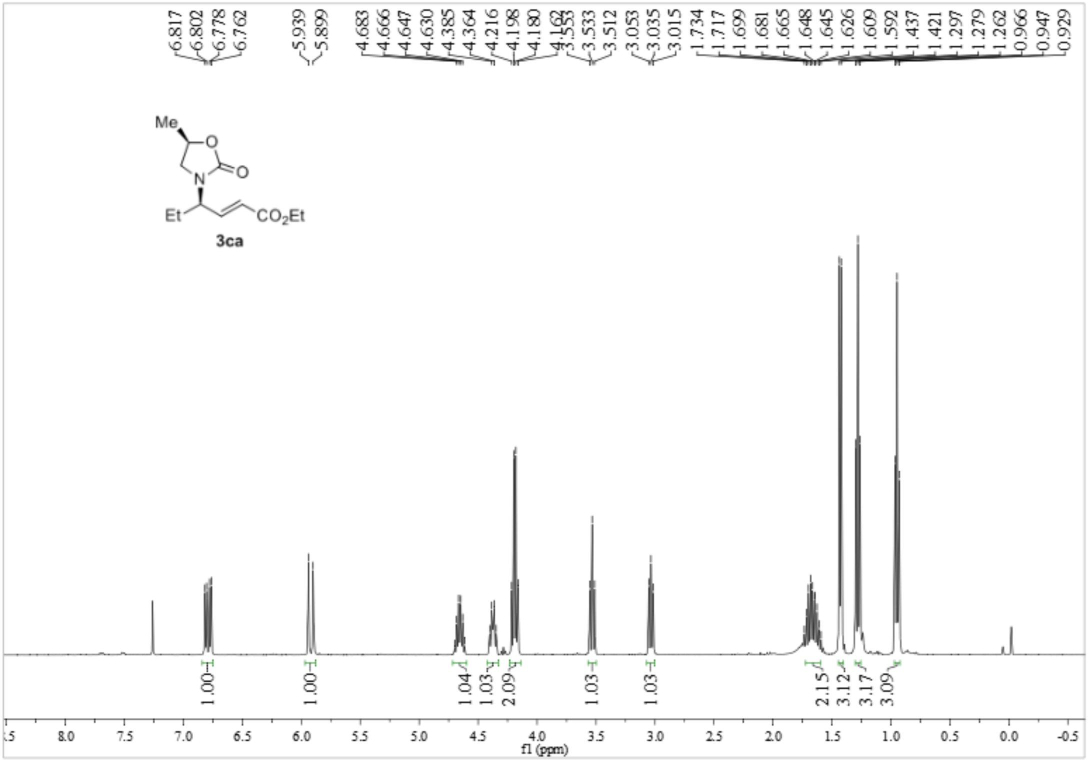
6.817
6.802
6.778
6.762
5.939
5.899
4.683
4.666
4.647
4.630
4.385
4.364
4.216
4.198
4.180
4.162
3.553
3.533
3.512
3.053
3.035
3.015
1.734
1.717
1.699
1.681
1.665
1.648
1.645
1.626
1.609
1.592
1.437
1.421
1.297
1.279
1.262
0.966
0.947
0.929
Me
O
N
O
Et
CO2Et
3ca
1.00
1.00
1.04
1.03
2.09
1.03
1.03
2.15
3.12
3.17
3.09
f1 (ppm)

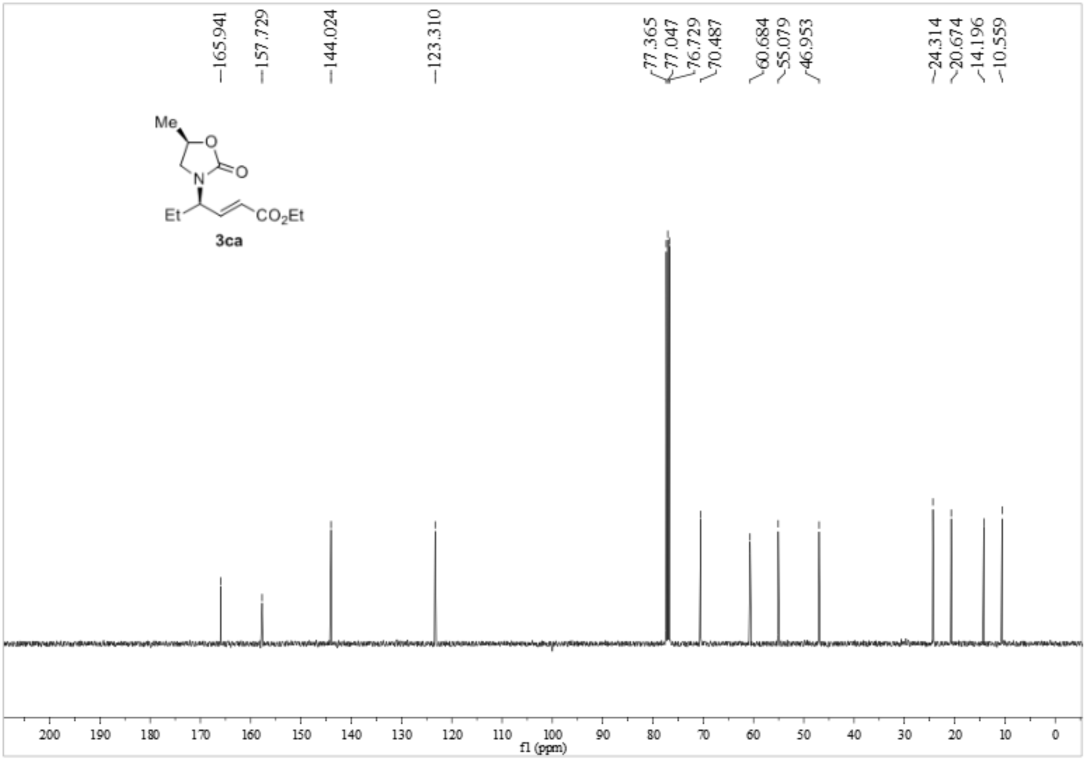
165.941
157.729
144.024
123.310
77.365
77.047
76.729
70.487
60.684
55.079
46.953
24.314
20.674
14.196
10.559
Me
O
N
O
Et
CO2Et
3ca
f1 (ppm)

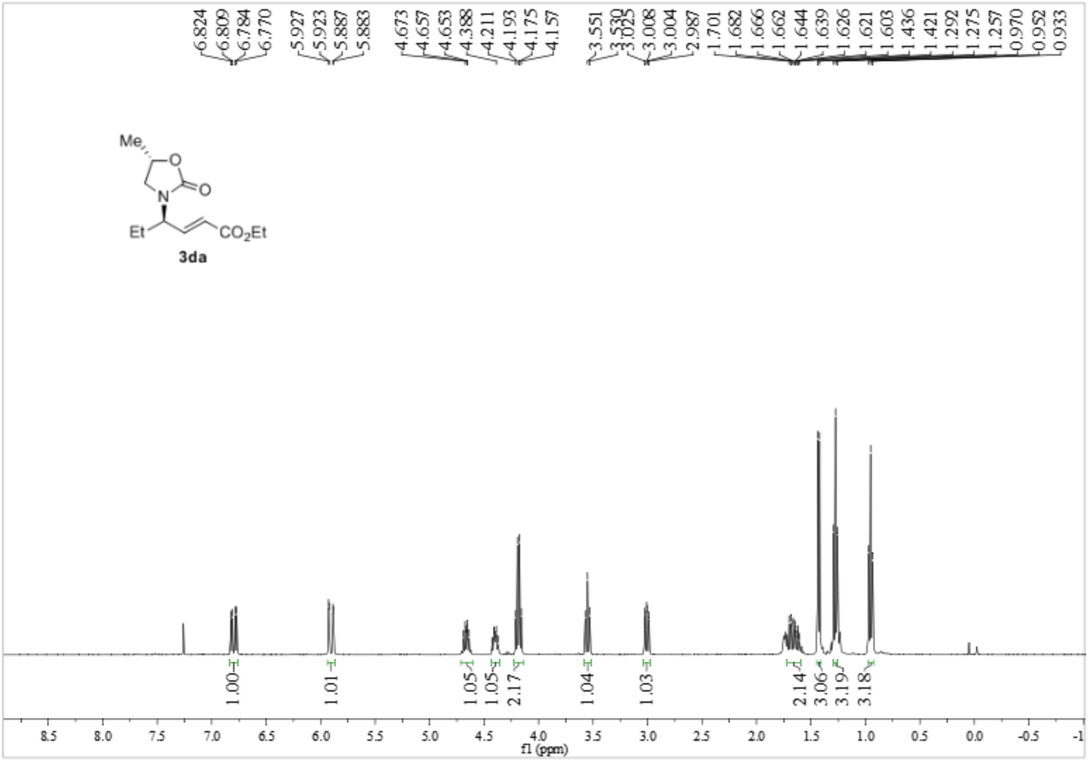
6.824
6.809
6.784
6.770
5.927
5.923
5.887
5.883
4.673
4.657
4.653
4.388
4.211
4.193
4.175
4.157
3.551
3.530
3.025
3.008
3.004
2.987
1.701
1.682
1.666
1.662
1.644
1.639
1.626
1.621
1.603
1.436
1.421
1.292
1.275
1.257
0.970
0.952
0.933
Me
O
N
O
Et
CO_2Et
3da
1.00
1.01
1.05
1.05
2.17
1.04
1.03
2.14
3.06
3.19
3.18
f1 (ppm)

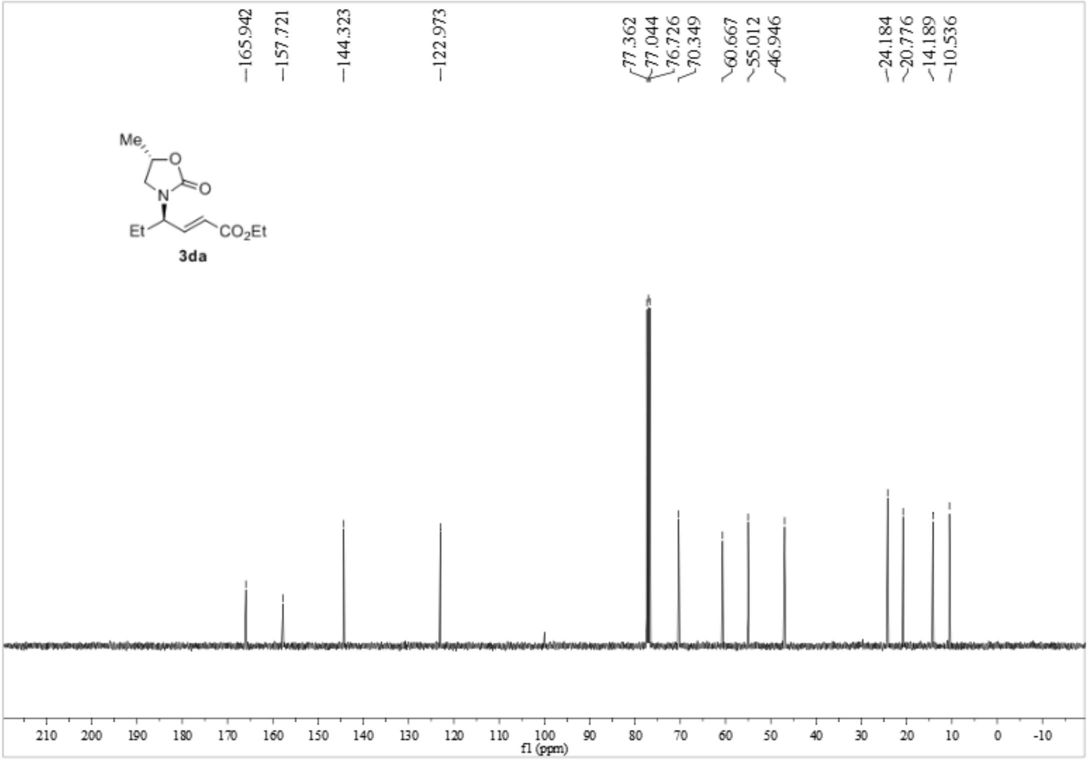
165.942
157.721
144.323
122.973
77.362
77.044
76.726
70.349
60.667
55.012
46.946
24.184
20.776
14.189
10.536
Me
O
N
O
Et
CO_2Et
3da
f1 (ppm)

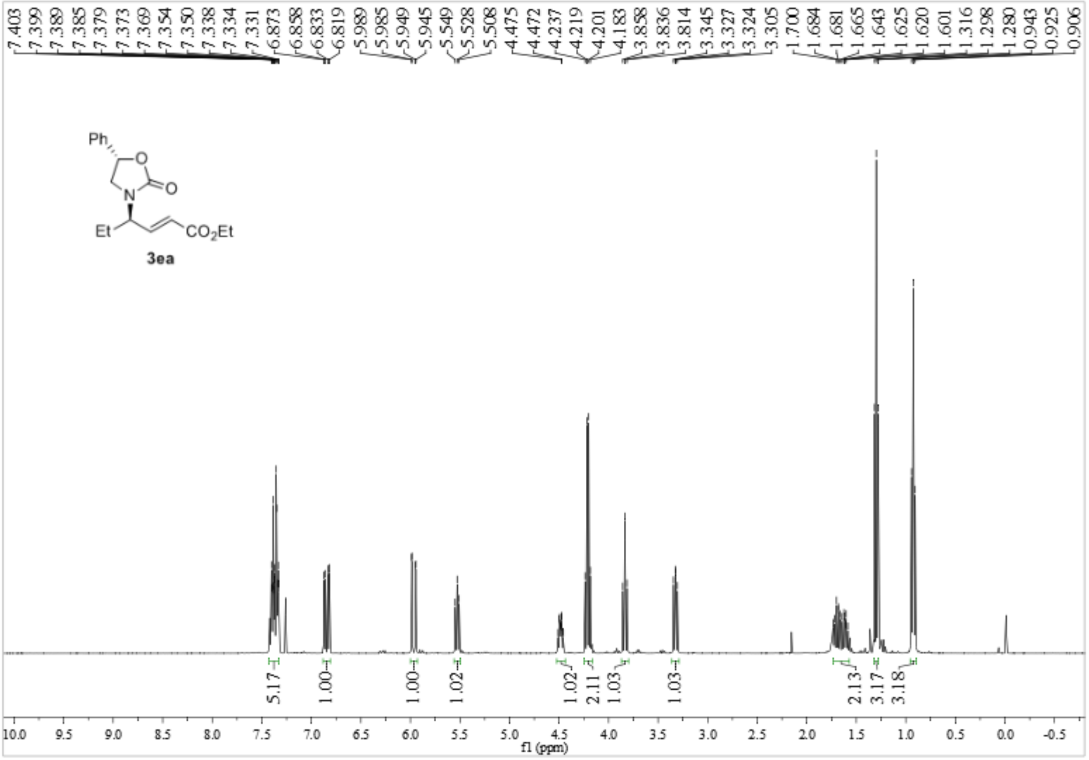

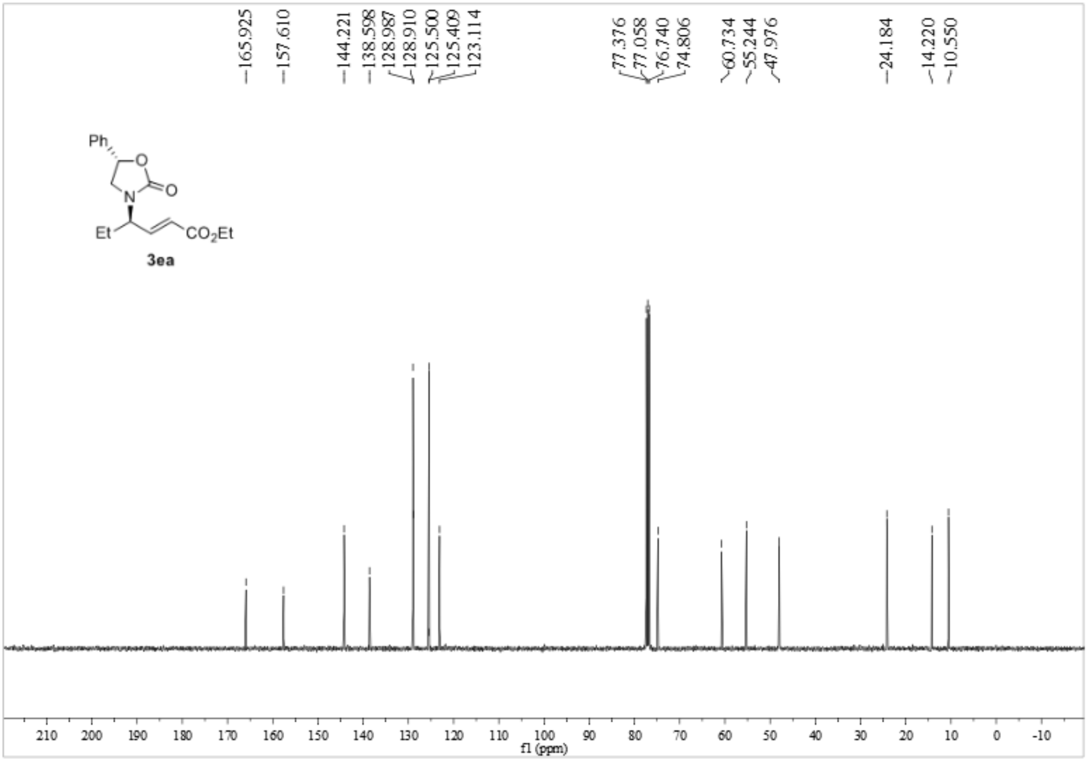

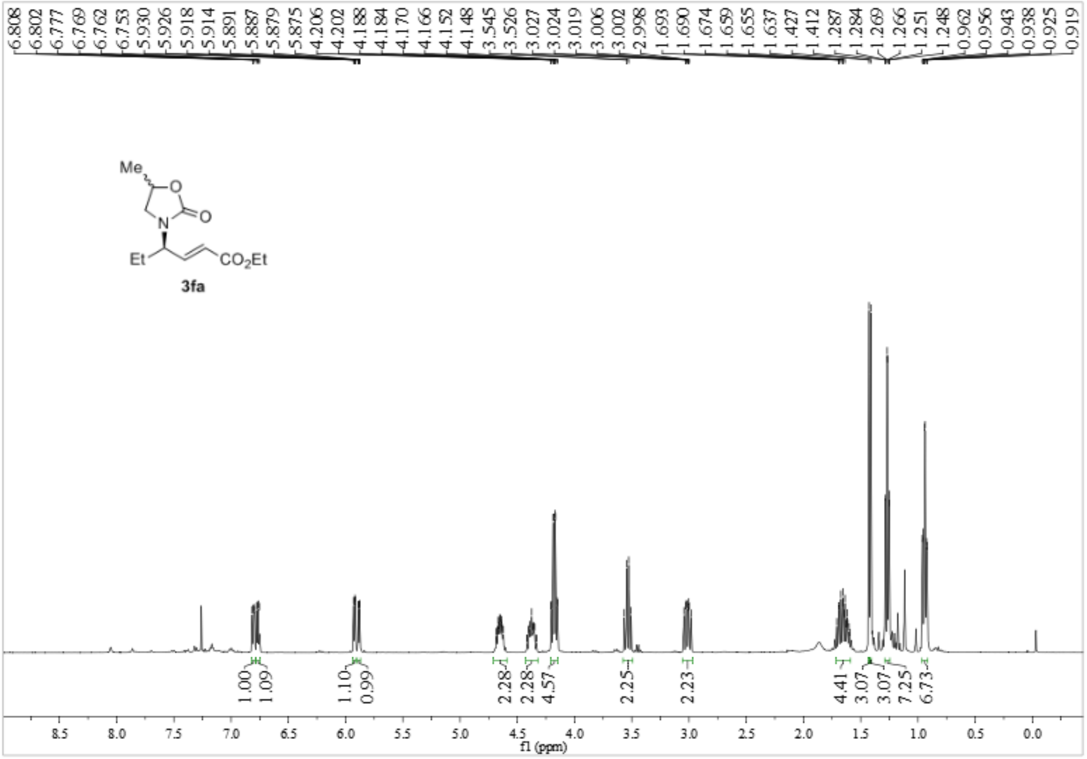
6.808
6.802
6.777
6.769
6.762
6.753
5.930
5.926
5.918
5.914
5.891
5.887
5.879
5.875
4.206
4.202
4.188
4.184
4.170
4.166
4.152
4.148
3.545
3.526
3.027
3.024
3.019
3.006
3.002
2.998
1.693
1.690
1.674
1.659
1.655
1.637
1.427
1.412
1.287
1.284
1.269
1.266
1.251
1.248
0.962
0.956
0.943
0.938
0.925
0.919
Me
O
N
O
Et
CO_2Et
3fa
1.00
1.09
1.10
0.99
2.28
2.28
4.57
2.25
2.23
4.41
3.07
3.07
7.25
6.73
f1 (ppm)

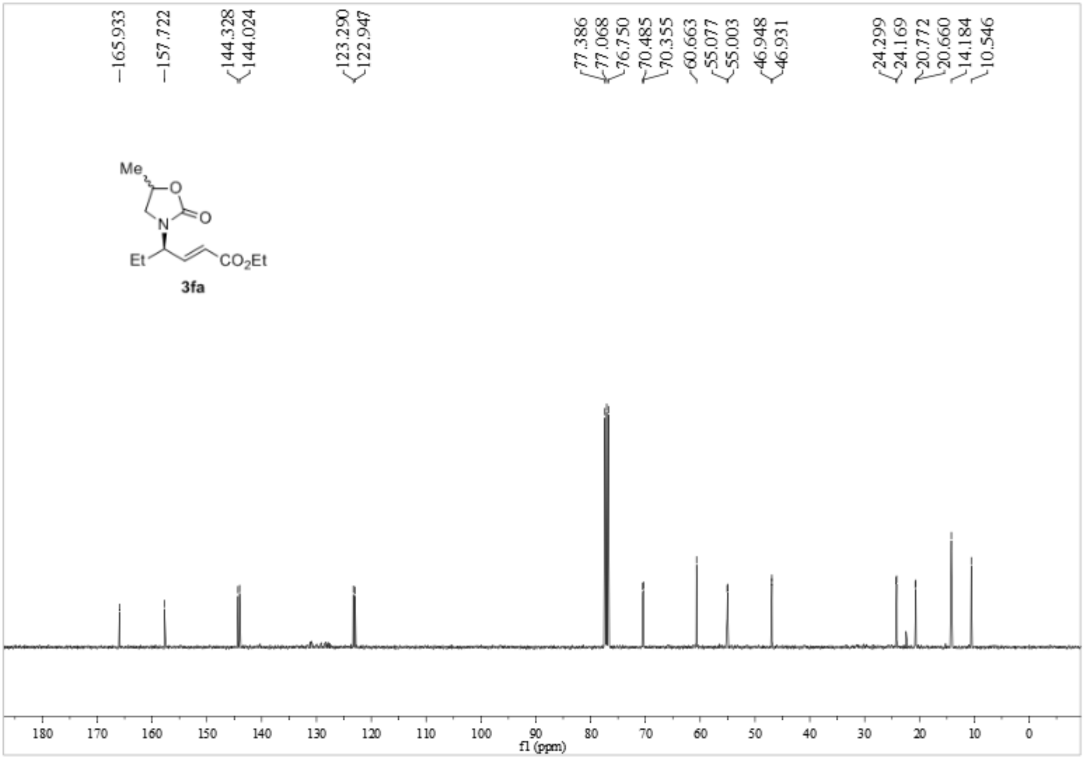
165.933
157.722
144.328
144.024
123.290
122.947
77.386
77.068
76.750
70.485
70.355
60.663
55.077
55.003
46.948
46.931
24.299
24.169
20.772
20.660
14.184
10.546
Me
O
N
O
Et
CO_2Et
3fa
f1 (ppm)

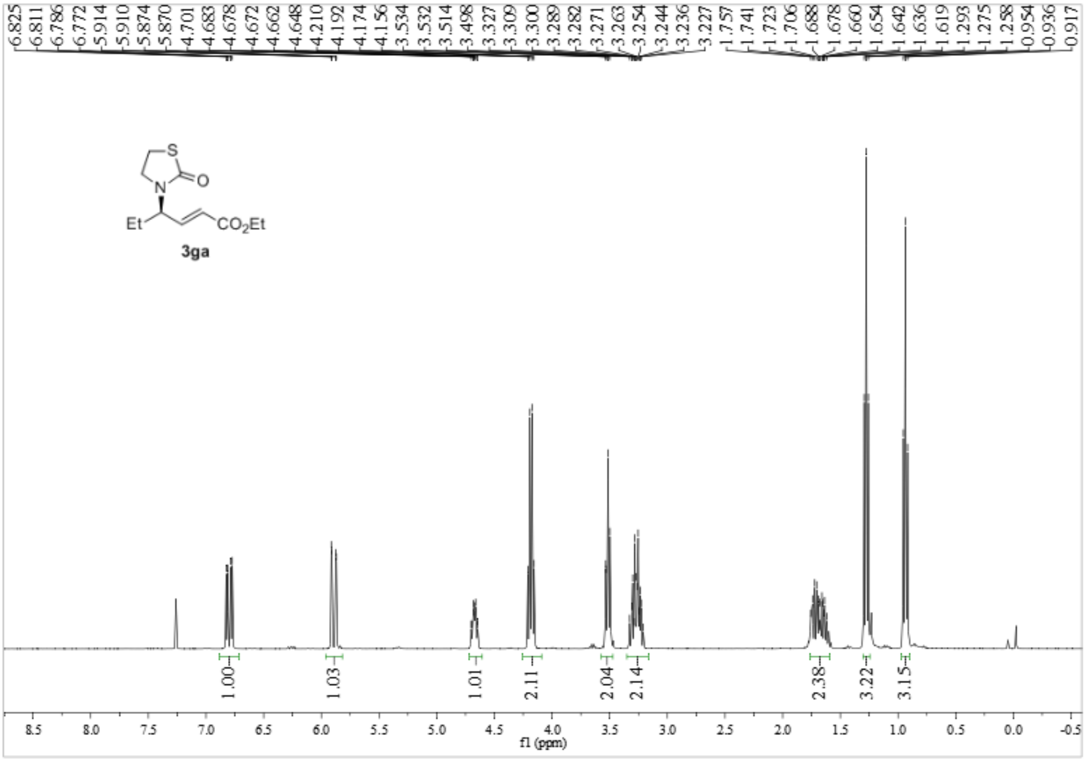

6.825
6.811
6.786
6.772
5.914
5.910
5.874
5.870
4.701
4.683
4.678
4.672
4.662
4.648
4.210
4.192
4.174
4.156
3.534
3.532
3.514
3.498
3.327
3.309
3.300
3.289
3.282
3.271
3.263
3.254
3.244
3.236
3.227
1.757
1.741
1.723
1.706
1.688
1.678
1.660
1.654
1.642
1.636
1.619
1.293
1.275
1.258
0.954
0.936
0.917
Et
CO2Et
3ga
1.00
1.03
1.01
2.11
2.04
2.14
2.38
3.22
3.15
f1 (ppm)

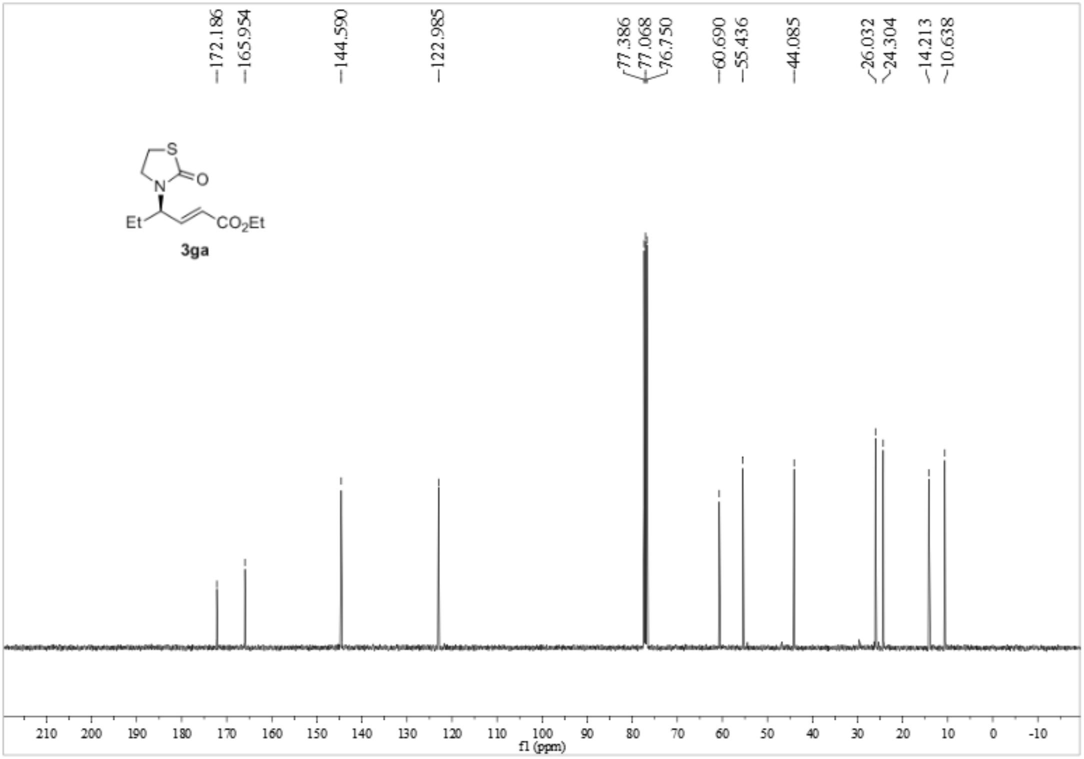

172.186
165.954
144.590
122.985
77.386
77.068
76.750
60.690
55.436
44.085
26.032
24.304
14.213
10.638
Et
CO2Et
3ga
f1 (ppm)

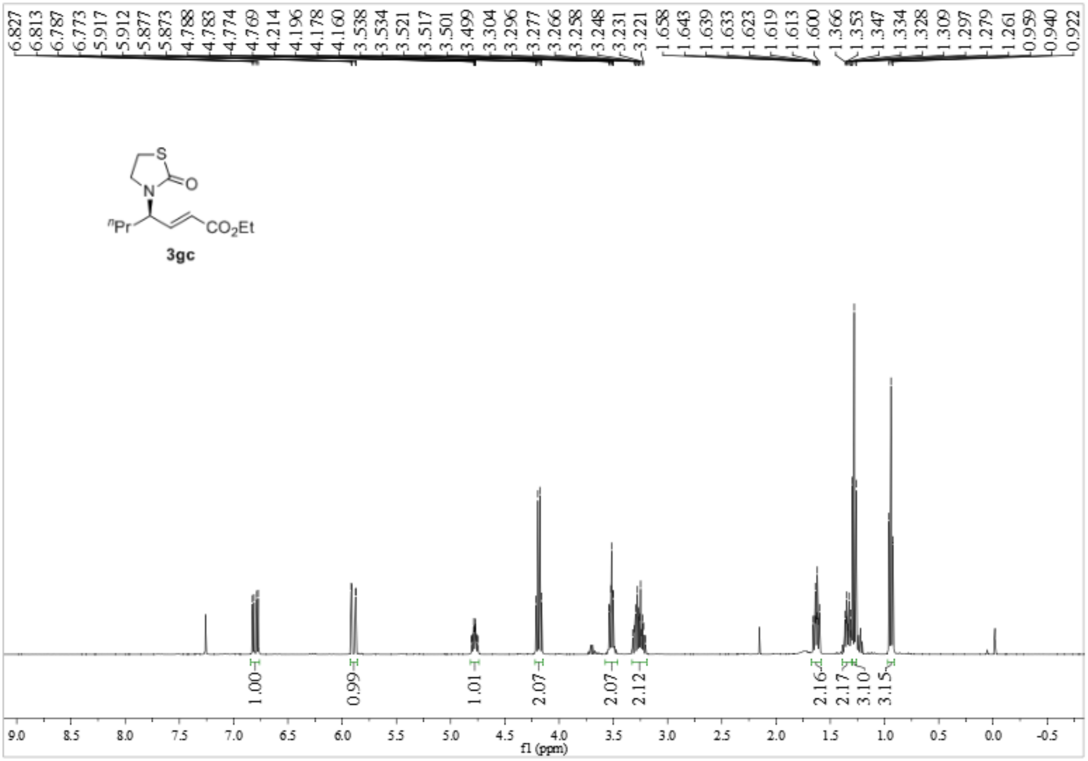

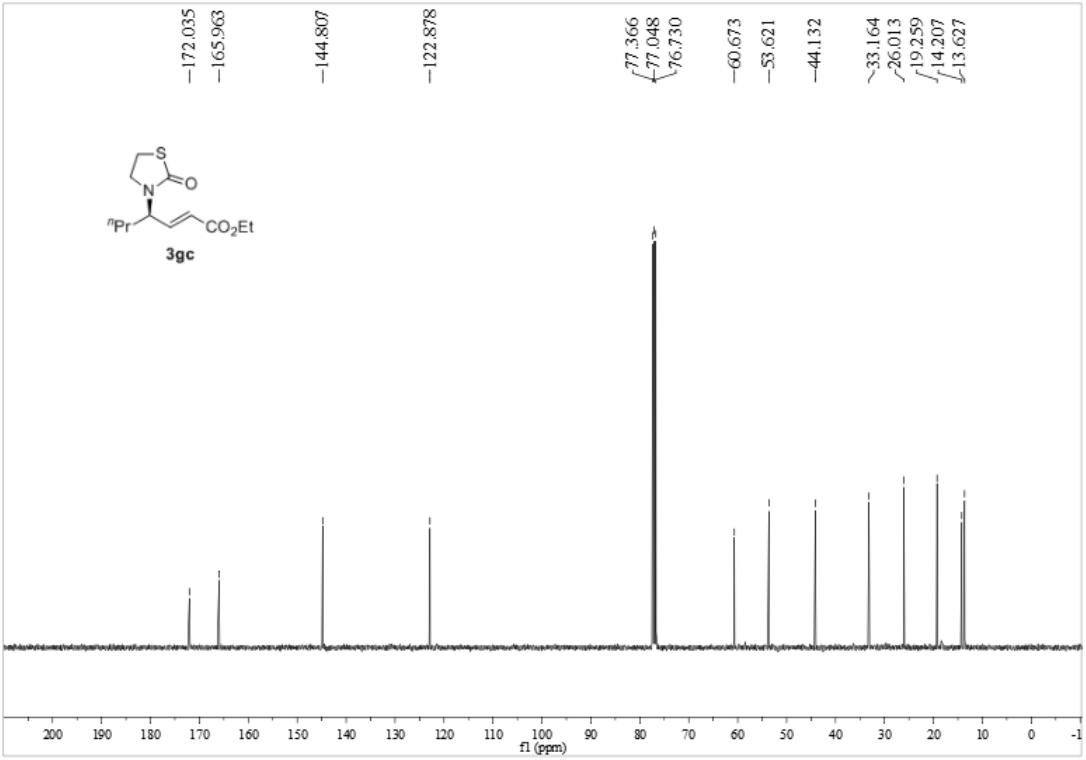

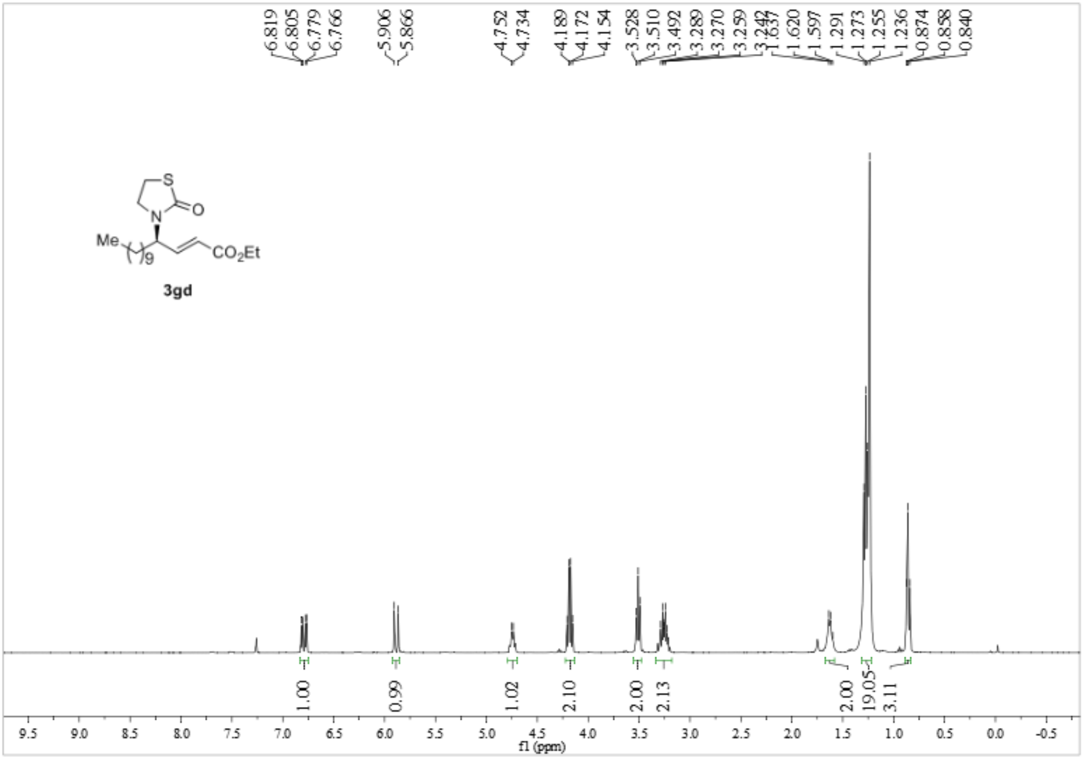
6.819
6.805
6.779
6.766
5.906
5.866
4.752
4.734
4.189
4.172
4.154
3.528
3.510
3.492
3.289
3.270
3.259
3.242
1.637
1.620
1.597
1.291
1.273
1.255
1.236
0.874
0.858
0.840
S
N
O
Me
9
CO2Et
3gd
1.00
0.99
1.02
2.10
2.00
2.13
2.00
19.05
3.11
f1 (ppm)

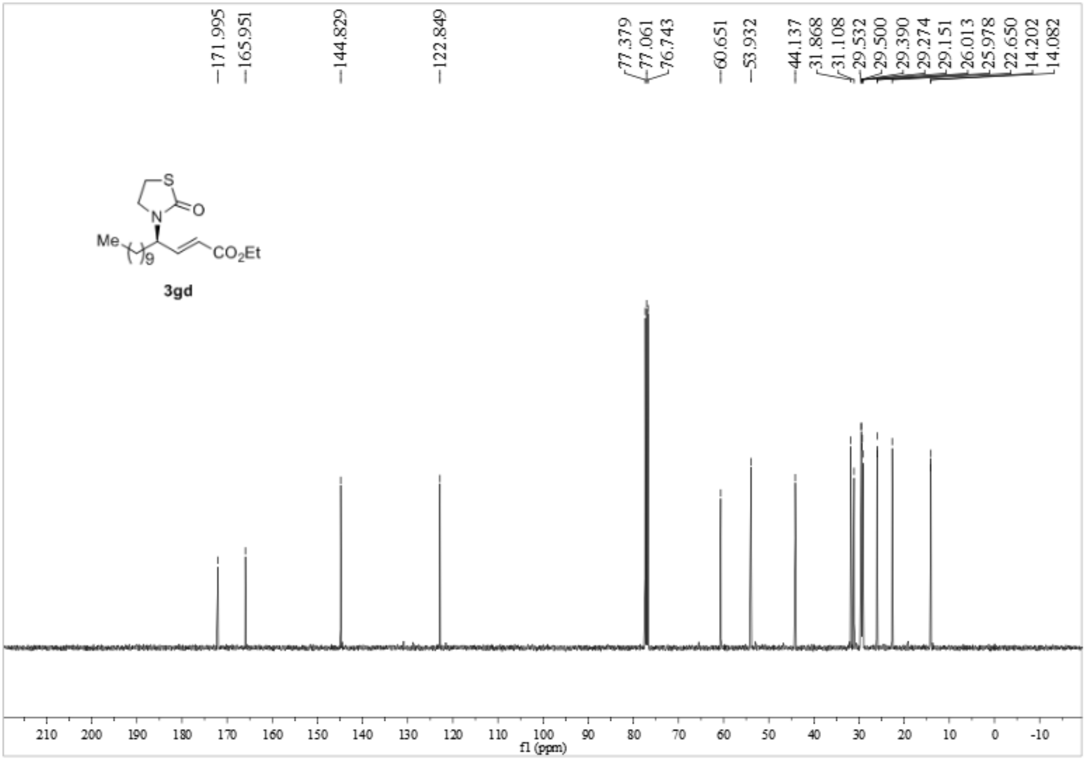
171.995
165.951
144.829
122.849
77.379
77.061
76.743
60.651
53.932
44.137
31.868
31.108
29.532
29.500
29.390
29.274
29.151
26.013
25.978
22.650
14.202
14.082
S
N
O
Me
9
CO2Et
3gd
f1 (ppm)

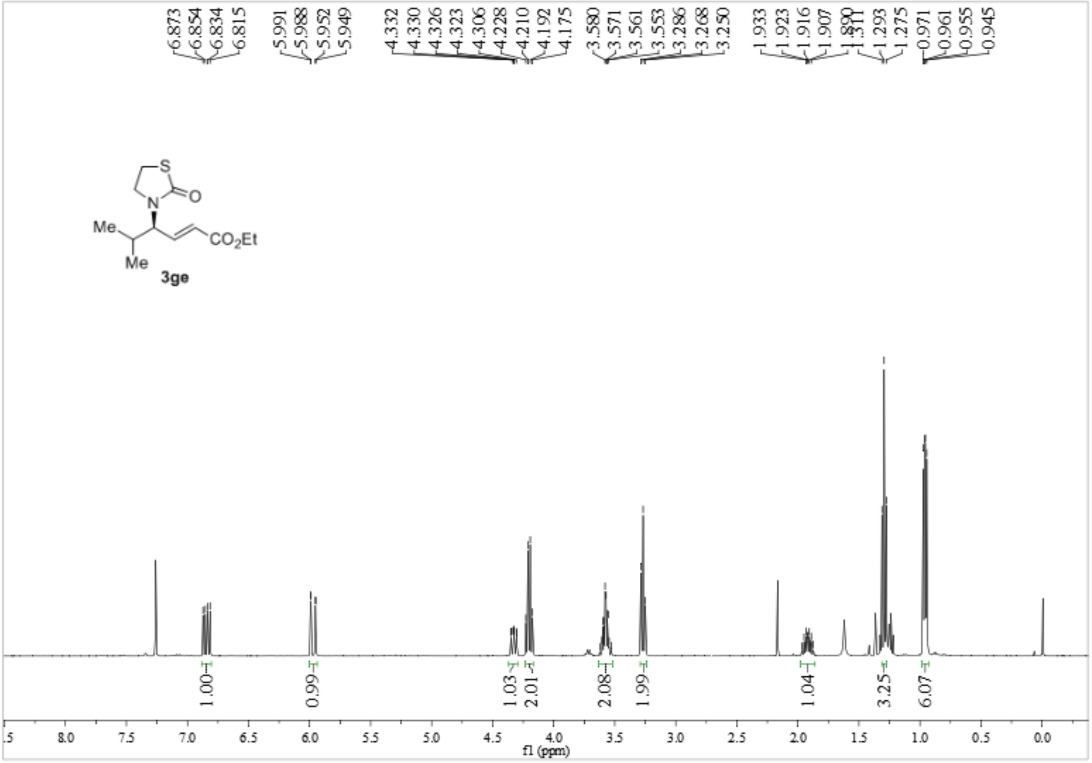
3ge
f1 (ppm)

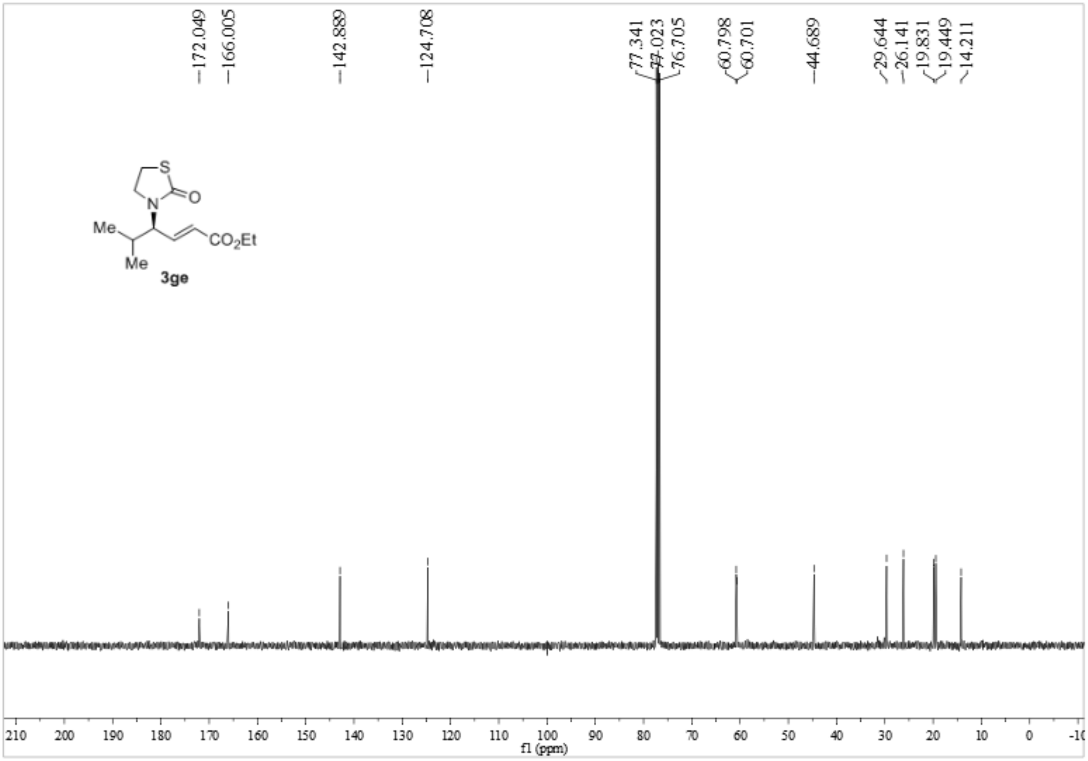
3ge
f1 (ppm)

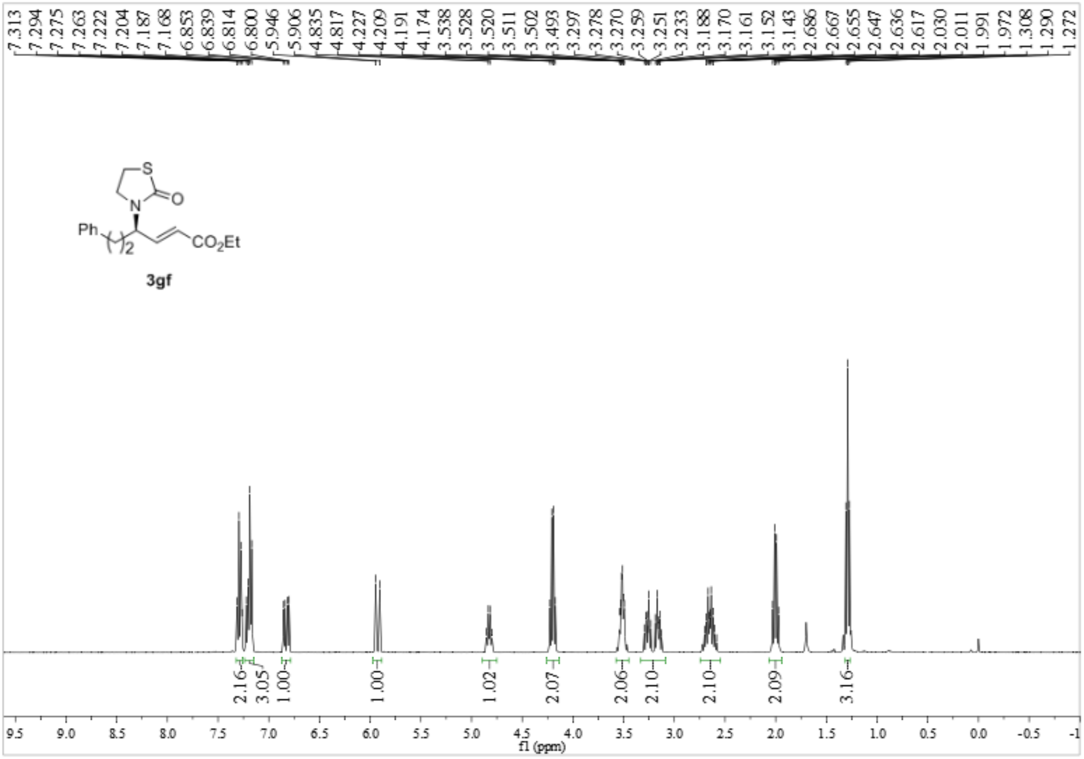
7.313 7.294 7.275 7.263 7.222 7.204 7.187 7.168 6.853 6.839 6.814 6.800 5.946 5.906 4.835 4.817 4.227 4.209 4.191 4.174 3.538 3.528 3.520 3.511 3.502 3.493 3.297 3.278 3.270 3.259 3.251 3.233 3.188 3.170 3.161 3.152 3.143 2.686 2.667 2.655 2.647 2.636 2.617 2.030 2.011 1.991 1.972 1.308 1.290 1.272
S
N
O
Ph
2
CO_2Et
3gf
2.16 3.05 1.00 1.00 1.02 2.07 2.06 2.10 2.10 2.09 3.16
f1 (ppm)

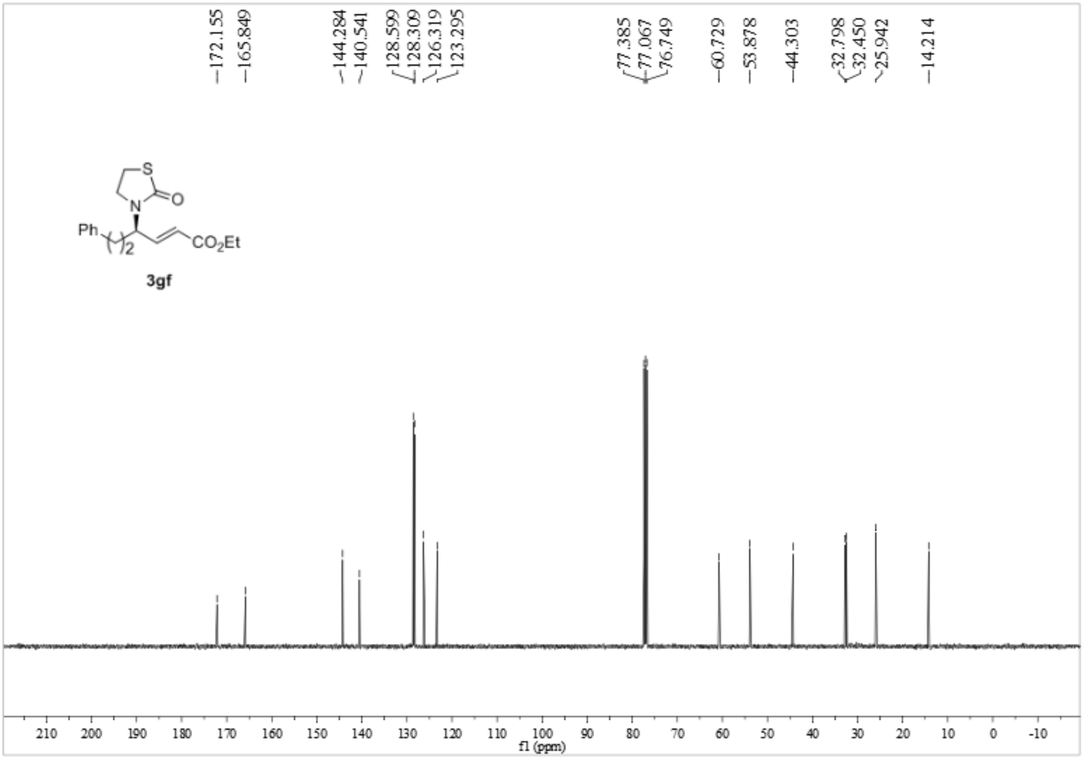
172.155 165.849 144.284 140.541 128.599 128.309 126.319 123.295 77.385 77.067 76.749 60.729 53.878 44.303 32.798 32.450 25.942 14.214
S
N
O
Ph
2
CO_2Et
3gf
f1 (ppm)

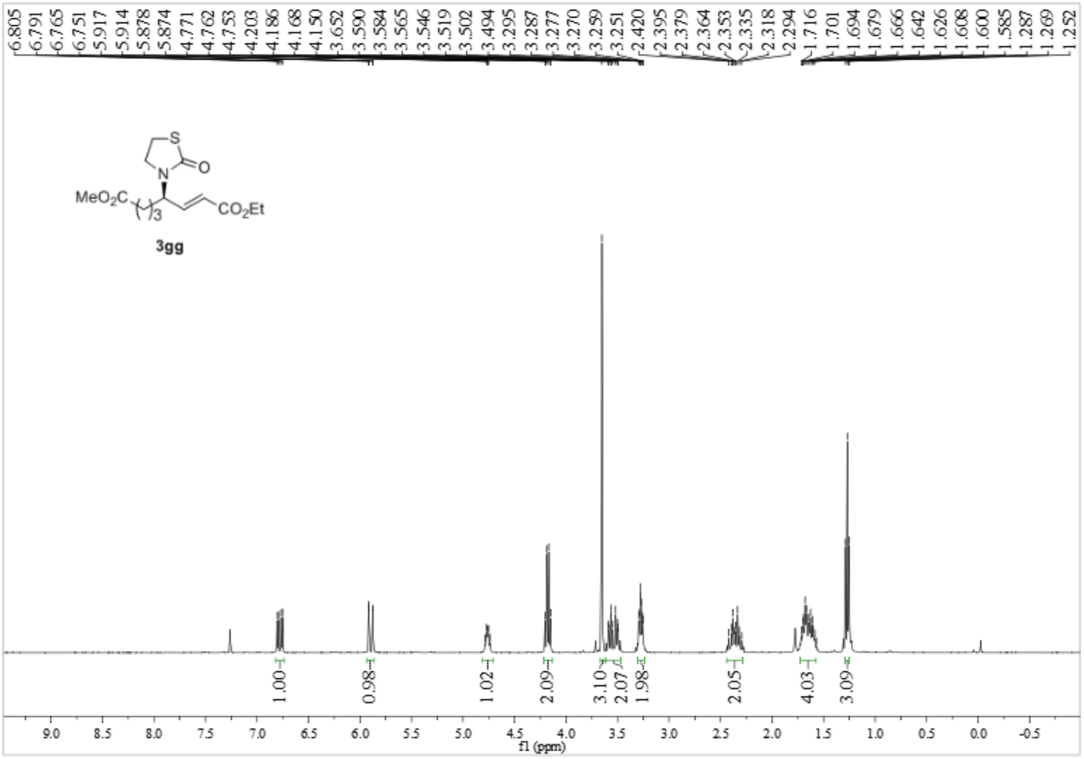
6.805
6.791
6.765
6.751
5.917
5.914
5.878
5.874
4.771
4.762
4.753
4.203
4.186
4.168
4.150
3.652
3.590
3.584
3.565
3.546
3.519
3.502
3.494
3.295
3.287
3.277
3.270
3.259
3.251
2.420
2.395
2.379
2.364
2.353
2.335
2.318
2.294
1.716
1.701
1.694
1.679
1.666
1.642
1.626
1.608
1.600
1.585
1.287
1.269
1.252
MeO2C
N
S
O
3
CO2Et
3gg
1.00
0.98
1.02
2.09
3.10
2.07
1.98
2.05
4.03
3.09
f1 (ppm)

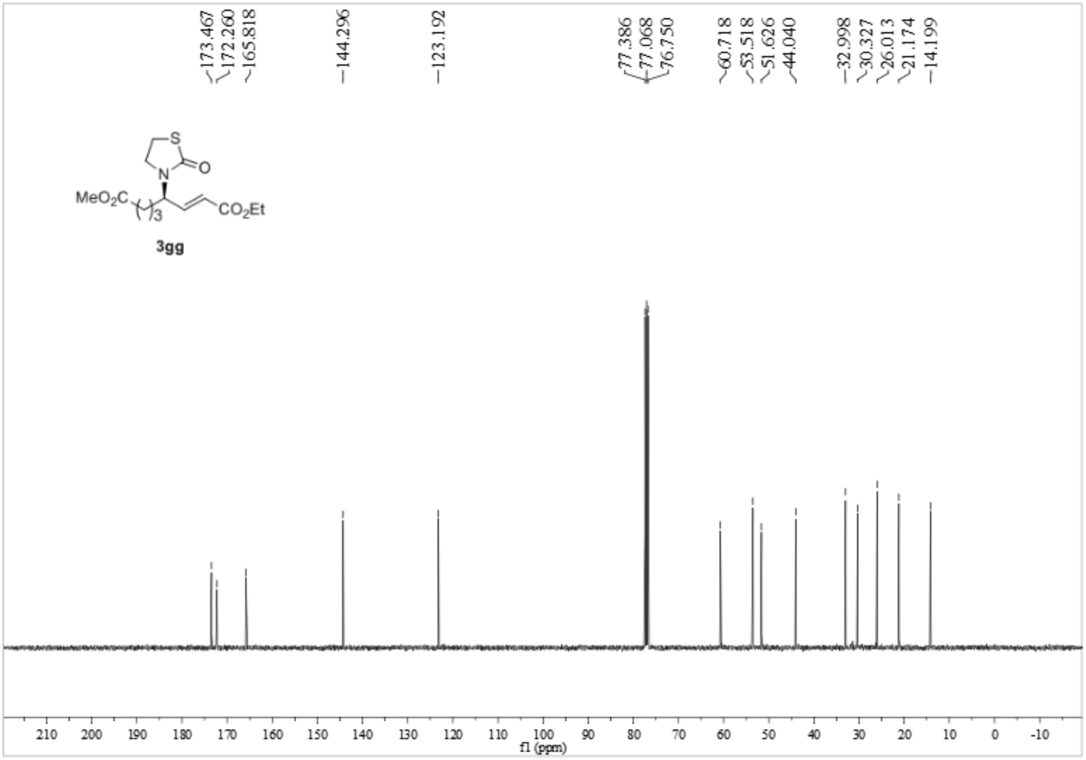
173.467
172.260
165.818
144.296
123.192
77.386
77.068
76.750
60.718
53.518
51.626
44.040
32.998
30.327
26.013
21.174
14.199
MeO2C
N
S
O
3
CO2Et
3gg
f1 (ppm)

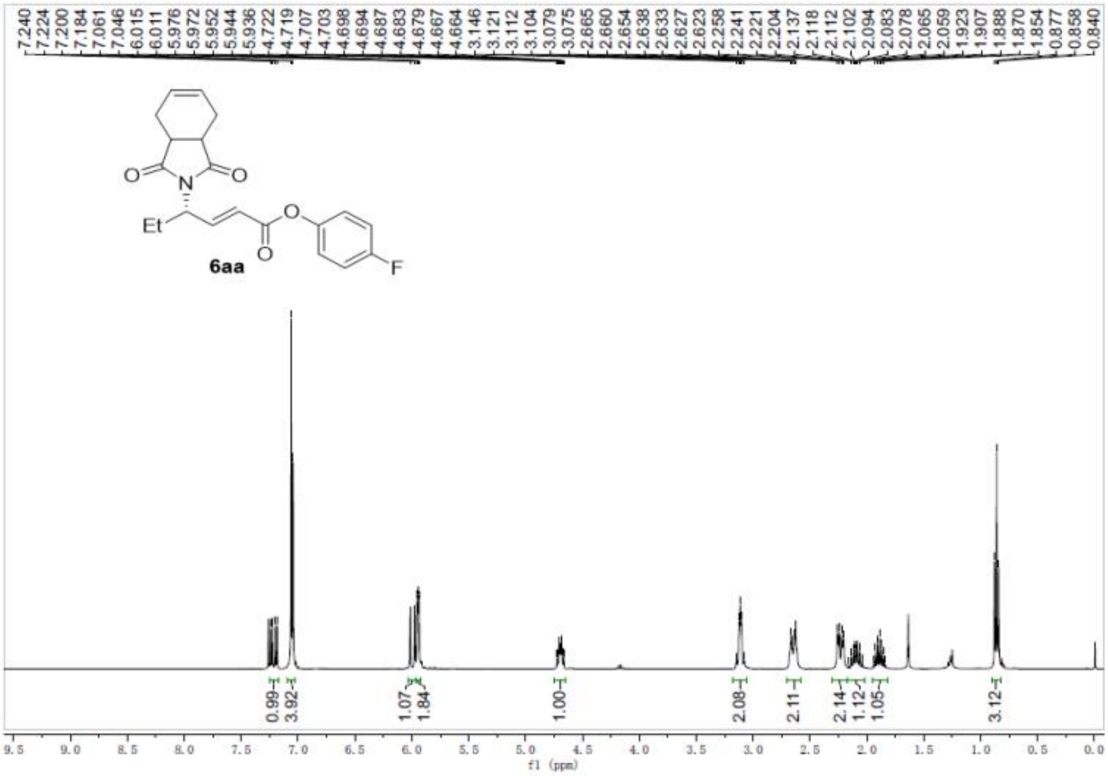

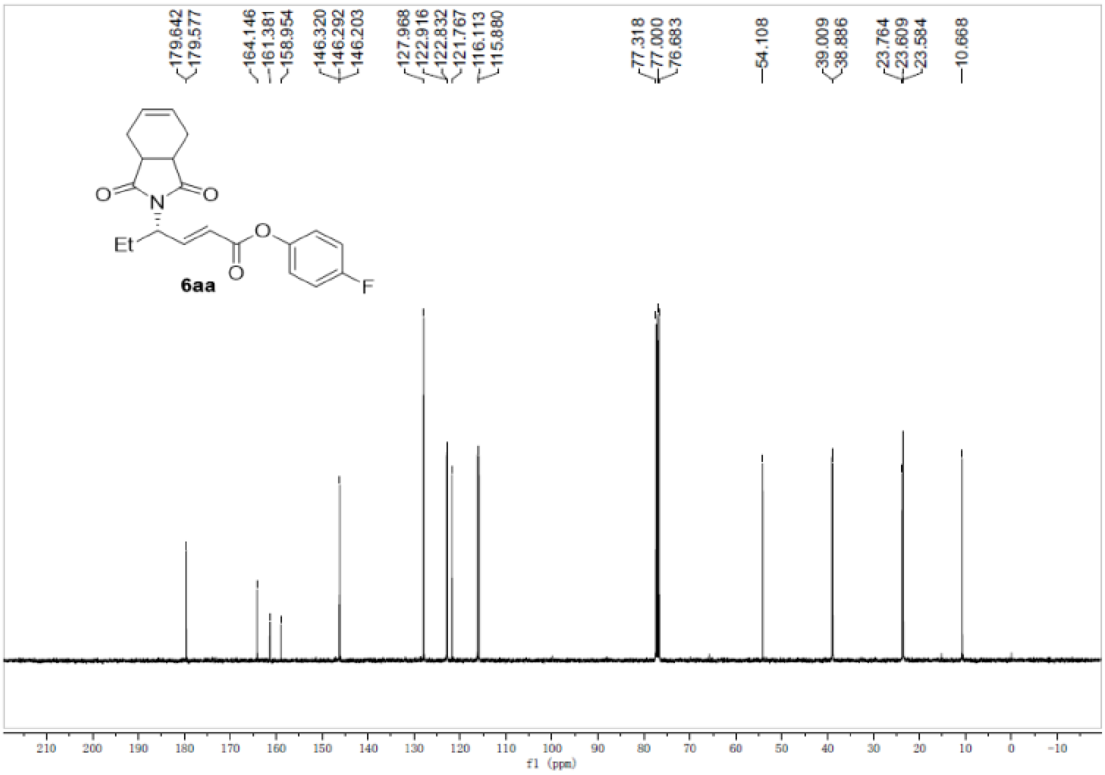

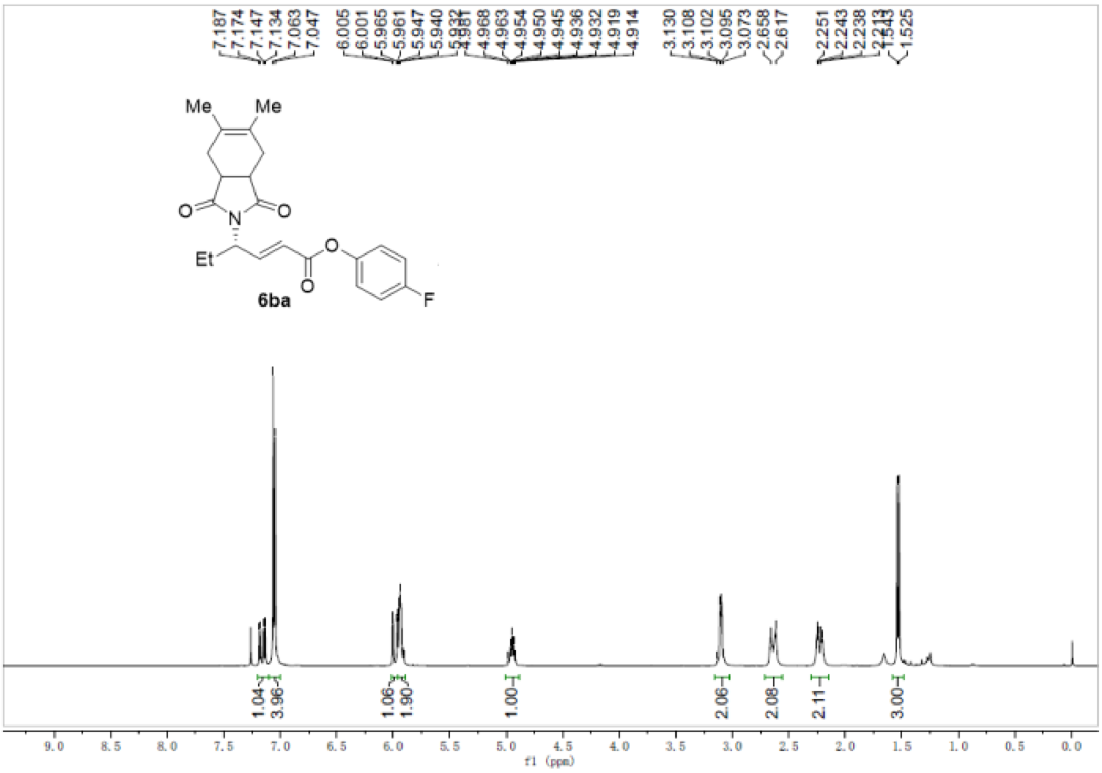
7.187
7.174
7.147
7.134
7.063
7.047
6.005
6.001
5.965
5.961
5.947
5.940
4.968
4.963
4.954
4.950
4.945
4.936
4.932
4.919
4.914
3.130
3.108
3.102
3.095
3.073
2.658
2.617
2.251
2.243
2.238
1.525
Me
Me
O
N
O
Et
O
O
F
6ba
1.04
3.96
1.06
1.90
1.00
2.06
2.08
2.11
3.00
f1 (ppm)

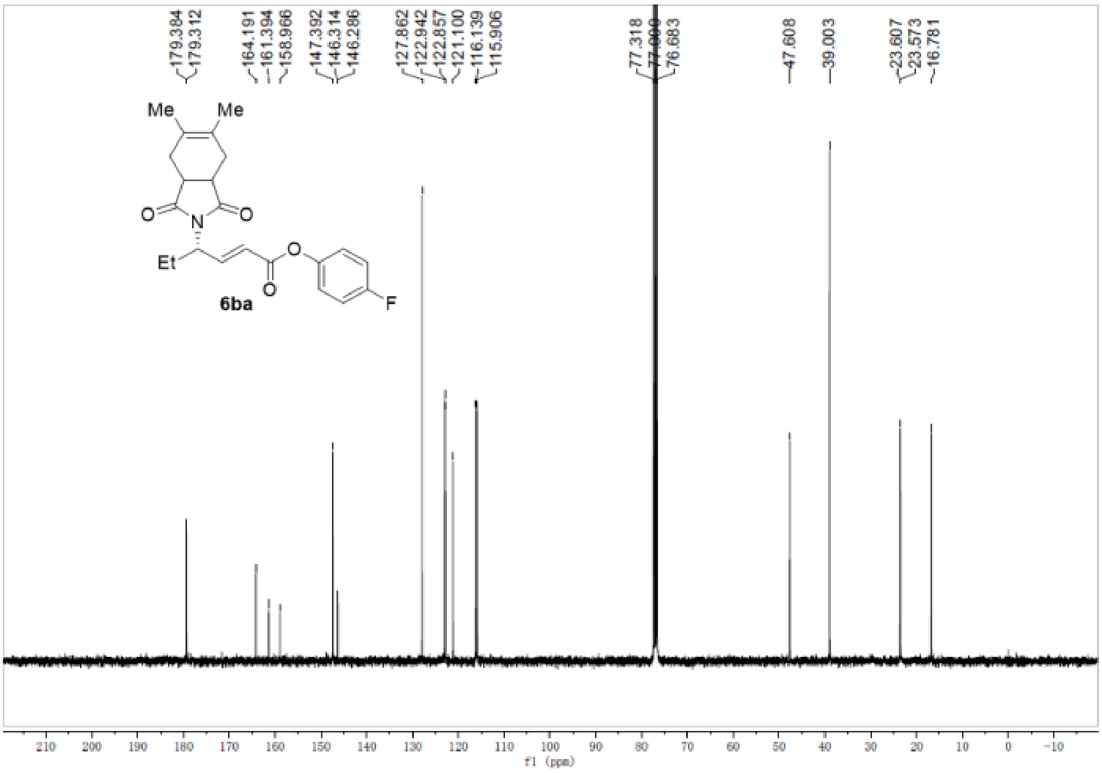
179.384
179.312
164.191
161.394
158.966
147.392
146.314
146.286
127.862
122.942
122.857
121.100
116.139
115.906
77.318
76.683
47.608
39.003
23.607
23.573
16.781
Me
Me
O
N
O
Et
O
O
F
6ba
f1 (ppm)

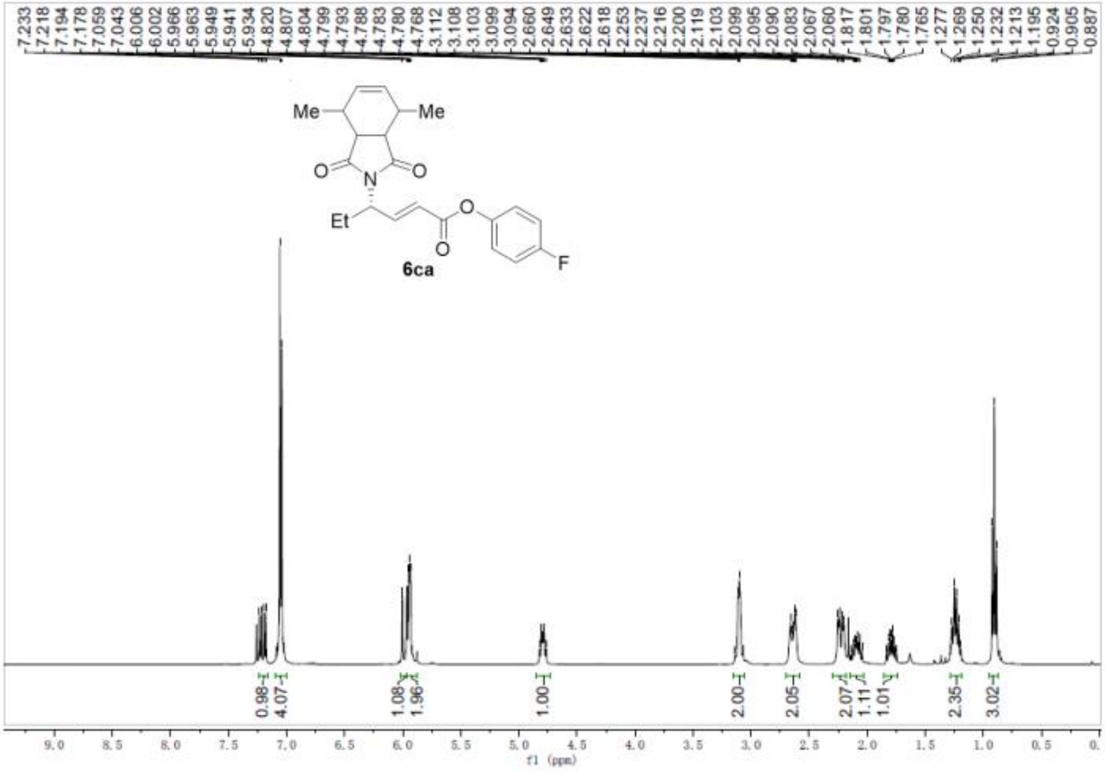
7.233
7.218
7.194
7.178
7.059
7.043
6.006
6.002
5.966
5.963
5.949
5.941
5.934
4.820
4.807
4.804
4.799
4.793
4.788
4.783
4.780
4.768
3.112
3.108
3.103
3.099
3.094
2.660
2.649
2.633
2.622
2.618
2.253
2.237
2.216
2.200
2.119
2.103
2.099
2.095
2.090
2.083
2.067
2.060
1.817
1.801
1.797
1.780
1.765
1.277
1.269
1.250
1.232
1.213
1.195
0.924
0.905
0.887
Me
Me
O
N
O
Et
O
O
F
6ca
0.98
4.07
1.08
1.96
1.00
2.00
2.05
2.07
1.11
1.01
2.35
3.02
f1 (ppm)

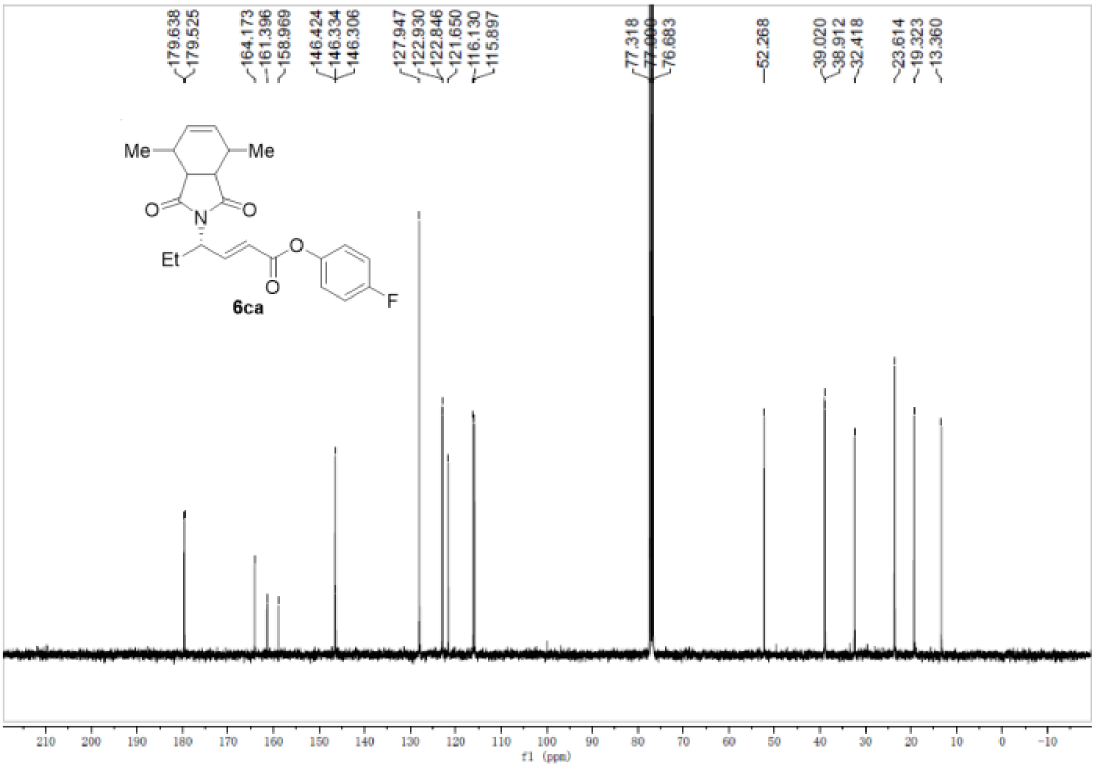
179.638
179.525
164.173
161.396
158.969
146.424
146.334
146.306
127.947
122.930
122.846
121.650
116.130
115.897
77.318
77.000
76.683
52.268
39.020
38.912
32.418
23.614
19.323
13.360
Me
Me
O
N
O
Et
O
O
F
6ca
f1 (ppm)

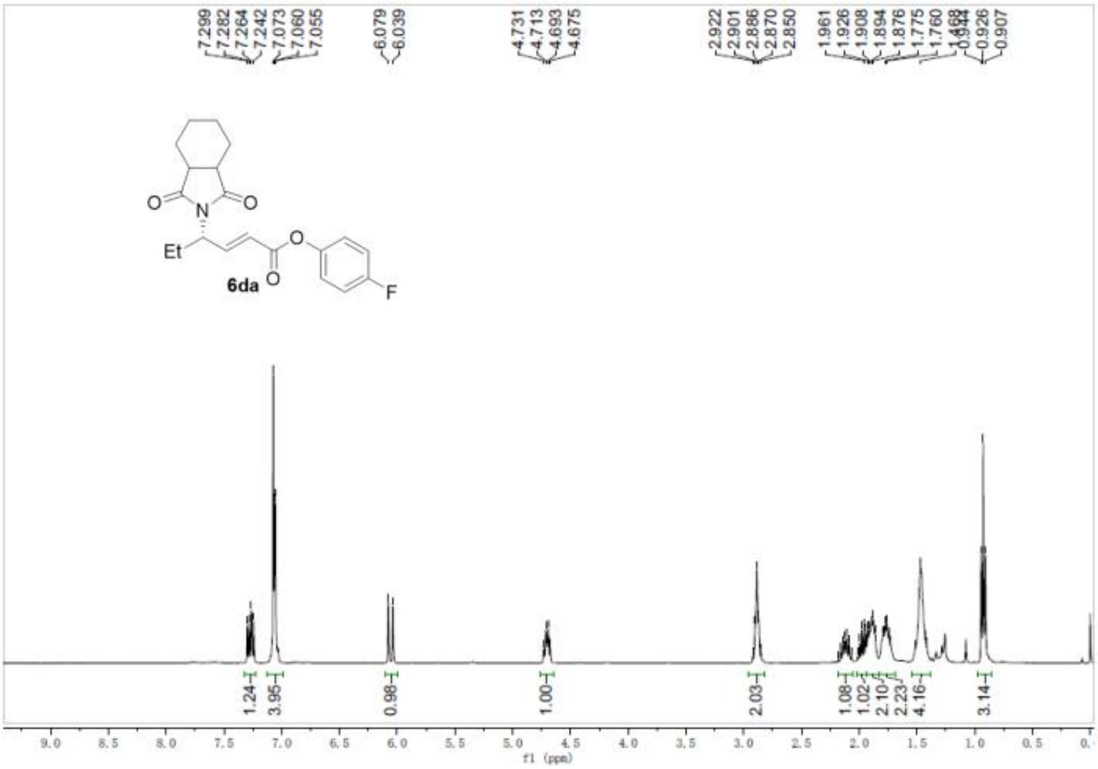
7.299
7.282
7.264
7.242
7.073
7.060
7.055
6.079
6.039
4.731
4.713
4.693
4.675
2.922
2.901
2.886
2.870
2.850
1.961
1.926
1.908
1.894
1.876
1.775
1.760
0.926
0.907
O
N
O
Et
O
O
F
6da
1.24
3.95
0.98
1.00
2.03
1.08
1.02
2.10
2.23
4.16
3.14
f1 (ppm)

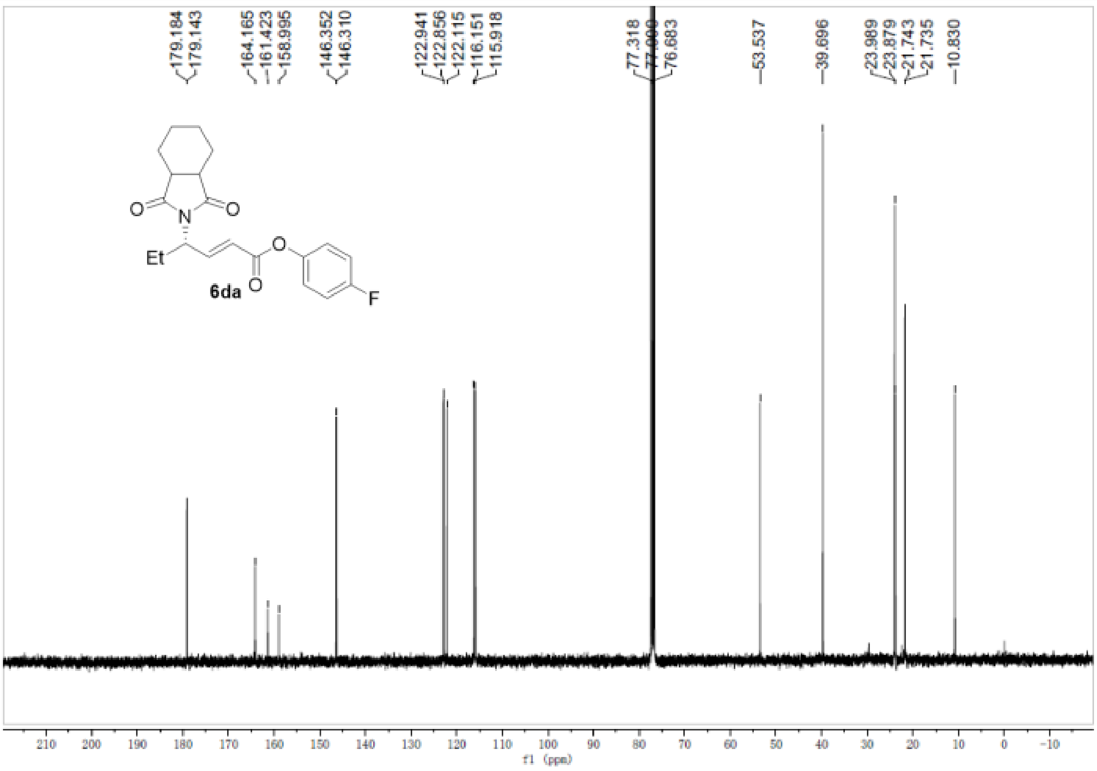
179.184
179.143
164.165
161.423
158.995
146.352
146.310
122.941
122.856
122.115
116.151
115.918
77.318
76.683
53.537
39.696
23.989
23.879
21.743
21.735
10.830
O
N
O
Et
O
O
F
6da
f1 (ppm)

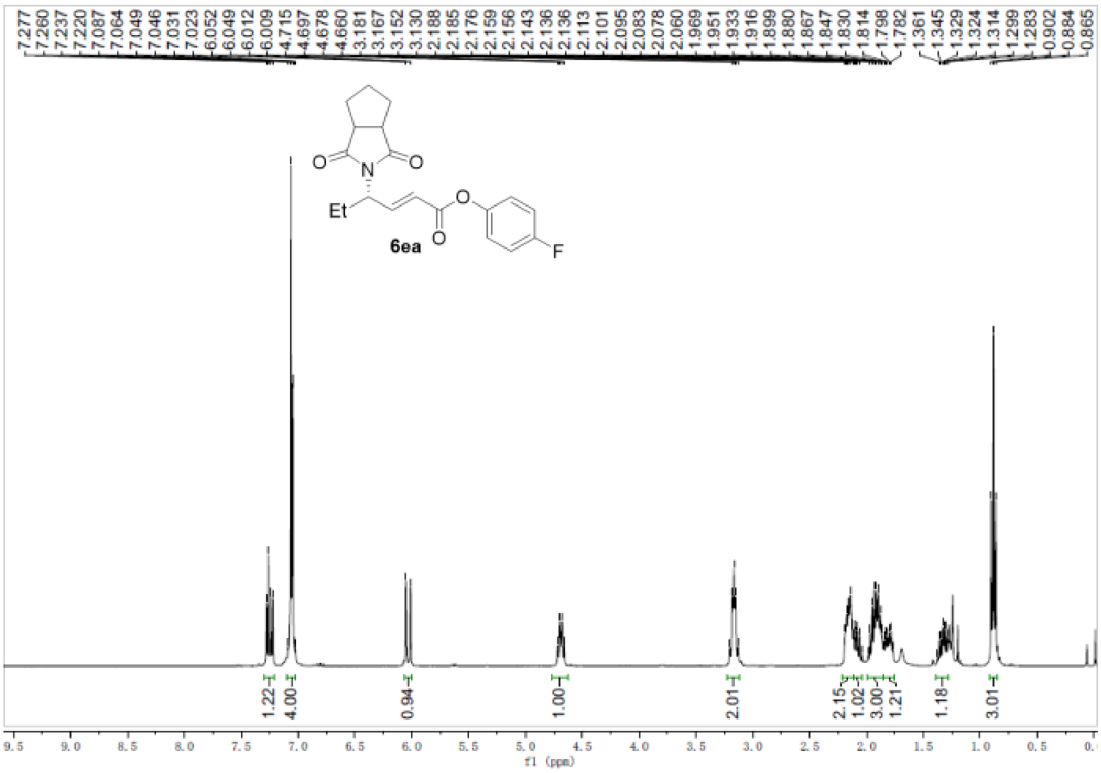

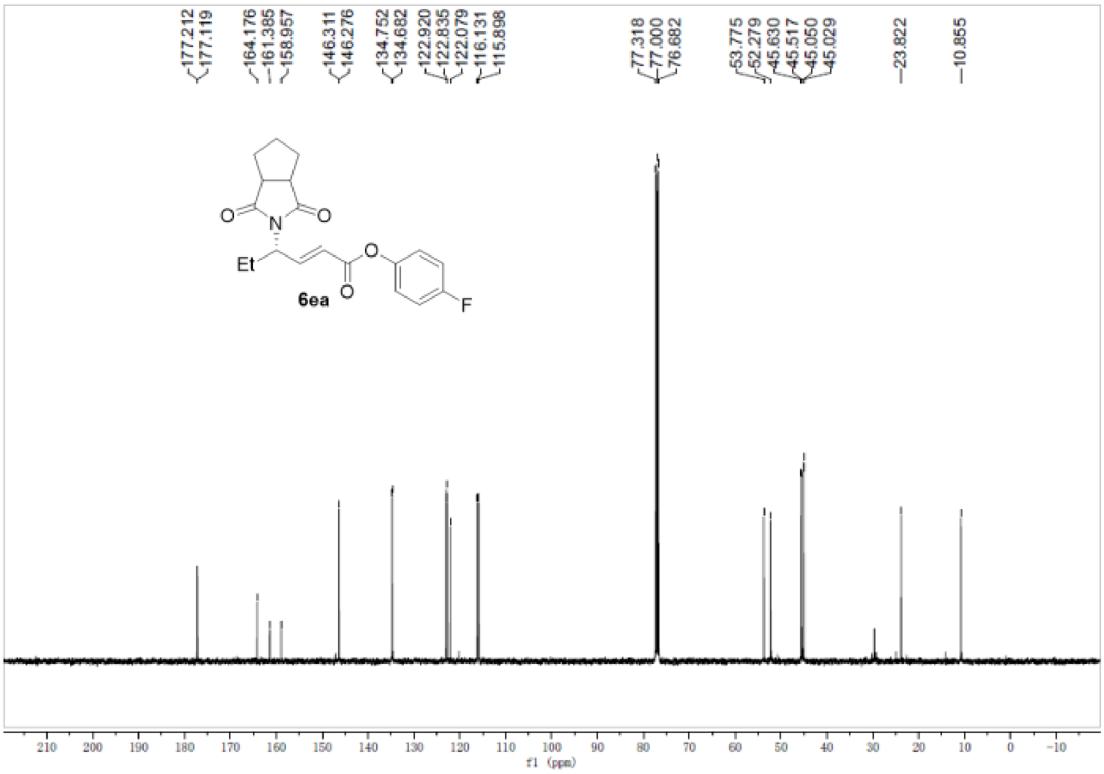

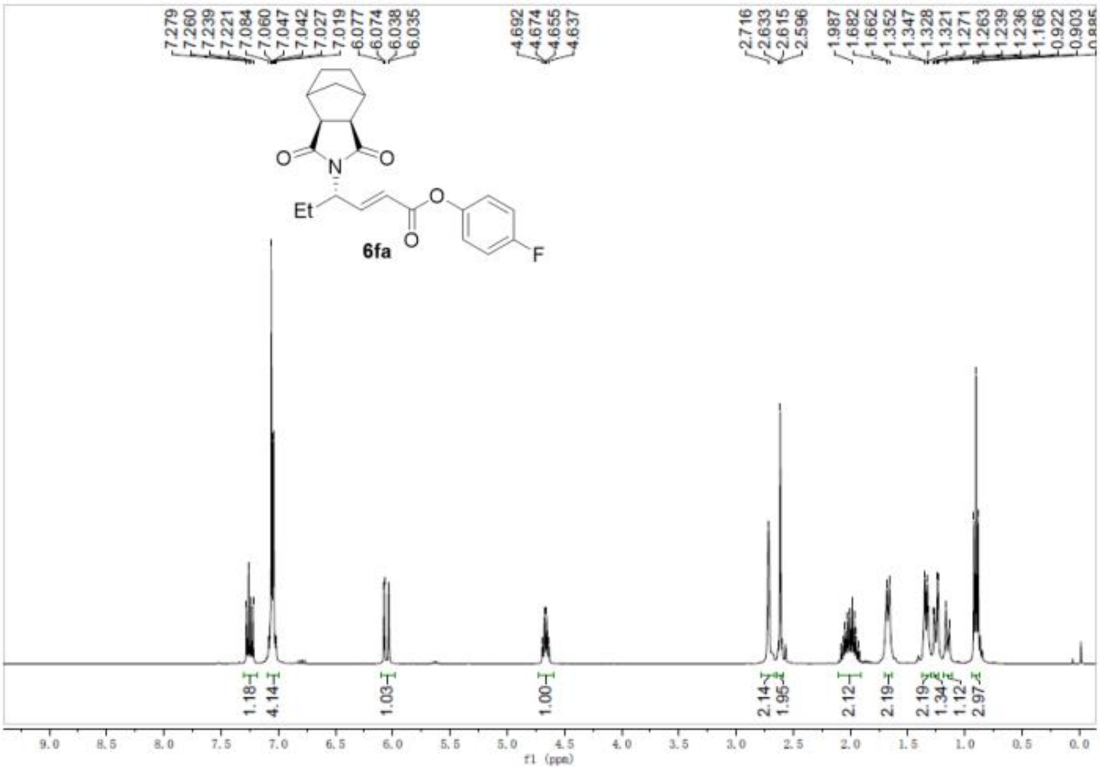
7.279
7.260
7.239
7.221
7.084
7.060
7.047
7.042
7.027
7.019
6.077
6.074
6.038
6.035
4.692
4.674
4.655
4.637
2.716
2.633
2.615
2.596
1.987
1.682
1.662
1.352
1.347
1.328
1.321
1.271
1.263
1.239
1.236
1.166
0.922
0.903
O
N
O
Et
O
O
6fa
F
1.18
4.14
1.03
1.00
2.14
1.95
2.12
2.19
2.19
1.34
1.12
2.97
9.0 8.5 8.0 7.5 7.0 6.5 6.0 5.5 5.0 4.5 4.0 3.5 3.0 2.5 2.0 1.5 1.0 0.5 0.0
f1 (ppm)

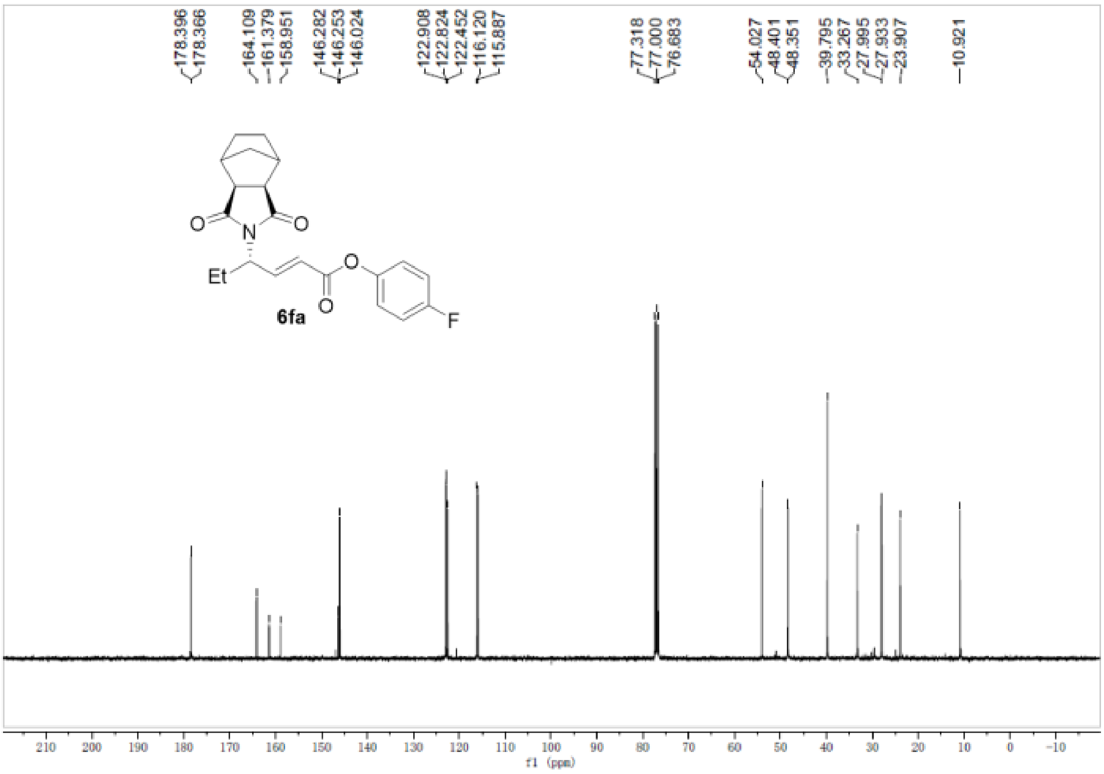
178.396
178.366
164.109
161.379
158.951
146.282
146.253
146.024
122.908
122.824
122.452
116.120
115.887
77.318
77.000
76.683
54.027
48.401
48.351
39.795
33.267
27.995
27.933
23.907
10.921
O
N
O
Et
O
O
6fa
F
210 200 190 180 170 160 150 140 130 120 110 100 90 80 70 60 50 40 30 20 10 0 -10
f1 (ppm)

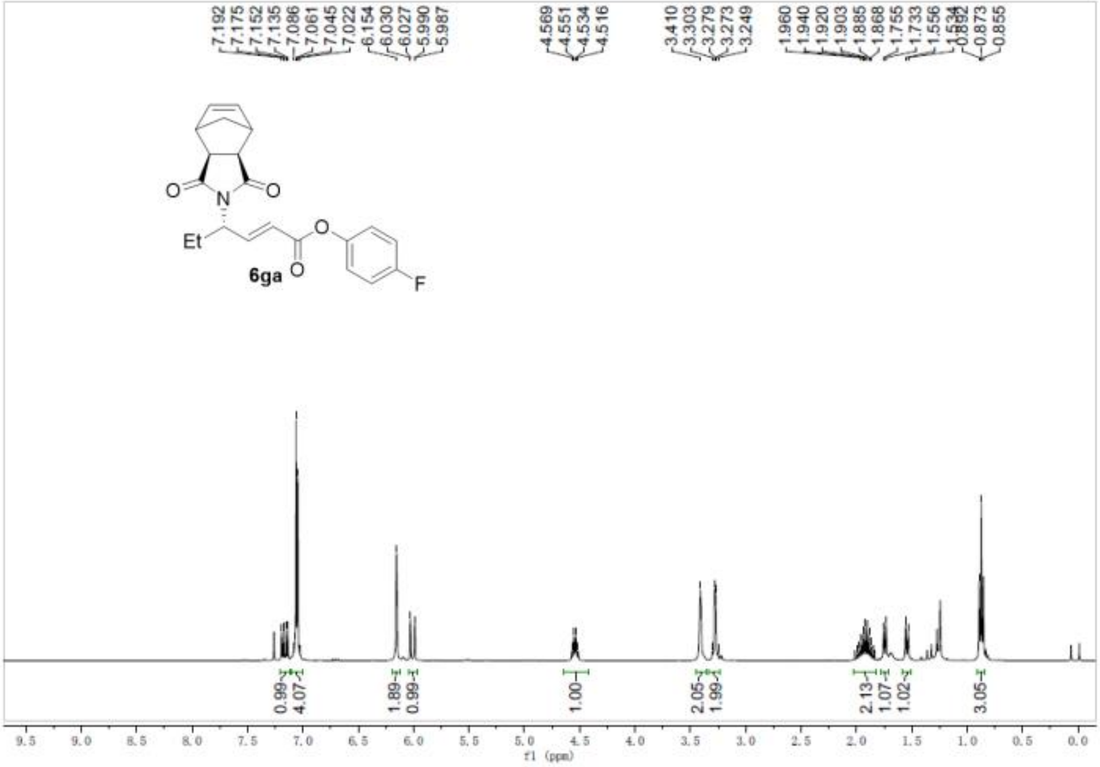

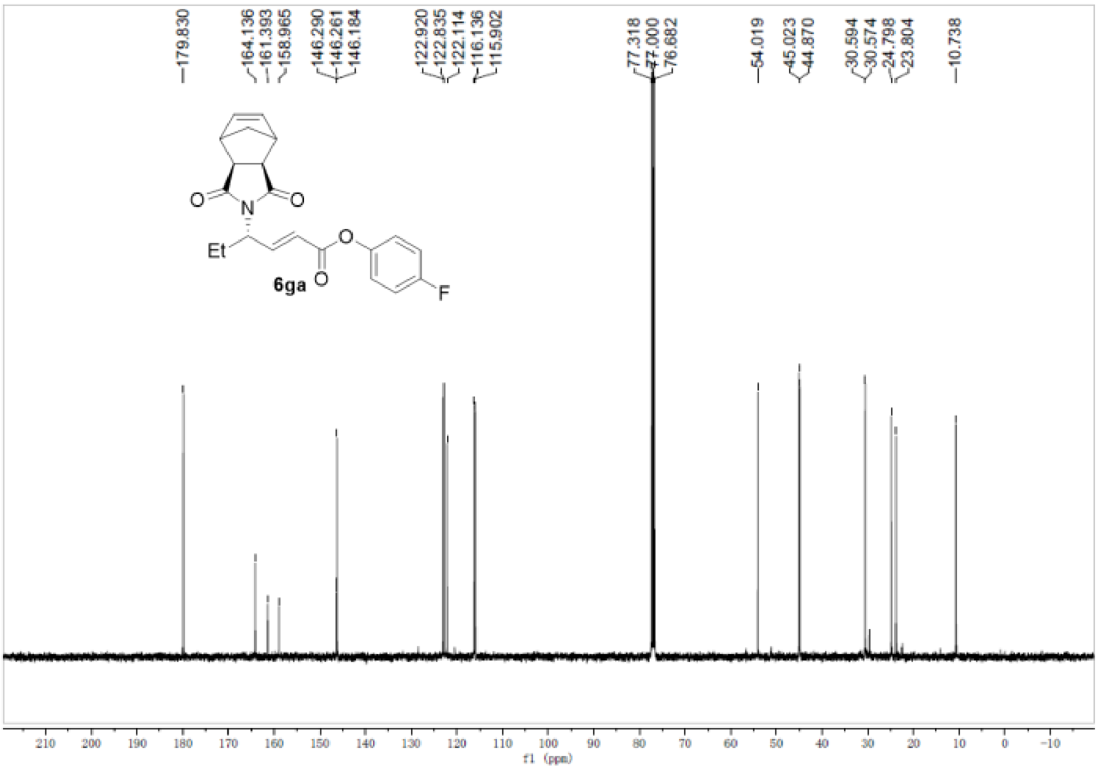

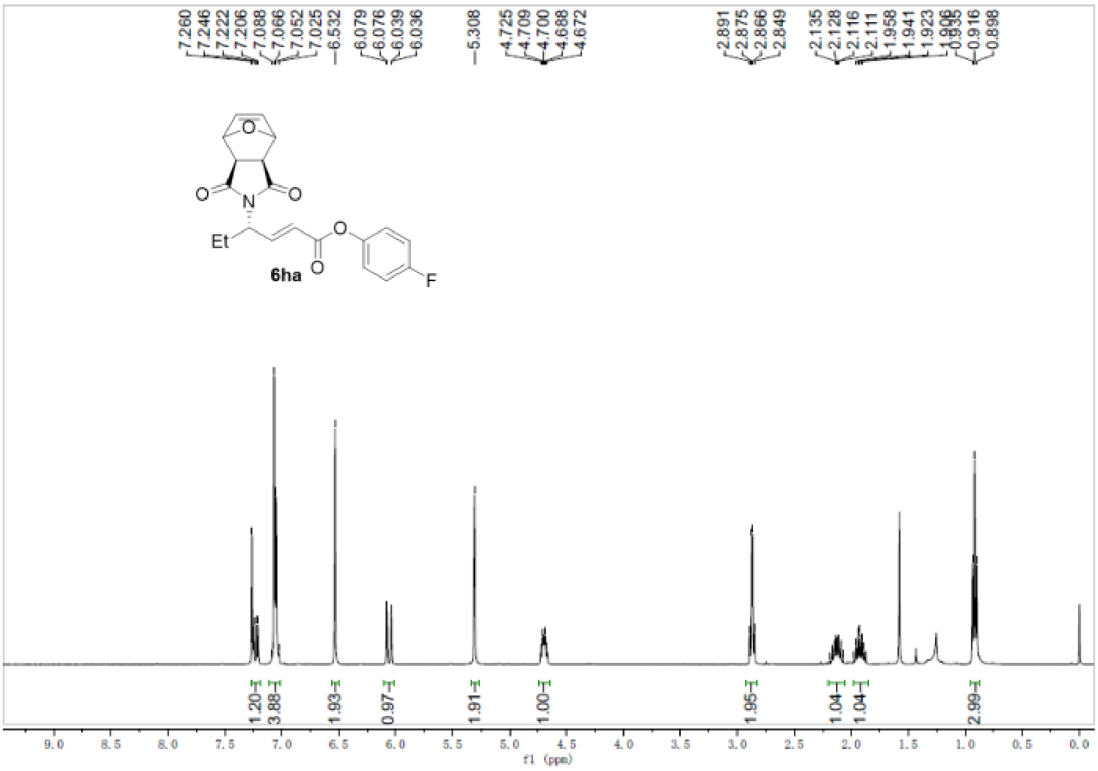
7.260
7.246
7.222
7.206
7.088
7.066
7.052
7.025
6.532
6.079
6.076
6.039
6.036
5.308
4.725
4.709
4.700
4.688
4.672
2.891
2.875
2.866
2.849
2.135
2.128
2.116
2.111
1.958
1.941
1.923
1.906
0.935
0.916
0.898
Et
6ha
F
1.20
3.88
1.93
0.97
1.91
1.00
1.95
1.04
1.04
2.99
f1 (ppm)

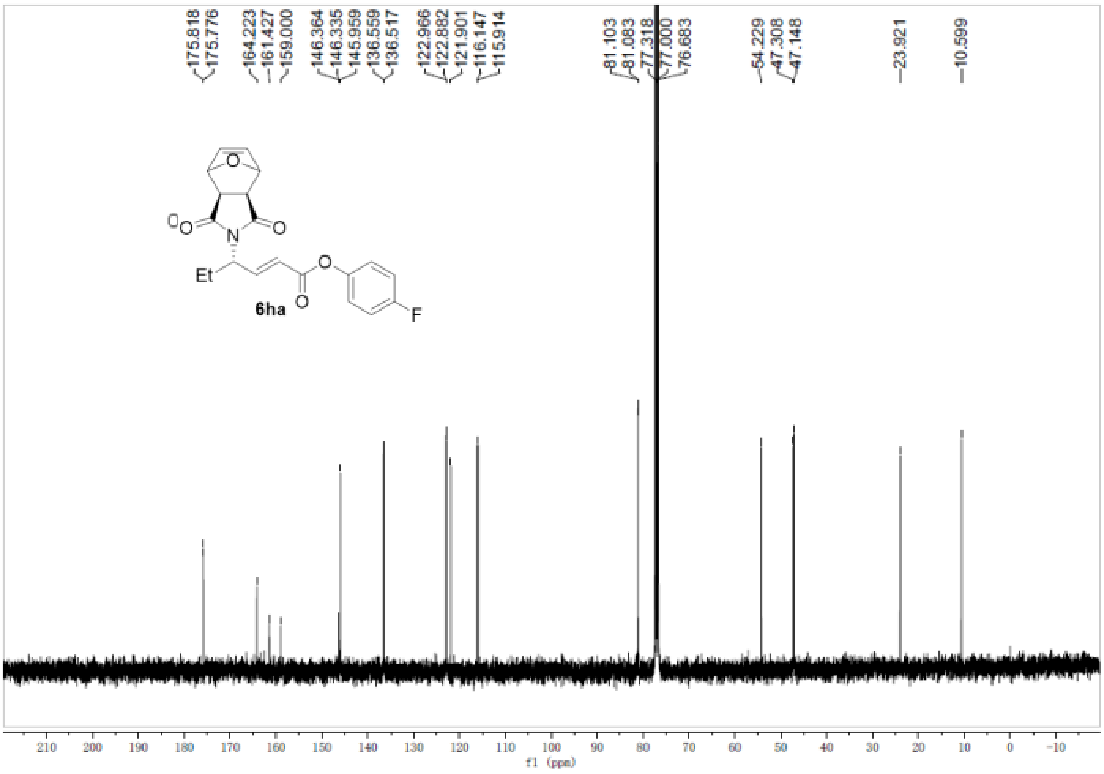
175.818
175.776
164.223
161.427
159.000
146.364
146.335
145.959
136.559
136.517
122.966
122.882
121.901
116.147
115.914
81.103
81.083
77.318
77.000
76.683
54.229
47.308
47.148
23.921
10.599
Et
6ha
F
f1 (ppm)

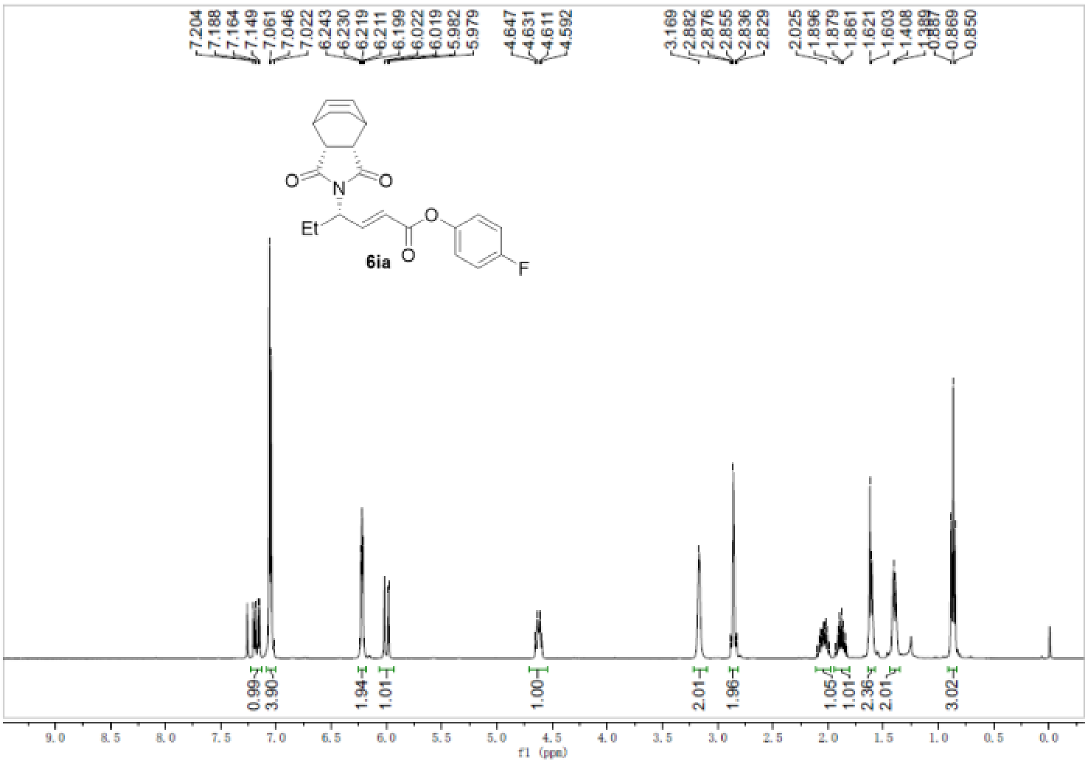

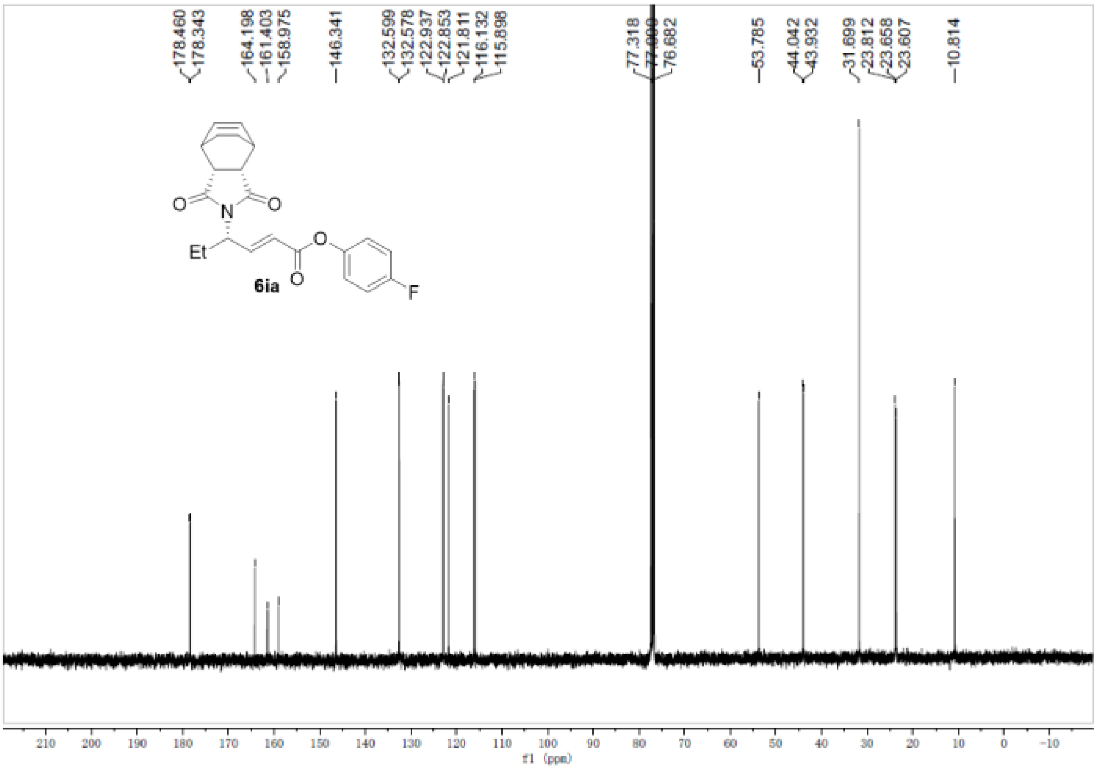

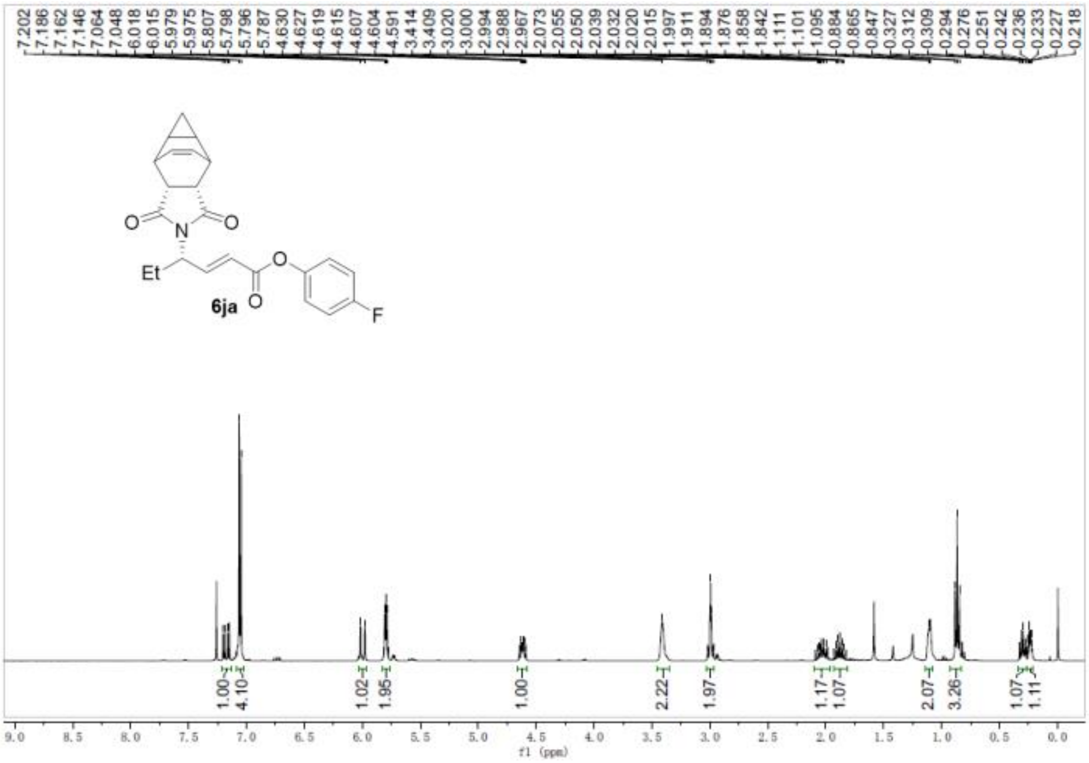

7.202 7.186 7.162 7.146 7.064 7.048 6.018 6.015 5.979 5.975 5.807 5.798 5.796 5.787 4.630 4.627 4.619 4.615 4.607 4.604 4.591 3.414 3.409 3.020 3.000 2.994 2.988 2.967 2.073 2.055 2.050 2.039 2.032 2.020 2.015 1.997 1.911 1.894 1.876 1.858 1.842 1.111 1.101 1.095 0.884 0.865 0.847 0.327 0.312 0.309 0.294 0.276 0.251 0.242 0.236 0.233 0.227 0.218
Et
6ja
F
1.00 4.10 1.02 1.95 1.00 2.22 1.97 1.17 1.07 2.07 3.26 1.07 1.11
f1 (ppm)

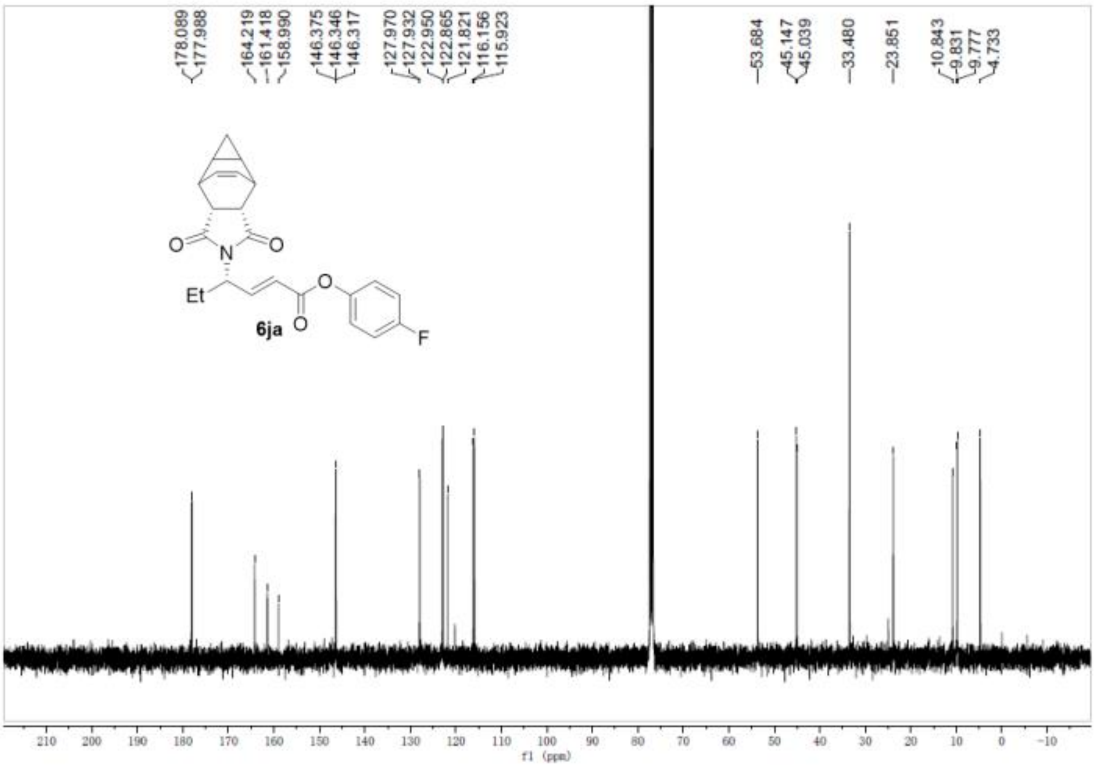

178.089 177.988 164.219 161.418 158.990 146.375 146.346 146.317 127.970 127.932 122.950 122.865 121.821 116.156 115.923 53.684 45.147 45.039 33.480 23.851 10.843 9.831 9.777 4.733
Et
6ja
F
f1 (ppm)

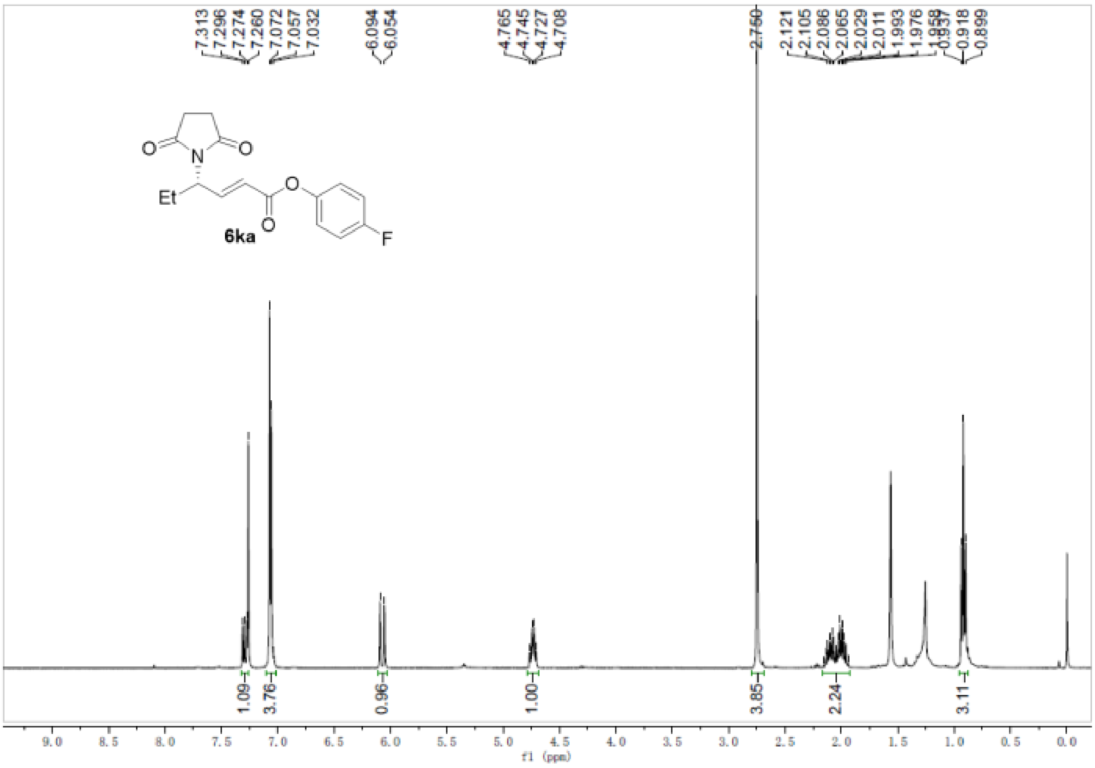

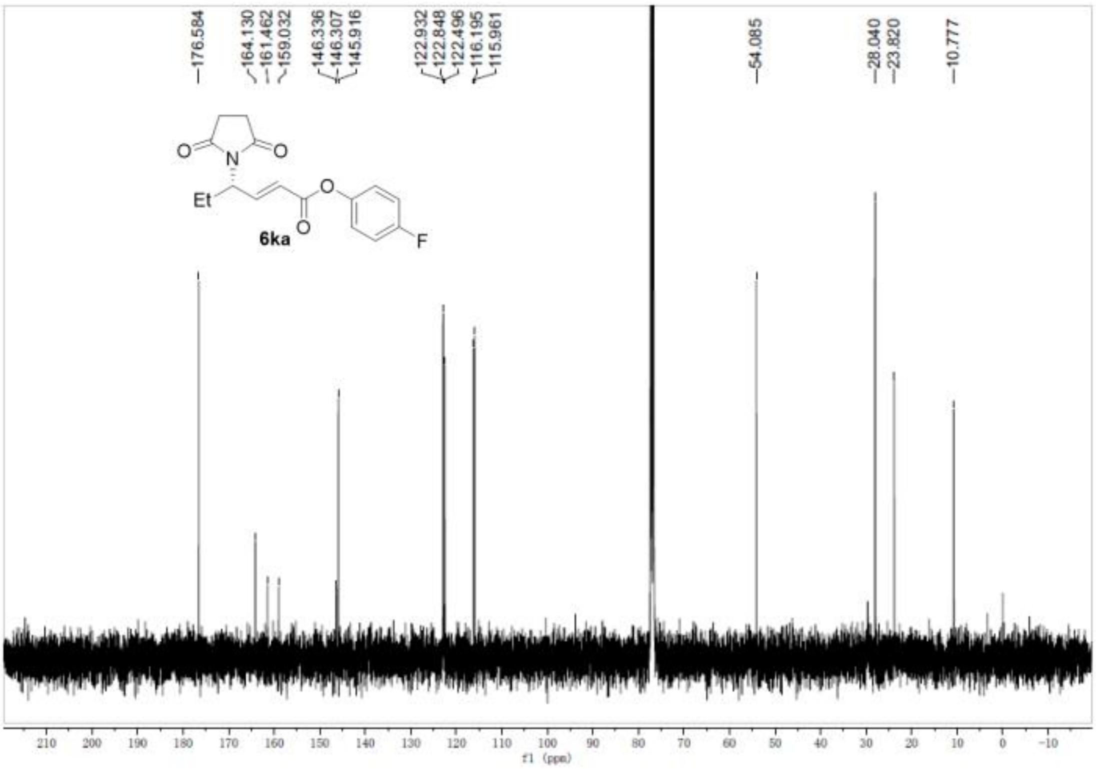

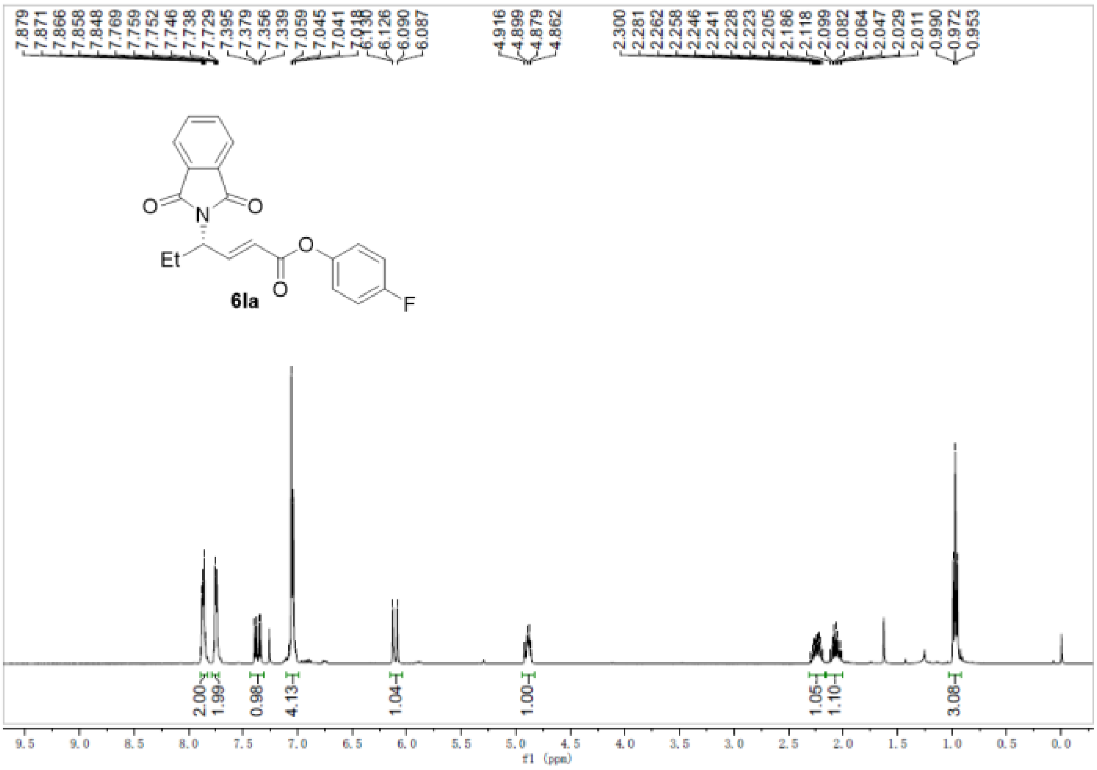
6la
Et
N
O
F
f1 (ppm)

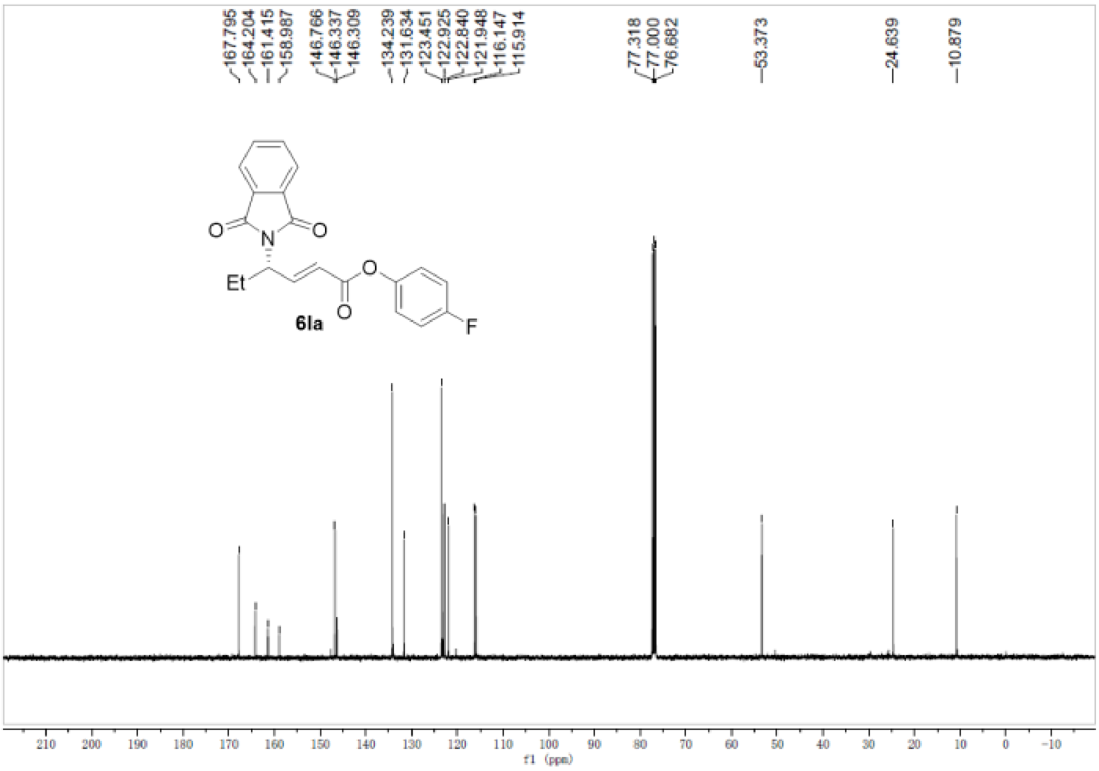
6la
Et
N
O
F
f1 (ppm)

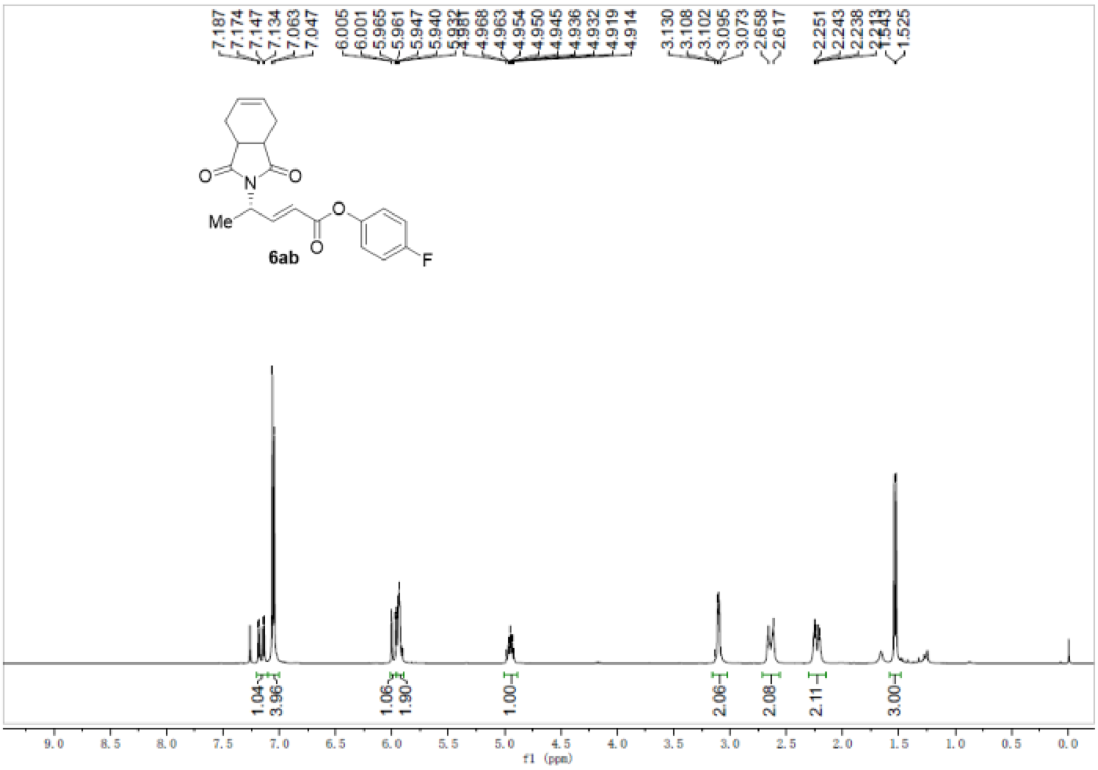

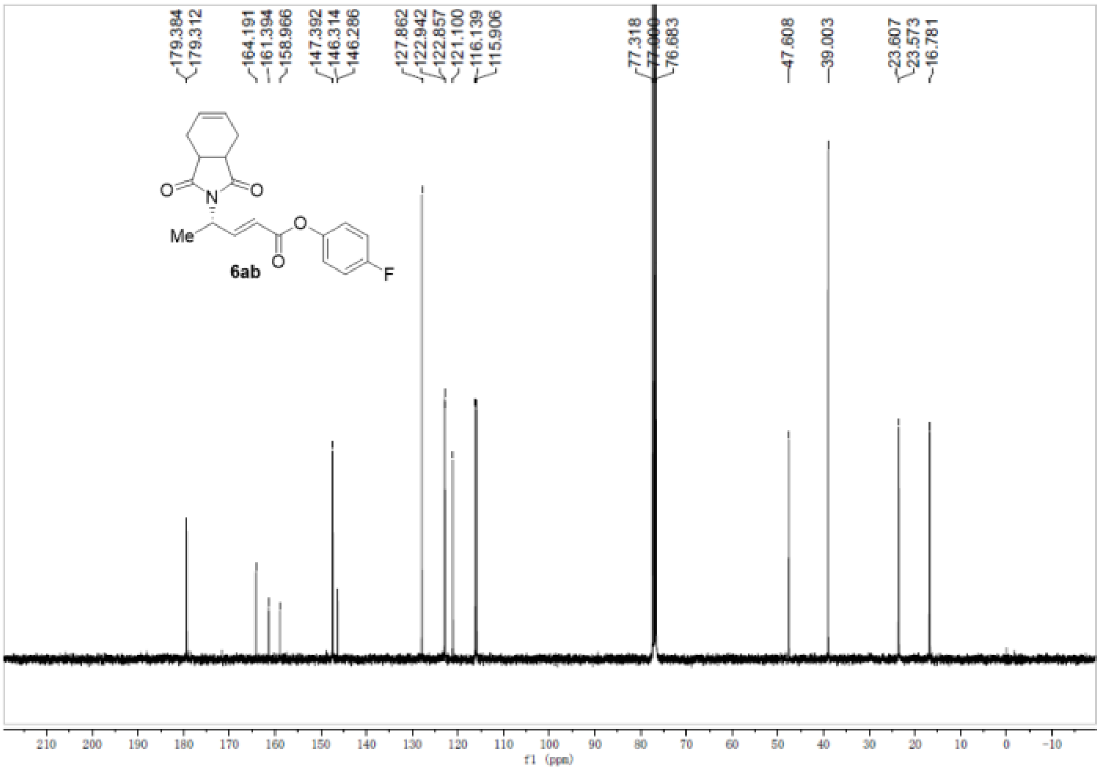

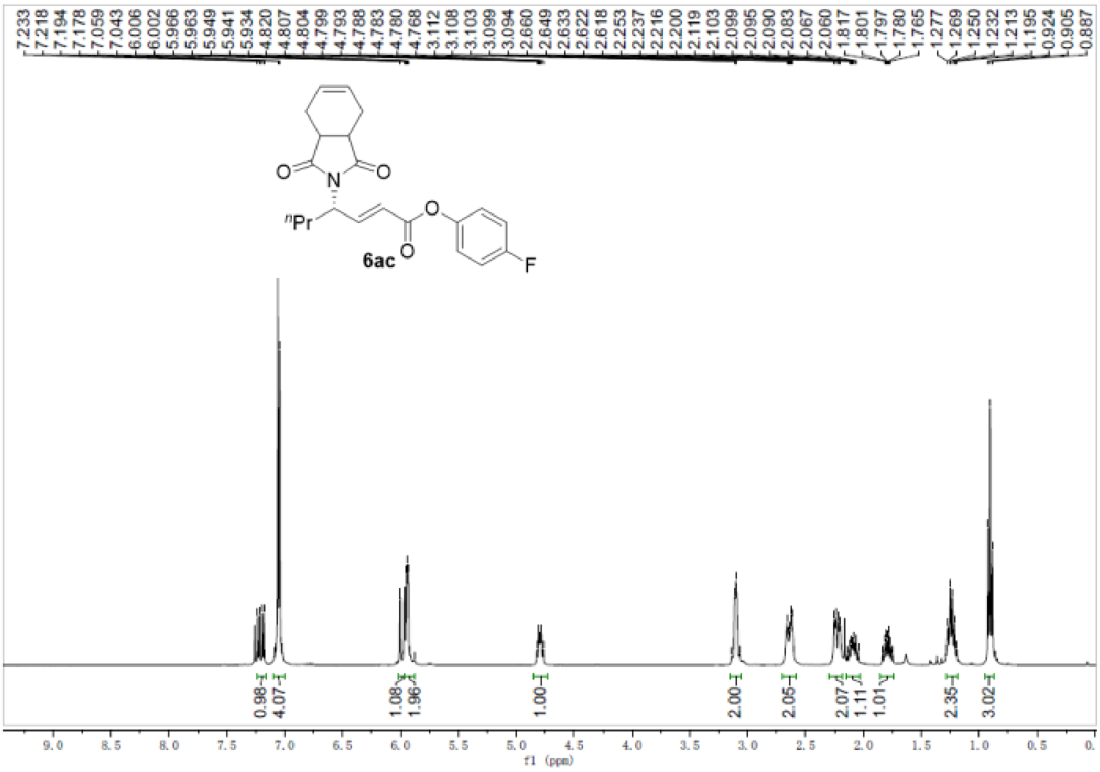

7.233
7.218
7.194
7.178
7.059
7.043
6.006
6.002
5.966
5.963
5.949
5.941
5.934
4.820
4.807
4.804
4.799
4.793
4.788
4.783
4.780
4.768
3.112
3.108
3.103
3.099
3.094
2.660
2.649
2.633
2.622
2.618
2.253
2.237
2.216
2.200
2.119
2.103
2.099
2.095
2.090
2.083
2.067
2.060
1.817
1.801
1.797
1.780
1.765
1.277
1.269
1.250
1.232
1.213
1.195
0.924
0.905
0.887
O
N
O
^{n}Pr
O
O
F
6ac
0.98
4.07
1.08
1.96
1.00
2.00
2.05
2.07
1.11
1.01
2.35
3.02
9.0 8.5 8.0 7.5 7.0 6.5 6.0 5.5 5.0 4.5 4.0 3.5 3.0 2.5 2.0 1.5 1.0 0.5 0.
f1 (ppm)

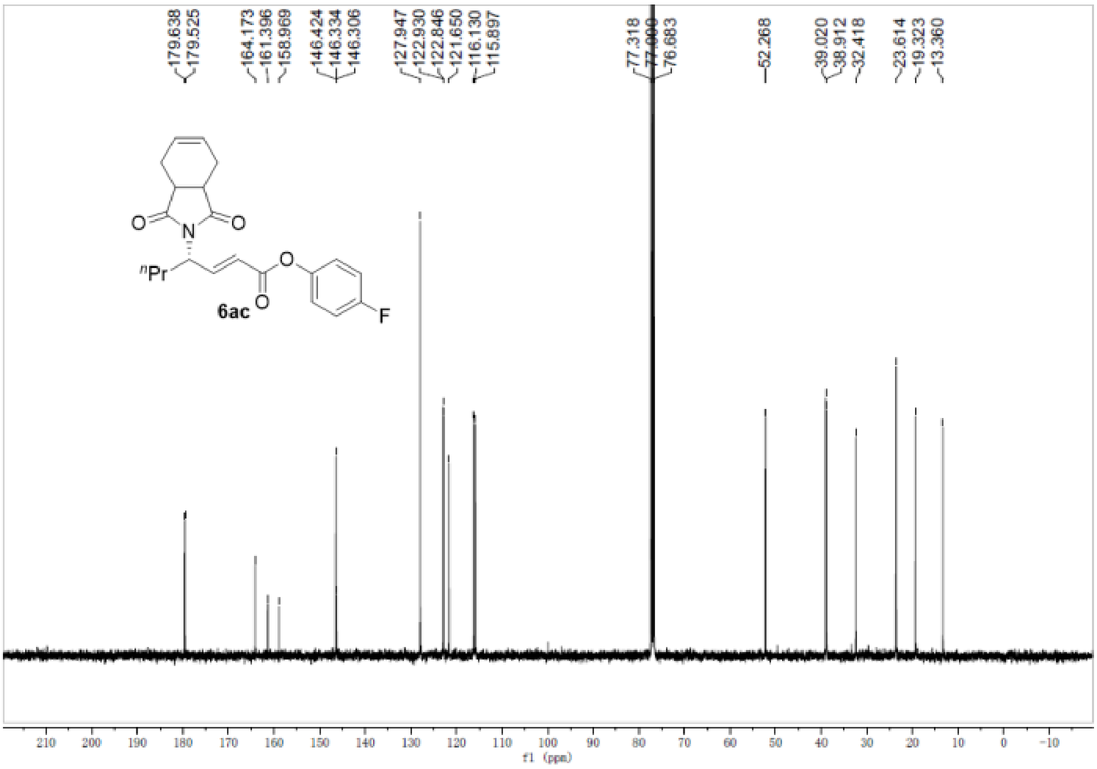

179.638
179.525
164.173
161.396
158.969
146.424
146.334
146.306
127.947
122.930
122.846
121.650
116.130
115.897
77.318
77.000
76.683
52.268
39.020
38.912
32.418
23.614
19.323
13.360
O
N
O
^{n}Pr
O
O
F
6ac
210 200 190 180 170 160 150 140 130 120 110 100 90 80 70 60 50 40 30 20 10 0 -10
f1 (ppm)

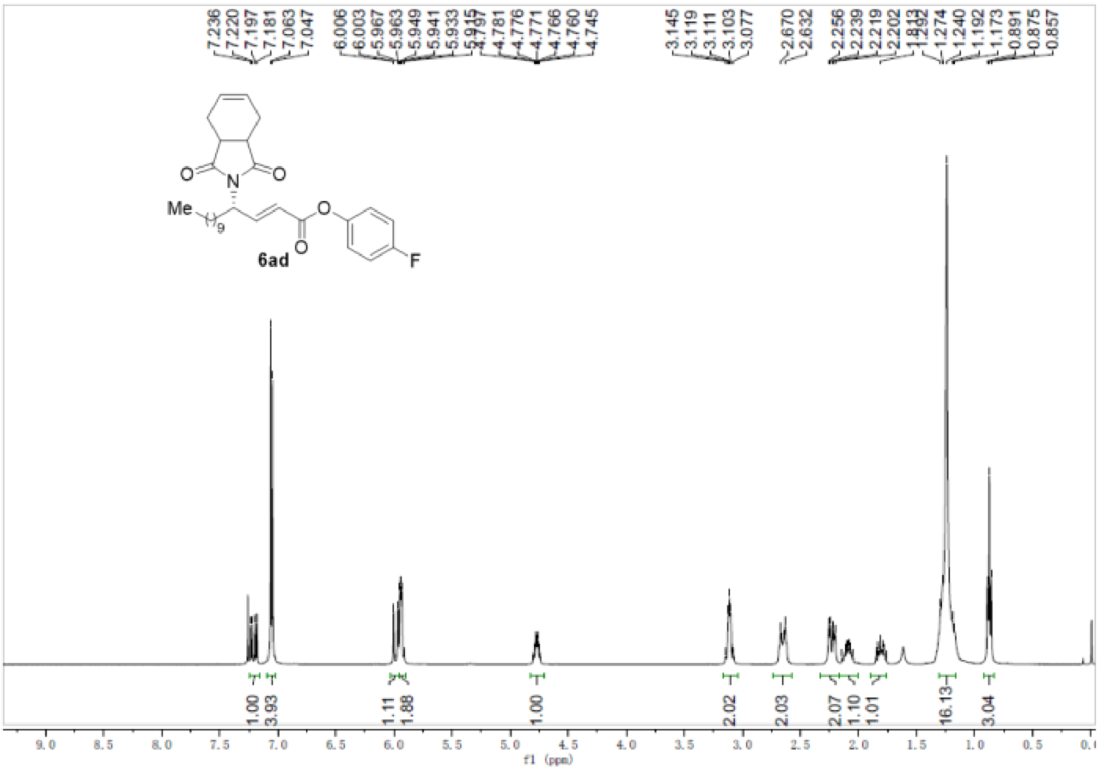

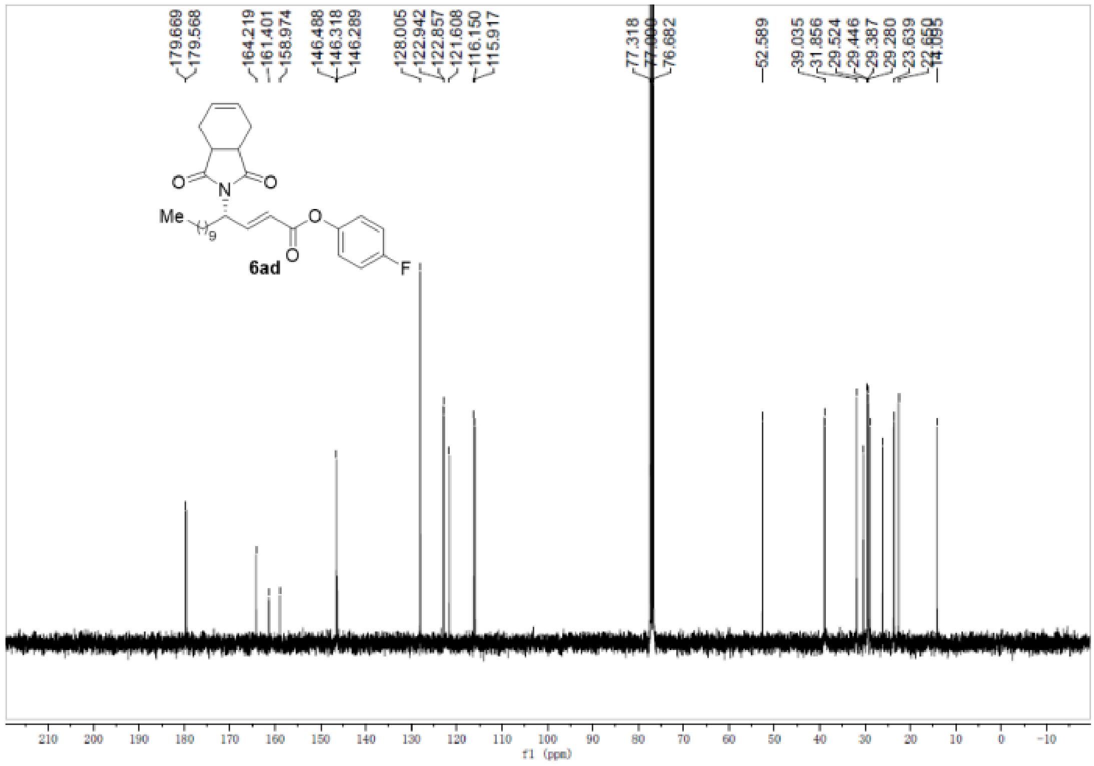

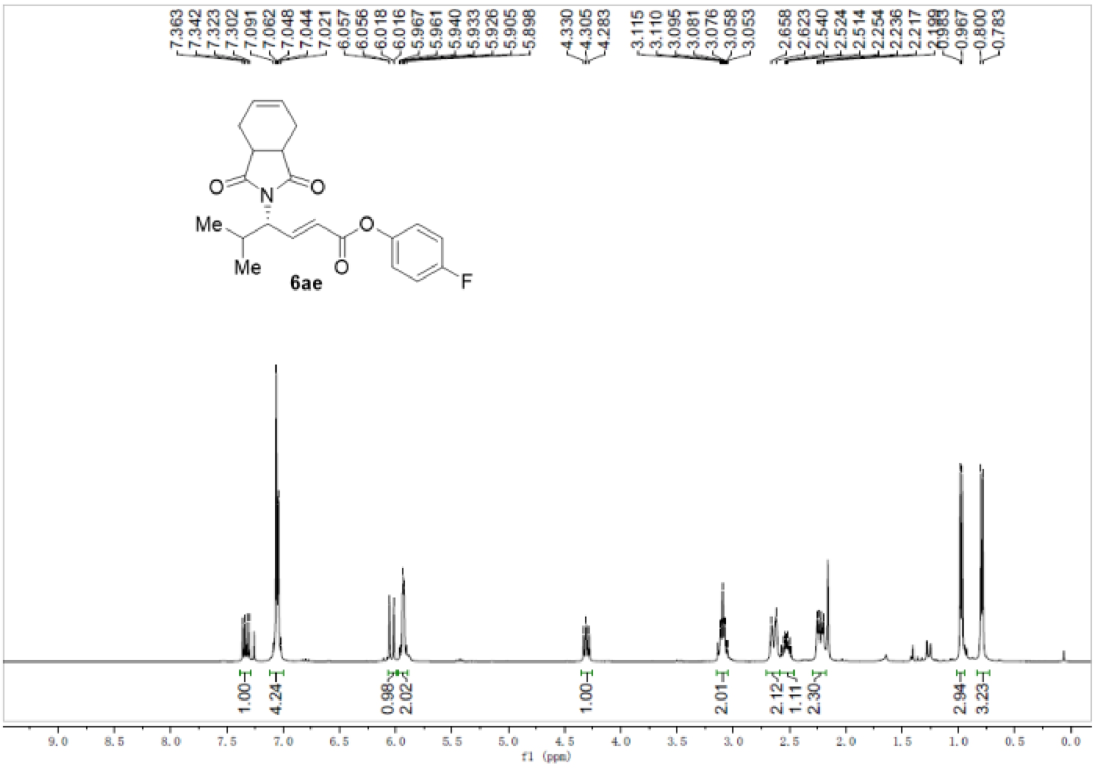
7.363
7.342
7.323
7.302
7.091
7.062
7.048
7.044
7.021
6.057
6.056
6.018
6.016
5.967
5.961
5.940
5.933
5.926
5.905
5.898
4.330
4.305
4.283
3.115
3.110
3.095
3.081
3.076
3.058
3.053
2.658
2.623
2.540
2.524
2.514
2.254
2.236
2.217
0.967
0.800
0.783
Me
Me
N
O
O
O
O
F
6ae
1.00
4.24
0.98
2.02
1.00
2.01
2.12
1.11
2.30
2.94
3.23
f1 (ppm)

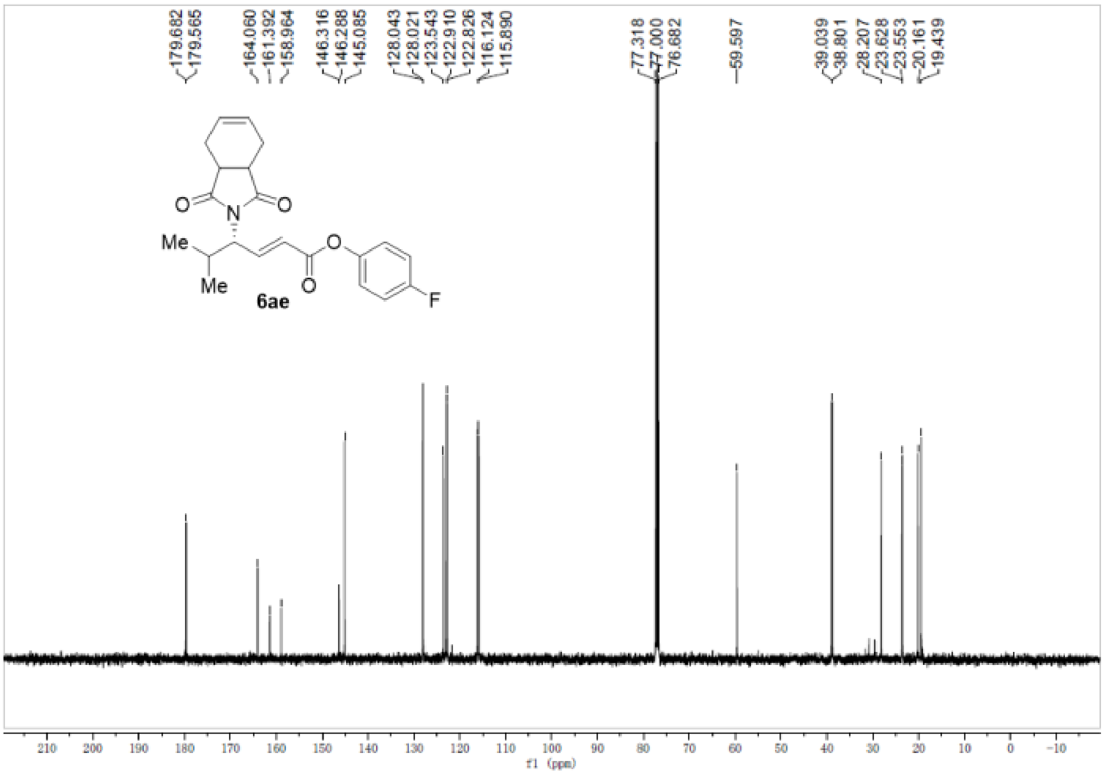
179.682
179.565
164.060
161.392
158.964
146.316
146.288
145.085
128.043
128.021
123.543
122.910
122.826
116.124
115.890
77.318
77.000
76.682
59.597
39.039
38.801
28.207
23.628
23.553
20.161
19.439
Me
Me
N
O
O
O
O
F
6ae
f1 (ppm)

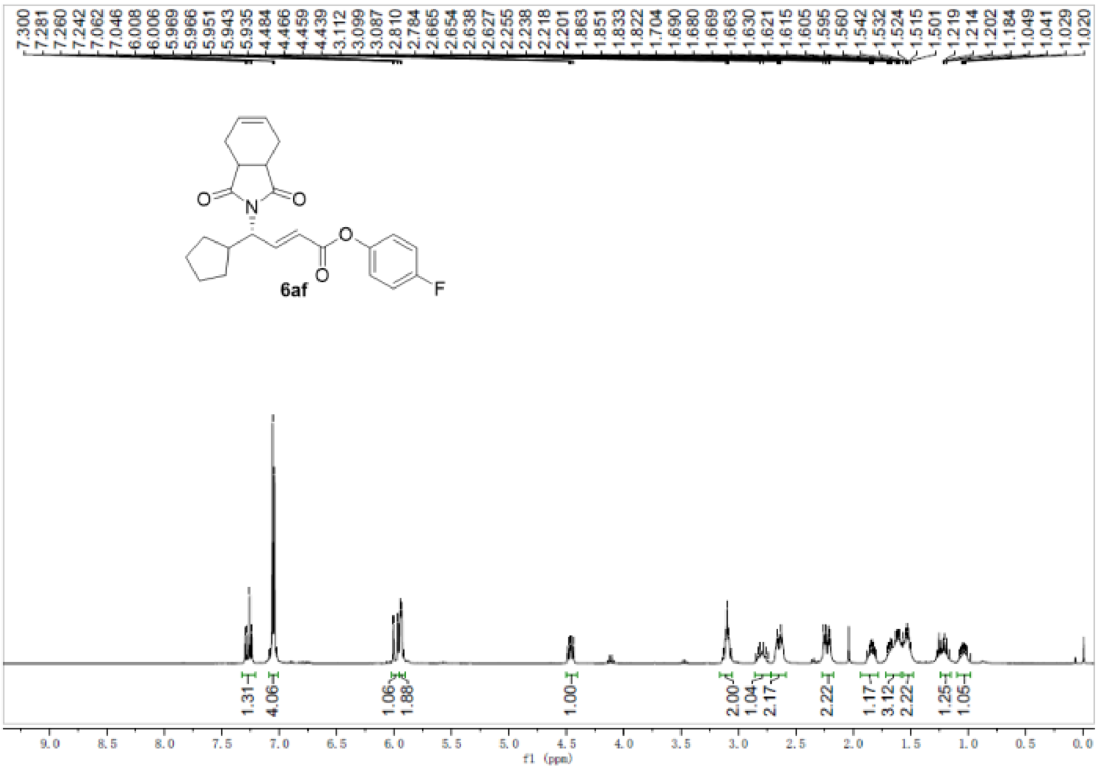

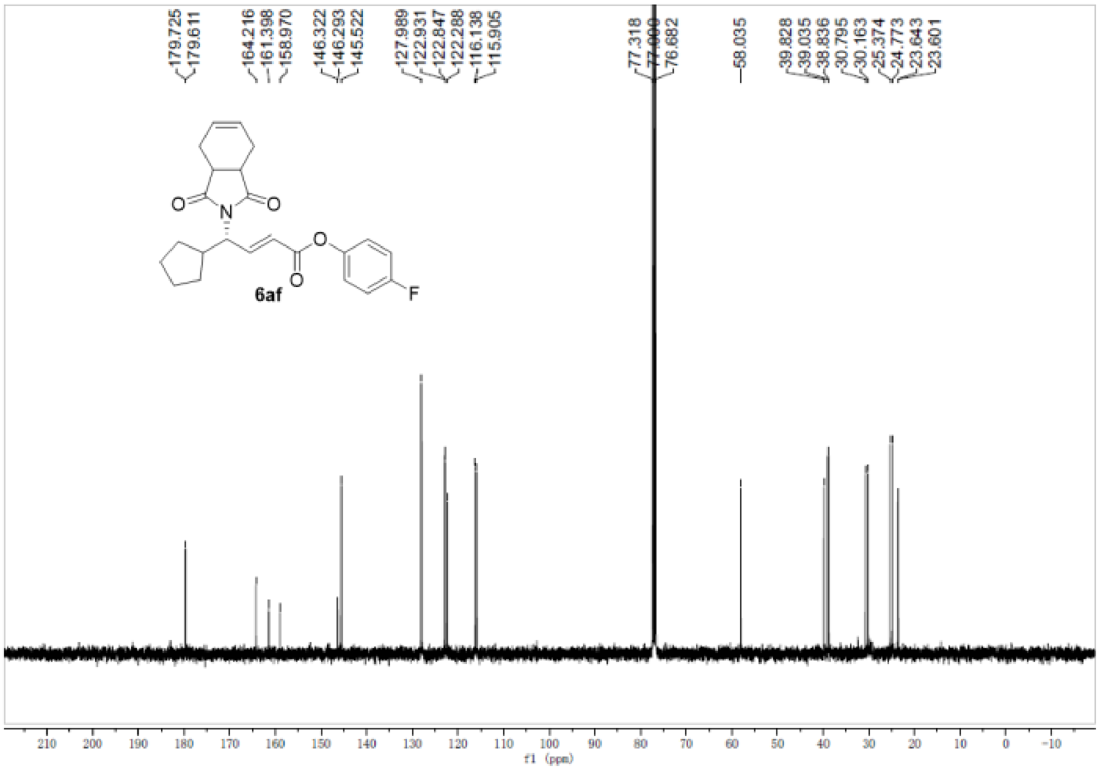

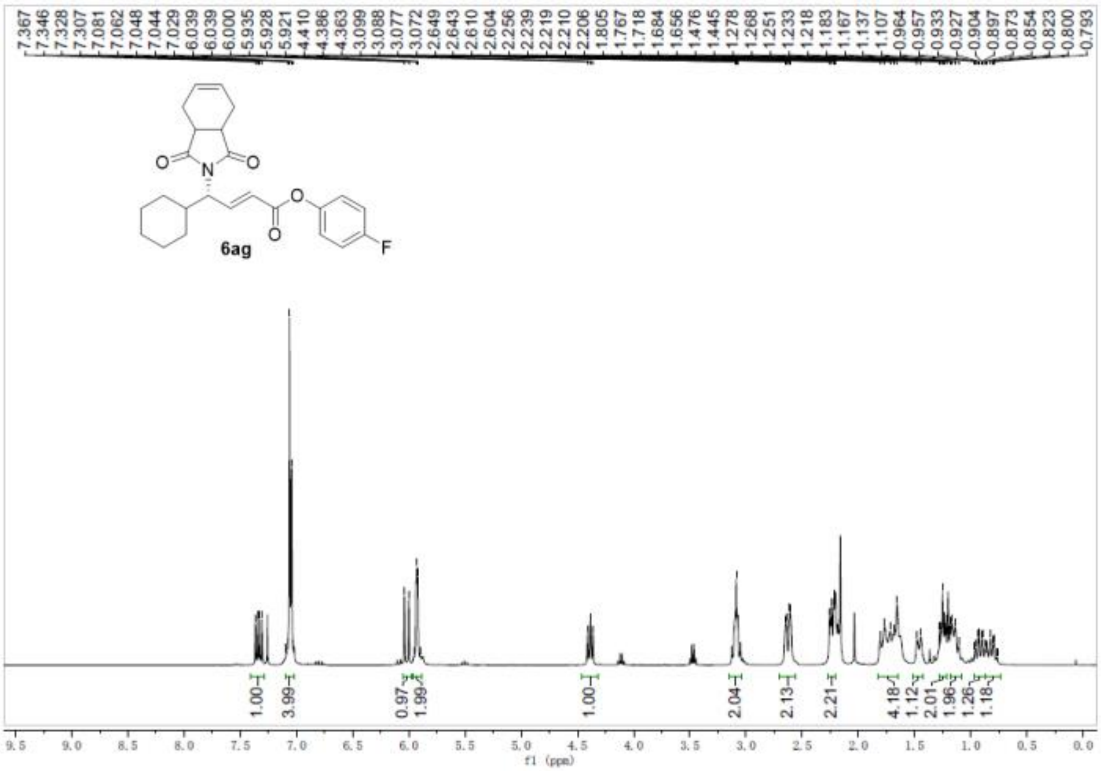
7.367
7.346
7.328
7.307
7.081
7.062
7.048
7.044
7.029
6.039
6.039
6.000
5.935
5.928
5.921
4.410
4.386
4.363
3.099
3.088
3.077
3.072
2.649
2.643
2.610
2.604
2.256
2.239
2.219
2.210
2.206
1.805
1.767
1.718
1.684
1.656
1.476
1.445
1.278
1.268
1.251
1.233
1.218
1.183
1.167
1.137
1.107
0.964
0.957
0.933
0.927
0.904
0.897
0.873
0.854
0.823
0.800
0.793
O
N
O
O
O
F
6ag
1.00
3.99
0.97
1.99
1.00
2.04
2.13
2.21
4.18
1.12
2.01
1.96
1.26
1.18
f1 (ppm)

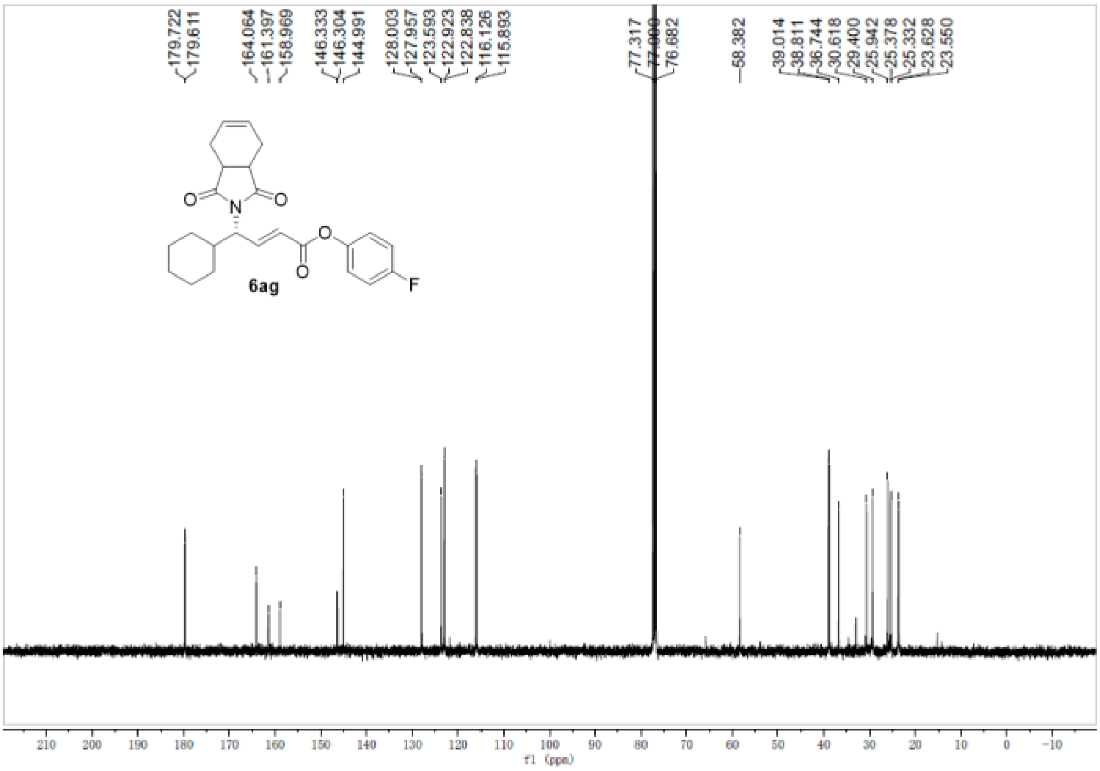
179.722
179.611
164.064
161.397
158.969
146.333
146.304
144.991
128.003
127.957
123.593
122.923
122.838
116.126
115.893
77.317
76.682
58.382
39.014
38.811
36.744
30.618
29.400
25.942
25.378
25.332
23.628
23.550
O
N
O
O
O
F
6ag
f1 (ppm)

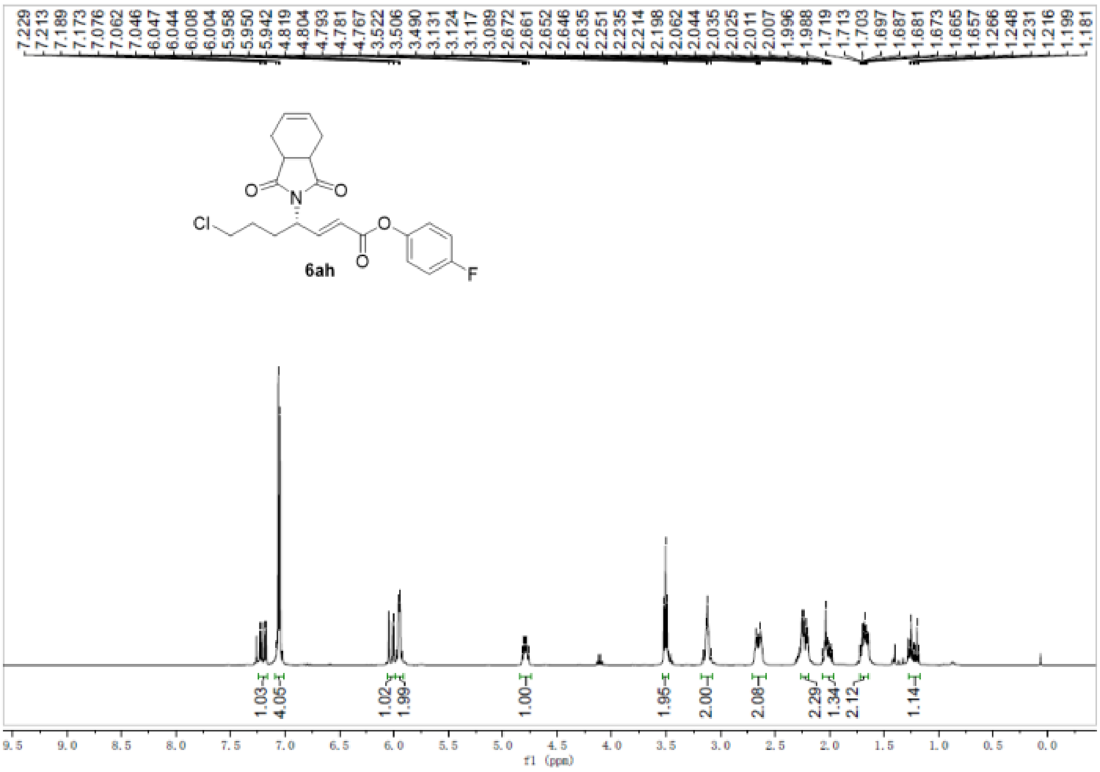

6ah

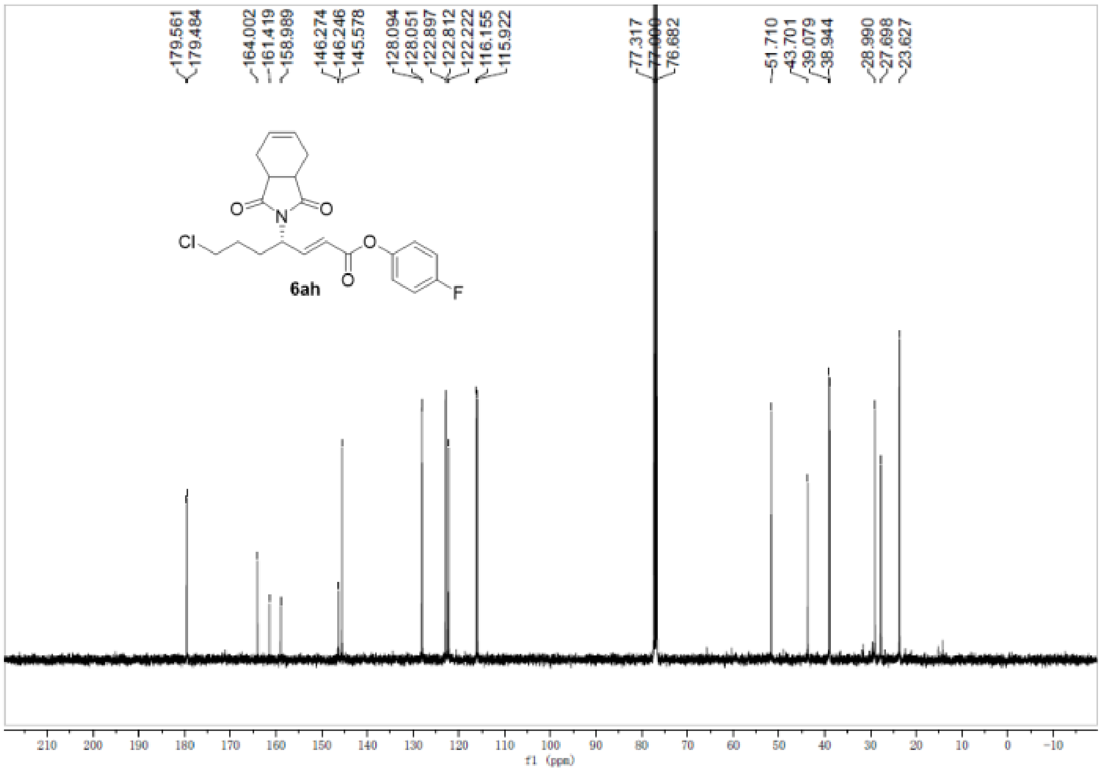

6ah

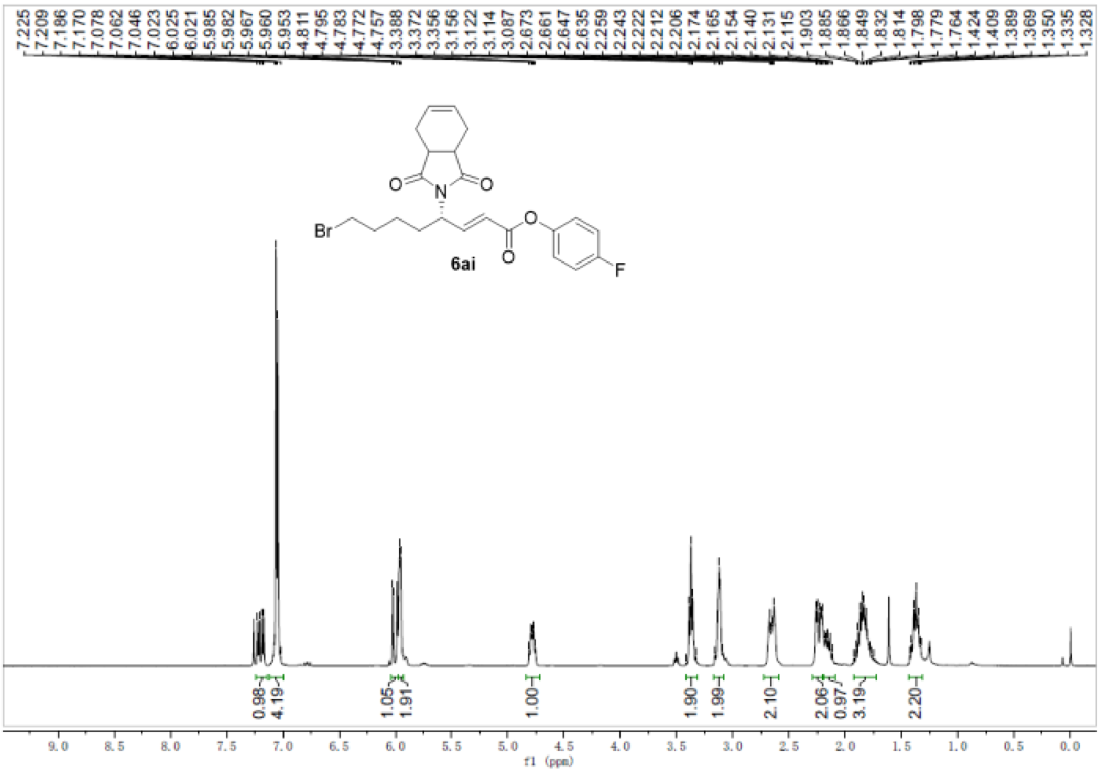

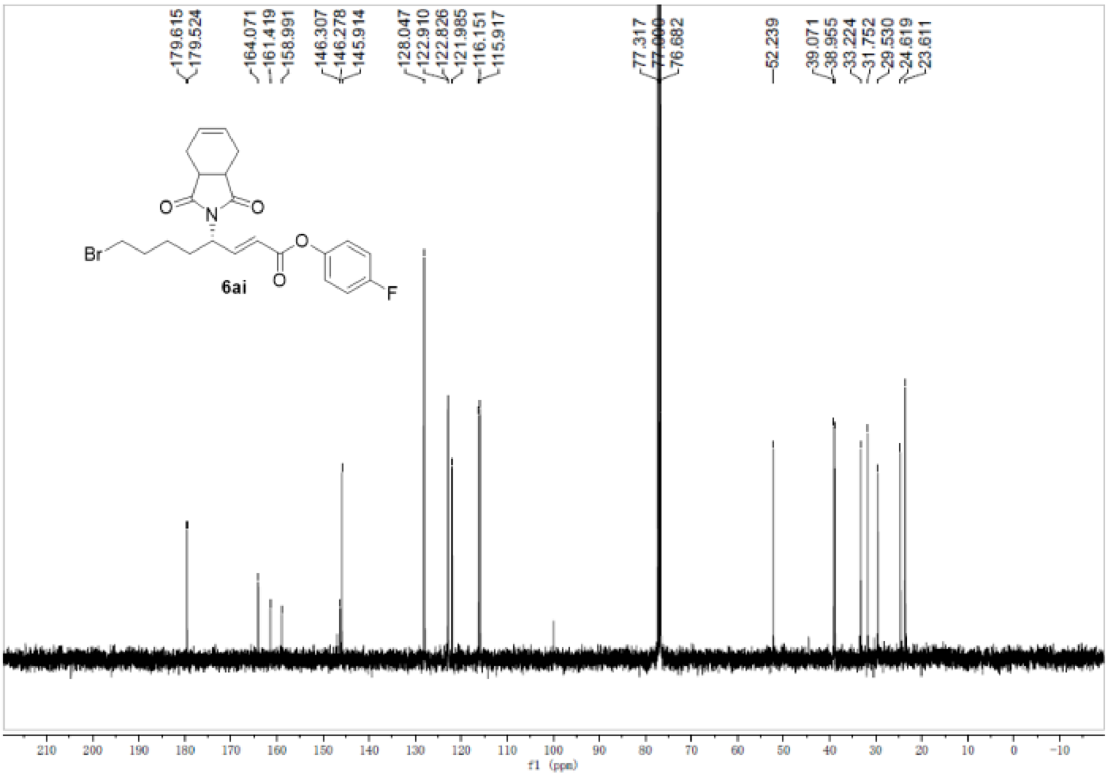

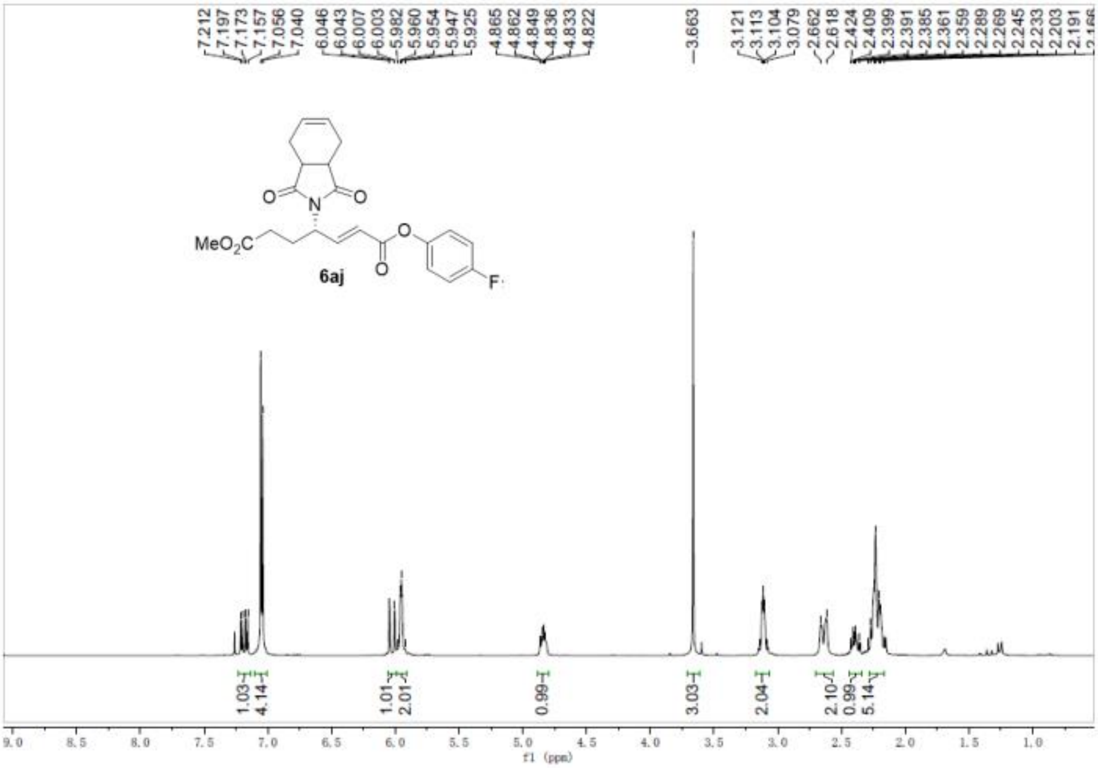
7.212
7.197
7.173
7.157
7.056
7.040
6.046
6.043
6.007
6.003
5.982
5.960
5.954
5.947
5.925
4.865
4.862
4.849
4.836
4.833
4.822
3.663
3.121
3.113
3.104
3.079
2.662
2.618
2.424
2.409
2.399
2.391
2.385
2.361
2.359
2.289
2.269
2.245
2.233
2.203
2.191
[illegible]
MeO2C
N
O
F
6aj
1.03
4.14
1.01
2.01
0.99
3.03
2.04
2.10
0.99
5.14
f1 (ppm)

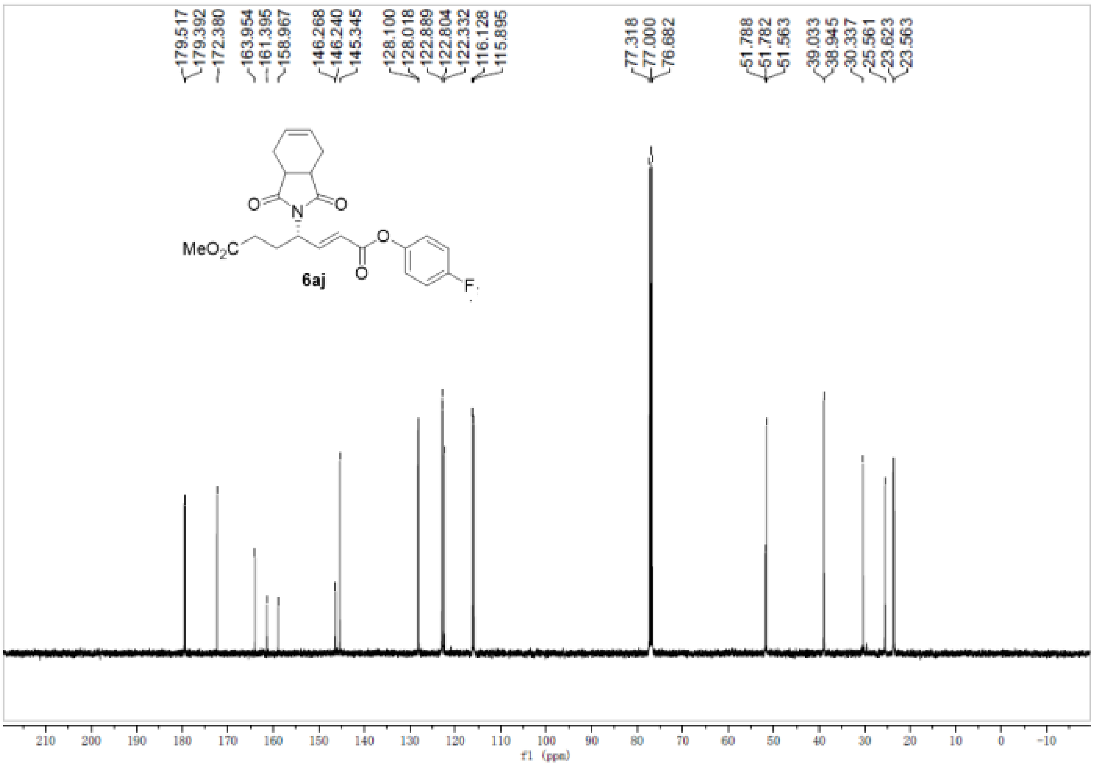
179.517
179.392
172.380
163.954
161.395
158.967
146.268
146.240
145.345
128.100
128.018
122.889
122.804
122.332
116.128
115.895
77.318
77.000
76.682
51.788
51.782
51.563
39.033
38.945
30.337
25.561
23.623
23.563
MeO2C
N
O
F
6aj
f1 (ppm)

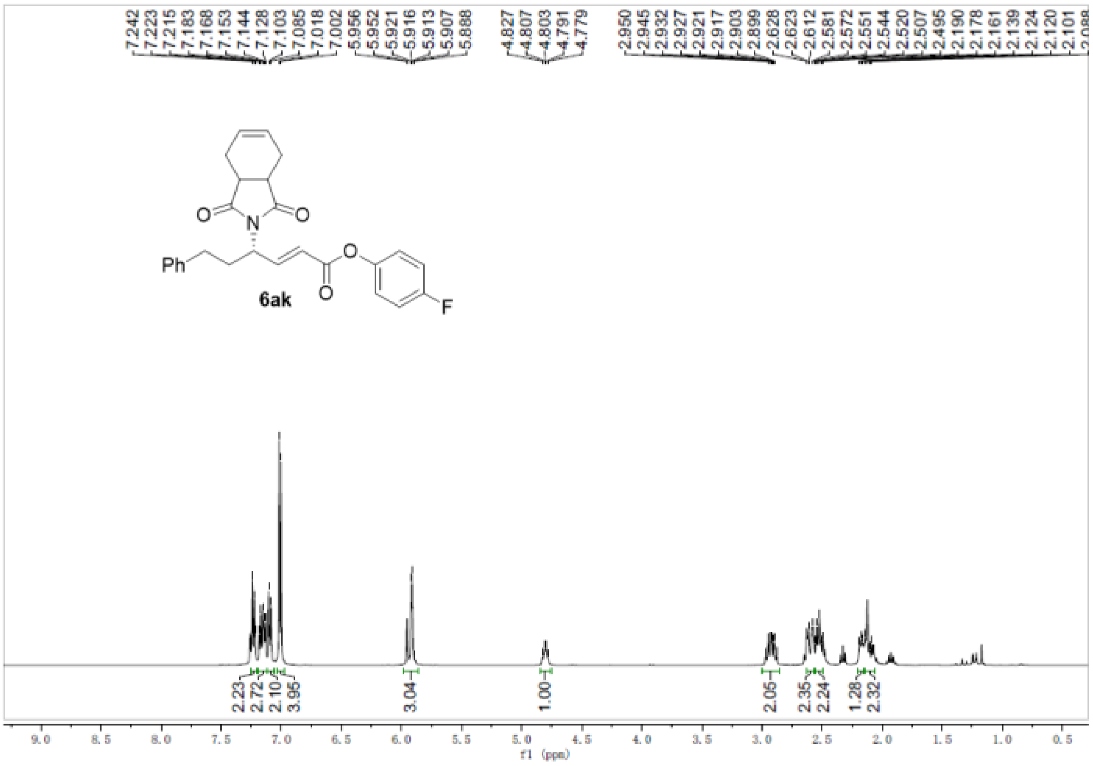

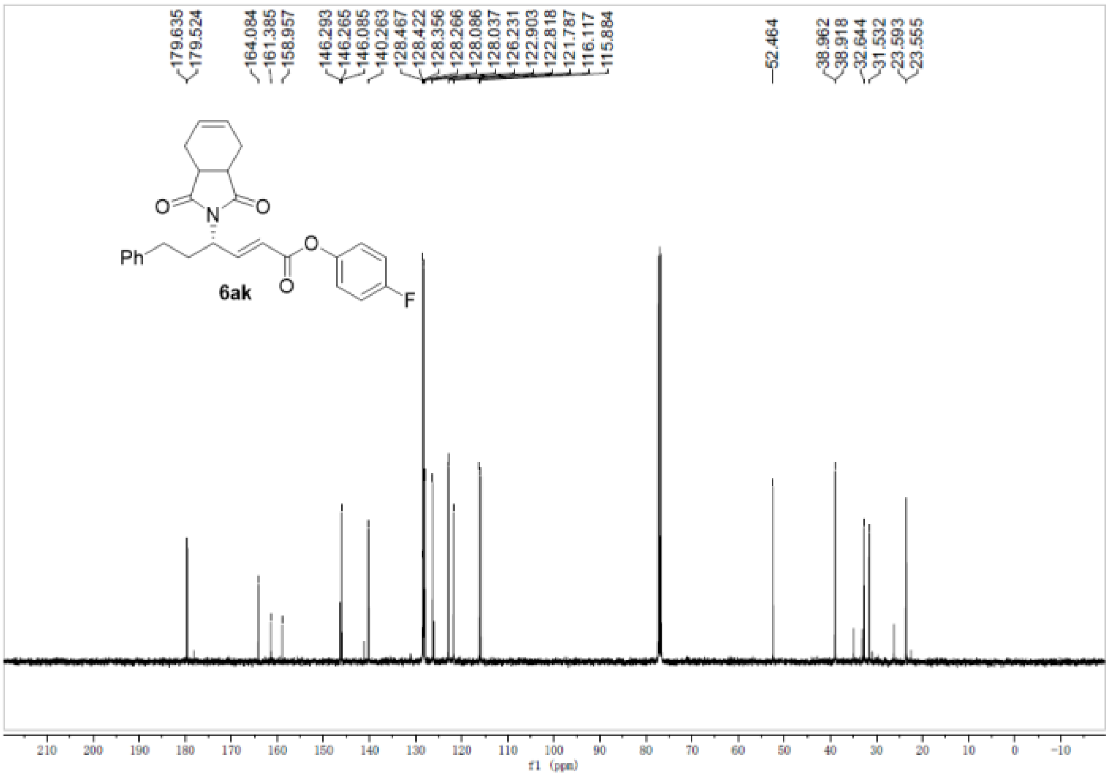

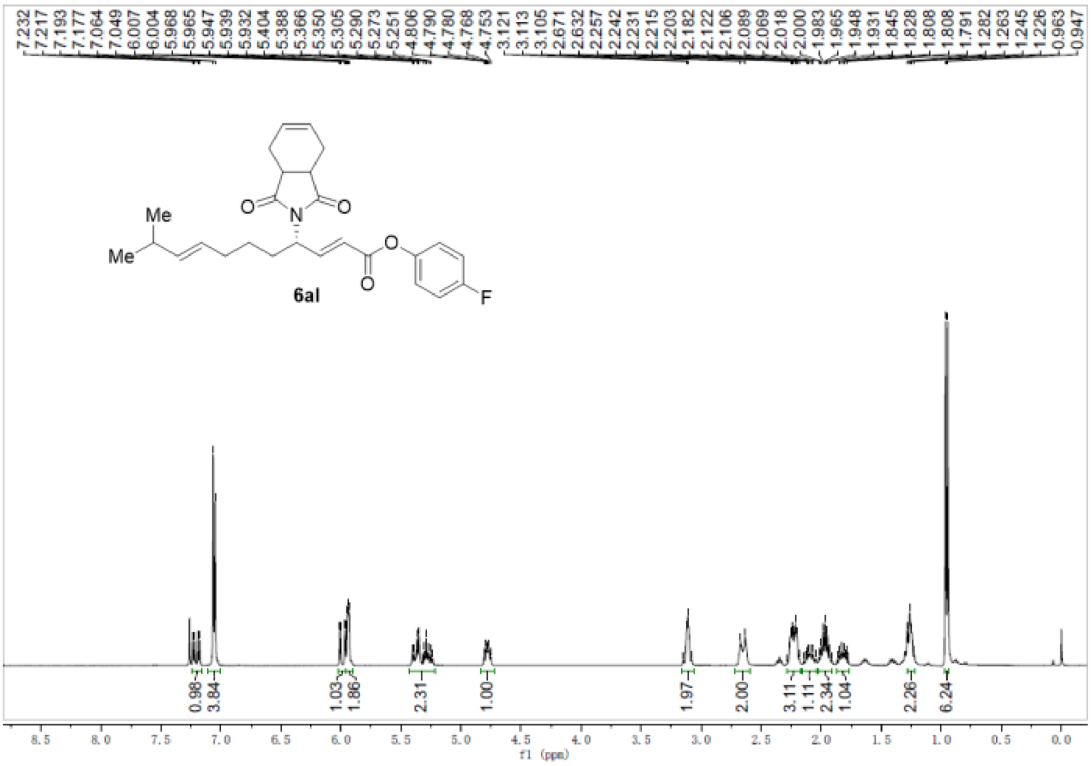
7.232
7.217
7.193
7.177
7.064
7.049
6.007
6.004
5.968
5.965
5.947
5.939
5.932
5.404
5.388
5.366
5.350
5.305
5.290
5.273
5.251
4.806
4.790
4.768
4.753
3.121
3.113
3.105
2.671
2.632
2.257
2.242
2.231
2.215
2.203
2.182
2.106
2.089
2.069
2.018
2.000
1.983
1.965
1.948
1.931
1.845
1.828
1.808
1.808
1.791
1.282
1.263
1.245
1.226
0.963
0.947
Me
Me
O
N
O
O
O
F
6al
0.98
3.84
1.03
1.86
2.31
1.00
1.97
2.00
3.11
1.11
2.34
1.04
2.26
6.24
f1 (ppm)

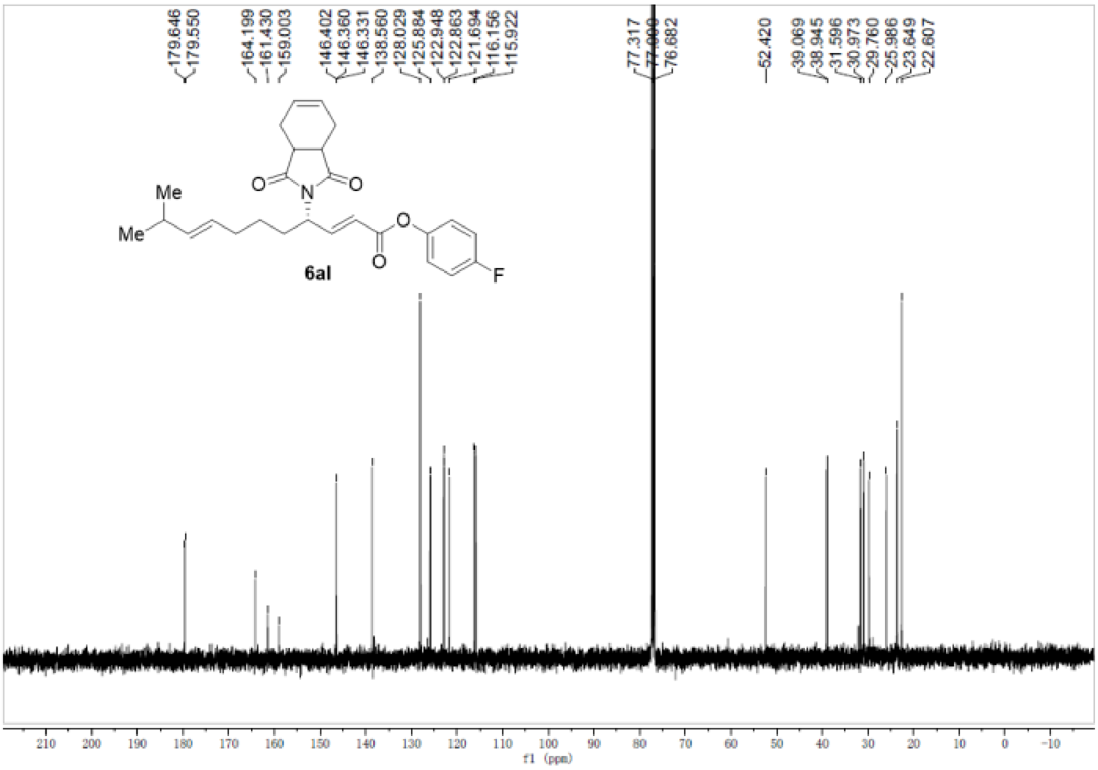
179.646
179.550
164.199
161.430
159.003
146.402
146.360
146.331
138.560
128.029
125.884
122.948
122.863
121.694
116.156
115.922
77.317
77.000
76.682
52.420
39.069
38.945
31.596
30.973
29.760
25.986
23.649
22.607
Me
Me
O
N
O
O
O
F
6al
f1 (ppm)

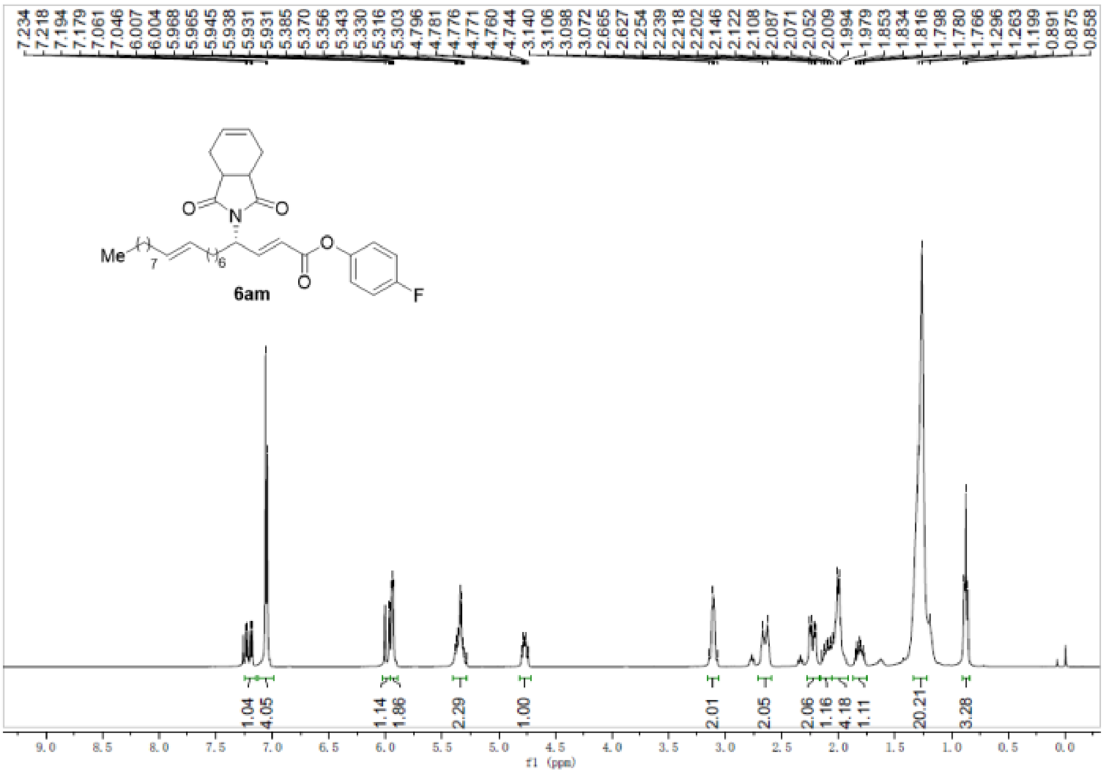

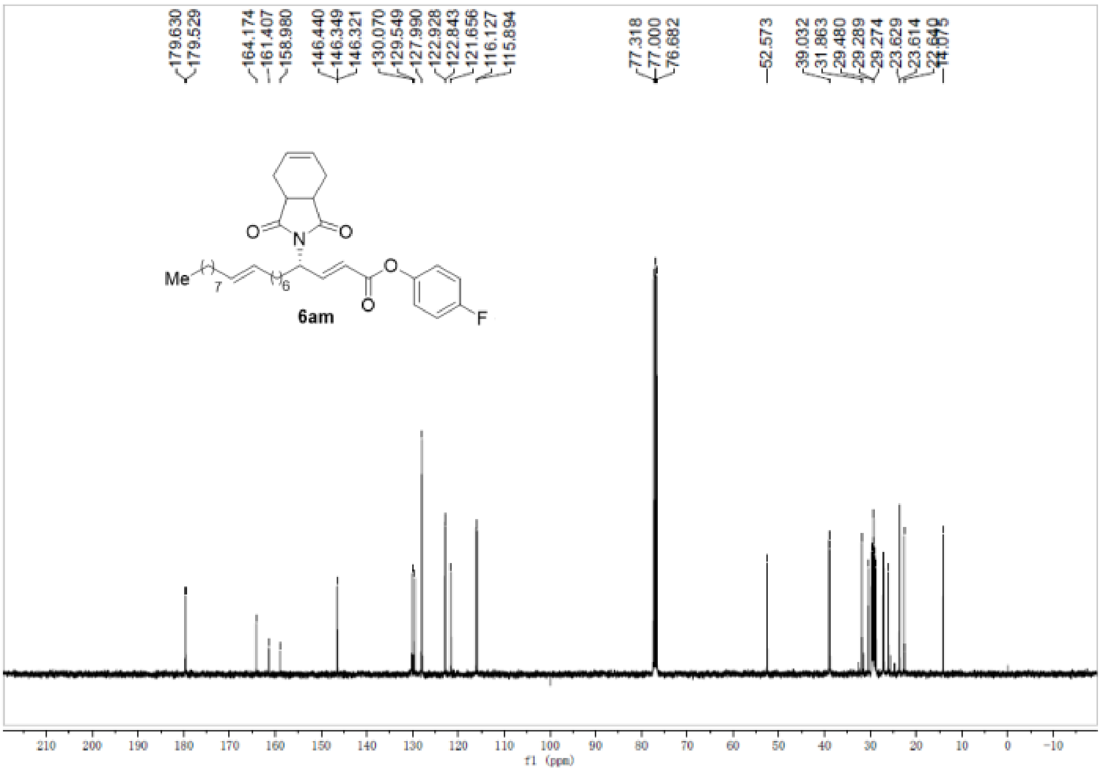

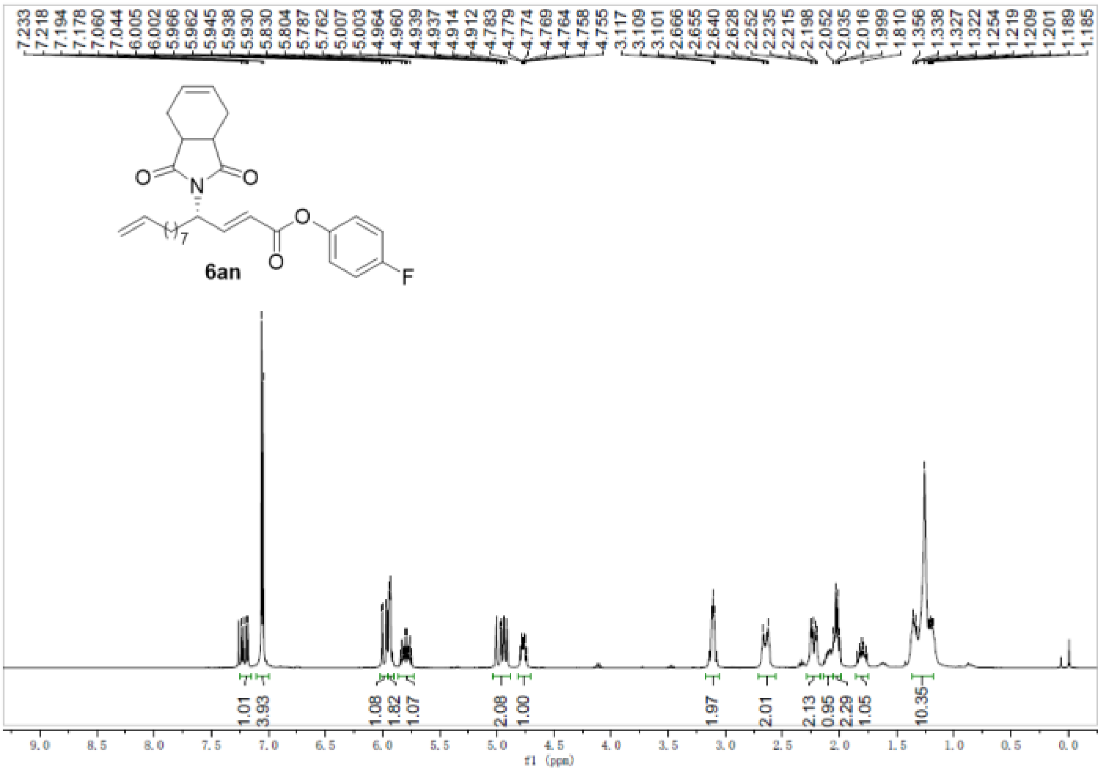
6an
O
N
O
O
O
F
7
7.233
7.218
7.194
7.178
7.060
7.044
6.005
6.002
5.966
5.962
5.945
5.938
5.930
5.830
5.804
5.787
5.762
5.007
5.003
4.964
4.960
4.939
4.937
4.914
4.912
4.783
4.779
4.774
4.769
4.764
4.758
4.755
3.117
3.109
3.101
2.666
2.655
2.640
2.628
2.252
2.235
2.215
2.198
2.052
2.035
2.016
1.999
1.810
1.356
1.338
1.327
1.322
1.254
1.219
1.209
1.201
1.189
1.185
1.01
3.93
1.08
1.82
1.07
2.08
1.00
1.97
2.01
2.13
0.95
2.29
1.05
10.35
f1 (ppm)

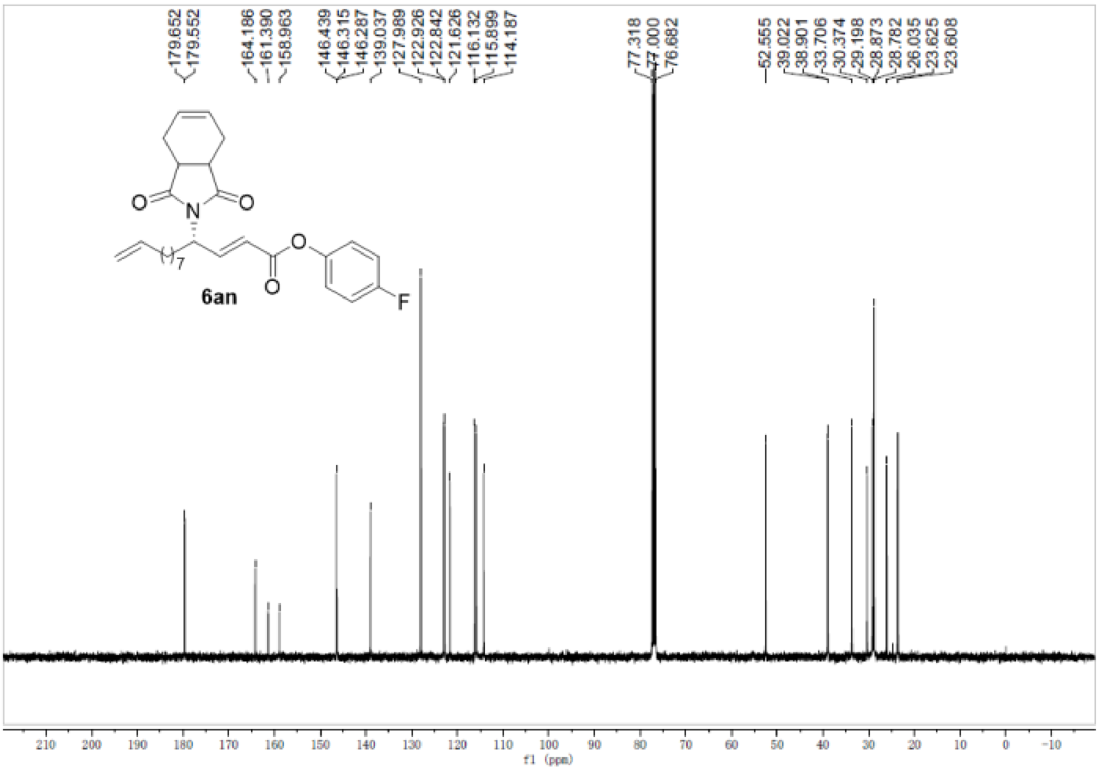
6an
O
N
O
O
O
F
7
179.652
179.552
164.186
161.390
158.963
146.439
146.315
146.287
139.037
127.989
122.926
122.842
121.626
116.132
115.899
114.187
77.318
77.000
76.682
52.555
39.022
38.901
33.706
30.374
29.198
28.873
28.782
26.035
23.625
23.608
f1 (ppm)

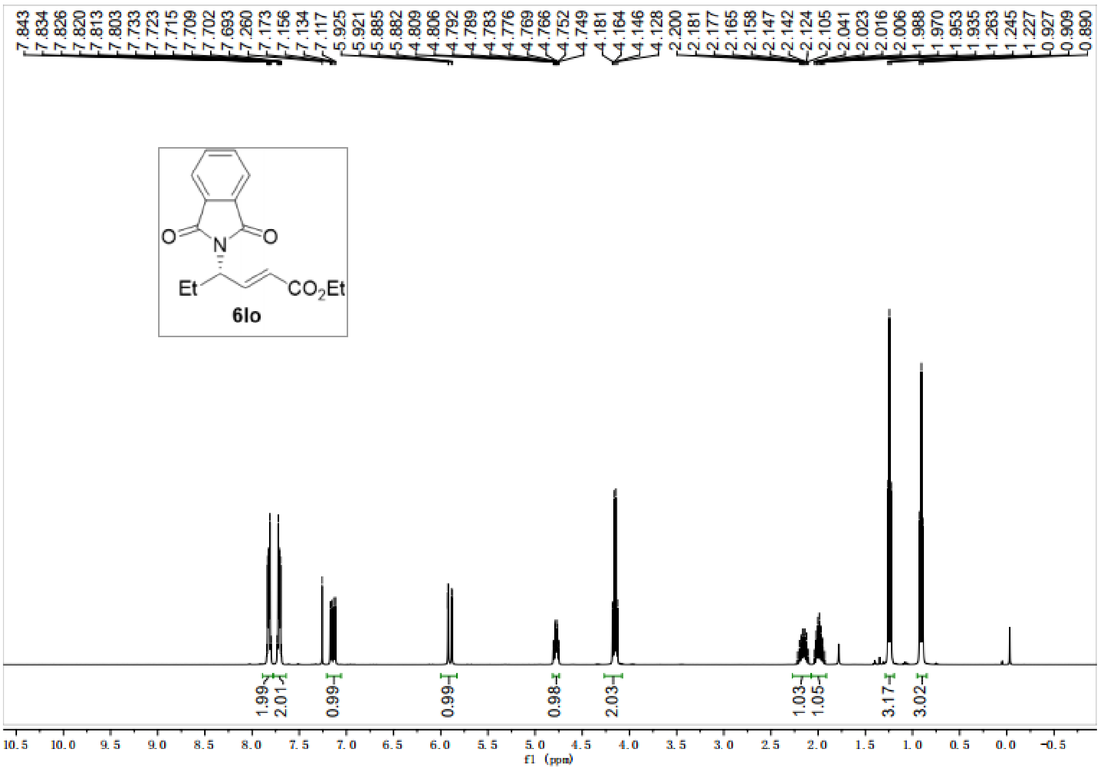
7.843
7.834
7.826
7.820
7.813
7.803
7.733
7.723
7.715
7.709
7.702
7.693
7.260
7.173
7.156
7.134
7.117
5.925
5.921
5.885
5.882
4.809
4.806
4.792
4.789
4.783
4.776
4.769
4.766
4.752
4.749
4.181
4.164
4.146
4.128
2.200
2.181
2.177
2.165
2.158
2.147
2.142
2.124
2.105
2.041
2.023
2.016
2.006
1.988
1.970
1.953
1.935
1.263
1.245
1.227
0.927
0.909
0.890
Et
CO2Et
O
N
O
6lo
1.99
2.01
0.99
0.99
0.98
2.03
1.03
1.05
3.17
3.02
f1 (ppm)

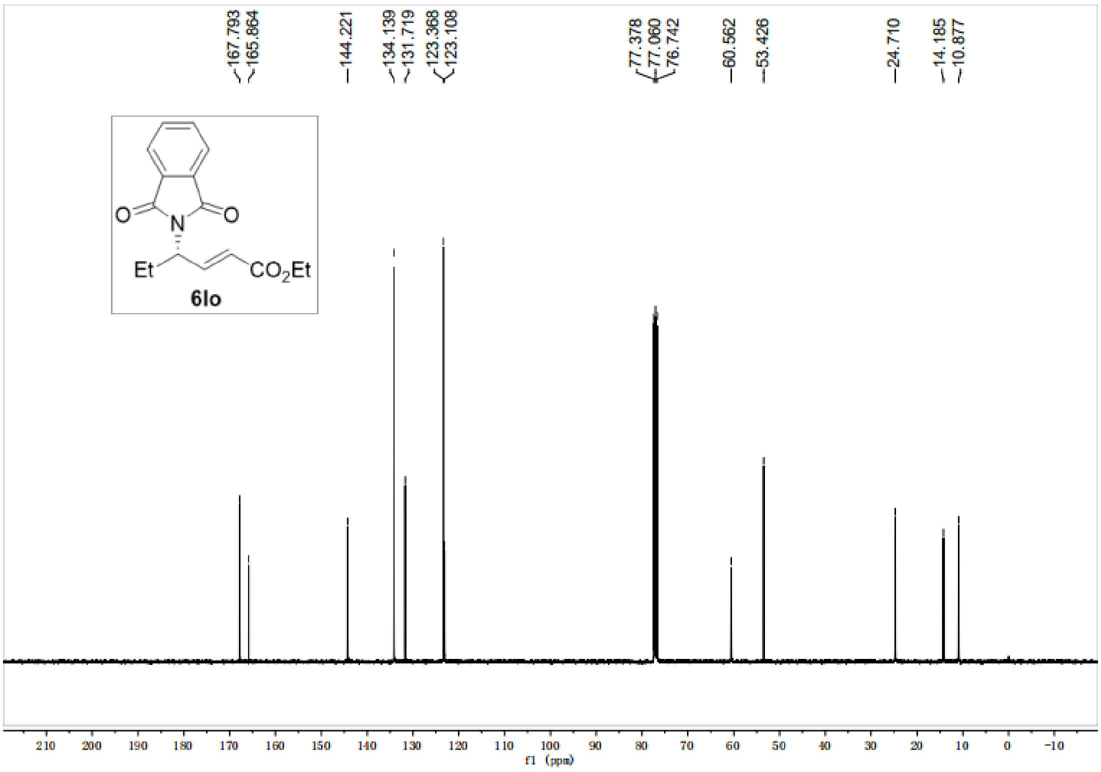
167.793
165.864
144.221
134.139
131.719
123.368
123.108
77.378
77.060
76.742
60.562
53.426
24.710
14.185
10.877
Et
CO2Et
O
N
O
6lo
f1 (ppm)

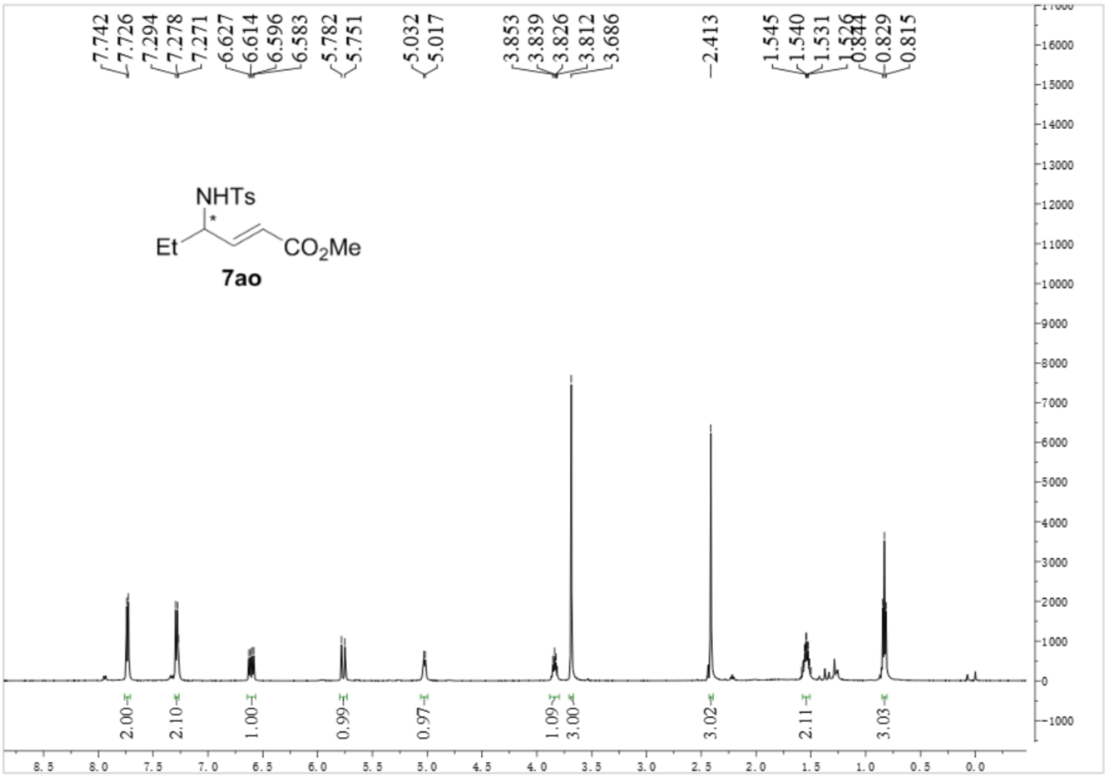
7.742
7.726
7.294
7.278
7.271
6.627
6.614
6.596
6.583
5.782
5.751
5.032
5.017
3.853
3.839
3.826
3.812
3.686
2.413
1.545
1.540
1.531
1.526
0.844
0.829
0.815
NHTs
Et
CO2Me
7ao
2.00
2.10
1.00
0.99
0.97
1.09
3.00
3.02
2.11
3.03

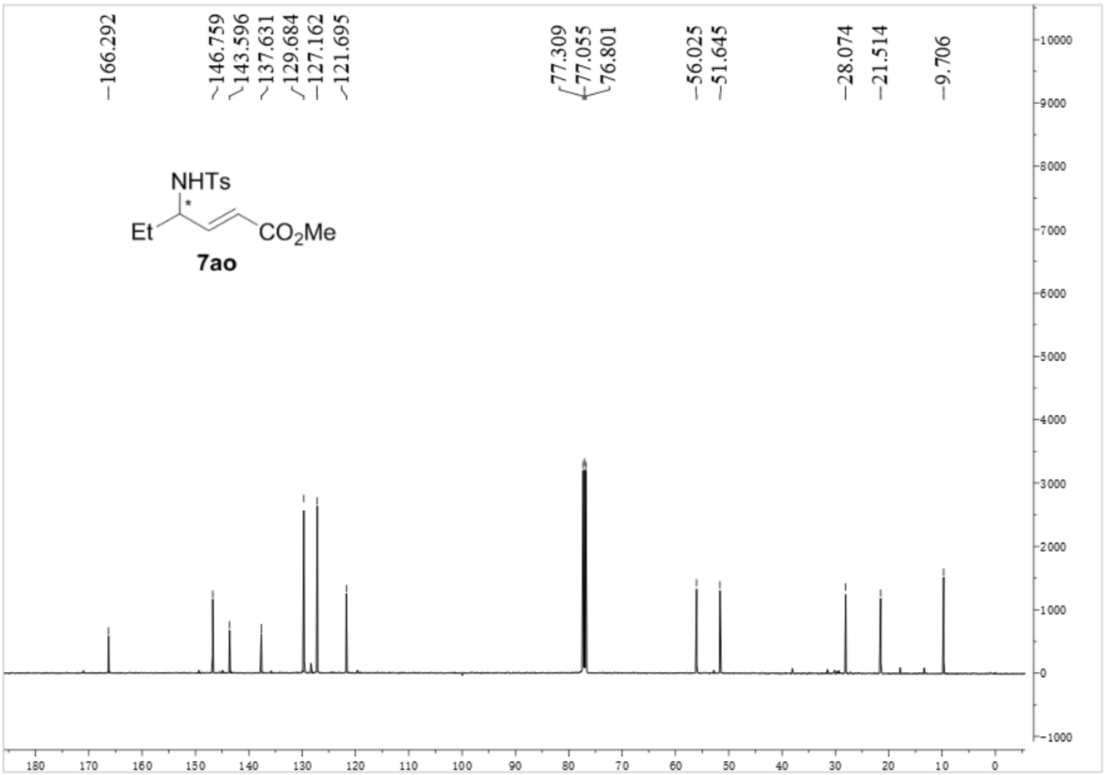
166.292
146.759
143.596
137.631
129.684
127.162
121.695
77.309
77.055
76.801
56.025
51.645
28.074
21.514
9.706
NHTs
Et
CO2Me
7ao

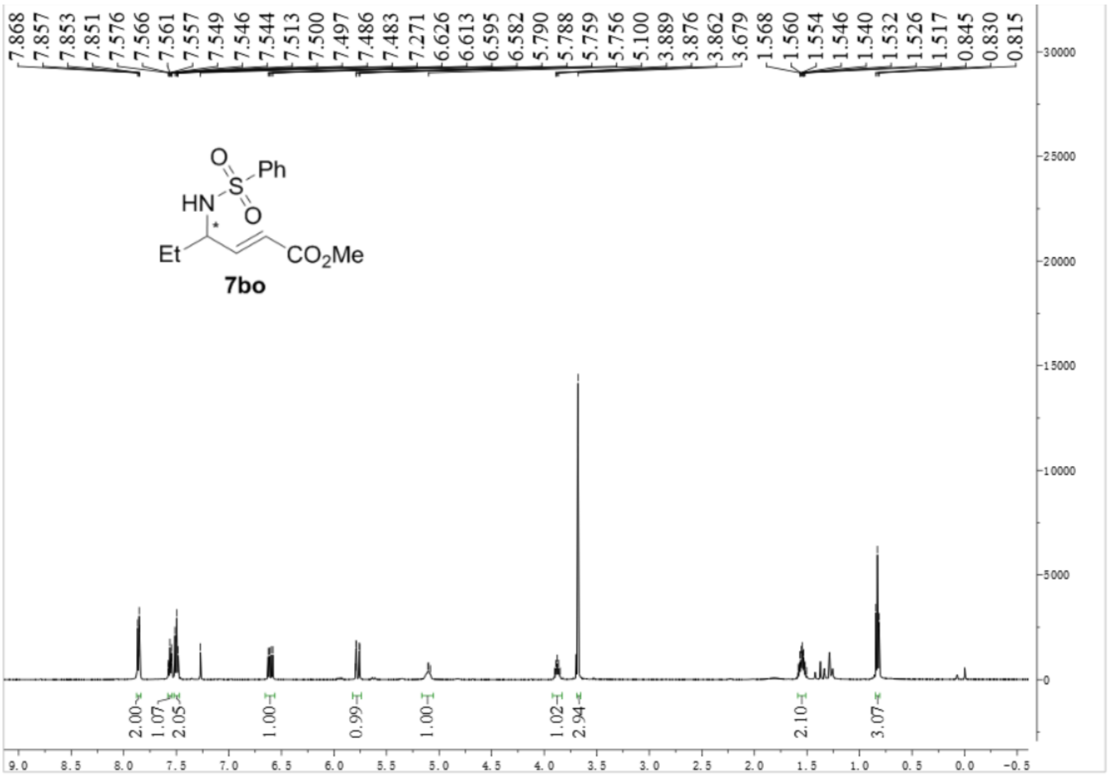
7.868
7.857
7.853
7.851
7.576
7.566
7.561
7.557
7.549
7.546
7.544
7.513
7.500
7.497
7.486
7.483
7.271
6.626
6.613
6.595
6.582
5.790
5.788
5.759
5.756
5.100
3.889
3.876
3.862
3.679
1.568
1.560
1.554
1.546
1.540
1.532
1.526
1.517
0.845
0.830
0.815
Ph
HN
Et
CO_2Me
7bo
2.00
1.07
2.05
1.00
0.99
1.00
1.02
2.94
2.10
3.07

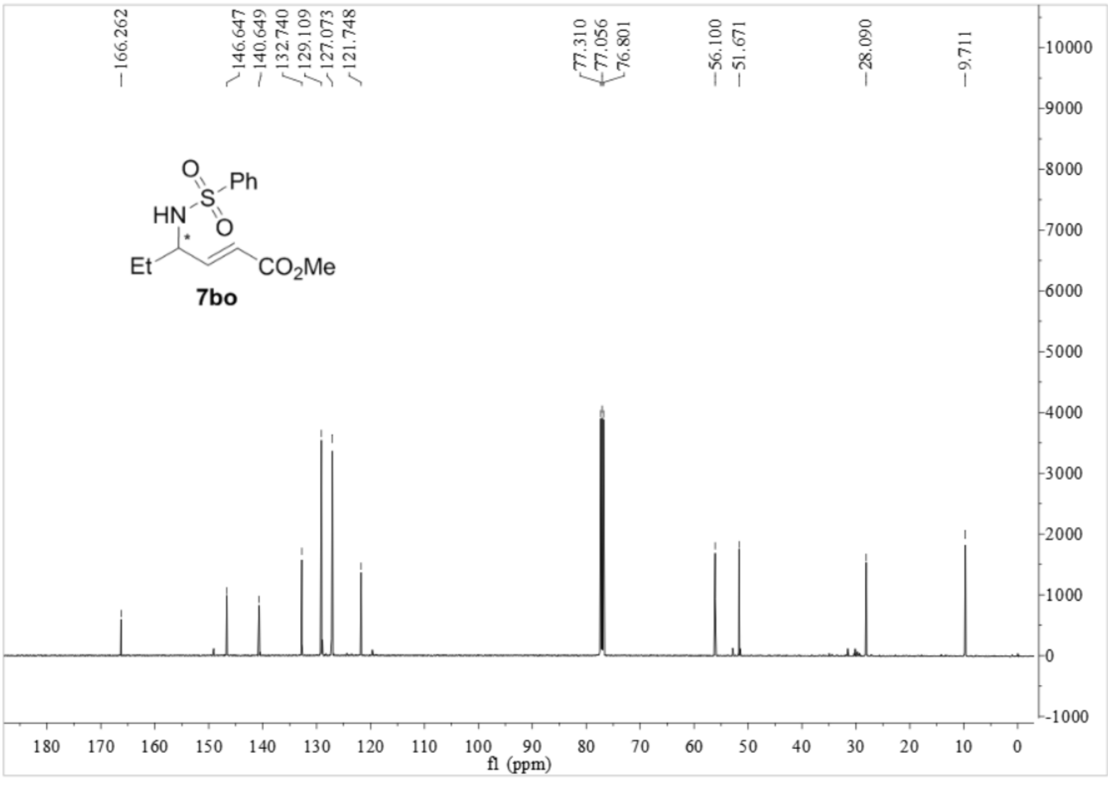
166.262
146.647
140.649
132.740
129.109
127.073
121.748
77.310
77.056
76.801
56.100
51.671
28.090
9.711
Ph
HN
Et
CO_2Me
7bo
f1 (ppm)

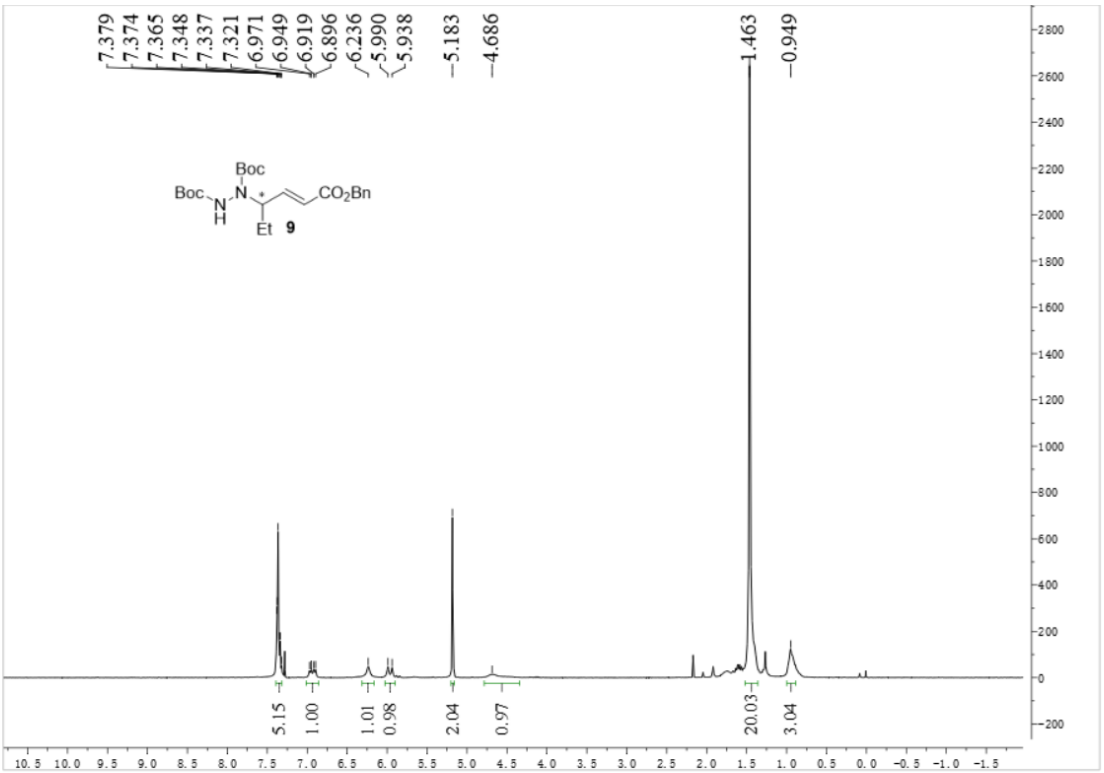

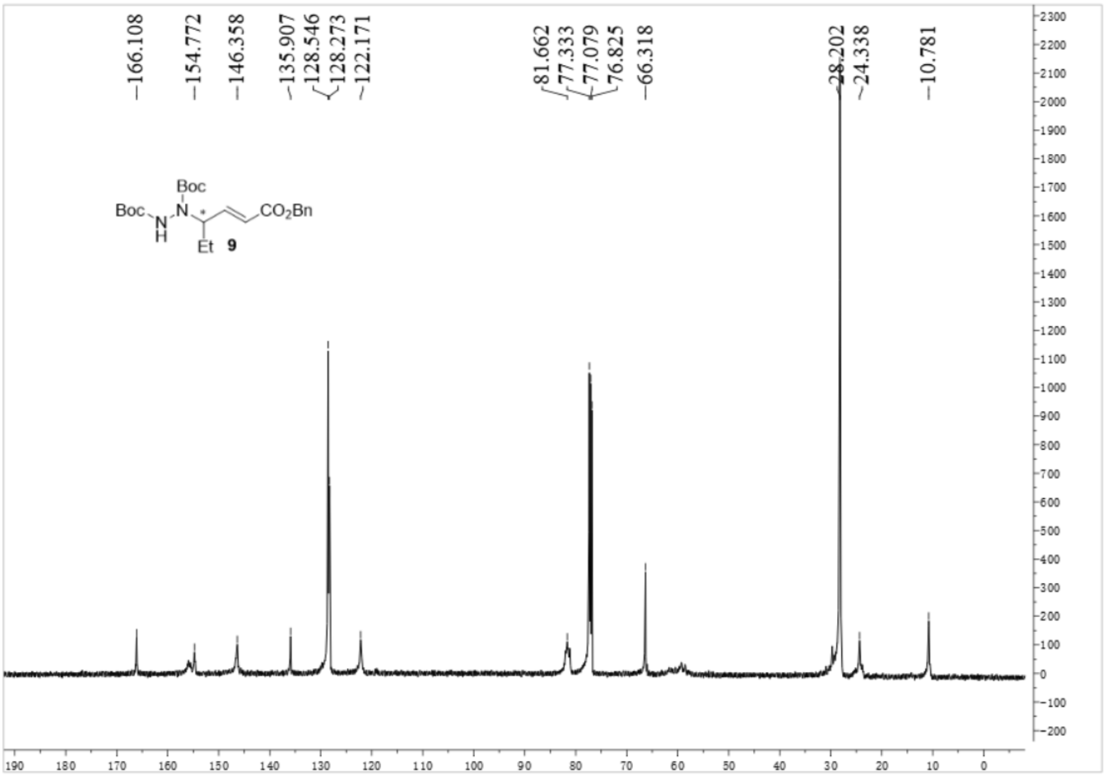

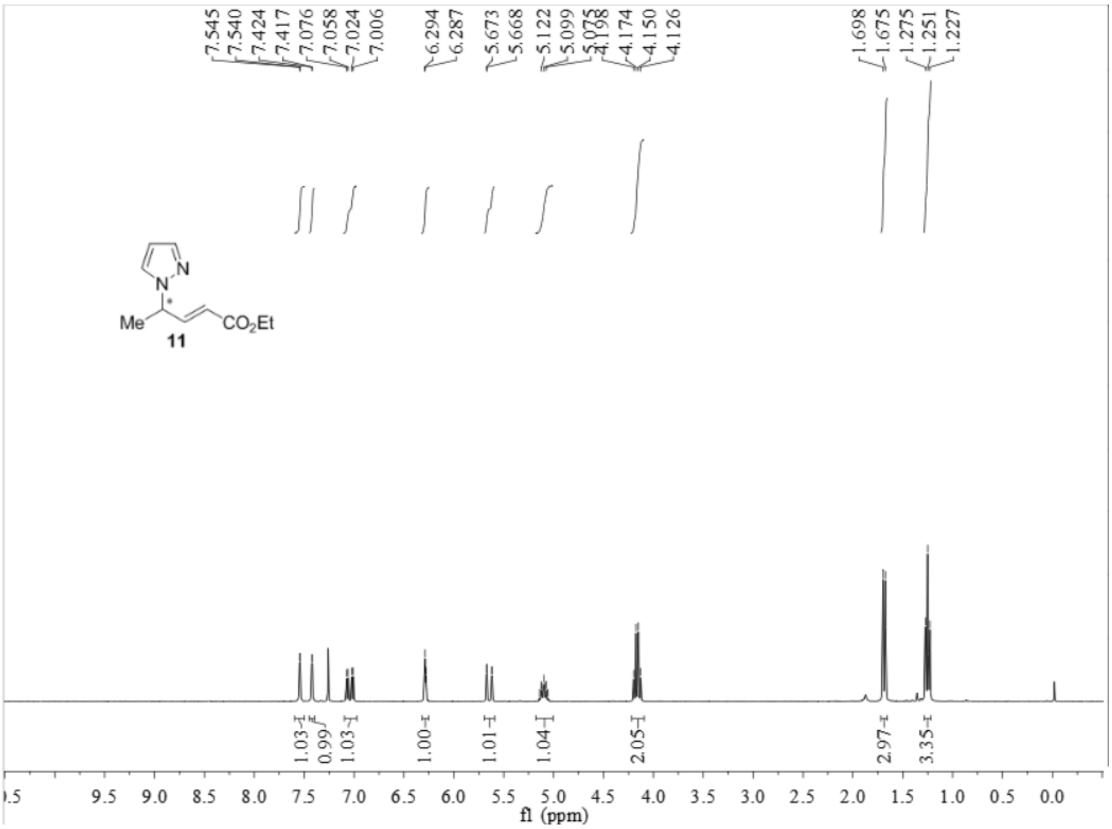
7.545
7.540
7.424
7.417
7.076
7.058
7.024
7.006
6.294
6.287
5.673
5.668
5.122
5.099
5.075
4.198
4.174
4.150
4.126
1.698
1.675
1.275
1.251
1.227
N
N
Me
CO2Et
11
1.03
0.99
1.03
1.00
1.01
1.04
2.05
2.97
3.35
9.5
9.0
8.5
8.0
7.5
7.0
6.5
6.0
5.5
5.0
4.5
4.0
3.5
3.0
2.5
2.0
1.5
1.0
0.5
0.0
f1 (ppm)

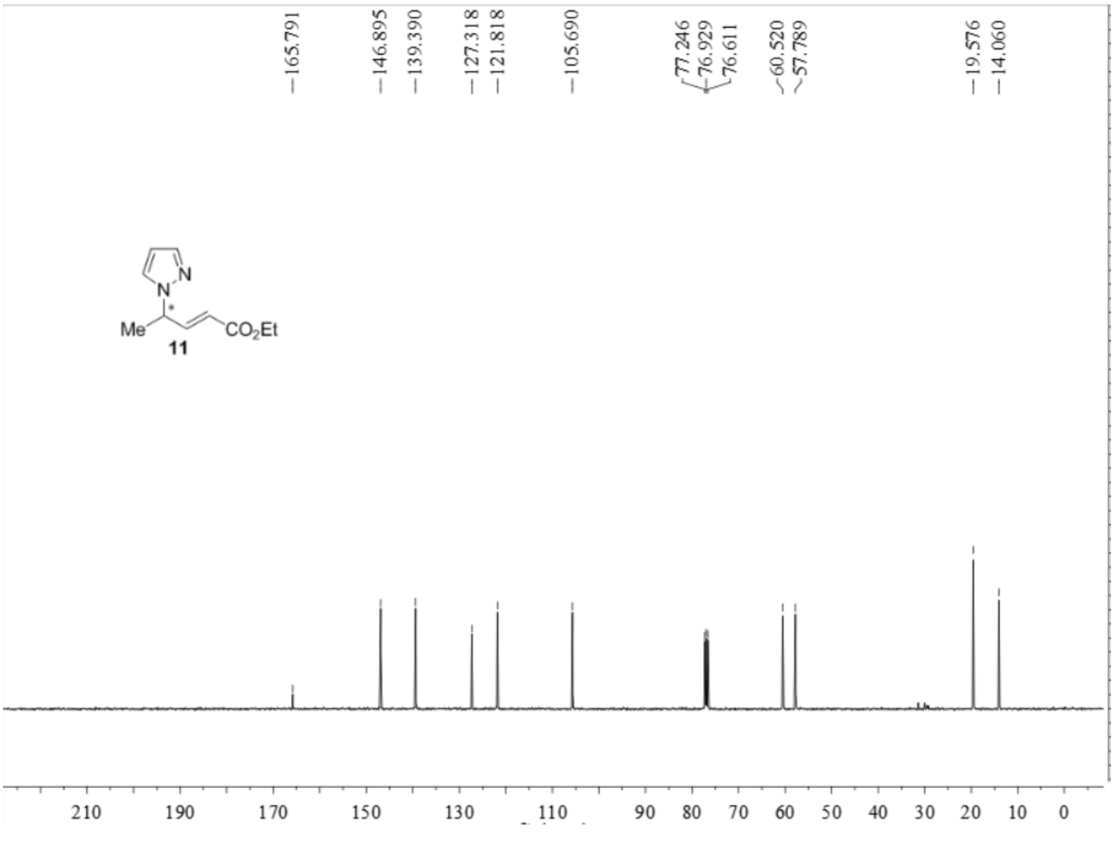
165.791
146.895
139.390
127.318
121.818
105.690
77.246
76.929
76.611
60.520
57.789
19.576
14.060
N
N
Me
CO2Et
11
210
190
170
150
130
110
90
80
70
60
50
40
30
20
10
0

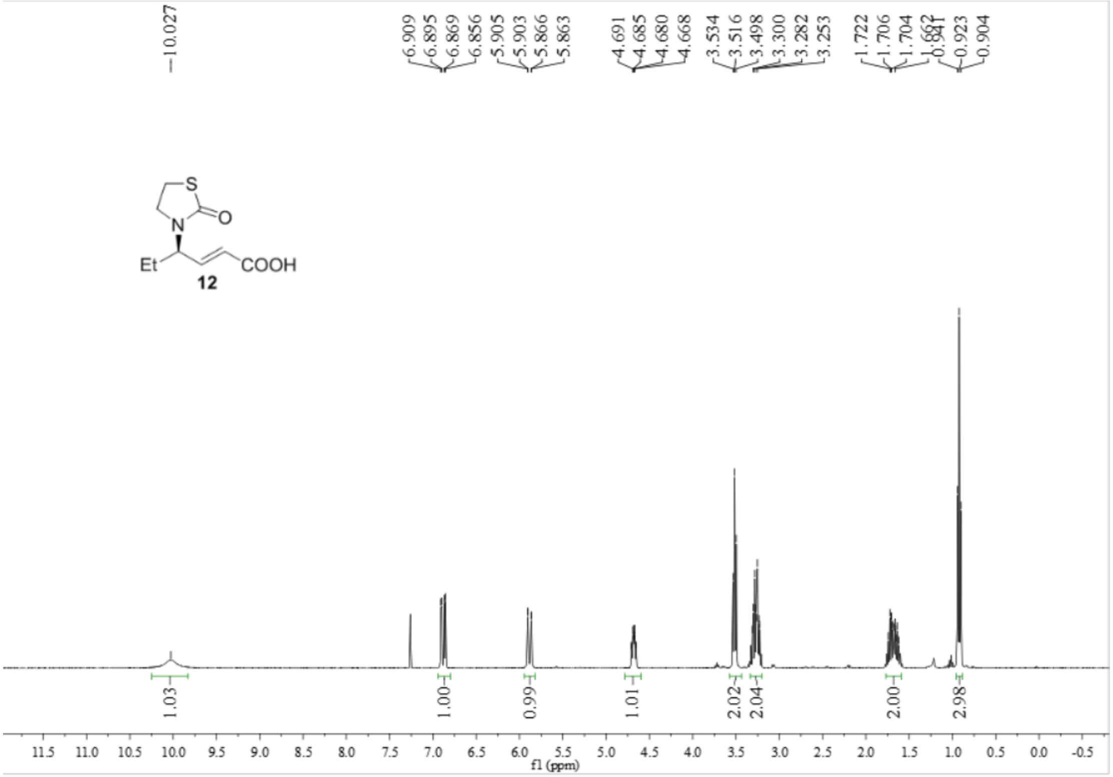
10.027
6.909
6.895
6.869
6.856
5.905
5.903
5.866
5.863
4.691
4.685
4.680
4.668
3.534
3.516
3.498
3.300
3.282
3.253
1.722
1.706
1.704
0.923
0.904
Et
N
S
O
COOH
12
1.03
1.00
0.99
1.01
2.02
2.04
2.00
2.98
f1 (ppm)

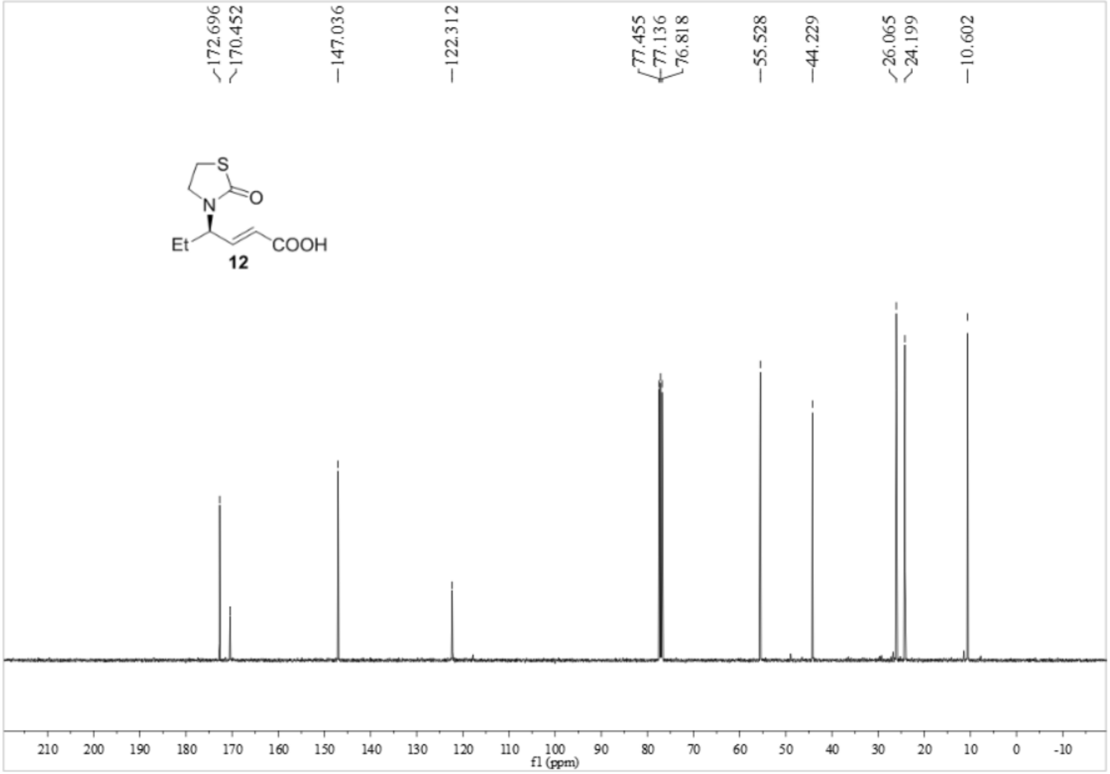
172.696
170.452
147.036
122.312
77.455
77.136
76.818
55.528
44.229
26.065
24.199
10.602
Et
N
S
O
COOH
12
f1 (ppm)

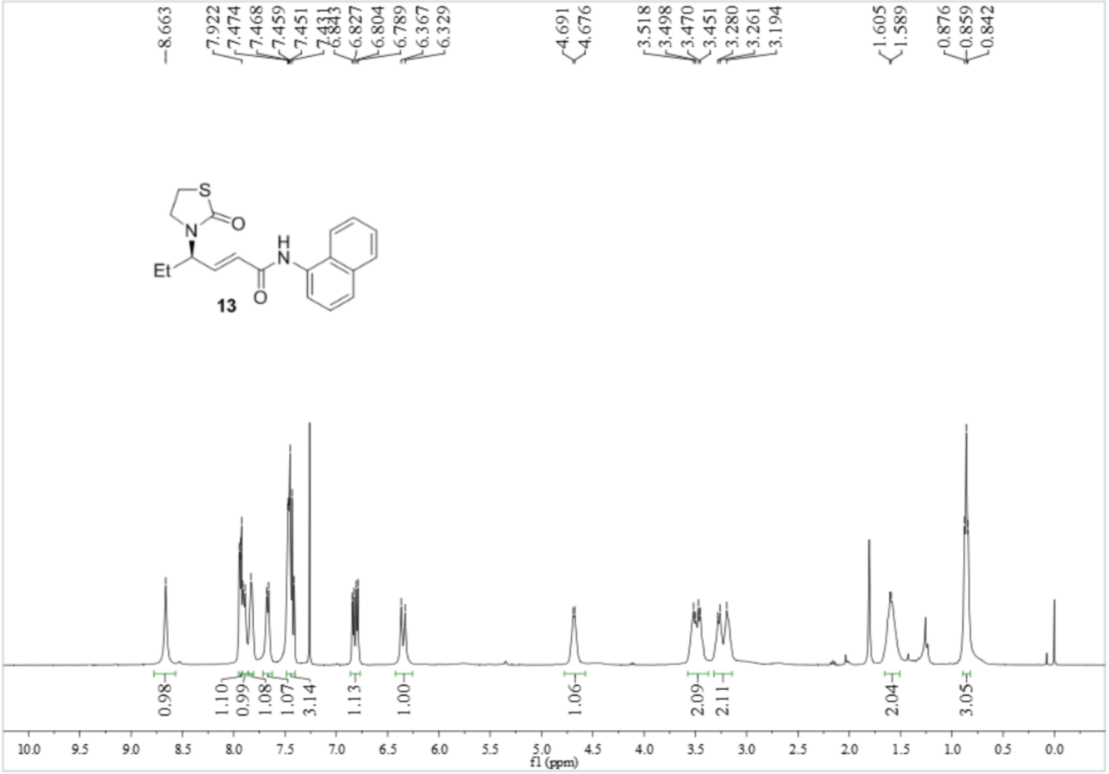

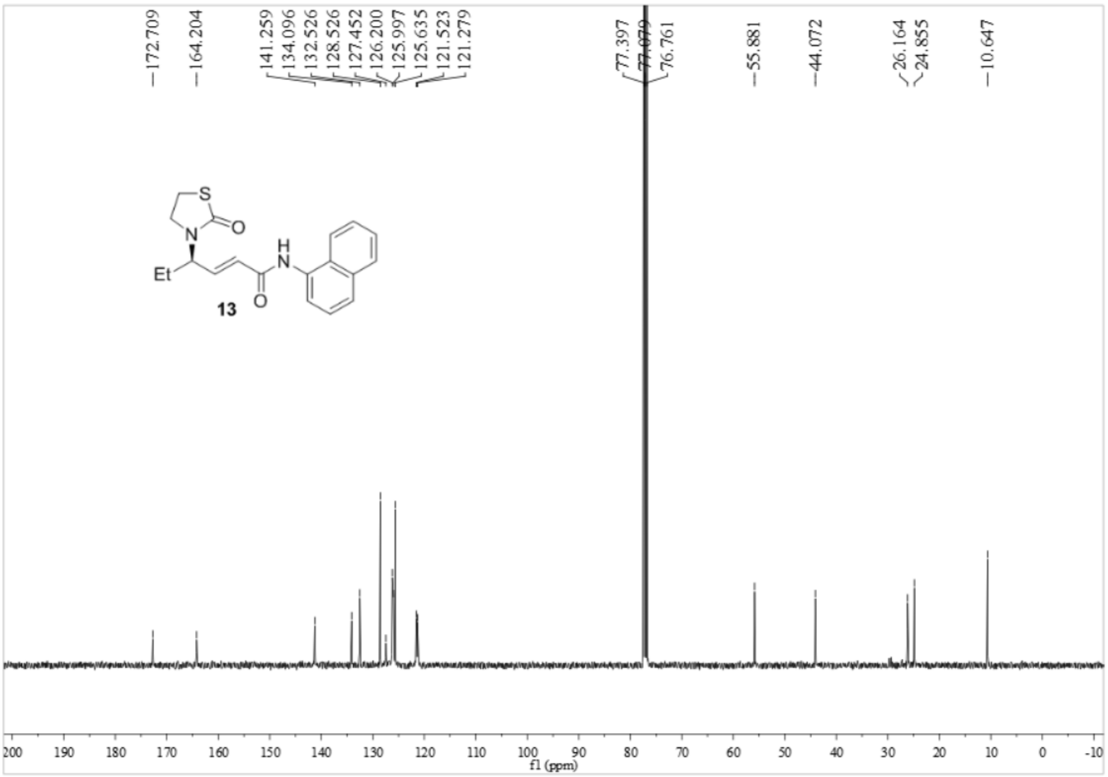

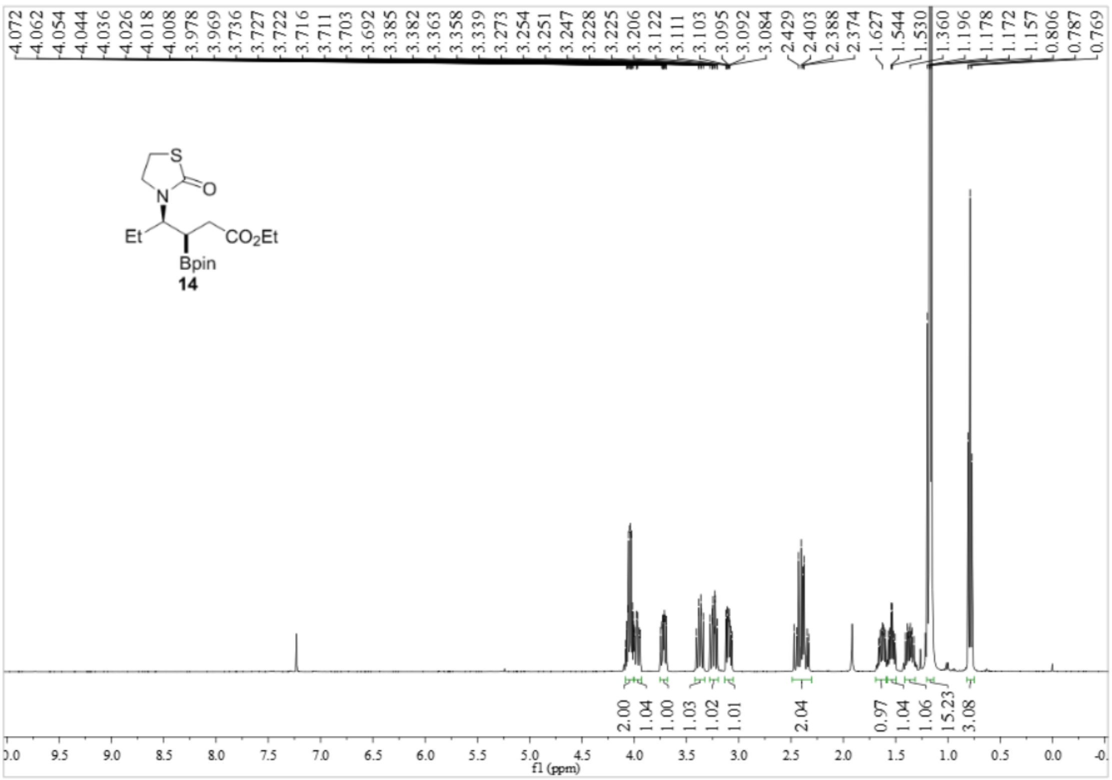
4.072 4.062 4.054 4.044 4.036 4.026 4.018 4.008 3.978 3.969 3.736 3.727 3.722 3.716 3.711 3.703 3.692 3.385 3.382 3.363 3.358 3.339 3.273 3.254 3.251 3.247 3.228 3.225 3.206 3.122 3.111 3.103 3.095 3.092 3.084 2.429 2.403 2.388 2.374 1.627 1.544 1.530 1.360 1.196 1.178 1.172 1.157 0.806 0.787 0.769
S
N
O
Et
CO2Et
Bpin
14
2.00 1.04 1.00 1.03 1.02 1.01 2.04 0.97 1.04 1.06 15.23 3.08
f1 (ppm)

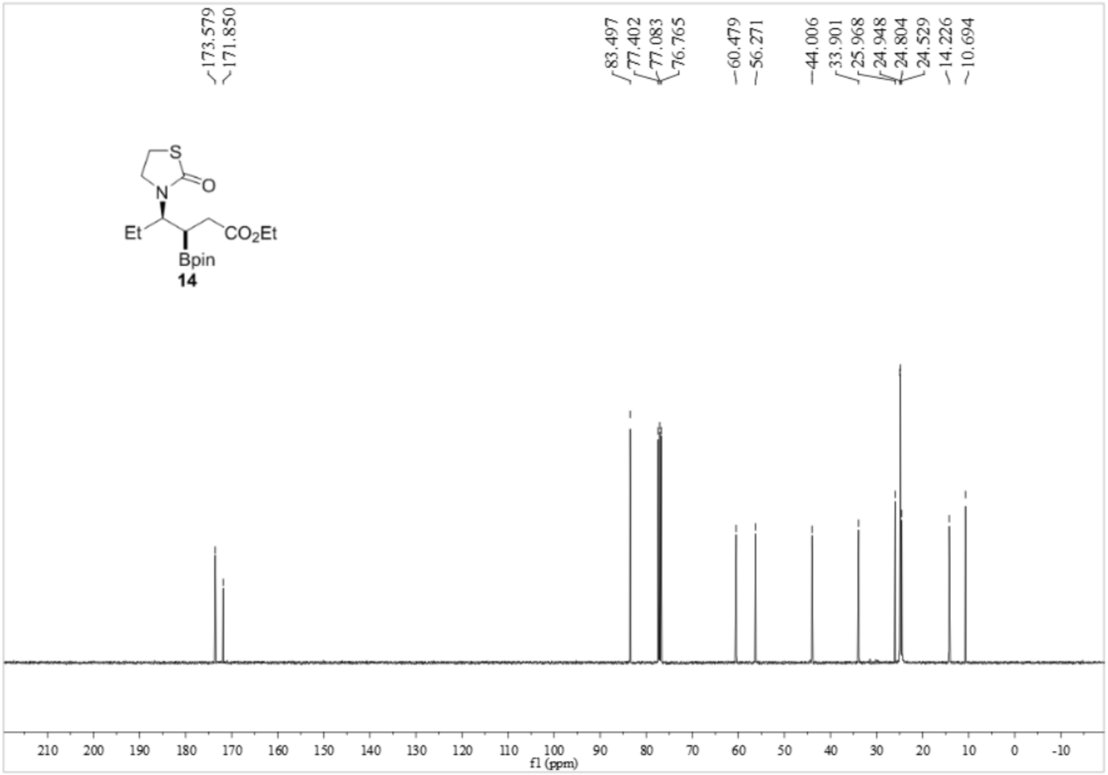
173.579 171.850 83.497 77.402 77.083 76.765 60.479 56.271 44.006 33.901 25.968 24.948 24.804 24.529 14.226 10.694
S
N
O
Et
CO2Et
Bpin
14
f1 (ppm)

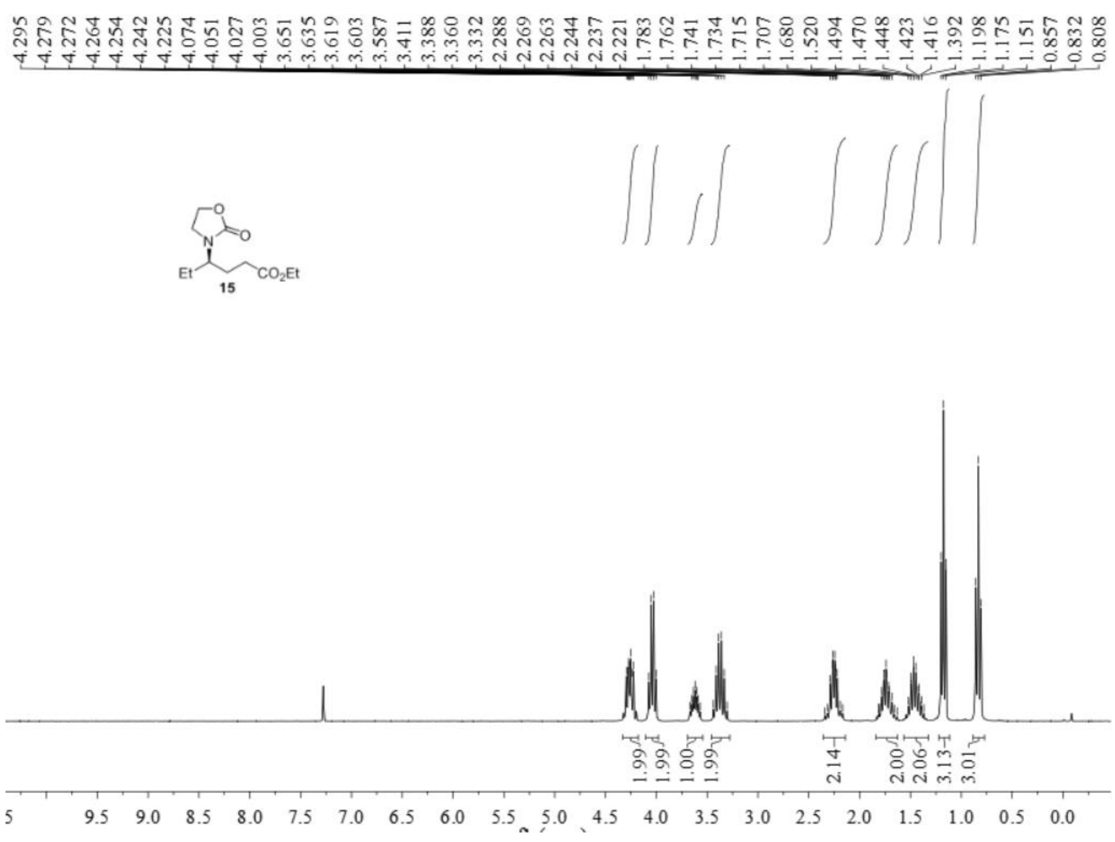

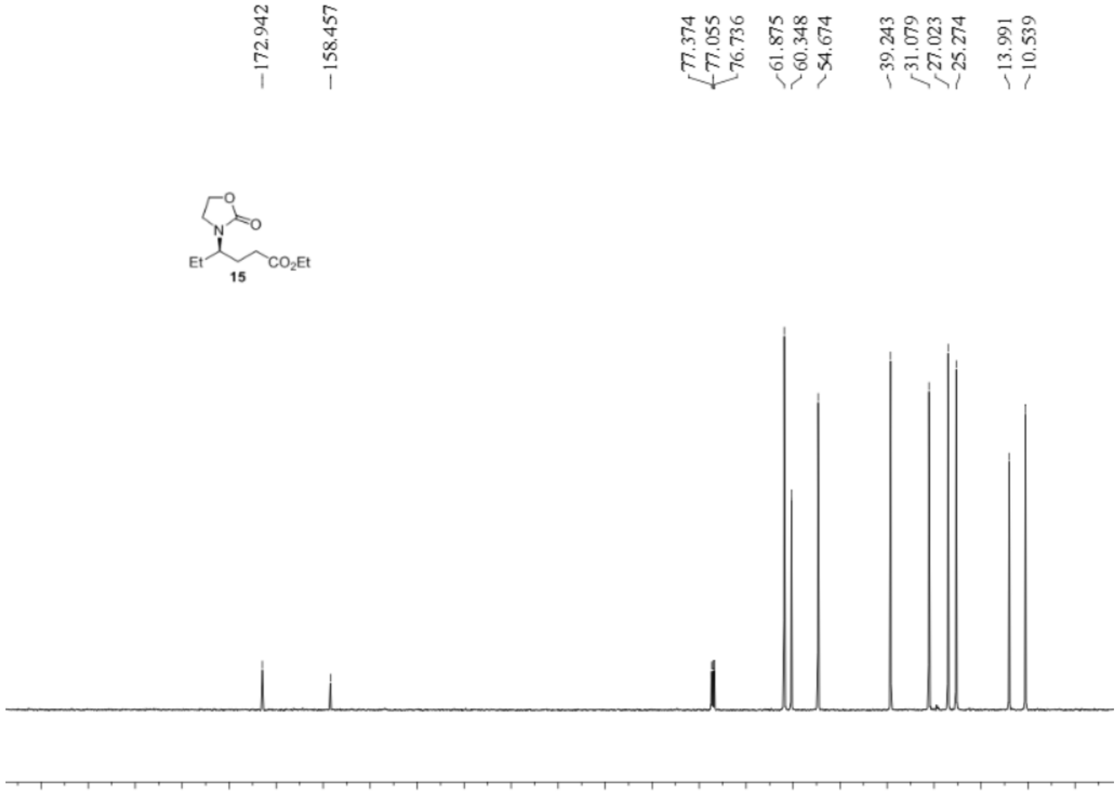

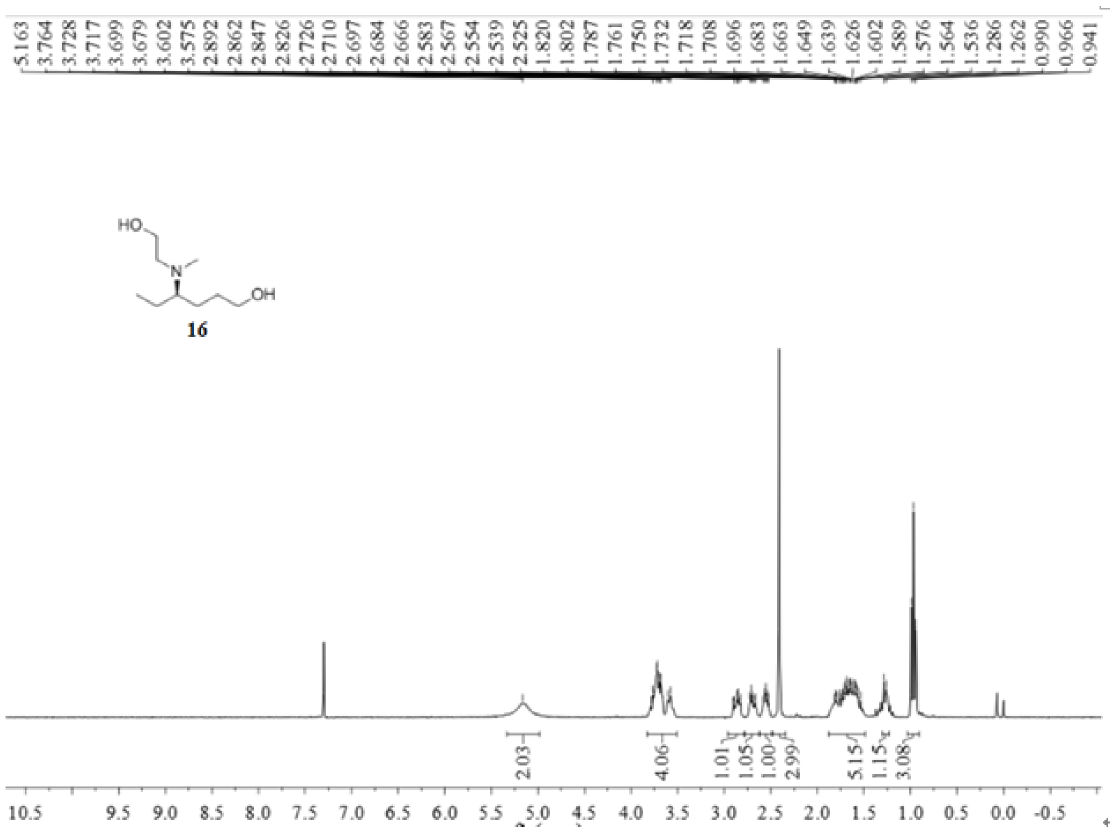

16

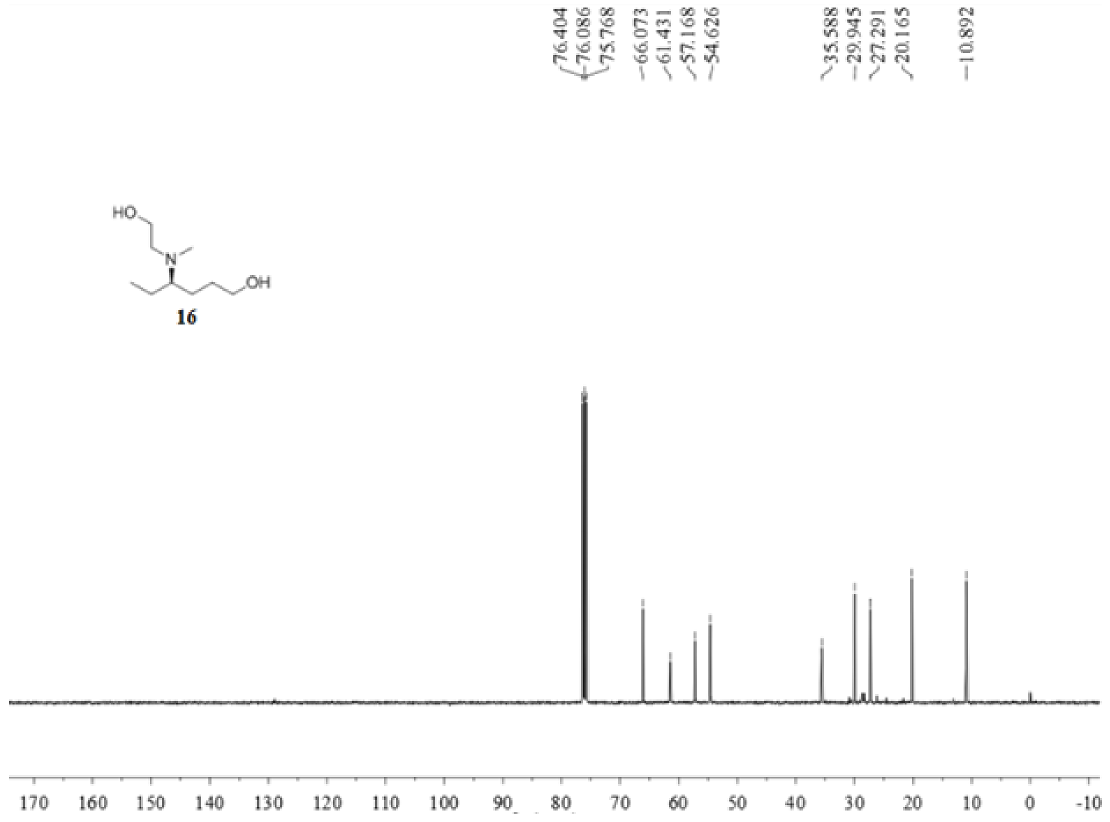

16

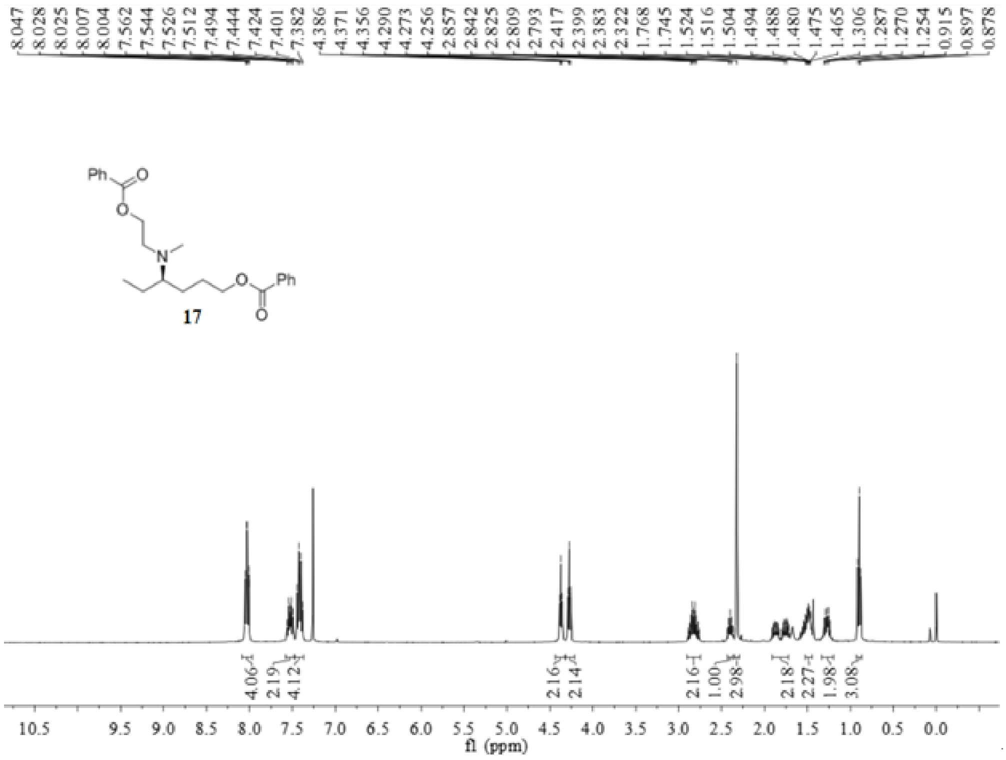
8.047
8.028
8.025
8.007
8.004
7.562
7.544
7.526
7.512
7.494
7.444
7.424
7.401
7.382
4.386
4.371
4.356
4.290
4.273
4.256
2.857
2.842
2.825
2.809
2.793
2.417
2.399
2.383
2.322
1.768
1.745
1.524
1.516
1.504
1.494
1.488
1.480
1.475
1.465
1.306
1.287
1.270
1.254
0.915
0.897
0.878
Ph
O
O
N
O
O
Ph
17
4.06
2.19
4.12
2.16
2.14
2.16
1.00
2.98
2.18
2.27
1.98
3.08
10.5 9.5 9.0 8.5 8.0 7.5 7.0 6.5 6.0 5.5 5.0 4.5 4.0 3.5 3.0 2.5 2.0 1.5 1.0 0.5 0.0
f1 (ppm)

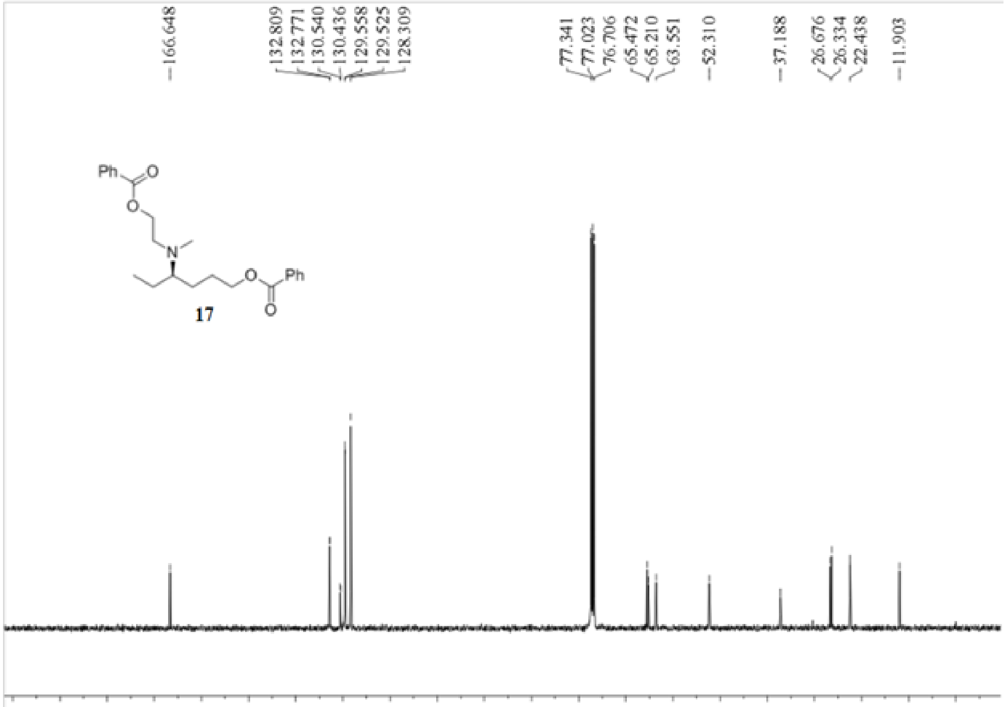
166.648
132.809
132.771
130.540
130.436
129.558
129.525
128.309
77.341
77.023
76.706
65.472
65.210
63.551
52.310
37.188
26.676
26.334
22.438
11.903
Ph
O
O
N
O
O
Ph
17
200 190 180 170 160 150 140 130 120 110 100 90 80 70 60 50 40 30 20 10 0

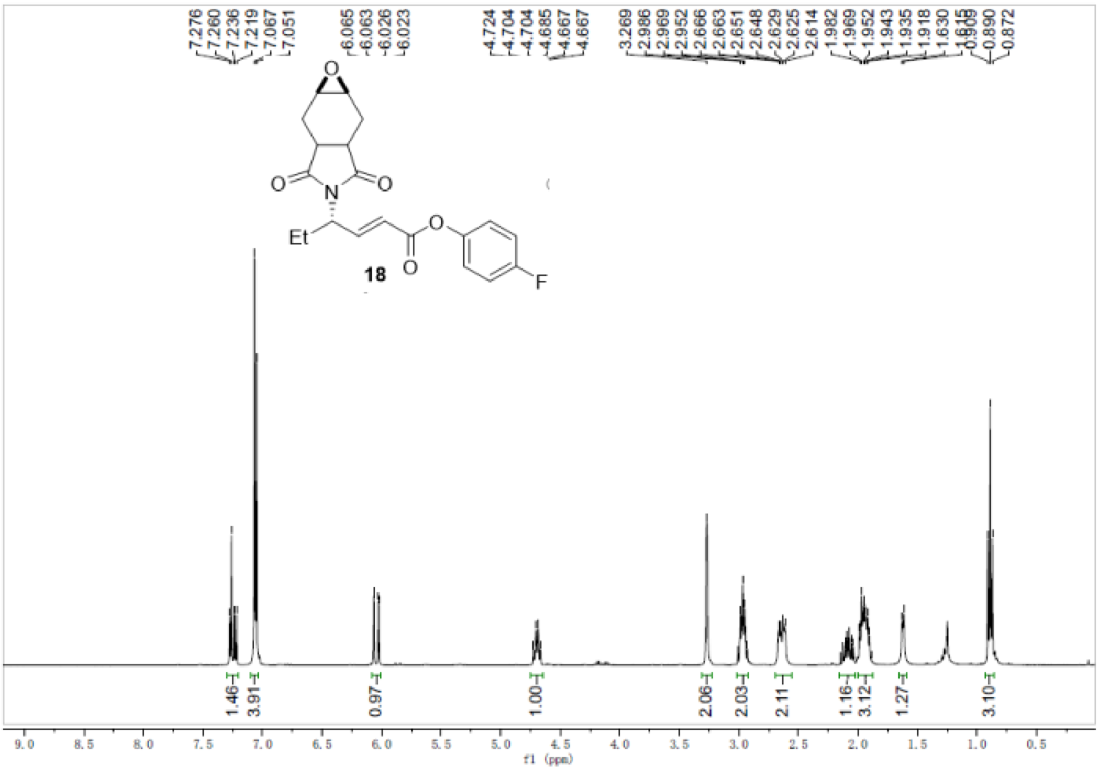

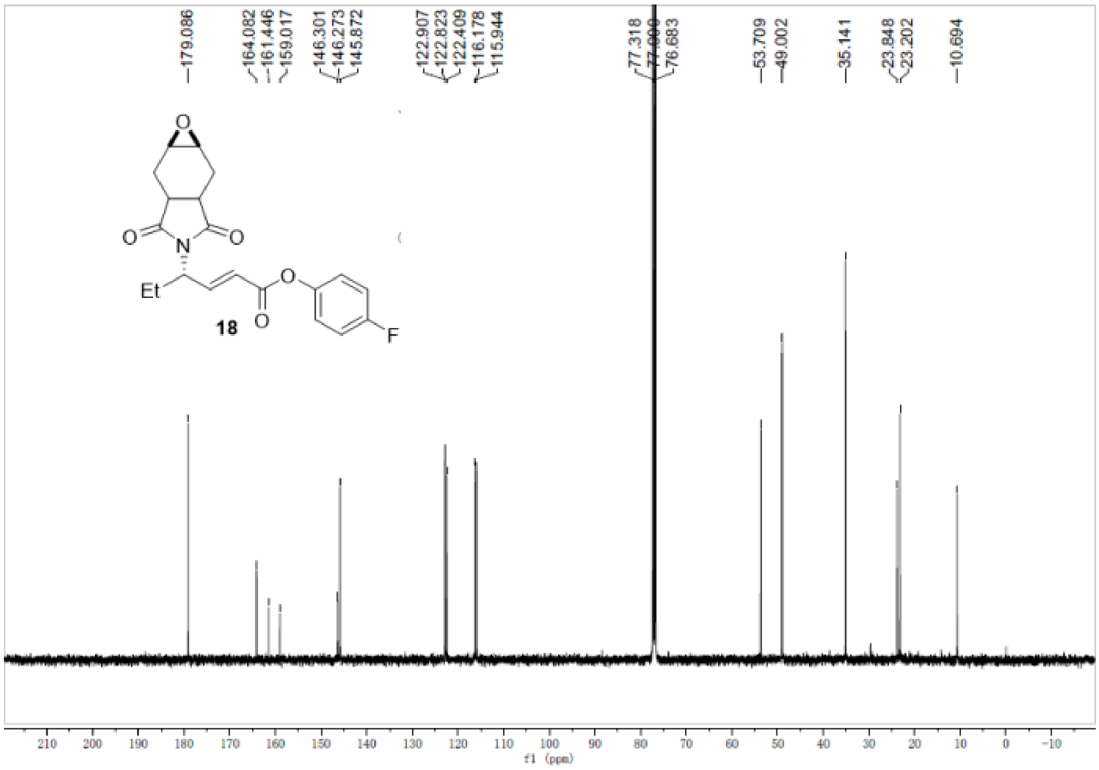

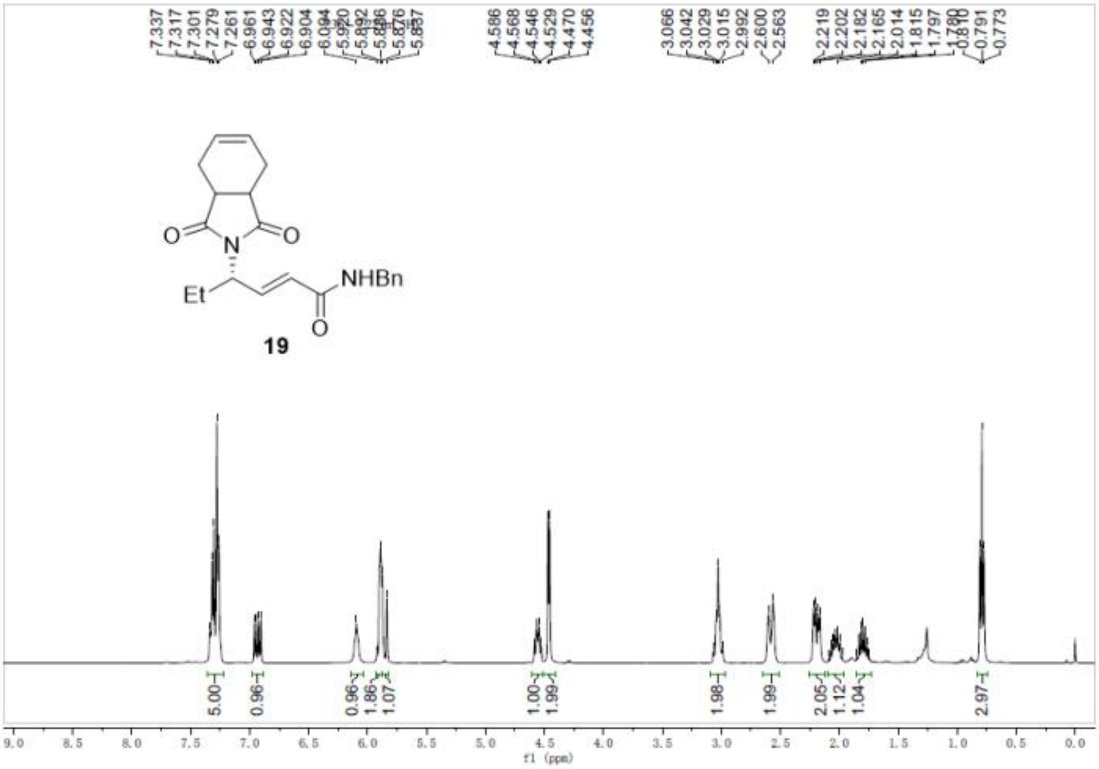
7.337
7.317
7.301
7.279
7.261
6.961
6.943
6.922
6.904
6.094
4.586
4.568
4.546
4.529
4.470
4.456
3.066
3.042
3.029
3.015
2.992
2.600
2.563
2.219
2.202
2.182
2.165
2.014
1.815
1.797
0.791
0.773
O
N
O
NHBn
Et
O
19
5.00
0.96
0.96
1.86
1.07
1.00
1.99
1.98
1.99
2.05
1.12
1.04
2.97
f1 (ppm)

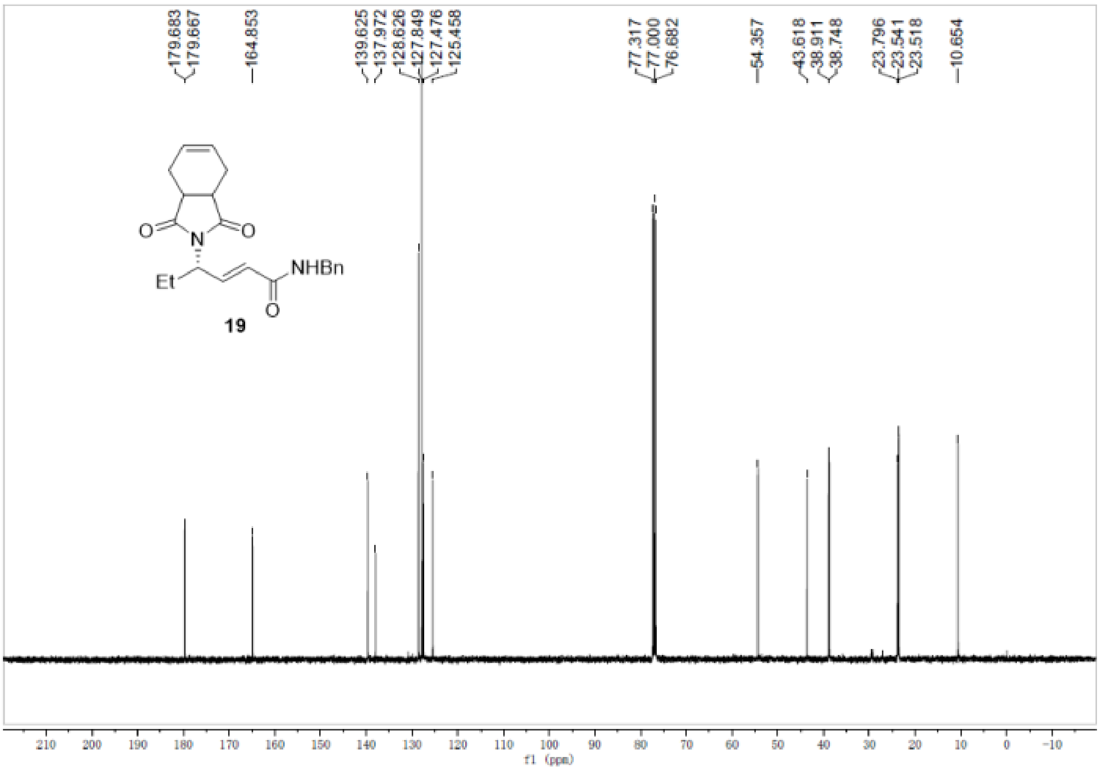
179.683
179.667
164.853
139.625
137.972
128.626
127.849
127.476
125.458
77.317
77.000
76.682
54.357
43.618
38.911
38.748
23.796
23.541
23.518
10.654
O
N
O
NHBn
Et
O
19
f1 (ppm)

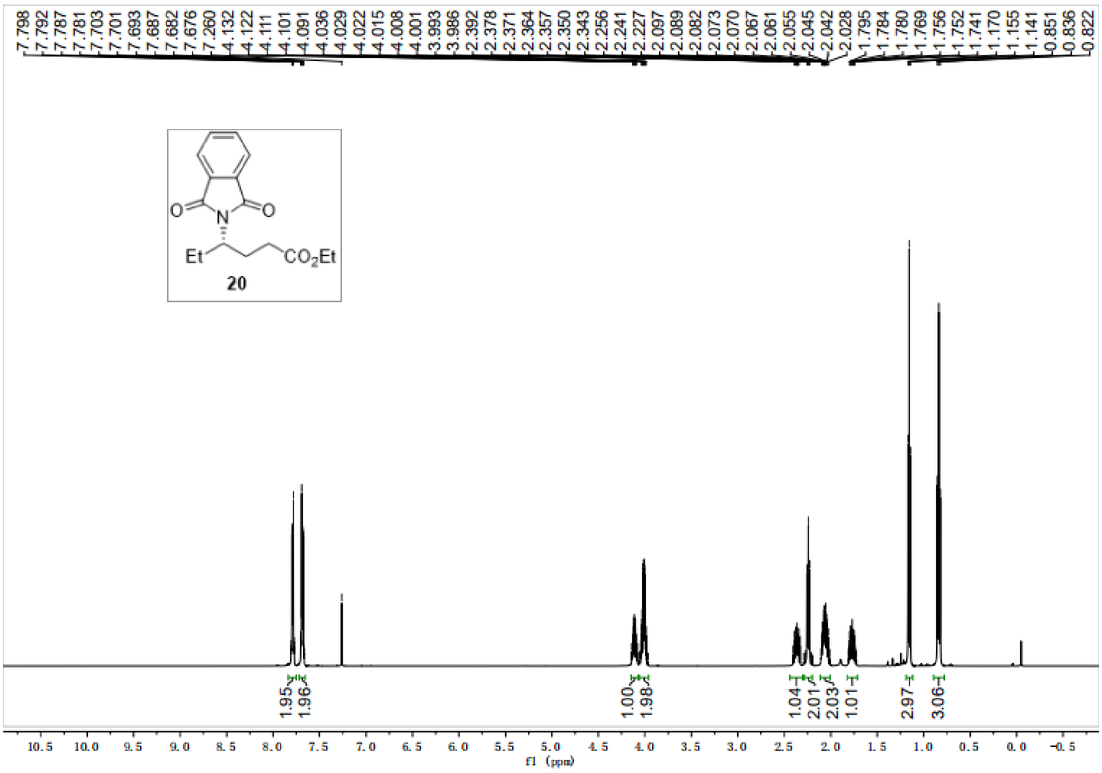

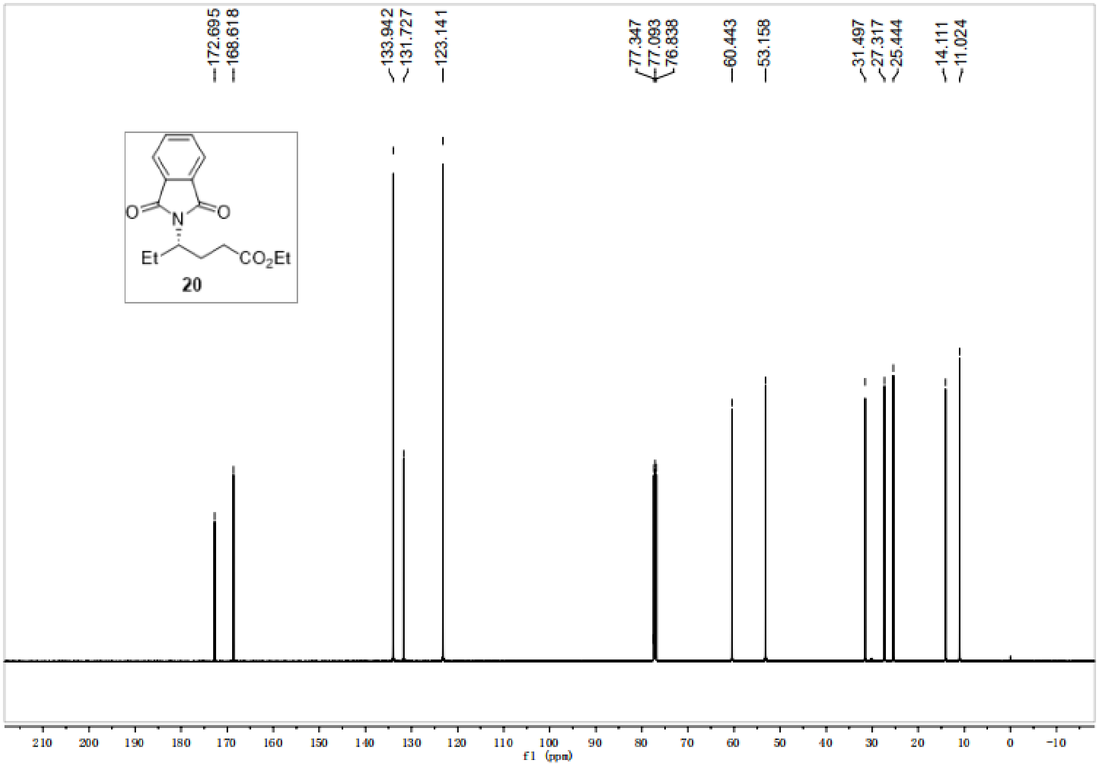

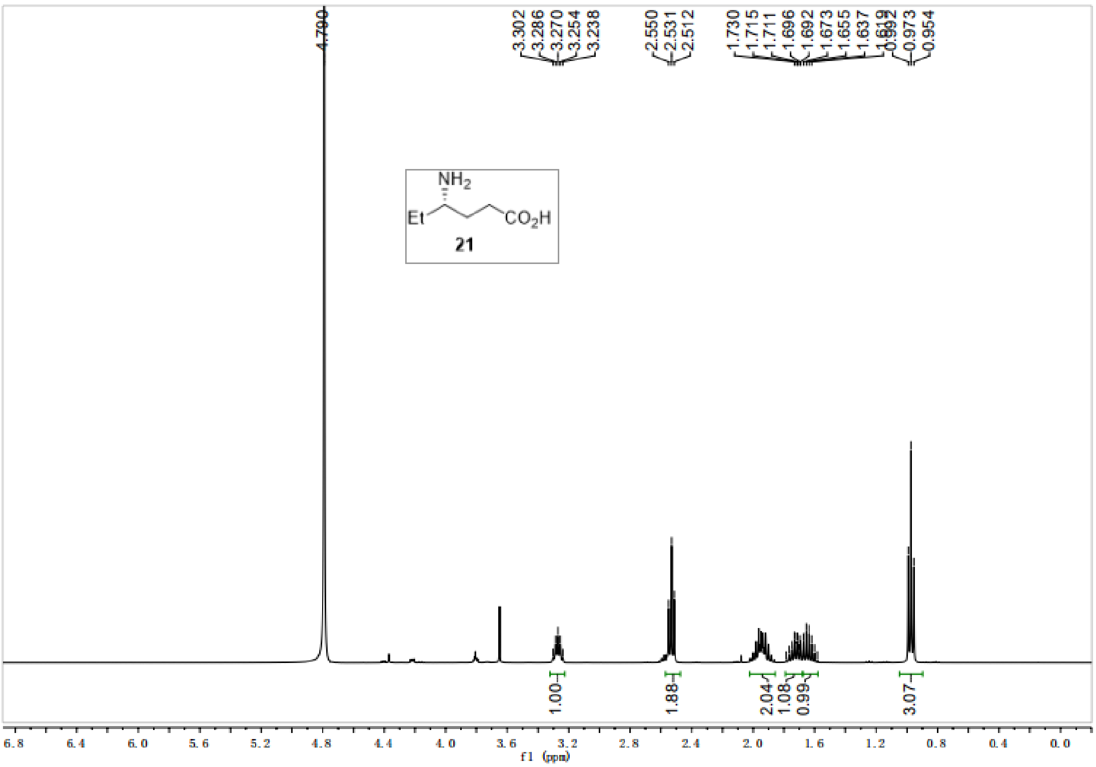
4.790
3.302
3.286
3.270
3.254
3.238
2.550
2.531
2.512
1.730
1.715
1.711
1.696
1.692
1.673
1.655
1.637
0.973
0.954
NH2
Et
CO2H
21
1.00
1.88
2.04
1.08
0.99
3.07
f1 (ppm)

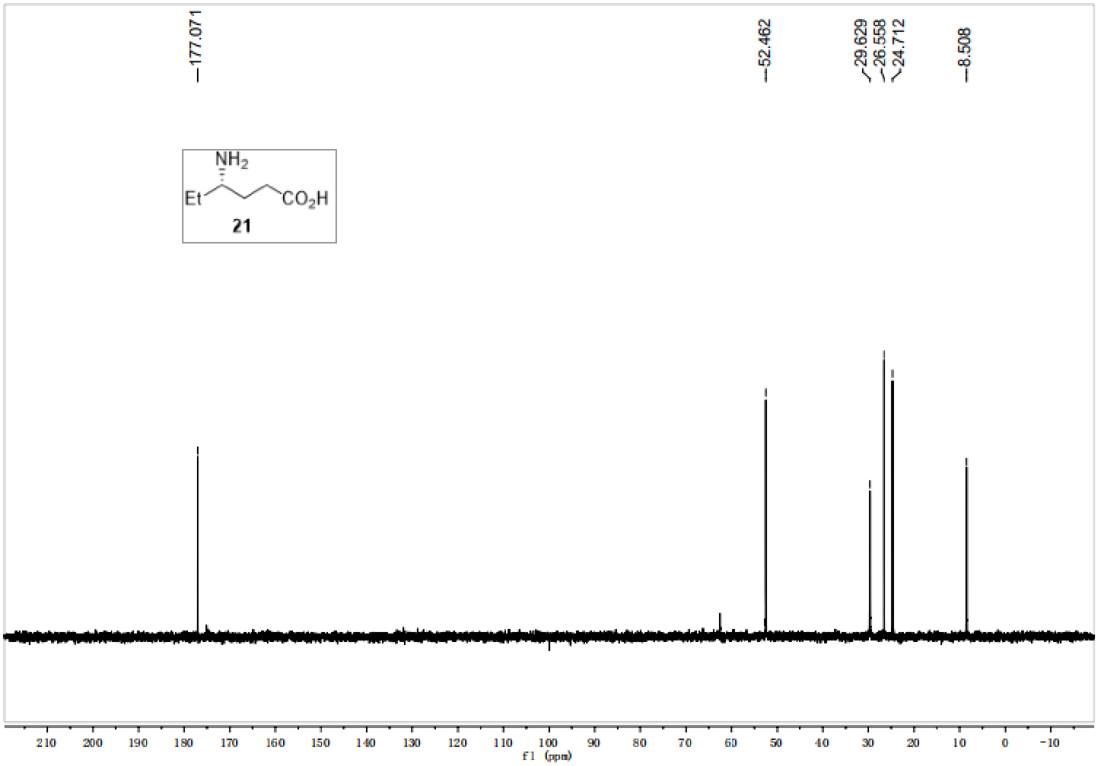
177.071
52.462
29.629
26.558
24.712
8.508
NH2
Et
CO2H
21
f1 (ppm)

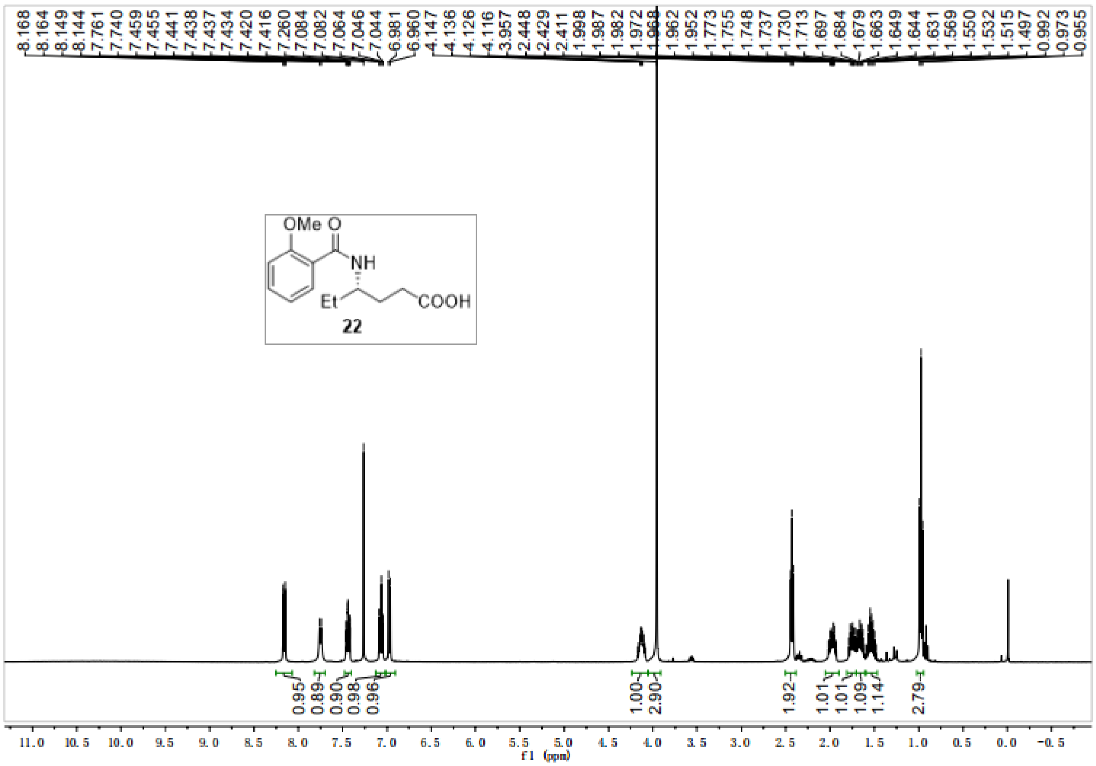

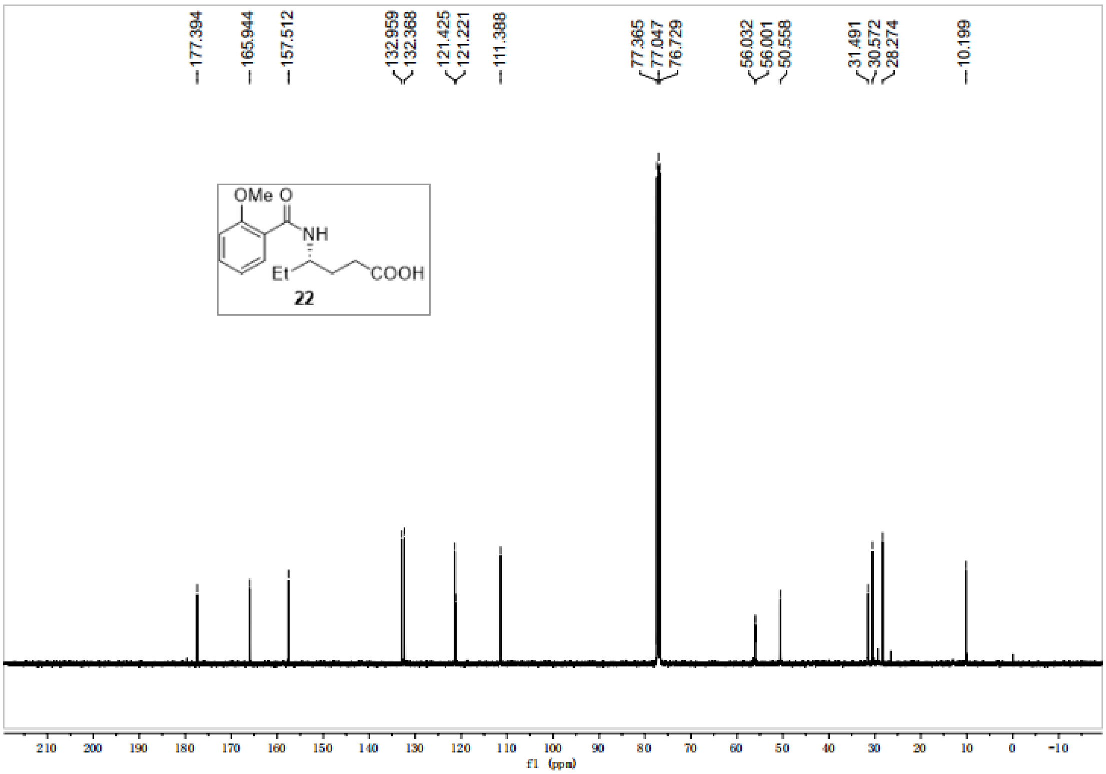

Supplement: Supplementary file 1 [file SC-010-C9SC04073K-s001.pdf]
